# Supplementary material for: Cre-assisted fine-mapping of neural circuits using orthogonal split inteins
Source: eLife. 2020 Apr 14;9:e53041. doi: 10.7554/eLife.53041 (PMC7217698; doi:10.7554/eLife.53041)
Supplement: Supplementary file 4. [file elife-53041-supp4.docx]

**HJP-176-IVS-Syn21-Cre_A_-gp41-1^N^-WPREw**

1 TAAGAAACCA TTATTATCAT GACATTAACC TATAAAAATA GGCGTATCAC

51 GAGGCCCTTT CGTCTTCAAG AATTCGTTTA TCACAAGTTT GTACAAAAAA

101 GCTGAACGAG AAACGTAAAA TGATATAAAT ATCAATATAT TAAATTAGAT

151 TTTGCATAAA AAACAGACTA CATAATACTG TAAAACACAA CATATCCAGT

201 CACTATGGCG GCCGCATTAG GCACCCCAGG CTTTACACTT TATGCTTCCG

251 GCTCGTATAA TGTGTGGATT TTGAGTTAGG ATCCGTCGAG ATTTTCAGGA

301 GCTAAGGAAG CTAAAATGGA GAAAAAAATC ACTGGATATA CCACCGTTGA

351 TATATCCCAA TGGCATCGTA AAGAACATTT TGAGGCATTT CAGTCAGTTG

401 CTCAATGTAC CTATAACCAG ACCGTTCAGC TGGATATTAC GGCCTTTTTA

451 AAGACCGTAA AGAAAAATAA GCACAAGTTT TATCCGGCCT TTATTCACAT

501 TCTTGCCCGC CTGATGAATG CTCATCCGGA ATTCCGTATG GCAATGAAAG

551 ACGGTGAGCT GGTGATATGG GATAGTGTTC ACCCTTGTTA CACCGTTTTC

601 CATGAGCAAA CTGAAACGTT TTCATCGCTC TGGAGTGAAT ACCACGACGA

651 TTTCCGGCAG TTTCTACACA TATATTCGCA AGATGTGGCG TGTTACGGTG

701 AAAACCTGGC CTATTTCCCT AAAGGGTTTA TTGAGAATAT GTTTTTCGTC

751 TCAGCCAATC CCTGGGTGAG TTTCACCAGT TTTGATTTAA ACGTGGCCAA

801 TATGGACAAC TTCTTCGCCC CCGTTTTCAC CATGGGCAAA TATTATACGC

851 AAGGCGACAA GGTGCTGATG CCGCTGGCGA TTCAGGTTCA TCATGCCGTT

901 TGTGATGGCT TCCATGTCGG CAGAATGCTT AATGAATTAC AACAGTACTG

951 CGATGAGTGG CAGGGCGGGG CGTAAACGCG TGGATCCGGC TTACTAAAAG

1001 CCAGATAACA GTATGCGTAT TTGCGCGCTG ATTTTTGCGG TATAAGAATA

1051 TATACTGATA TGTATACCCG AAGTATGTCA AAAAGAGGTA TGCTATGAAG

1101 CAGCGTATTA CAGTGACAGT TGACAGCGAC AGCTATCAGT TGCTCAAGGC

1151 ATATATGATG TCAATATCTC CGGTCTGGTA AGCACAACCA TGCAGAATGA

1201 AGCCCGTCGT CTGCGTGCCG AACGCTGGAA AGCGGAAAAT CAGGAAGGGA

1251 TGGCTGAGGT CGCCCGGTTT ATTGAAATGA ACGGCTCTTT TGCTGACGAG

1301 AACAGGGGCT GGTGAAATGC AGTTTAAGGT TTACACCTAT AAAAGAGAGA

1351 GCCGTTATCG TCTGTTTGTG GATGTACAGA GTGATATTAT TGACACGCCC

1401 GGGCGACGGA TGGTGATCCC CCTGGCCAGT GCACGTCTGC TGTCAGATAA

1451 AGTCTCCCGT GAACTTTACC CGGTGGTGCA TATCGGGGAT GAAAGCTGGC

1501 GCATGATGAC CACCGATATG GCCAGTGTGC CGGTCTCCGT TATCGGGGAA

1551 GAAGTGGCTG ATCTCAGCCA CCGCGAAAAT GACATCAAAA ACGCCATTAA

1601 CCTGATGTTC TGGGGAATAT AAATGTCAGG CTCCCTTATA CACAGCCAGT

1651 CTGCAGGTCG ACCATAGTGA CTGGATATGT TGTGTTTTAC AGTATTATGT

1701 AGTCTGTTTT TTATGCAAAA TCTAATTTAA TATATTGATA TTTATATCAT

1751 TTTACGTTTC TCGTTCAGCT TTCTTGTACA AAGTGGTGAT AAACGGCCGG

1801 CCAGATCTGG TACCGCTAGA AAAAGGTAGG TTCAACCACT GATGCCTAGG

1851 CACACCGAAA CGACTAACCC TAATTCTTAT CCTTTACTTC AGGGCTAGCA

1901 ACTTAAAAAA AAAAATCAAA ATGAGTAACT TGCTCACGGT ACACCAAAAT

1951 TTGCCGGCCC TGCCTGTGGA CGCCACGTCC GACGAGGTCC GAAAGAATCT

2001 GATGGACATG TTCCGTGACA GGCAGGCCTT TAGCGAGCAC ACCTGGAAGA

2051 TGCTGCTCTC GGTGTGCAGG TCCTGGGCCG CTTGGTGCAA GCTGAACAAC

2101 CGAAAGTGGT TTCCCGCGGA GCCCGAGGAC GTCCGCGATT ATCTGCTGTA

2151 CCTGCAGGCT CGCGGACTGG CCGTCAAAAC AATACAGCAA CATTTGGGTC

2201 AGCTGAACAT GCTCCACAGG CGCAGCGGAC TGCCCCGGCC ATCCGATTGC

2251 CTGGATCTGA AGACCCAGGT GCAGACCCCC CAGGGCATGA AGGAGATCAG

2301 CAACATCCAG GTGGGCGATC TGGTGCTGAG CAACACCGGC TACAACGAGG

2351 TGCTGAACGT GTTCCCCAAG AGCAAGAAGA AGAGCTACAA GATCACCCTG

2401 GAGGATGGCA AGGAGATCAT CTGCAGCGAG GAGCACCTGT TCCCCACCCA

2451 GACCGGCGAG ATGAACATCA GCGGCGGCCT GAAGGAGGGC ATGTGCCTGT

2501 ACGTGAAGGA GTAAACCGGT GTTTAAACAA TCAACCTCTG GATTACAAAA

2551 TTTGTGAAAG ATTGACTGGT ATTCTTAACT ATGTTGCTCC TTTTACGCTA

2601 TGTGGATACG CTGCTTTAAT GCCTTTGTAT CATGCTATTG CTTCCCGTAT

2651 GGCTTTCATT TTCTCCTCCT TGTATAAATC CTGGTTGCTG TCTCTTTATG

2701 AGGAGTTGTG GCCCGTTGTC AGGCAACGTG GCGTGGTGTG CACTGTGTTT

2751 GCTGACGCAA CCCCCACTGG TTGGGGCATT GCCACCACCT GTCAGCTCCT

2801 TTCCGGGACT TTCGCTTTCC CCCTCCCTAT TGCCACGGCG GAACTCATCG

2851 CCGCCTGCCT TGCCCGCTGC TGGACAGGGG CTCGGCTGTT GGGCACTGAC

2901 AATTCCGTGG TGTTGTCGGG GAAATCATCG TCCTTTCCTT GGCTGCTCGC

2951 CTGTGTTGCC ACCTGGATTC TGCGCGGGAC GTCCTTCTGC TACGTCCCTT

3001 CGGCCCTCAA TCCAGCGGAC CTTCCTTCCC GCGGCCTGCT GCCGGCTCTG

3051 CGGCCTCTTC CGCGTCTTCG CCTTCGCCCT CAGACGAGTC GGATCTCCCT

3101 TTGGGCCGCC TCCCCGCCTG AAGCTTATCG ATACCGTCGA CTAAAGCCAA

3151 ATAGAAAATT ATTCAGTTCC TGGCTTAAGT TTTTAAAAGT GATATTATTT

3201 ATTTGGTTGT AACCAACCAA AAGAATGTAA ATAACTAATA CATAATTATG

3251 TTAGTTTTAA GTTAGCAACA AATTGATTTT AGCTATATTA GCTACTTGGT

3301 TAATAAATAG AATATATTTA TTTAAAGATA ATTGCGTTTT TATTGTCAGG

3351 GAGTGAGTTT GCTTAAAAAC TCGTTTAGAT CCACTAGTTC TAGAGCGGCC

3401 GCTGGCCACG GGTGCGCATG ATCGTGCTCC TGTCGTTGAG GACCCGGCTA

3451 GGCTGGCGGG GTTGCCTTAC TGGTTAGCAG AATGAATCAC CGATACGCGA

3501 GCGAACGTGA AGCGACTGCT GCTGCAAAAC GTCTGCGACC TGAGCAACAA

3551 CATGAATGGT CTTCGGTTTC CGTGTTTCGT AAAGTCTGGA AACGCGGAAG

3601 TCAGCGCCCT GCACCATTAT GTTCCGGAGG CGCGCCCTAG TTCCAGTGAA

3651 ATCCAAGCAT TTTCTAAATT AAATGTATTC TTATTATTAT AGTTGTTATT

3701 TTTGATATAT ATAAACAACA CTATTATGCC CACCATTTTT TTGAGATGCA

3751 TCTACACAAG GAACAAACAC TGGATGTCAC TTTCAGTTCA AATTGTAACG

3801 CTAATCACTC CGAACAGGTC ACAAAAAATT ACCTTAAAAA GTCATAATAT

3851 TAAATTAGAA TAAATATAGC TGTGAGGGAA ATATATACAA ATATATTGGA

3901 GCAAATAAAT TGTACATACA AATATTTATT ACTAATTTCT ATTGAGACGA

3951 AATGAACCAC TCGGAACCAT TTGAGCGAAC CGAATCGCGC GGAACTAACG

4001 ACAGTCGCTC CAAGGTCGTC GAACAAAAGG TGAATGTGTT GCGGAGAGCG

4051 GGTGGGAGAC AGCGAAAGAG CAACTACGAA ACGTGGTGTG GTGGAGGTGA

4101 ATTATGAAGA GGGCGCGCGA TTTGAAAAGT ATGTATATAA AAAATATATC

4151 CCGGTGTTTT ATGTAGCGAT AAACGAGTTT TTGATGTAAG GTATGCAGGT

4201 GTGTAAGTCT TTTGGTTAGA AGACAAATCC AAAGTCTACT TGTGGGGATG

4251 TTCGAAGGGG AAATACTTGT ATTCTATAGG TCATATCTTG TTTTTATTGG

4301 CACAAATATA ATTACATTAG CTTTTTGAGG GGGCAATAAA CAGTAAACAC

4351 GATGGTAATA ATGGTAAAAA AAAAAAACAA GCAGTTATTT CGGATATATG

4401 TCGGCTACTC CTTGCGTCGG GCCCGAAGTC TTAGAGCCAG ATATGCGAGC

4451 ACCCGGAAGC TCACGATGAG AATGGCCAGA CCCACGTAGT CCAGCGGCAG

4501 ATCGGCGGCG GAGAAGTTAA GCGTCTCCAG GATGACCTTG CCCGAACTGG

4551 GGCACGTGGT GTTCGACGAT GTGCAGCTAA TTTCGCCCGG CTCCACGTCC

4601 GCCCATTGGT TAATCAGCAG ACCCTCGTTG GCGTAACGGA ACCATGAGAG

4651 GTACGACAAC CATTTGAGGT ATACTGGCAC CGAGCCCGAG TTCAAGAAGA

4701 AGCCGCCAAA GAGCAGGAAT GGTATGATAA CCGGCGGACC CACAGACAGC

4751 GCCATCGAGG TCGAGGAGCT GGCGCAGGAT ATTAGATATC CGAAGGACGT

4801 TGACACATTG GCCACCAGAG TGACCAGCGC CAGGCAGTTG AAGAAGTGCA

4851 GCACTCCGGC CCGCAGTCCG ATCATCGGAT AGGCAATCGC CGTGAAGACC

4901 AGTGGCACTG TGAGAAAAAG CGGCAATTCG GCAATCGTTT TGCCCAGAAA

4951 GTATGTGTCA CAGCGATAAA GTCGACTTCG GGCCTCCCTC ATAAAAACTG

5001 GCAGCTCTGA GGTGAACACC TAAATCGAAT CGATTCATTA GAAAGTTAGT

5051 AAATTATTGA AATGCAAATG TATTCTAAAC ATGACTTACA TTTATCGTGG

5101 CAAAGACGTT TTGAAAGGTC ATGTTGGTCA GGAAGAGGAA GATGGCTCCG

5151 TTGATATTCA TCACACCCAC TTGCGTGAGT TGTTGGCCCA AAAAGATGAG

5201 GCCAATCAAG ATGGCAACCA TCTGCAAATT AAAATGTTAC TCGCATCTCA

5251 TTAATATTCG CGAGTTAAAT GAAATTTATT TATCTTCTGC AAAACTATAA

5301 ACTATACATC TCATTGAAAA AAACTAAGAA GGGTGTGGAA TCAGGCAATT

5351 CTATCTAAAA TCTAGCGAAT TTGTTTCCAA GAATTGTAAG CGTTATATCA

5401 TTTGTTTCCA CTGGAACCAC TCACCGTTGT CTGAATAAGT CGCACTTTTA

5451 CGAGGAGTGG TTCCTTGAGC ACCGACAGCC AGGATCGCCA CAGGACCGCC

5501 CGGAACTGCA TGAACCAGGT GGCCTTGTAG GTGTACCCAT TCTCCGGCTG

5551 CTCCAGTGGC TTCTCCAGAT TTTTGGTGGC CAACAACTGC TCCATATCCC

5601 GGGCTACTTT GCTAATGGCA AAATTGTCGC ATATCTTGGC GATCCGATCA

5651 CGGGACTCGA TCTCCCGTCC GGGCACAACG GCCAACACCT GTACGTAAAA

5701 GTCCGCCGGA TTGTAGTTGG TAGGACACTG GGCACCCACG CTGGATAGGA

5751 GTTGAGATGT TATGTAATAC TAGATACCCT TAATAAACAC ATCGAACTCA

5801 CTAGGAAAAG AAGTCGACGG CTTCGCTGGG AGTGCCCAAG AAAGCTACCC

5851 TGCCCTCGGC CATCAGAAGG ATCTTGTCAA AGAGCTCAAA CAGCTCGGAA

5901 GACGGCTGAT GAATGGTCAG GATGACGGTC TTGCCCTTCT GCGACAGCTT

5951 CTTCAGCACC TGGACGACGC TGTGGGCGGT AAAGGAGTCC AGTCCGGAGG

6001 TGGGCTCATC GCAGATCAGA AGCGGCGGAT CGGTTAGAGC CTCGGAGGCG

6051 AATGCCAGAC GCTTCCTTTC TCCGCCGGAC AGACCTTTCA CCCTGCCGGG

6101 CACACCGATG ATCGTGTGCT GACATTTGCT GAGCGAAAGC TCCTGGATCA

6151 CCTGATCCAC GCGGGCCACT CGCTGCCGAT AGGTCAGATG TCGTGGCATC

6201 CGCACCATGG CTTGGAAAAT CAGGTGTTCC CTGGCCGTTA GGGAGCCGAT

6251 AAAGAGGTCA TCCTGCTGGA CATAGGCGCA CCTGGCCTGC ATCTCCTTGG

6301 CGTCCACAGG TTGGCCATTG AGCAGTCGCA TCCCGGATGG CGATACTTGG

6351 ATGCCCTGCG GCGATCGAAA GGCAAGGGCA TTCAGCAGGG TCGTCTTTCC

6401 GGCACCGGAA CTGCCCATCA CGGCCAAAAG TTCGCCCGGA TAGGCCACGC

6451 CGCAAACTGA GTTTCAAATT GGTAATTGGA CCCTTTATTA AGATTTCACA

6501 CAGATCAGCC GACTGCGAAT AGAAACTCAC CGTTCTTGAG CAAATGTTTC

6551 CTGGGCGCCG GTATGTGTCG CTCGTTGCAG AATAGTCCGC GTGTCCGGTT

6601 GACCAGCTGC CGCCATCCGG AGCCCGGCTG ATTGACCGCC CCAAAGATGT

6651 CCATATTGTG CCAGGCATAG GTGAGGTTCT CGGCTAGTTG GCCGCTCCCT

6701 GAACCGGAGT CCTCCGGCGG ACTGGGTGGC CGGAGCGTGC CGTAGTTTTT

6751 GGCCTGCCCG AAGCCCTGGT TAATGCAGCT CTGCGAAGCC GCTCCGCTGT

6801 CACCCTGCAA TGATAGGGGA TCTCAAATAT CAACTACTAG CGTTATGCTC

6851 ATCTAACCCC GAACAAAAAG TACCCCGAAG TATCCTACGA AGTAGGTTTA

6901 TACTTTTATT TATTTTTTGT GCATCTAGGA TCAGCTTAAA ATATCTGGTT

6951 GTTATATTTT TTGTAAAAAA GAATATAGTC GAAAATGAAT GCCTTTAGAT

7001 GTCTTGATCA TGATATGATC TCAAAAATTG TCTTATATAG CGAGAACAGC

7051 TACCAGAATA ATCTGTTTCG TGTCACTATT TGTTTGTGCA ATTGCGGTTT

7101 GGGATTTTTG TGGGTCGCAG TTCTCACGCC GCATACAATT TGATGTTGCA

7151 ATCGCAGTTC CTATAGATCA AGTGAACTTA AGATGTATGC ACATGTACTA

7201 CTCACATTGT TCAGATGCTC GGCAGATGGG TGTTTGCTGC CTCCGCGAAT

7251 TAATAGCTCC TGATCCTCTT GGCCCATTGC CGGGATTTTT CACACTTTCC

7301 CCTGCTTACC CACCCAAAAC CAATCACCAC CCCAATCACT CAAAAAACAA

7351 ACAAAAATAA GAAGCGAGAG GAGTTTTGGC ACAGCACTTT GTGTTTAATT

7401 GATGGCGTAA ACCGCTTGGA GCTTCGTCAC GAAACCGCTG ACAAAATGCA

7451 ACTGAAGGCG GACATTGACG CTACGTAACG CTACAAACGG TGGCGAAAGA

7501 GATAGCGGAC GCAGCGGCGA AAGAGACGGC GATATTTCTG TGGACAGAGA

7551 AGGAGGCAAA CAGCGCTGAC TTTGAGTGGA ATGTCATTTT GAGTGAGAGG

7601 TAATCGAAAG AACCTGGTAC ATCAAATACC CTTGGATCGA AGTAAATTTA

7651 AAACTGATCA GATAAGTTCA ATGATATCCA GTGCAGTAAA AAAAAAAAAT

7701 GTTTTTTTTA TCTACTTTCC GCAAAAATGG GTTTTATTAA CTTACATACA

7751 TACTAGGCGC GCCCATATGT TCGGCTTGTC GACATGCCCG CCGTGACCGT

7801 CGAGAACCCG CTGACGCTGC CCCGCGTATC CGCACCCGCC GACGCCGTCG

7851 CACGTCCCGT GCTCACCGTG ACCACCGCGC CCAGCGGTTT CGAGGGCGAG

7901 GGCTTCCCGG TGCGCCGCGC GTTCGCCGGG ATCAACTACC GCCACCTCGA

7951 CCCGTTCATC ATGATGGACC AGATGGGTGA GGTGGAGTAC GCGCCCGGGG

8001 AGCCCAAGGG CACGCCCTGG CACCCGCACC GCGGCTTCGA GACCGTGACC

8051 TACATCGTCG ACGGTAACAT GTGAGCAAAA GGCCAGCAAA AGGCCAGGAA

8101 CCGTAAAAAG GCCGCGTTGC TGGCGTTTTT CCATAGGCTC CGCCCCCCTG

8151 ACGAGCATCA CAAAAATCGA CGCTCAAGTC AGAGGTGGCG AAACCCGACA

8201 GGACTATAAA GATACCAGGC GTTTCCCCCT GGAAGCTCCC TCGTGCGCTC

8251 TCCTGTTCCG ACCCTGCCGC TTACCGGATA CCTGTCCGCC TTTCTCCCTT

8301 CGGGAAGCGT GGCGCTTTCT CATAGCTCAC GCTGTAGGTA TCTCAGTTCG

8351 GTGTAGGTCG TTCGCTCCAA GCTGGGCTGT GTGCACGAAC CCCCCGTTCA

8401 GCCCGACCGC TGCGCCTTAT CCGGTAACTA TCGTCTTGAG TCCAACCCGG

8451 TAAGACACGA CTTATCGCCA CTGGCAGCAG CCACTGGTAA CAGGATTAGC

8501 AGAGCGAGGT ATGTAGGCGG TGCTACAGAG TTCTTGAAGT GGTGGCCTAA

8551 CTACGGCTAC ACTAGAAGAA CAGTATTTGG TATCTGCGCT CTGCTGAAGC

8601 CAGTTACCTT CGGAAAAAGA GTTGGTAGCT CTTGATCCGG CAAACAAACC

8651 ACCGCTGGTA GCGGTGGTTT TTTTGTTTGC AAGCAGCAGA TTACGCGCAG

8701 AAAAAAAGGA TCTCAAGAAG ATCCTTTGAT CTTTTCTACG GGGTCTGACG

8751 CTCAGTGGAA CGAAAACTCA CGTTAAGGGA TTTTGGTCAT GAGATTATCA

8801 AAAAGGATCT TCACCTAGAT CCTTTTAAAT TAAAAATGAA GTTTTAAATC

8851 AATCTAAAGT ATATATGAGT AAACTTGGTC TGACAGTTAC CAATGCTTAA

8901 TCAGTGAGGC ACCTATCTCA GCGATCTGTC TATTTCGTTC ATCCATAGTT

8951 GCCTGACTCC CCGTCGTGTA GATAACTACG ATACGGGAGG GCTTACCATC

9001 TGGCCCCAGT GCTGCAATGA TACCGCGAGA CCCACGCTCA CCGGCTCCAG

9051 ATTTATCAGC AATAAACCAG CCAGCCGGAA GGGCCGAGCG CAGAAGTGGT

9101 CCTGCAACTT TATCCGCCTC CATCCAGTCT ATTAATTGTT GCCGGGAAGC

9151 TAGAGTAAGT AGTTCGCCAG TTAATAGTTT GCGCAACGTT GTTGCCATTG

9201 CTACAGGCAT CGTGGTGTCA CGCTCGTCGT TTGGTATGGC TTCATTCAGC

9251 TCCGGTTCCC AACGATCAAG GCGAGTTACA TGATCCCCCA TGTTGTGCAA

9301 AAAAGCGGTT AGCTCCTTCG GTCCTCCGAT CGTTGTCAGA AGTAAGTTGG

9351 CCGCAGTGTT ATCACTCATG GTTATGGCAG CACTGCATAA TTCTCTTACT

9401 GTCATGCCAT CCGTAAGATG CTTTTCTGTG ACTGGTGAGT ACTCAACCAA

9451 GTCATTCTGA GAATAGTGTA TGCGGCGACC GAGTTGCTCT TGCCCGGCGT

9501 CAATACGGGA TAATACCGCG CCACATAGCA GAACTTTAAA AGTGCTCATC

9551 ATTGGAAAAC GTTCTTCGGG GCGAAAACTC TCAAGGATCT TACCGCTGTT

9601 GAGATCCAGT TCGATGTAAC CCACTCGTGC ACCCAACTGA TCTTCAGCAT

9651 CTTTTACTTT CACCAGCGTT TCTGGGTGAG CAAAAACAGG AAGGCAAAAT

9701 GCCGCAAAAA AGGGAATAAG GGCGACACGG AAATGTTGAA TACTCATACT

9751 CTTCCTTTTT CAATATTATT GAAGCATTTA TCAGGGTTAT TGTCTCATGA

9801 GCGGATACAT ATTTGAATGT ATTTAGAAAA ATAAACAAAT AGGGGTTCCG

9851 CGCACATTTC CCCGAAAAGT GCCACCTGAG TC

**HJP-180-IVS-Syn21-gp41-1^C^-Cre_B_-NrdJ-1^N^-WPREw**

1 CTGAGTCTAA GAAACCATTA TTATCATGAC ATTAACCTAT AAAAATAGGC

51 GTATCACGAG GCCCTTTCGT CTTCAAGAAT TCGTTTATCA CAAGTTTGTA

101 CAAAAAAGCT GAACGAGAAA CGTAAAATGA TATAAATATC AATATATTAA

151 ATTAGATTTT GCATAAAAAA CAGACTACAT AATACTGTAA AACACAACAT

201 ATCCAGTCAC TATGGCGGCC GCATTAGGCA CCCCAGGCTT TACACTTTAT

251 GCTTCCGGCT CGTATAATGT GTGGATTTTG AGTTAGGATC CGTCGAGATT

301 TTCAGGAGCT AAGGAAGCTA AAATGGAGAA AAAAATCACT GGATATACCA

351 CCGTTGATAT ATCCCAATGG CATCGTAAAG AACATTTTGA GGCATTTCAG

401 TCAGTTGCTC AATGTACCTA TAACCAGACC GTTCAGCTGG ATATTACGGC

451 CTTTTTAAAG ACCGTAAAGA AAAATAAGCA CAAGTTTTAT CCGGCCTTTA

501 TTCACATTCT TGCCCGCCTG ATGAATGCTC ATCCGGAATT CCGTATGGCA

551 ATGAAAGACG GTGAGCTGGT GATATGGGAT AGTGTTCACC CTTGTTACAC

601 CGTTTTCCAT GAGCAAACTG AAACGTTTTC ATCGCTCTGG AGTGAATACC

651 ACGACGATTT CCGGCAGTTT CTACACATAT ATTCGCAAGA TGTGGCGTGT

701 TACGGTGAAA ACCTGGCCTA TTTCCCTAAA GGGTTTATTG AGAATATGTT

751 TTTCGTCTCA GCCAATCCCT GGGTGAGTTT CACCAGTTTT GATTTAAACG

801 TGGCCAATAT GGACAACTTC TTCGCCCCCG TTTTCACCAT GGGCAAATAT

851 TATACGCAAG GCGACAAGGT GCTGATGCCG CTGGCGATTC AGGTTCATCA

901 TGCCGTTTGT GATGGCTTCC ATGTCGGCAG AATGCTTAAT GAATTACAAC

951 AGTACTGCGA TGAGTGGCAG GGCGGGGCGT AAACGCGTGG ATCCGGCTTA

1001 CTAAAAGCCA GATAACAGTA TGCGTATTTG CGCGCTGATT TTTGCGGTAT

1051 AAGAATATAT ACTGATATGT ATACCCGAAG TATGTCAAAA AGAGGTATGC

1101 TATGAAGCAG CGTATTACAG TGACAGTTGA CAGCGACAGC TATCAGTTGC

1151 TCAAGGCATA TATGATGTCA ATATCTCCGG TCTGGTAAGC ACAACCATGC

1201 AGAATGAAGC CCGTCGTCTG CGTGCCGAAC GCTGGAAAGC GGAAAATCAG

1251 GAAGGGATGG CTGAGGTCGC CCGGTTTATT GAAATGAACG GCTCTTTTGC

1301 TGACGAGAAC AGGGGCTGGT GAAATGCAGT TTAAGGTTTA CACCTATAAA

1351 AGAGAGAGCC GTTATCGTCT GTTTGTGGAT GTACAGAGTG ATATTATTGA

1401 CACGCCCGGG CGACGGATGG TGATCCCCCT GGCCAGTGCA CGTCTGCTGT

1451 CAGATAAAGT CTCCCGTGAA CTTTACCCGG TGGTGCATAT CGGGGATGAA

1501 AGCTGGCGCA TGATGACCAC CGATATGGCC AGTGTGCCGG TCTCCGTTAT

1551 CGGGGAAGAA GTGGCTGATC TCAGCCACCG CGAAAATGAC ATCAAAAACG

1601 CCATTAACCT GATGTTCTGG GGAATATAAA TGTCAGGCTC CCTTATACAC

1651 AGCCAGTCTG CAGGTCGACC ATAGTGACTG GATATGTTGT GTTTTACAGT

1701 ATTATGTAGT CTGTTTTTTA TGCAAAATCT AATTTAATAT ATTGATATTT

1751 ATATCATTTT ACGTTTCTCG TTCAGCTTTC TTGTACAAAG TGGTGATAAA

1801 CGGCCGGCCA GATCTGGTAC CGCTAGAAAA AGGTAGGTTC AACCACTGAT

1851 GCCTAGGCAC ACCGAAACGA CTAACCCTAA TTCTTATCCT TTACTTCAGG

1901 GCTAGCAACT TAAAAAAAAA AATCAAAATG ATGCTGAAGA AGATCCTGAA

1951 GATCGAGGAG CTGGATGAGC GCGAGCTGAT CGATATCGAG GTGAGCGGCA

2001 ACCACCTGTT CTACGCCAAC GATATCCTGA CCCACAACAG CAATGCAGTG

2051 AGCCTGGTAA TGCGTCGAAT ACGCAAGGAA AACGTGGACG CCGGCGAGAG

2101 GGCCAAACAG GCGTTGGCTT TCGAACGCAC CGATTTCGAT CAGGTGCGGT

2151 CGTTGATGGA GAACAGCGAT CGATGCCAGG ACATCCGGAA CTTGGCGTTT

2201 TTGGGCATTG CCTATAACAC CCTCCTTCGC ATCGCCGAGA TAGCGCGTAT

2251 CCGAGTTAAA GACATTTCCC GTACAGACGG AGGCCGCATG CTTATTCACA

2301 TCGGACGCAC GAAAACCCTG GTCAGCACTG CTGGCGTGGA GAAAGCACTT

2351 TCCCTGGGTG TGACGAAATT GGTCGAGCGA TGGATCAGTG TGAGTGGCGT

2401 CGCCGATGAC CCCAACAATT ATCTGTTCTG CAGGGTGCGC AAAAATGGAG

2451 TGGCAGCCCC CTGCCTGGTG GGCAGCAGCG AGATCATCAC CCGCAACTAC

2501 GGCAAGACCA CCATCAAGGA GGTGGTGGAG ATCTTCGATA ACGATAAGAA

2551 CATCCAGGTG CTGGCCTTCA ACACCCACAC CGATAACATC GAGTGGGCCC

2601 CCATCAAGGC CGCCCAGCTG ACCCGCCCCA ACGCCGAGCT GGTGGAGCTG

2651 GAGATCGATA CCCTGCACGG CGTGAAGACC ATCCGCTGCA CCCCCGATCA

2701 CCCCGTGTAC ACCAAGAACC GCGGCTACGT GCGCGCCGAT GAGCTGACCG

2751 ATGATGATGA GCTGGTGGTG GCCATCTAAA CCGGTGTTTA AACAATCAAC

2801 CTCTGGATTA CAAAATTTGT GAAAGATTGA CTGGTATTCT TAACTATGTT

2851 GCTCCTTTTA CGCTATGTGG ATACGCTGCT TTAATGCCTT TGTATCATGC

2901 TATTGCTTCC CGTATGGCTT TCATTTTCTC CTCCTTGTAT AAATCCTGGT

2951 TGCTGTCTCT TTATGAGGAG TTGTGGCCCG TTGTCAGGCA ACGTGGCGTG

3001 GTGTGCACTG TGTTTGCTGA CGCAACCCCC ACTGGTTGGG GCATTGCCAC

3051 CACCTGTCAG CTCCTTTCCG GGACTTTCGC TTTCCCCCTC CCTATTGCCA

3101 CGGCGGAACT CATCGCCGCC TGCCTTGCCC GCTGCTGGAC AGGGGCTCGG

3151 CTGTTGGGCA CTGACAATTC CGTGGTGTTG TCGGGGAAAT CATCGTCCTT

3201 TCCTTGGCTG CTCGCCTGTG TTGCCACCTG GATTCTGCGC GGGACGTCCT

3251 TCTGCTACGT CCCTTCGGCC CTCAATCCAG CGGACCTTCC TTCCCGCGGC

3301 CTGCTGCCGG CTCTGCGGCC TCTTCCGCGT CTTCGCCTTC GCCCTCAGAC

3351 GAGTCGGATC TCCCTTTGGG CCGCCTCCCC GCCTGAAGCT TATCGATACC

3401 GTCGACTAAA GCCAAATAGA AAATTATTCA GTTCCTGGCT TAAGTTTTTA

3451 AAAGTGATAT TATTTATTTG GTTGTAACCA ACCAAAAGAA TGTAAATAAC

3501 TAATACATAA TTATGTTAGT TTTAAGTTAG CAACAAATTG ATTTTAGCTA

3551 TATTAGCTAC TTGGTTAATA AATAGAATAT ATTTATTTAA AGATAATTGC

3601 GTTTTTATTG TCAGGGAGTG AGTTTGCTTA AAAACTCGTT TAGATCCACT

3651 AGTTCTAGAG CGGCCGCTGG CCACGGGTGC GCATGATCGT GCTCCTGTCG

3701 TTGAGGACCC GGCTAGGCTG GCGGGGTTGC CTTACTGGTT AGCAGAATGA

3751 ATCACCGATA CGCGAGCGAA CGTGAAGCGA CTGCTGCTGC AAAACGTCTG

3801 CGACCTGAGC AACAACATGA ATGGTCTTCG GTTTCCGTGT TTCGTAAAGT

3851 CTGGAAACGC GGAAGTCAGC GCCCTGCACC ATTATGTTCC GGAGGCGCGC

3901 CCTAGTTCCA GTGAAATCCA AGCATTTTCT AAATTAAATG TATTCTTATT

3951 ATTATAGTTG TTATTTTTGA TATATATAAA CAACACTATT ATGCCCACCA

4001 TTTTTTTGAG ATGCATCTAC ACAAGGAACA AACACTGGAT GTCACTTTCA

4051 GTTCAAATTG TAACGCTAAT CACTCCGAAC AGGTCACAAA AAATTACCTT

4101 AAAAAGTCAT AATATTAAAT TAGAATAAAT ATAGCTGTGA GGGAAATATA

4151 TACAAATATA TTGGAGCAAA TAAATTGTAC ATACAAATAT TTATTACTAA

4201 TTTCTATTGA GACGAAATGA ACCACTCGGA ACCATTTGAG CGAACCGAAT

4251 CGCGCGGAAC TAACGACAGT CGCTCCAAGG TCGTCGAACA AAAGGTGAAT

4301 GTGTTGCGGA GAGCGGGTGG GAGACAGCGA AAGAGCAACT ACGAAACGTG

4351 GTGTGGTGGA GGTGAATTAT GAAGAGGGCG CGCGATTTGA AAAGTATGTA

4401 TATAAAAAAT ATATCCCGGT GTTTTATGTA GCGATAAACG AGTTTTTGAT

4451 GTAAGGTATG CAGGTGTGTA AGTCTTTTGG TTAGAAGACA AATCCAAAGT

4501 CTACTTGTGG GGATGTTCGA AGGGGAAATA CTTGTATTCT ATAGGTCATA

4551 TCTTGTTTTT ATTGGCACAA ATATAATTAC ATTAGCTTTT TGAGGGGGCA

4601 ATAAACAGTA AACACGATGG TAATAATGGT AAAAAAAAAA AACAAGCAGT

4651 TATTTCGGAT ATATGTCGGC TACTCCTTGC GTCGGGCCCG AAGTCTTAGA

4701 GCCAGATATG CGAGCACCCG GAAGCTCACG ATGAGAATGG CCAGACCCAC

4751 GTAGTCCAGC GGCAGATCGG CGGCGGAGAA GTTAAGCGTC TCCAGGATGA

4801 CCTTGCCCGA ACTGGGGCAC GTGGTGTTCG ACGATGTGCA GCTAATTTCG

4851 CCCGGCTCCA CGTCCGCCCA TTGGTTAATC AGCAGACCCT CGTTGGCGTA

4901 ACGGAACCAT GAGAGGTACG ACAACCATTT GAGGTATACT GGCACCGAGC

4951 CCGAGTTCAA GAAGAAGCCG CCAAAGAGCA GGAATGGTAT GATAACCGGC

5001 GGACCCACAG ACAGCGCCAT CGAGGTCGAG GAGCTGGCGC AGGATATTAG

5051 ATATCCGAAG GACGTTGACA CATTGGCCAC CAGAGTGACC AGCGCCAGGC

5101 AGTTGAAGAA GTGCAGCACT CCGGCCCGCA GTCCGATCAT CGGATAGGCA

5151 ATCGCCGTGA AGACCAGTGG CACTGTGAGA AAAAGCGGCA ATTCGGCAAT

5201 CGTTTTGCCC AGAAAGTATG TGTCACAGCG ATAAAGTCGA CTTCGGGCCT

5251 CCCTCATAAA AACTGGCAGC TCTGAGGTGA ACACCTAAAT CGAATCGATT

5301 CATTAGAAAG TTAGTAAATT ATTGAAATGC AAATGTATTC TAAACATGAC

5351 TTACATTTAT CGTGGCAAAG ACGTTTTGAA AGGTCATGTT GGTCAGGAAG

5401 AGGAAGATGG CTCCGTTGAT ATTCATCACA CCCACTTGCG TGAGTTGTTG

5451 GCCCAAAAAG ATGAGGCCAA TCAAGATGGC AACCATCTGC AAATTAAAAT

5501 GTTACTCGCA TCTCATTAAT ATTCGCGAGT TAAATGAAAT TTATTTATCT

5551 TCTGCAAAAC TATAAACTAT ACATCTCATT GAAAAAAACT AAGAAGGGTG

5601 TGGAATCAGG CAATTCTATC TAAAATCTAG CGAATTTGTT TCCAAGAATT

5651 GTAAGCGTTA TATCATTTGT TTCCACTGGA ACCACTCACC GTTGTCTGAA

5701 TAAGTCGCAC TTTTACGAGG AGTGGTTCCT TGAGCACCGA CAGCCAGGAT

5751 CGCCACAGGA CCGCCCGGAA CTGCATGAAC CAGGTGGCCT TGTAGGTGTA

5801 CCCATTCTCC GGCTGCTCCA GTGGCTTCTC CAGATTTTTG GTGGCCAACA

5851 ACTGCTCCAT ATCCCGGGCT ACTTTGCTAA TGGCAAAATT GTCGCATATC

5901 TTGGCGATCC GATCACGGGA CTCGATCTCC CGTCCGGGCA CAACGGCCAA

5951 CACCTGTACG TAAAAGTCCG CCGGATTGTA GTTGGTAGGA CACTGGGCAC

6001 CCACGCTGGA TAGGAGTTGA GATGTTATGT AATACTAGAT ACCCTTAATA

6051 AACACATCGA ACTCACTAGG AAAAGAAGTC GACGGCTTCG CTGGGAGTGC

6101 CCAAGAAAGC TACCCTGCCC TCGGCCATCA GAAGGATCTT GTCAAAGAGC

6151 TCAAACAGCT CGGAAGACGG CTGATGAATG GTCAGGATGA CGGTCTTGCC

6201 CTTCTGCGAC AGCTTCTTCA GCACCTGGAC GACGCTGTGG GCGGTAAAGG

6251 AGTCCAGTCC GGAGGTGGGC TCATCGCAGA TCAGAAGCGG CGGATCGGTT

6301 AGAGCCTCGG AGGCGAATGC CAGACGCTTC CTTTCTCCGC CGGACAGACC

6351 TTTCACCCTG CCGGGCACAC CGATGATCGT GTGCTGACAT TTGCTGAGCG

6401 AAAGCTCCTG GATCACCTGA TCCACGCGGG CCACTCGCTG CCGATAGGTC

6451 AGATGTCGTG GCATCCGCAC CATGGCTTGG AAAATCAGGT GTTCCCTGGC

6501 CGTTAGGGAG CCGATAAAGA GGTCATCCTG CTGGACATAG GCGCACCTGG

6551 CCTGCATCTC CTTGGCGTCC ACAGGTTGGC CATTGAGCAG TCGCATCCCG

6601 GATGGCGATA CTTGGATGCC CTGCGGCGAT CGAAAGGCAA GGGCATTCAG

6651 CAGGGTCGTC TTTCCGGCAC CGGAACTGCC CATCACGGCC AAAAGTTCGC

6701 CCGGATAGGC CACGCCGCAA ACTGAGTTTC AAATTGGTAA TTGGACCCTT

6751 TATTAAGATT TCACACAGAT CAGCCGACTG CGAATAGAAA CTCACCGTTC

6801 TTGAGCAAAT GTTTCCTGGG CGCCGGTATG TGTCGCTCGT TGCAGAATAG

6851 TCCGCGTGTC CGGTTGACCA GCTGCCGCCA TCCGGAGCCC GGCTGATTGA

6901 CCGCCCCAAA GATGTCCATA TTGTGCCAGG CATAGGTGAG GTTCTCGGCT

6951 AGTTGGCCGC TCCCTGAACC GGAGTCCTCC GGCGGACTGG GTGGCCGGAG

7001 CGTGCCGTAG TTTTTGGCCT GCCCGAAGCC CTGGTTAATG CAGCTCTGCG

7051 AAGCCGCTCC GCTGTCACCC TGCAATGATA GGGGATCTCA AATATCAACT

7101 ACTAGCGTTA TGCTCATCTA ACCCCGAACA AAAAGTACCC CGAAGTATCC

7151 TACGAAGTAG GTTTATACTT TTATTTATTT TTTGTGCATC TAGGATCAGC

7201 TTAAAATATC TGGTTGTTAT ATTTTTTGTA AAAAAGAATA TAGTCGAAAA

7251 TGAATGCCTT TAGATGTCTT GATCATGATA TGATCTCAAA AATTGTCTTA

7301 TATAGCGAGA ACAGCTACCA GAATAATCTG TTTCGTGTCA CTATTTGTTT

7351 GTGCAATTGC GGTTTGGGAT TTTTGTGGGT CGCAGTTCTC ACGCCGCATA

7401 CAATTTGATG TTGCAATCGC AGTTCCTATA GATCAAGTGA ACTTAAGATG

7451 TATGCACATG TACTACTCAC ATTGTTCAGA TGCTCGGCAG ATGGGTGTTT

7501 GCTGCCTCCG CGAATTAATA GCTCCTGATC CTCTTGGCCC ATTGCCGGGA

7551 TTTTTCACAC TTTCCCCTGC TTACCCACCC AAAACCAATC ACCACCCCAA

7601 TCACTCAAAA AACAAACAAA AATAAGAAGC GAGAGGAGTT TTGGCACAGC

7651 ACTTTGTGTT TAATTGATGG CGTAAACCGC TTGGAGCTTC GTCACGAAAC

7701 CGCTGACAAA ATGCAACTGA AGGCGGACAT TGACGCTACG TAACGCTACA

7751 AACGGTGGCG AAAGAGATAG CGGACGCAGC GGCGAAAGAG ACGGCGATAT

7801 TTCTGTGGAC AGAGAAGGAG GCAAACAGCG CTGACTTTGA GTGGAATGTC

7851 ATTTTGAGTG AGAGGTAATC GAAAGAACCT GGTACATCAA ATACCCTTGG

7901 ATCGAAGTAA ATTTAAAACT GATCAGATAA GTTCAATGAT ATCCAGTGCA

7951 GTAAAAAAAA AAAATGTTTT TTTTATCTAC TTTCCGCAAA AATGGGTTTT

8001 ATTAACTTAC ATACATACTA GGCGCGCCCA TATGTTCGGC TTGTCGACAT

8051 GCCCGCCGTG ACCGTCGAGA ACCCGCTGAC GCTGCCCCGC GTATCCGCAC

8101 CCGCCGACGC CGTCGCACGT CCCGTGCTCA CCGTGACCAC CGCGCCCAGC

8151 GGTTTCGAGG GCGAGGGCTT CCCGGTGCGC CGCGCGTTCG CCGGGATCAA

8201 CTACCGCCAC CTCGACCCGT TCATCATGAT GGACCAGATG GGTGAGGTGG

8251 AGTACGCGCC CGGGGAGCCC AAGGGCACGC CCTGGCACCC GCACCGCGGC

8301 TTCGAGACCG TGACCTACAT CGTCGACGGT AACATGTGAG CAAAAGGCCA

8351 GCAAAAGGCC AGGAACCGTA AAAAGGCCGC GTTGCTGGCG TTTTTCCATA

8401 GGCTCCGCCC CCCTGACGAG CATCACAAAA ATCGACGCTC AAGTCAGAGG

8451 TGGCGAAACC CGACAGGACT ATAAAGATAC CAGGCGTTTC CCCCTGGAAG

8501 CTCCCTCGTG CGCTCTCCTG TTCCGACCCT GCCGCTTACC GGATACCTGT

8551 CCGCCTTTCT CCCTTCGGGA AGCGTGGCGC TTTCTCATAG CTCACGCTGT

8601 AGGTATCTCA GTTCGGTGTA GGTCGTTCGC TCCAAGCTGG GCTGTGTGCA

8651 CGAACCCCCC GTTCAGCCCG ACCGCTGCGC CTTATCCGGT AACTATCGTC

8701 TTGAGTCCAA CCCGGTAAGA CACGACTTAT CGCCACTGGC AGCAGCCACT

8751 GGTAACAGGA TTAGCAGAGC GAGGTATGTA GGCGGTGCTA CAGAGTTCTT

8801 GAAGTGGTGG CCTAACTACG GCTACACTAG AAGAACAGTA TTTGGTATCT

8851 GCGCTCTGCT GAAGCCAGTT ACCTTCGGAA AAAGAGTTGG TAGCTCTTGA

8901 TCCGGCAAAC AAACCACCGC TGGTAGCGGT GGTTTTTTTG TTTGCAAGCA

8951 GCAGATTACG CGCAGAAAAA AAGGATCTCA AGAAGATCCT TTGATCTTTT

9001 CTACGGGGTC TGACGCTCAG TGGAACGAAA ACTCACGTTA AGGGATTTTG

9051 GTCATGAGAT TATCAAAAAG GATCTTCACC TAGATCCTTT TAAATTAAAA

9101 ATGAAGTTTT AAATCAATCT AAAGTATATA TGAGTAAACT TGGTCTGACA

9151 GTTACCAATG CTTAATCAGT GAGGCACCTA TCTCAGCGAT CTGTCTATTT

9201 CGTTCATCCA TAGTTGCCTG ACTCCCCGTC GTGTAGATAA CTACGATACG

9251 GGAGGGCTTA CCATCTGGCC CCAGTGCTGC AATGATACCG CGAGACCCAC

9301 GCTCACCGGC TCCAGATTTA TCAGCAATAA ACCAGCCAGC CGGAAGGGCC

9351 GAGCGCAGAA GTGGTCCTGC AACTTTATCC GCCTCCATCC AGTCTATTAA

9401 TTGTTGCCGG GAAGCTAGAG TAAGTAGTTC GCCAGTTAAT AGTTTGCGCA

9451 ACGTTGTTGC CATTGCTACA GGCATCGTGG TGTCACGCTC GTCGTTTGGT

9501 ATGGCTTCAT TCAGCTCCGG TTCCCAACGA TCAAGGCGAG TTACATGATC

9551 CCCCATGTTG TGCAAAAAAG CGGTTAGCTC CTTCGGTCCT CCGATCGTTG

9601 TCAGAAGTAA GTTGGCCGCA GTGTTATCAC TCATGGTTAT GGCAGCACTG

9651 CATAATTCTC TTACTGTCAT GCCATCCGTA AGATGCTTTT CTGTGACTGG

9701 TGAGTACTCA ACCAAGTCAT TCTGAGAATA GTGTATGCGG CGACCGAGTT

9751 GCTCTTGCCC GGCGTCAATA CGGGATAATA CCGCGCCACA TAGCAGAACT

9801 TTAAAAGTGC TCATCATTGG AAAACGTTCT TCGGGGCGAA AACTCTCAAG

9851 GATCTTACCG CTGTTGAGAT CCAGTTCGAT GTAACCCACT CGTGCACCCA

9901 ACTGATCTTC AGCATCTTTT ACTTTCACCA GCGTTTCTGG GTGAGCAAAA

9951 ACAGGAAGGC AAAATGCCGC AAAAAAGGGA ATAAGGGCGA CACGGAAATG

10001 TTGAATACTC ATACTCTTCC TTTTTCAATA TTATTGAAGC ATTTATCAGG

10051 GTTATTGTCT CATGAGCGGA TACATATTTG AATGTATTTA GAAAAATAAA

10101 CAAATAGGGG TTCCGCGCAC ATTTCCCCGA AAAGTGCCAC

**HJP-179-IVS-Syn21-NrdJ-1^C^-Cre_C_-WPREw**

1 CTAAGAAACC ATTATTATCA TGACATTAAC CTATAAAAAT AGGCGTATCA

51 CGAGGCCCTT TCGTCTTCAA GAATTCGTTT ATCACAAGTT TGTACAAAAA

101 AGCTGAACGA GAAACGTAAA ATGATATAAA TATCAATATA TTAAATTAGA

151 TTTTGCATAA AAAACAGACT ACATAATACT GTAAAACACA ACATATCCAG

201 TCACTATGGC GGCCGCATTA GGCACCCCAG GCTTTACACT TTATGCTTCC

251 GGCTCGTATA ATGTGTGGAT TTTGAGTTAG GATCCGTCGA GATTTTCAGG

301 AGCTAAGGAA GCTAAAATGG AGAAAAAAAT CACTGGATAT ACCACCGTTG

351 ATATATCCCA ATGGCATCGT AAAGAACATT TTGAGGCATT TCAGTCAGTT

401 GCTCAATGTA CCTATAACCA GACCGTTCAG CTGGATATTA CGGCCTTTTT

451 AAAGACCGTA AAGAAAAATA AGCACAAGTT TTATCCGGCC TTTATTCACA

501 TTCTTGCCCG CCTGATGAAT GCTCATCCGG AATTCCGTAT GGCAATGAAA

551 GACGGTGAGC TGGTGATATG GGATAGTGTT CACCCTTGTT ACACCGTTTT

601 CCATGAGCAA ACTGAAACGT TTTCATCGCT CTGGAGTGAA TACCACGACG

651 ATTTCCGGCA GTTTCTACAC ATATATTCGC AAGATGTGGC GTGTTACGGT

701 GAAAACCTGG CCTATTTCCC TAAAGGGTTT ATTGAGAATA TGTTTTTCGT

751 CTCAGCCAAT CCCTGGGTGA GTTTCACCAG TTTTGATTTA AACGTGGCCA

801 ATATGGACAA CTTCTTCGCC CCCGTTTTCA CCATGGGCAA ATATTATACG

851 CAAGGCGACA AGGTGCTGAT GCCGCTGGCG ATTCAGGTTC ATCATGCCGT

901 TTGTGATGGC TTCCATGTCG GCAGAATGCT TAATGAATTA CAACAGTACT

951 GCGATGAGTG GCAGGGCGGG GCGTAAACGC GTGGATCCGG CTTACTAAAA

1001 GCCAGATAAC AGTATGCGTA TTTGCGCGCT GATTTTTGCG GTATAAGAAT

1051 ATATACTGAT ATGTATACCC GAAGTATGTC AAAAAGAGGT ATGCTATGAA

1101 GCAGCGTATT ACAGTGACAG TTGACAGCGA CAGCTATCAG TTGCTCAAGG

1151 CATATATGAT GTCAATATCT CCGGTCTGGT AAGCACAACC ATGCAGAATG

1201 AAGCCCGTCG TCTGCGTGCC GAACGCTGGA AAGCGGAAAA TCAGGAAGGG

1251 ATGGCTGAGG TCGCCCGGTT TATTGAAATG AACGGCTCTT TTGCTGACGA

1301 GAACAGGGGC TGGTGAAATG CAGTTTAAGG TTTACACCTA TAAAAGAGAG

1351 AGCCGTTATC GTCTGTTTGT GGATGTACAG AGTGATATTA TTGACACGCC

1401 CGGGCGACGG ATGGTGATCC CCCTGGCCAG TGCACGTCTG CTGTCAGATA

1451 AAGTCTCCCG TGAACTTTAC CCGGTGGTGC ATATCGGGGA TGAAAGCTGG

1501 CGCATGATGA CCACCGATAT GGCCAGTGTG CCGGTCTCCG TTATCGGGGA

1551 AGAAGTGGCT GATCTCAGCC ACCGCGAAAA TGACATCAAA AACGCCATTA

1601 ACCTGATGTT CTGGGGAATA TAAATGTCAG GCTCCCTTAT ACACAGCCAG

1651 TCTGCAGGTC GACCATAGTG ACTGGATATG TTGTGTTTTA CAGTATTATG

1701 TAGTCTGTTT TTTATGCAAA ATCTAATTTA ATATATTGAT ATTTATATCA

1751 TTTTACGTTT CTCGTTCAGC TTTCTTGTAC AAAGTGGTGA TAAACGGCCG

1801 GCCAGATCTG GTACCGCTAG AAAAAGGTAG GTTCAACCAC TGATGCCTAG

1851 GCACACCGAA ACGACTAACC CTAATTCTTA TCCTTTACTT CAGGGCTAGC

1901 AACTTAAAAA AAAAAATCAA AATGGAGGCC AAGACCTACA TCGGCAAGCT

1951 GAAGAGCCGC AAGATCGTGA GCAACGAGGA TACCTACGAT ATCCAGACCA

2001 GCACCCACAA CTTCTTCGCC AACGATATCC TGGTGCACAA CTCGGCAACA

2051 AGTCAGTTGA GTACGCGCGC GTTGGAGGGA ATCTTCGAGG CCACTCACCG

2101 ATTGATTTAC GGTGCAAAAG ATGACTCCGG CCAACGTTAC TTGGCCTGGT

2151 CCGGTCACAG CGCACGTGTG GGTGCCGCCC GTGACATGGC GAGGGCCGGT

2201 GTTTCCATCC CAGAAATCAT GCAAGCGGGC GGATGGACCA ACGTGAATAT

2251 TGTAATGAAC TACATTCGTA ATCTGGATAG TGAAACAGGC GCAATGGTGC

2301 GCCTGCTGGA GGACGGAGAT TAAACCGGTG TTTAAACAAT CAACCTCTGG

2351 ATTACAAAAT TTGTGAAAGA TTGACTGGTA TTCTTAACTA TGTTGCTCCT

2401 TTTACGCTAT GTGGATACGC TGCTTTAATG CCTTTGTATC ATGCTATTGC

2451 TTCCCGTATG GCTTTCATTT TCTCCTCCTT GTATAAATCC TGGTTGCTGT

2501 CTCTTTATGA GGAGTTGTGG CCCGTTGTCA GGCAACGTGG CGTGGTGTGC

2551 ACTGTGTTTG CTGACGCAAC CCCCACTGGT TGGGGCATTG CCACCACCTG

2601 TCAGCTCCTT TCCGGGACTT TCGCTTTCCC CCTCCCTATT GCCACGGCGG

2651 AACTCATCGC CGCCTGCCTT GCCCGCTGCT GGACAGGGGC TCGGCTGTTG

2701 GGCACTGACA ATTCCGTGGT GTTGTCGGGG AAATCATCGT CCTTTCCTTG

2751 GCTGCTCGCC TGTGTTGCCA CCTGGATTCT GCGCGGGACG TCCTTCTGCT

2801 ACGTCCCTTC GGCCCTCAAT CCAGCGGACC TTCCTTCCCG CGGCCTGCTG

2851 CCGGCTCTGC GGCCTCTTCC GCGTCTTCGC CTTCGCCCTC AGACGAGTCG

2901 GATCTCCCTT TGGGCCGCCT CCCCGCCTGA AGCTTATCGA TACCGTCGAC

2951 TAAAGCCAAA TAGAAAATTA TTCAGTTCCT GGCTTAAGTT TTTAAAAGTG

3001 ATATTATTTA TTTGGTTGTA ACCAACCAAA AGAATGTAAA TAACTAATAC

3051 ATAATTATGT TAGTTTTAAG TTAGCAACAA ATTGATTTTA GCTATATTAG

3101 CTACTTGGTT AATAAATAGA ATATATTTAT TTAAAGATAA TTGCGTTTTT

3151 ATTGTCAGGG AGTGAGTTTG CTTAAAAACT CGTTTAGATC CACTAGTTCT

3201 AGAGCGGCCG CTGGCCACGG GTGCGCATGA TCGTGCTCCT GTCGTTGAGG

3251 ACCCGGCTAG GCTGGCGGGG TTGCCTTACT GGTTAGCAGA ATGAATCACC

3301 GATACGCGAG CGAACGTGAA GCGACTGCTG CTGCAAAACG TCTGCGACCT

3351 GAGCAACAAC ATGAATGGTC TTCGGTTTCC GTGTTTCGTA AAGTCTGGAA

3401 ACGCGGAAGT CAGCGCCCTG CACCATTATG TTCCGGAGGC GCGCCCTAGT

3451 TCCAGTGAAA TCCAAGCATT TTCTAAATTA AATGTATTCT TATTATTATA

3501 GTTGTTATTT TTGATATATA TAAACAACAC TATTATGCCC ACCATTTTTT

3551 TGAGATGCAT CTACACAAGG AACAAACACT GGATGTCACT TTCAGTTCAA

3601 ATTGTAACGC TAATCACTCC GAACAGGTCA CAAAAAATTA CCTTAAAAAG

3651 TCATAATATT AAATTAGAAT AAATATAGCT GTGAGGGAAA TATATACAAA

3701 TATATTGGAG CAAATAAATT GTACATACAA ATATTTATTA CTAATTTCTA

3751 TTGAGACGAA ATGAACCACT CGGAACCATT TGAGCGAACC GAATCGCGCG

3801 GAACTAACGA CAGTCGCTCC AAGGTCGTCG AACAAAAGGT GAATGTGTTG

3851 CGGAGAGCGG GTGGGAGACA GCGAAAGAGC AACTACGAAA CGTGGTGTGG

3901 TGGAGGTGAA TTATGAAGAG GGCGCGCGAT TTGAAAAGTA TGTATATAAA

3951 AAATATATCC CGGTGTTTTA TGTAGCGATA AACGAGTTTT TGATGTAAGG

4001 TATGCAGGTG TGTAAGTCTT TTGGTTAGAA GACAAATCCA AAGTCTACTT

4051 GTGGGGATGT TCGAAGGGGA AATACTTGTA TTCTATAGGT CATATCTTGT

4101 TTTTATTGGC ACAAATATAA TTACATTAGC TTTTTGAGGG GGCAATAAAC

4151 AGTAAACACG ATGGTAATAA TGGTAAAAAA AAAAAACAAG CAGTTATTTC

4201 GGATATATGT CGGCTACTCC TTGCGTCGGG CCCGAAGTCT TAGAGCCAGA

4251 TATGCGAGCA CCCGGAAGCT CACGATGAGA ATGGCCAGAC CCACGTAGTC

4301 CAGCGGCAGA TCGGCGGCGG AGAAGTTAAG CGTCTCCAGG ATGACCTTGC

4351 CCGAACTGGG GCACGTGGTG TTCGACGATG TGCAGCTAAT TTCGCCCGGC

4401 TCCACGTCCG CCCATTGGTT AATCAGCAGA CCCTCGTTGG CGTAACGGAA

4451 CCATGAGAGG TACGACAACC ATTTGAGGTA TACTGGCACC GAGCCCGAGT

4501 TCAAGAAGAA GCCGCCAAAG AGCAGGAATG GTATGATAAC CGGCGGACCC

4551 ACAGACAGCG CCATCGAGGT CGAGGAGCTG GCGCAGGATA TTAGATATCC

4601 GAAGGACGTT GACACATTGG CCACCAGAGT GACCAGCGCC AGGCAGTTGA

4651 AGAAGTGCAG CACTCCGGCC CGCAGTCCGA TCATCGGATA GGCAATCGCC

4701 GTGAAGACCA GTGGCACTGT GAGAAAAAGC GGCAATTCGG CAATCGTTTT

4751 GCCCAGAAAG TATGTGTCAC AGCGATAAAG TCGACTTCGG GCCTCCCTCA

4801 TAAAAACTGG CAGCTCTGAG GTGAACACCT AAATCGAATC GATTCATTAG

4851 AAAGTTAGTA AATTATTGAA ATGCAAATGT ATTCTAAACA TGACTTACAT

4901 TTATCGTGGC AAAGACGTTT TGAAAGGTCA TGTTGGTCAG GAAGAGGAAG

4951 ATGGCTCCGT TGATATTCAT CACACCCACT TGCGTGAGTT GTTGGCCCAA

5001 AAAGATGAGG CCAATCAAGA TGGCAACCAT CTGCAAATTA AAATGTTACT

5051 CGCATCTCAT TAATATTCGC GAGTTAAATG AAATTTATTT ATCTTCTGCA

5101 AAACTATAAA CTATACATCT CATTGAAAAA AACTAAGAAG GGTGTGGAAT

5151 CAGGCAATTC TATCTAAAAT CTAGCGAATT TGTTTCCAAG AATTGTAAGC

5201 GTTATATCAT TTGTTTCCAC TGGAACCACT CACCGTTGTC TGAATAAGTC

5251 GCACTTTTAC GAGGAGTGGT TCCTTGAGCA CCGACAGCCA GGATCGCCAC

5301 AGGACCGCCC GGAACTGCAT GAACCAGGTG GCCTTGTAGG TGTACCCATT

5351 CTCCGGCTGC TCCAGTGGCT TCTCCAGATT TTTGGTGGCC AACAACTGCT

5401 CCATATCCCG GGCTACTTTG CTAATGGCAA AATTGTCGCA TATCTTGGCG

5451 ATCCGATCAC GGGACTCGAT CTCCCGTCCG GGCACAACGG CCAACACCTG

5501 TACGTAAAAG TCCGCCGGAT TGTAGTTGGT AGGACACTGG GCACCCACGC

5551 TGGATAGGAG TTGAGATGTT ATGTAATACT AGATACCCTT AATAAACACA

5601 TCGAACTCAC TAGGAAAAGA AGTCGACGGC TTCGCTGGGA GTGCCCAAGA

5651 AAGCTACCCT GCCCTCGGCC ATCAGAAGGA TCTTGTCAAA GAGCTCAAAC

5701 AGCTCGGAAG ACGGCTGATG AATGGTCAGG ATGACGGTCT TGCCCTTCTG

5751 CGACAGCTTC TTCAGCACCT GGACGACGCT GTGGGCGGTA AAGGAGTCCA

5801 GTCCGGAGGT GGGCTCATCG CAGATCAGAA GCGGCGGATC GGTTAGAGCC

5851 TCGGAGGCGA ATGCCAGACG CTTCCTTTCT CCGCCGGACA GACCTTTCAC

5901 CCTGCCGGGC ACACCGATGA TCGTGTGCTG ACATTTGCTG AGCGAAAGCT

5951 CCTGGATCAC CTGATCCACG CGGGCCACTC GCTGCCGATA GGTCAGATGT

6001 CGTGGCATCC GCACCATGGC TTGGAAAATC AGGTGTTCCC TGGCCGTTAG

6051 GGAGCCGATA AAGAGGTCAT CCTGCTGGAC ATAGGCGCAC CTGGCCTGCA

6101 TCTCCTTGGC GTCCACAGGT TGGCCATTGA GCAGTCGCAT CCCGGATGGC

6151 GATACTTGGA TGCCCTGCGG CGATCGAAAG GCAAGGGCAT TCAGCAGGGT

6201 CGTCTTTCCG GCACCGGAAC TGCCCATCAC GGCCAAAAGT TCGCCCGGAT

6251 AGGCCACGCC GCAAACTGAG TTTCAAATTG GTAATTGGAC CCTTTATTAA

6301 GATTTCACAC AGATCAGCCG ACTGCGAATA GAAACTCACC GTTCTTGAGC

6351 AAATGTTTCC TGGGCGCCGG TATGTGTCGC TCGTTGCAGA ATAGTCCGCG

6401 TGTCCGGTTG ACCAGCTGCC GCCATCCGGA GCCCGGCTGA TTGACCGCCC

6451 CAAAGATGTC CATATTGTGC CAGGCATAGG TGAGGTTCTC GGCTAGTTGG

6501 CCGCTCCCTG AACCGGAGTC CTCCGGCGGA CTGGGTGGCC GGAGCGTGCC

6551 GTAGTTTTTG GCCTGCCCGA AGCCCTGGTT AATGCAGCTC TGCGAAGCCG

6601 CTCCGCTGTC ACCCTGCAAT GATAGGGGAT CTCAAATATC AACTACTAGC

6651 GTTATGCTCA TCTAACCCCG AACAAAAAGT ACCCCGAAGT ATCCTACGAA

6701 GTAGGTTTAT ACTTTTATTT ATTTTTTGTG CATCTAGGAT CAGCTTAAAA

6751 TATCTGGTTG TTATATTTTT TGTAAAAAAG AATATAGTCG AAAATGAATG

6801 CCTTTAGATG TCTTGATCAT GATATGATCT CAAAAATTGT CTTATATAGC

6851 GAGAACAGCT ACCAGAATAA TCTGTTTCGT GTCACTATTT GTTTGTGCAA

6901 TTGCGGTTTG GGATTTTTGT GGGTCGCAGT TCTCACGCCG CATACAATTT

6951 GATGTTGCAA TCGCAGTTCC TATAGATCAA GTGAACTTAA GATGTATGCA

7001 CATGTACTAC TCACATTGTT CAGATGCTCG GCAGATGGGT GTTTGCTGCC

7051 TCCGCGAATT AATAGCTCCT GATCCTCTTG GCCCATTGCC GGGATTTTTC

7101 ACACTTTCCC CTGCTTACCC ACCCAAAACC AATCACCACC CCAATCACTC

7151 AAAAAACAAA CAAAAATAAG AAGCGAGAGG AGTTTTGGCA CAGCACTTTG

7201 TGTTTAATTG ATGGCGTAAA CCGCTTGGAG CTTCGTCACG AAACCGCTGA

7251 CAAAATGCAA CTGAAGGCGG ACATTGACGC TACGTAACGC TACAAACGGT

7301 GGCGAAAGAG ATAGCGGACG CAGCGGCGAA AGAGACGGCG ATATTTCTGT

7351 GGACAGAGAA GGAGGCAAAC AGCGCTGACT TTGAGTGGAA TGTCATTTTG

7401 AGTGAGAGGT AATCGAAAGA ACCTGGTACA TCAAATACCC TTGGATCGAA

7451 GTAAATTTAA AACTGATCAG ATAAGTTCAA TGATATCCAG TGCAGTAAAA

7501 AAAAAAAATG TTTTTTTTAT CTACTTTCCG CAAAAATGGG TTTTATTAAC

7551 TTACATACAT ACTAGGCGCG CCCATATGTT CGGCTTGTCG ACATGCCCGC

7601 CGTGACCGTC GAGAACCCGC TGACGCTGCC CCGCGTATCC GCACCCGCCG

7651 ACGCCGTCGC ACGTCCCGTG CTCACCGTGA CCACCGCGCC CAGCGGTTTC

7701 GAGGGCGAGG GCTTCCCGGT GCGCCGCGCG TTCGCCGGGA TCAACTACCG

7751 CCACCTCGAC CCGTTCATCA TGATGGACCA GATGGGTGAG GTGGAGTACG

7801 CGCCCGGGGA GCCCAAGGGC ACGCCCTGGC ACCCGCACCG CGGCTTCGAG

7851 ACCGTGACCT ACATCGTCGA CGGTAACATG TGAGCAAAAG GCCAGCAAAA

7901 GGCCAGGAAC CGTAAAAAGG CCGCGTTGCT GGCGTTTTTC CATAGGCTCC

7951 GCCCCCCTGA CGAGCATCAC AAAAATCGAC GCTCAAGTCA GAGGTGGCGA

8001 AACCCGACAG GACTATAAAG ATACCAGGCG TTTCCCCCTG GAAGCTCCCT

8051 CGTGCGCTCT CCTGTTCCGA CCCTGCCGCT TACCGGATAC CTGTCCGCCT

8101 TTCTCCCTTC GGGAAGCGTG GCGCTTTCTC ATAGCTCACG CTGTAGGTAT

8151 CTCAGTTCGG TGTAGGTCGT TCGCTCCAAG CTGGGCTGTG TGCACGAACC

8201 CCCCGTTCAG CCCGACCGCT GCGCCTTATC CGGTAACTAT CGTCTTGAGT

8251 CCAACCCGGT AAGACACGAC TTATCGCCAC TGGCAGCAGC CACTGGTAAC

8301 AGGATTAGCA GAGCGAGGTA TGTAGGCGGT GCTACAGAGT TCTTGAAGTG

8351 GTGGCCTAAC TACGGCTACA CTAGAAGAAC AGTATTTGGT ATCTGCGCTC

8401 TGCTGAAGCC AGTTACCTTC GGAAAAAGAG TTGGTAGCTC TTGATCCGGC

8451 AAACAAACCA CCGCTGGTAG CGGTGGTTTT TTTGTTTGCA AGCAGCAGAT

8501 TACGCGCAGA AAAAAAGGAT CTCAAGAAGA TCCTTTGATC TTTTCTACGG

8551 GGTCTGACGC TCAGTGGAAC GAAAACTCAC GTTAAGGGAT TTTGGTCATG

8601 AGATTATCAA AAAGGATCTT CACCTAGATC CTTTTAAATT AAAAATGAAG

8651 TTTTAAATCA ATCTAAAGTA TATATGAGTA AACTTGGTCT GACAGTTACC

8701 AATGCTTAAT CAGTGAGGCA CCTATCTCAG CGATCTGTCT ATTTCGTTCA

8751 TCCATAGTTG CCTGACTCCC CGTCGTGTAG ATAACTACGA TACGGGAGGG

8801 CTTACCATCT GGCCCCAGTG CTGCAATGAT ACCGCGAGAC CCACGCTCAC

8851 CGGCTCCAGA TTTATCAGCA ATAAACCAGC CAGCCGGAAG GGCCGAGCGC

8901 AGAAGTGGTC CTGCAACTTT ATCCGCCTCC ATCCAGTCTA TTAATTGTTG

8951 CCGGGAAGCT AGAGTAAGTA GTTCGCCAGT TAATAGTTTG CGCAACGTTG

9001 TTGCCATTGC TACAGGCATC GTGGTGTCAC GCTCGTCGTT TGGTATGGCT

9051 TCATTCAGCT CCGGTTCCCA ACGATCAAGG CGAGTTACAT GATCCCCCAT

9101 GTTGTGCAAA AAAGCGGTTA GCTCCTTCGG TCCTCCGATC GTTGTCAGAA

9151 GTAAGTTGGC CGCAGTGTTA TCACTCATGG TTATGGCAGC ACTGCATAAT

9201 TCTCTTACTG TCATGCCATC CGTAAGATGC TTTTCTGTGA CTGGTGAGTA

9251 CTCAACCAAG TCATTCTGAG AATAGTGTAT GCGGCGACCG AGTTGCTCTT

9301 GCCCGGCGTC AATACGGGAT AATACCGCGC CACATAGCAG AACTTTAAAA

9351 GTGCTCATCA TTGGAAAACG TTCTTCGGGG CGAAAACTCT CAAGGATCTT

9401 ACCGCTGTTG AGATCCAGTT CGATGTAACC CACTCGTGCA CCCAACTGAT

9451 CTTCAGCATC TTTTACTTTC ACCAGCGTTT CTGGGTGAGC AAAAACAGGA

9501 AGGCAAAATG CCGCAAAAAA GGGAATAAGG GCGACACGGA AATGTTGAAT

9551 ACTCATACTC TTCCTTTTTC AATATTATTG AAGCATTTAT CAGGGTTATT

9601 GTCTCATGAG CGGATACATA TTTGAATGTA TTTAGAAAAA TAAACAAATA

9651 GGGGTTCCGC GCACATTTCC CCGAAAAGTG CCACCTGAGT

**HJP-178-IVS-Syn21-Cre_AB_-NrdJ-1_N_-WPREw**

1 TAAGAAACCA TTATTATCAT GACATTAACC TATAAAAATA GGCGTATCAC

51 GAGGCCCTTT CGTCTTCAAG AATTCGTTTA TCACAAGTTT GTACAAAAAA

101 GCTGAACGAG AAACGTAAAA TGATATAAAT ATCAATATAT TAAATTAGAT

151 TTTGCATAAA AAACAGACTA CATAATACTG TAAAACACAA CATATCCAGT

201 CACTATGGCG GCCGCATTAG GCACCCCAGG CTTTACACTT TATGCTTCCG

251 GCTCGTATAA TGTGTGGATT TTGAGTTAGG ATCCGTCGAG ATTTTCAGGA

301 GCTAAGGAAG CTAAAATGGA GAAAAAAATC ACTGGATATA CCACCGTTGA

351 TATATCCCAA TGGCATCGTA AAGAACATTT TGAGGCATTT CAGTCAGTTG

401 CTCAATGTAC CTATAACCAG ACCGTTCAGC TGGATATTAC GGCCTTTTTA

451 AAGACCGTAA AGAAAAATAA GCACAAGTTT TATCCGGCCT TTATTCACAT

501 TCTTGCCCGC CTGATGAATG CTCATCCGGA ATTCCGTATG GCAATGAAAG

551 ACGGTGAGCT GGTGATATGG GATAGTGTTC ACCCTTGTTA CACCGTTTTC

601 CATGAGCAAA CTGAAACGTT TTCATCGCTC TGGAGTGAAT ACCACGACGA

651 TTTCCGGCAG TTTCTACACA TATATTCGCA AGATGTGGCG TGTTACGGTG

701 AAAACCTGGC CTATTTCCCT AAAGGGTTTA TTGAGAATAT GTTTTTCGTC

751 TCAGCCAATC CCTGGGTGAG TTTCACCAGT TTTGATTTAA ACGTGGCCAA

801 TATGGACAAC TTCTTCGCCC CCGTTTTCAC CATGGGCAAA TATTATACGC

851 AAGGCGACAA GGTGCTGATG CCGCTGGCGA TTCAGGTTCA TCATGCCGTT

901 TGTGATGGCT TCCATGTCGG CAGAATGCTT AATGAATTAC AACAGTACTG

951 CGATGAGTGG CAGGGCGGGG CGTAAACGCG TGGATCCGGC TTACTAAAAG

1001 CCAGATAACA GTATGCGTAT TTGCGCGCTG ATTTTTGCGG TATAAGAATA

1051 TATACTGATA TGTATACCCG AAGTATGTCA AAAAGAGGTA TGCTATGAAG

1101 CAGCGTATTA CAGTGACAGT TGACAGCGAC AGCTATCAGT TGCTCAAGGC

1151 ATATATGATG TCAATATCTC CGGTCTGGTA AGCACAACCA TGCAGAATGA

1201 AGCCCGTCGT CTGCGTGCCG AACGCTGGAA AGCGGAAAAT CAGGAAGGGA

1251 TGGCTGAGGT CGCCCGGTTT ATTGAAATGA ACGGCTCTTT TGCTGACGAG

1301 AACAGGGGCT GGTGAAATGC AGTTTAAGGT TTACACCTAT AAAAGAGAGA

1351 GCCGTTATCG TCTGTTTGTG GATGTACAGA GTGATATTAT TGACACGCCC

1401 GGGCGACGGA TGGTGATCCC CCTGGCCAGT GCACGTCTGC TGTCAGATAA

1451 AGTCTCCCGT GAACTTTACC CGGTGGTGCA TATCGGGGAT GAAAGCTGGC

1501 GCATGATGAC CACCGATATG GCCAGTGTGC CGGTCTCCGT TATCGGGGAA

1551 GAAGTGGCTG ATCTCAGCCA CCGCGAAAAT GACATCAAAA ACGCCATTAA

1601 CCTGATGTTC TGGGGAATAT AAATGTCAGG CTCCCTTATA CACAGCCAGT

1651 CTGCAGGTCG ACCATAGTGA CTGGATATGT TGTGTTTTAC AGTATTATGT

1701 AGTCTGTTTT TTATGCAAAA TCTAATTTAA TATATTGATA TTTATATCAT

1751 TTTACGTTTC TCGTTCAGCT TTCTTGTACA AAGTGGTGAT AAACGGCCGG

1801 CCAGATCTGG TACCGCTAGA AAAAGGTAGG TTCAACCACT GATGCCTAGG

1851 CACACCGAAA CGACTAACCC TAATTCTTAT CCTTTACTTC AGGGCTAGCA

1901 ACTTAAAAAA AAAAATCAAA ATGAGTAACT TGCTCACGGT ACACCAAAAT

1951 TTGCCGGCCC TGCCTGTGGA CGCCACGTCC GACGAGGTCC GAAAGAATCT

2001 GATGGACATG TTCCGTGACA GGCAGGCCTT TAGCGAGCAC ACCTGGAAGA

2051 TGCTGCTCTC GGTGTGCAGG TCCTGGGCCG CTTGGTGCAA GCTGAACAAC

2101 CGAAAGTGGT TTCCCGCGGA GCCCGAGGAC GTCCGCGATT ATCTGCTGTA

2151 CCTGCAGGCT CGCGGACTGG CCGTCAAAAC AATACAGCAA CATTTGGGTC

2201 AGCTGAACAT GCTCCACAGG CGCAGCGGAC TGCCCCGGCC ATCCGATAGC

2251 AATGCAGTGA GCCTGGTAAT GCGTCGAATA CGCAAGGAAA ACGTGGACGC

2301 CGGCGAGAGG GCCAAACAGG CGTTGGCTTT CGAACGCACC GATTTCGATC

2351 AGGTGCGGTC GTTGATGGAG AACAGCGATC GATGCCAGGA CATCCGGAAC

2401 TTGGCGTTTT TGGGCATTGC CTATAACACC CTCCTTCGCA TCGCCGAGAT

2451 AGCGCGTATC CGAGTTAAAG ACATTTCCCG TACAGACGGA GGCCGCATGC

2501 TTATTCACAT CGGACGCACG AAAACCCTGG TCAGCACTGC TGGCGTGGAG

2551 AAAGCACTTT CCCTGGGTGT GACGAAATTG GTCGAGCGAT GGATCAGTGT

2601 GAGTGGCGTC GCCGATGACC CCAACAATTA TCTGTTCTGC AGGGTGCGCA

2651 AAAATGGAGT GGCAGCCCCC TGCCTGGTGG GCAGCAGCGA GATCATCACC

2701 CGCAACTACG GCAAGACCAC CATCAAGGAG GTGGTGGAGA TCTTCGATAA

2751 CGATAAGAAC ATCCAGGTGC TGGCCTTCAA CACCCACACC GATAACATCG

2801 AGTGGGCCCC CATCAAGGCC GCCCAGCTGA CCCGCCCCAA CGCCGAGCTG

2851 GTGGAGCTGG AGATCGATAC CCTGCACGGC GTGAAGACCA TCCGCTGCAC

2901 CCCCGATCAC CCCGTGTACA CCAAGAACCG CGGCTACGTG CGCGCCGATG

2951 AGCTGACCGA TGATGATGAG CTGGTGGTGG CCATCTAAAC CGGTGTTTAA

3001 ACAATCAACC TCTGGATTAC AAAATTTGTG AAAGATTGAC TGGTATTCTT

3051 AACTATGTTG CTCCTTTTAC GCTATGTGGA TACGCTGCTT TAATGCCTTT

3101 GTATCATGCT ATTGCTTCCC GTATGGCTTT CATTTTCTCC TCCTTGTATA

3151 AATCCTGGTT GCTGTCTCTT TATGAGGAGT TGTGGCCCGT TGTCAGGCAA

3201 CGTGGCGTGG TGTGCACTGT GTTTGCTGAC GCAACCCCCA CTGGTTGGGG

3251 CATTGCCACC ACCTGTCAGC TCCTTTCCGG GACTTTCGCT TTCCCCCTCC

3301 CTATTGCCAC GGCGGAACTC ATCGCCGCCT GCCTTGCCCG CTGCTGGACA

3351 GGGGCTCGGC TGTTGGGCAC TGACAATTCC GTGGTGTTGT CGGGGAAATC

3401 ATCGTCCTTT CCTTGGCTGC TCGCCTGTGT TGCCACCTGG ATTCTGCGCG

3451 GGACGTCCTT CTGCTACGTC CCTTCGGCCC TCAATCCAGC GGACCTTCCT

3501 TCCCGCGGCC TGCTGCCGGC TCTGCGGCCT CTTCCGCGTC TTCGCCTTCG

3551 CCCTCAGACG AGTCGGATCT CCCTTTGGGC CGCCTCCCCG CCTGAAGCTT

3601 ATCGATACCG TCGACTAAAG CCAAATAGAA AATTATTCAG TTCCTGGCTT

3651 AAGTTTTTAA AAGTGATATT ATTTATTTGG TTGTAACCAA CCAAAAGAAT

3701 GTAAATAACT AATACATAAT TATGTTAGTT TTAAGTTAGC AACAAATTGA

3751 TTTTAGCTAT ATTAGCTACT TGGTTAATAA ATAGAATATA TTTATTTAAA

3801 GATAATTGCG TTTTTATTGT CAGGGAGTGA GTTTGCTTAA AAACTCGTTT

3851 AGATCCACTA GTTCTAGAGC GGCCGCTGGC CACGGGTGCG CATGATCGTG

3901 CTCCTGTCGT TGAGGACCCG GCTAGGCTGG CGGGGTTGCC TTACTGGTTA

3951 GCAGAATGAA TCACCGATAC GCGAGCGAAC GTGAAGCGAC TGCTGCTGCA

4001 AAACGTCTGC GACCTGAGCA ACAACATGAA TGGTCTTCGG TTTCCGTGTT

4051 TCGTAAAGTC TGGAAACGCG GAAGTCAGCG CCCTGCACCA TTATGTTCCG

4101 GAGGCGCGCC CTAGTTCCAG TGAAATCCAA GCATTTTCTA AATTAAATGT

4151 ATTCTTATTA TTATAGTTGT TATTTTTGAT ATATATAAAC AACACTATTA

4201 TGCCCACCAT TTTTTTGAGA TGCATCTACA CAAGGAACAA ACACTGGATG

4251 TCACTTTCAG TTCAAATTGT AACGCTAATC ACTCCGAACA GGTCACAAAA

4301 AATTACCTTA AAAAGTCATA ATATTAAATT AGAATAAATA TAGCTGTGAG

4351 GGAAATATAT ACAAATATAT TGGAGCAAAT AAATTGTACA TACAAATATT

4401 TATTACTAAT TTCTATTGAG ACGAAATGAA CCACTCGGAA CCATTTGAGC

4451 GAACCGAATC GCGCGGAACT AACGACAGTC GCTCCAAGGT CGTCGAACAA

4501 AAGGTGAATG TGTTGCGGAG AGCGGGTGGG AGACAGCGAA AGAGCAACTA

4551 CGAAACGTGG TGTGGTGGAG GTGAATTATG AAGAGGGCGC GCGATTTGAA

4601 AAGTATGTAT ATAAAAAATA TATCCCGGTG TTTTATGTAG CGATAAACGA

4651 GTTTTTGATG TAAGGTATGC AGGTGTGTAA GTCTTTTGGT TAGAAGACAA

4701 ATCCAAAGTC TACTTGTGGG GATGTTCGAA GGGGAAATAC TTGTATTCTA

4751 TAGGTCATAT CTTGTTTTTA TTGGCACAAA TATAATTACA TTAGCTTTTT

4801 GAGGGGGCAA TAAACAGTAA ACACGATGGT AATAATGGTA AAAAAAAAAA

4851 ACAAGCAGTT ATTTCGGATA TATGTCGGCT ACTCCTTGCG TCGGGCCCGA

4901 AGTCTTAGAG CCAGATATGC GAGCACCCGG AAGCTCACGA TGAGAATGGC

4951 CAGACCCACG TAGTCCAGCG GCAGATCGGC GGCGGAGAAG TTAAGCGTCT

5001 CCAGGATGAC CTTGCCCGAA CTGGGGCACG TGGTGTTCGA CGATGTGCAG

5051 CTAATTTCGC CCGGCTCCAC GTCCGCCCAT TGGTTAATCA GCAGACCCTC

5101 GTTGGCGTAA CGGAACCATG AGAGGTACGA CAACCATTTG AGGTATACTG

5151 GCACCGAGCC CGAGTTCAAG AAGAAGCCGC CAAAGAGCAG GAATGGTATG

5201 ATAACCGGCG GACCCACAGA CAGCGCCATC GAGGTCGAGG AGCTGGCGCA

5251 GGATATTAGA TATCCGAAGG ACGTTGACAC ATTGGCCACC AGAGTGACCA

5301 GCGCCAGGCA GTTGAAGAAG TGCAGCACTC CGGCCCGCAG TCCGATCATC

5351 GGATAGGCAA TCGCCGTGAA GACCAGTGGC ACTGTGAGAA AAAGCGGCAA

5401 TTCGGCAATC GTTTTGCCCA GAAAGTATGT GTCACAGCGA TAAAGTCGAC

5451 TTCGGGCCTC CCTCATAAAA ACTGGCAGCT CTGAGGTGAA CACCTAAATC

5501 GAATCGATTC ATTAGAAAGT TAGTAAATTA TTGAAATGCA AATGTATTCT

5551 AAACATGACT TACATTTATC GTGGCAAAGA CGTTTTGAAA GGTCATGTTG

5601 GTCAGGAAGA GGAAGATGGC TCCGTTGATA TTCATCACAC CCACTTGCGT

5651 GAGTTGTTGG CCCAAAAAGA TGAGGCCAAT CAAGATGGCA ACCATCTGCA

5701 AATTAAAATG TTACTCGCAT CTCATTAATA TTCGCGAGTT AAATGAAATT

5751 TATTTATCTT CTGCAAAACT ATAAACTATA CATCTCATTG AAAAAAACTA

5801 AGAAGGGTGT GGAATCAGGC AATTCTATCT AAAATCTAGC GAATTTGTTT

5851 CCAAGAATTG TAAGCGTTAT ATCATTTGTT TCCACTGGAA CCACTCACCG

5901 TTGTCTGAAT AAGTCGCACT TTTACGAGGA GTGGTTCCTT GAGCACCGAC

5951 AGCCAGGATC GCCACAGGAC CGCCCGGAAC TGCATGAACC AGGTGGCCTT

6001 GTAGGTGTAC CCATTCTCCG GCTGCTCCAG TGGCTTCTCC AGATTTTTGG

6051 TGGCCAACAA CTGCTCCATA TCCCGGGCTA CTTTGCTAAT GGCAAAATTG

6101 TCGCATATCT TGGCGATCCG ATCACGGGAC TCGATCTCCC GTCCGGGCAC

6151 AACGGCCAAC ACCTGTACGT AAAAGTCCGC CGGATTGTAG TTGGTAGGAC

6201 ACTGGGCACC CACGCTGGAT AGGAGTTGAG ATGTTATGTA ATACTAGATA

6251 CCCTTAATAA ACACATCGAA CTCACTAGGA AAAGAAGTCG ACGGCTTCGC

6301 TGGGAGTGCC CAAGAAAGCT ACCCTGCCCT CGGCCATCAG AAGGATCTTG

6351 TCAAAGAGCT CAAACAGCTC GGAAGACGGC TGATGAATGG TCAGGATGAC

6401 GGTCTTGCCC TTCTGCGACA GCTTCTTCAG CACCTGGACG ACGCTGTGGG

6451 CGGTAAAGGA GTCCAGTCCG GAGGTGGGCT CATCGCAGAT CAGAAGCGGC

6501 GGATCGGTTA GAGCCTCGGA GGCGAATGCC AGACGCTTCC TTTCTCCGCC

6551 GGACAGACCT TTCACCCTGC CGGGCACACC GATGATCGTG TGCTGACATT

6601 TGCTGAGCGA AAGCTCCTGG ATCACCTGAT CCACGCGGGC CACTCGCTGC

6651 CGATAGGTCA GATGTCGTGG CATCCGCACC ATGGCTTGGA AAATCAGGTG

6701 TTCCCTGGCC GTTAGGGAGC CGATAAAGAG GTCATCCTGC TGGACATAGG

6751 CGCACCTGGC CTGCATCTCC TTGGCGTCCA CAGGTTGGCC ATTGAGCAGT

6801 CGCATCCCGG ATGGCGATAC TTGGATGCCC TGCGGCGATC GAAAGGCAAG

6851 GGCATTCAGC AGGGTCGTCT TTCCGGCACC GGAACTGCCC ATCACGGCCA

6901 AAAGTTCGCC CGGATAGGCC ACGCCGCAAA CTGAGTTTCA AATTGGTAAT

6951 TGGACCCTTT ATTAAGATTT CACACAGATC AGCCGACTGC GAATAGAAAC

7001 TCACCGTTCT TGAGCAAATG TTTCCTGGGC GCCGGTATGT GTCGCTCGTT

7051 GCAGAATAGT CCGCGTGTCC GGTTGACCAG CTGCCGCCAT CCGGAGCCCG

7101 GCTGATTGAC CGCCCCAAAG ATGTCCATAT TGTGCCAGGC ATAGGTGAGG

7151 TTCTCGGCTA GTTGGCCGCT CCCTGAACCG GAGTCCTCCG GCGGACTGGG

7201 TGGCCGGAGC GTGCCGTAGT TTTTGGCCTG CCCGAAGCCC TGGTTAATGC

7251 AGCTCTGCGA AGCCGCTCCG CTGTCACCCT GCAATGATAG GGGATCTCAA

7301 ATATCAACTA CTAGCGTTAT GCTCATCTAA CCCCGAACAA AAAGTACCCC

7351 GAAGTATCCT ACGAAGTAGG TTTATACTTT TATTTATTTT TTGTGCATCT

7401 AGGATCAGCT TAAAATATCT GGTTGTTATA TTTTTTGTAA AAAAGAATAT

7451 AGTCGAAAAT GAATGCCTTT AGATGTCTTG ATCATGATAT GATCTCAAAA

7501 ATTGTCTTAT ATAGCGAGAA CAGCTACCAG AATAATCTGT TTCGTGTCAC

7551 TATTTGTTTG TGCAATTGCG GTTTGGGATT TTTGTGGGTC GCAGTTCTCA

7601 CGCCGCATAC AATTTGATGT TGCAATCGCA GTTCCTATAG ATCAAGTGAA

7651 CTTAAGATGT ATGCACATGT ACTACTCACA TTGTTCAGAT GCTCGGCAGA

7701 TGGGTGTTTG CTGCCTCCGC GAATTAATAG CTCCTGATCC TCTTGGCCCA

7751 TTGCCGGGAT TTTTCACACT TTCCCCTGCT TACCCACCCA AAACCAATCA

7801 CCACCCCAAT CACTCAAAAA ACAAACAAAA ATAAGAAGCG AGAGGAGTTT

7851 TGGCACAGCA CTTTGTGTTT AATTGATGGC GTAAACCGCT TGGAGCTTCG

7901 TCACGAAACC GCTGACAAAA TGCAACTGAA GGCGGACATT GACGCTACGT

7951 AACGCTACAA ACGGTGGCGA AAGAGATAGC GGACGCAGCG GCGAAAGAGA

8001 CGGCGATATT TCTGTGGACA GAGAAGGAGG CAAACAGCGC TGACTTTGAG

8051 TGGAATGTCA TTTTGAGTGA GAGGTAATCG AAAGAACCTG GTACATCAAA

8101 TACCCTTGGA TCGAAGTAAA TTTAAAACTG ATCAGATAAG TTCAATGATA

8151 TCCAGTGCAG TAAAAAAAAA AAATGTTTTT TTTATCTACT TTCCGCAAAA

8201 ATGGGTTTTA TTAACTTACA TACATACTAG GCGCGCCCAT ATGTTCGGCT

8251 TGTCGACATG CCCGCCGTGA CCGTCGAGAA CCCGCTGACG CTGCCCCGCG

8301 TATCCGCACC CGCCGACGCC GTCGCACGTC CCGTGCTCAC CGTGACCACC

8351 GCGCCCAGCG GTTTCGAGGG CGAGGGCTTC CCGGTGCGCC GCGCGTTCGC

8401 CGGGATCAAC TACCGCCACC TCGACCCGTT CATCATGATG GACCAGATGG

8451 GTGAGGTGGA GTACGCGCCC GGGGAGCCCA AGGGCACGCC CTGGCACCCG

8501 CACCGCGGCT TCGAGACCGT GACCTACATC GTCGACGGTA ACATGTGAGC

8551 AAAAGGCCAG CAAAAGGCCA GGAACCGTAA AAAGGCCGCG TTGCTGGCGT

8601 TTTTCCATAG GCTCCGCCCC CCTGACGAGC ATCACAAAAA TCGACGCTCA

8651 AGTCAGAGGT GGCGAAACCC GACAGGACTA TAAAGATACC AGGCGTTTCC

8701 CCCTGGAAGC TCCCTCGTGC GCTCTCCTGT TCCGACCCTG CCGCTTACCG

8751 GATACCTGTC CGCCTTTCTC CCTTCGGGAA GCGTGGCGCT TTCTCATAGC

8801 TCACGCTGTA GGTATCTCAG TTCGGTGTAG GTCGTTCGCT CCAAGCTGGG

8851 CTGTGTGCAC GAACCCCCCG TTCAGCCCGA CCGCTGCGCC TTATCCGGTA

8901 ACTATCGTCT TGAGTCCAAC CCGGTAAGAC ACGACTTATC GCCACTGGCA

8951 GCAGCCACTG GTAACAGGAT TAGCAGAGCG AGGTATGTAG GCGGTGCTAC

9001 AGAGTTCTTG AAGTGGTGGC CTAACTACGG CTACACTAGA AGAACAGTAT

9051 TTGGTATCTG CGCTCTGCTG AAGCCAGTTA CCTTCGGAAA AAGAGTTGGT

9101 AGCTCTTGAT CCGGCAAACA AACCACCGCT GGTAGCGGTG GTTTTTTTGT

9151 TTGCAAGCAG CAGATTACGC GCAGAAAAAA AGGATCTCAA GAAGATCCTT

9201 TGATCTTTTC TACGGGGTCT GACGCTCAGT GGAACGAAAA CTCACGTTAA

9251 GGGATTTTGG TCATGAGATT ATCAAAAAGG ATCTTCACCT AGATCCTTTT

9301 AAATTAAAAA TGAAGTTTTA AATCAATCTA AAGTATATAT GAGTAAACTT

9351 GGTCTGACAG TTACCAATGC TTAATCAGTG AGGCACCTAT CTCAGCGATC

9401 TGTCTATTTC GTTCATCCAT AGTTGCCTGA CTCCCCGTCG TGTAGATAAC

9451 TACGATACGG GAGGGCTTAC CATCTGGCCC CAGTGCTGCA ATGATACCGC

9501 GAGACCCACG CTCACCGGCT CCAGATTTAT CAGCAATAAA CCAGCCAGCC

9551 GGAAGGGCCG AGCGCAGAAG TGGTCCTGCA ACTTTATCCG CCTCCATCCA

9601 GTCTATTAAT TGTTGCCGGG AAGCTAGAGT AAGTAGTTCG CCAGTTAATA

9651 GTTTGCGCAA CGTTGTTGCC ATTGCTACAG GCATCGTGGT GTCACGCTCG

9701 TCGTTTGGTA TGGCTTCATT CAGCTCCGGT TCCCAACGAT CAAGGCGAGT

9751 TACATGATCC CCCATGTTGT GCAAAAAAGC GGTTAGCTCC TTCGGTCCTC

9801 CGATCGTTGT CAGAAGTAAG TTGGCCGCAG TGTTATCACT CATGGTTATG

9851 GCAGCACTGC ATAATTCTCT TACTGTCATG CCATCCGTAA GATGCTTTTC

9901 TGTGACTGGT GAGTACTCAA CCAAGTCATT CTGAGAATAG TGTATGCGGC

9951 GACCGAGTTG CTCTTGCCCG GCGTCAATAC GGGATAATAC CGCGCCACAT

10001 AGCAGAACTT TAAAAGTGCT CATCATTGGA AAACGTTCTT CGGGGCGAAA

10051 ACTCTCAAGG ATCTTACCGC TGTTGAGATC CAGTTCGATG TAACCCACTC

10101 GTGCACCCAA CTGATCTTCA GCATCTTTTA CTTTCACCAG CGTTTCTGGG

10151 TGAGCAAAAA CAGGAAGGCA AAATGCCGCA AAAAAGGGAA TAAGGGCGAC

10201 ACGGAAATGT TGAATACTCA TACTCTTCCT TTTTCAATAT TATTGAAGCA

10251 TTTATCAGGG TTATTGTCTC ATGAGCGGAT ACATATTTGA ATGTATTTAG

10301 AAAAATAAAC AAATAGGGGT TCCGCGCACA TTTCCCCGAA AAGTGCCACC

10351 TGAGTC

**HJP-177-IVS-Syn21-gp41-1^C^-Cre_BC_-WPREw**

1 TAAGAAACCA TTATTATCAT GACATTAACC TATAAAAATA GGCGTATCAC

51 GAGGCCCTTT CGTCTTCAAG AATTCGTTTA TCACAAGTTT GTACAAAAAA

101 GCTGAACGAG AAACGTAAAA TGATATAAAT ATCAATATAT TAAATTAGAT

151 TTTGCATAAA AAACAGACTA CATAATACTG TAAAACACAA CATATCCAGT

201 CACTATGGCG GCCGCATTAG GCACCCCAGG CTTTACACTT TATGCTTCCG

251 GCTCGTATAA TGTGTGGATT TTGAGTTAGG ATCCGTCGAG ATTTTCAGGA

301 GCTAAGGAAG CTAAAATGGA GAAAAAAATC ACTGGATATA CCACCGTTGA

351 TATATCCCAA TGGCATCGTA AAGAACATTT TGAGGCATTT CAGTCAGTTG

401 CTCAATGTAC CTATAACCAG ACCGTTCAGC TGGATATTAC GGCCTTTTTA

451 AAGACCGTAA AGAAAAATAA GCACAAGTTT TATCCGGCCT TTATTCACAT

501 TCTTGCCCGC CTGATGAATG CTCATCCGGA ATTCCGTATG GCAATGAAAG

551 ACGGTGAGCT GGTGATATGG GATAGTGTTC ACCCTTGTTA CACCGTTTTC

601 CATGAGCAAA CTGAAACGTT TTCATCGCTC TGGAGTGAAT ACCACGACGA

651 TTTCCGGCAG TTTCTACACA TATATTCGCA AGATGTGGCG TGTTACGGTG

701 AAAACCTGGC CTATTTCCCT AAAGGGTTTA TTGAGAATAT GTTTTTCGTC

751 TCAGCCAATC CCTGGGTGAG TTTCACCAGT TTTGATTTAA ACGTGGCCAA

801 TATGGACAAC TTCTTCGCCC CCGTTTTCAC CATGGGCAAA TATTATACGC

851 AAGGCGACAA GGTGCTGATG CCGCTGGCGA TTCAGGTTCA TCATGCCGTT

901 TGTGATGGCT TCCATGTCGG CAGAATGCTT AATGAATTAC AACAGTACTG

951 CGATGAGTGG CAGGGCGGGG CGTAAACGCG TGGATCCGGC TTACTAAAAG

1001 CCAGATAACA GTATGCGTAT TTGCGCGCTG ATTTTTGCGG TATAAGAATA

1051 TATACTGATA TGTATACCCG AAGTATGTCA AAAAGAGGTA TGCTATGAAG

1101 CAGCGTATTA CAGTGACAGT TGACAGCGAC AGCTATCAGT TGCTCAAGGC

1151 ATATATGATG TCAATATCTC CGGTCTGGTA AGCACAACCA TGCAGAATGA

1201 AGCCCGTCGT CTGCGTGCCG AACGCTGGAA AGCGGAAAAT CAGGAAGGGA

1251 TGGCTGAGGT CGCCCGGTTT ATTGAAATGA ACGGCTCTTT TGCTGACGAG

1301 AACAGGGGCT GGTGAAATGC AGTTTAAGGT TTACACCTAT AAAAGAGAGA

1351 GCCGTTATCG TCTGTTTGTG GATGTACAGA GTGATATTAT TGACACGCCC

1401 GGGCGACGGA TGGTGATCCC CCTGGCCAGT GCACGTCTGC TGTCAGATAA

1451 AGTCTCCCGT GAACTTTACC CGGTGGTGCA TATCGGGGAT GAAAGCTGGC

1501 GCATGATGAC CACCGATATG GCCAGTGTGC CGGTCTCCGT TATCGGGGAA

1551 GAAGTGGCTG ATCTCAGCCA CCGCGAAAAT GACATCAAAA ACGCCATTAA

1601 CCTGATGTTC TGGGGAATAT AAATGTCAGG CTCCCTTATA CACAGCCAGT

1651 CTGCAGGTCG ACCATAGTGA CTGGATATGT TGTGTTTTAC AGTATTATGT

1701 AGTCTGTTTT TTATGCAAAA TCTAATTTAA TATATTGATA TTTATATCAT

1751 TTTACGTTTC TCGTTCAGCT TTCTTGTACA AAGTGGTGAT AAACGGCCGG

1801 CCAGATCTGG TACCGCTAGA AAAAGGTAGG TTCAACCACT GATGCCTAGG

1851 CACACCGAAA CGACTAACCC TAATTCTTAT CCTTTACTTC AGGGCTAGCA

1901 ACTTAAAAAA AAAAATCAAA ATGATGCTGA AGAAGATCCT GAAGATCGAG

1951 GAGCTGGATG AGCGCGAGCT GATCGATATC GAGGTGAGCG GCAACCACCT

2001 GTTCTACGCC AACGATATCC TGACCCACAA CAGCAATGCA GTGAGCCTGG

2051 TAATGCGTCG AATACGCAAG GAAAACGTGG ACGCCGGCGA GAGGGCCAAA

2101 CAGGCGTTGG CTTTCGAACG CACCGATTTC GATCAGGTGC GGTCGTTGAT

2151 GGAGAACAGC GATCGATGCC AGGACATCCG GAACTTGGCG TTTTTGGGCA

2201 TTGCCTATAA CACCCTCCTT CGCATCGCCG AGATAGCGCG TATCCGAGTT

2251 AAAGACATTT CCCGTACAGA CGGAGGCCGC ATGCTTATTC ACATCGGACG

2301 CACGAAAACC CTGGTCAGCA CTGCTGGCGT GGAGAAAGCA CTTTCCCTGG

2351 GTGTGACGAA ATTGGTCGAG CGATGGATCA GTGTGAGTGG CGTCGCCGAT

2401 GACCCCAACA ATTATCTGTT CTGCAGGGTG CGCAAAAATG GAGTGGCAGC

2451 CCCCTCGGCA ACAAGTCAGT TGAGTACGCG CGCGTTGGAG GGAATCTTCG

2501 AGGCCACTCA CCGATTGATT TACGGTGCAA AAGATGACTC CGGCCAACGT

2551 TACTTGGCCT GGTCCGGTCA CAGCGCACGT GTGGGTGCCG CCCGTGACAT

2601 GGCGAGGGCC GGTGTTTCCA TCCCAGAAAT CATGCAAGCG GGCGGATGGA

2651 CCAACGTGAA TATTGTAATG AACTACATTC GTAATCTGGA TAGTGAAACA

2701 GGCGCAATGG TGCGCCTGCT GGAGGACGGA GATTAAACCG GTGTTTAAAC

2751 AATCAACCTC TGGATTACAA AATTTGTGAA AGATTGACTG GTATTCTTAA

2801 CTATGTTGCT CCTTTTACGC TATGTGGATA CGCTGCTTTA ATGCCTTTGT

2851 ATCATGCTAT TGCTTCCCGT ATGGCTTTCA TTTTCTCCTC CTTGTATAAA

2901 TCCTGGTTGC TGTCTCTTTA TGAGGAGTTG TGGCCCGTTG TCAGGCAACG

2951 TGGCGTGGTG TGCACTGTGT TTGCTGACGC AACCCCCACT GGTTGGGGCA

3001 TTGCCACCAC CTGTCAGCTC CTTTCCGGGA CTTTCGCTTT CCCCCTCCCT

3051 ATTGCCACGG CGGAACTCAT CGCCGCCTGC CTTGCCCGCT GCTGGACAGG

3101 GGCTCGGCTG TTGGGCACTG ACAATTCCGT GGTGTTGTCG GGGAAATCAT

3151 CGTCCTTTCC TTGGCTGCTC GCCTGTGTTG CCACCTGGAT TCTGCGCGGG

3201 ACGTCCTTCT GCTACGTCCC TTCGGCCCTC AATCCAGCGG ACCTTCCTTC

3251 CCGCGGCCTG CTGCCGGCTC TGCGGCCTCT TCCGCGTCTT CGCCTTCGCC

3301 CTCAGACGAG TCGGATCTCC CTTTGGGCCG CCTCCCCGCC TGAAGCTTAT

3351 CGATACCGTC GACTAAAGCC AAATAGAAAA TTATTCAGTT CCTGGCTTAA

3401 GTTTTTAAAA GTGATATTAT TTATTTGGTT GTAACCAACC AAAAGAATGT

3451 AAATAACTAA TACATAATTA TGTTAGTTTT AAGTTAGCAA CAAATTGATT

3501 TTAGCTATAT TAGCTACTTG GTTAATAAAT AGAATATATT TATTTAAAGA

3551 TAATTGCGTT TTTATTGTCA GGGAGTGAGT TTGCTTAAAA ACTCGTTTAG

3601 ATCCACTAGT TCTAGAGCGG CCGCTGGCCA CGGGTGCGCA TGATCGTGCT

3651 CCTGTCGTTG AGGACCCGGC TAGGCTGGCG GGGTTGCCTT ACTGGTTAGC

3701 AGAATGAATC ACCGATACGC GAGCGAACGT GAAGCGACTG CTGCTGCAAA

3751 ACGTCTGCGA CCTGAGCAAC AACATGAATG GTCTTCGGTT TCCGTGTTTC

3801 GTAAAGTCTG GAAACGCGGA AGTCAGCGCC CTGCACCATT ATGTTCCGGA

3851 GGCGCGCCCT AGTTCCAGTG AAATCCAAGC ATTTTCTAAA TTAAATGTAT

3901 TCTTATTATT ATAGTTGTTA TTTTTGATAT ATATAAACAA CACTATTATG

3951 CCCACCATTT TTTTGAGATG CATCTACACA AGGAACAAAC ACTGGATGTC

4001 ACTTTCAGTT CAAATTGTAA CGCTAATCAC TCCGAACAGG TCACAAAAAA

4051 TTACCTTAAA AAGTCATAAT ATTAAATTAG AATAAATATA GCTGTGAGGG

4101 AAATATATAC AAATATATTG GAGCAAATAA ATTGTACATA CAAATATTTA

4151 TTACTAATTT CTATTGAGAC GAAATGAACC ACTCGGAACC ATTTGAGCGA

4201 ACCGAATCGC GCGGAACTAA CGACAGTCGC TCCAAGGTCG TCGAACAAAA

4251 GGTGAATGTG TTGCGGAGAG CGGGTGGGAG ACAGCGAAAG AGCAACTACG

4301 AAACGTGGTG TGGTGGAGGT GAATTATGAA GAGGGCGCGC GATTTGAAAA

4351 GTATGTATAT AAAAAATATA TCCCGGTGTT TTATGTAGCG ATAAACGAGT

4401 TTTTGATGTA AGGTATGCAG GTGTGTAAGT CTTTTGGTTA GAAGACAAAT

4451 CCAAAGTCTA CTTGTGGGGA TGTTCGAAGG GGAAATACTT GTATTCTATA

4501 GGTCATATCT TGTTTTTATT GGCACAAATA TAATTACATT AGCTTTTTGA

4551 GGGGGCAATA AACAGTAAAC ACGATGGTAA TAATGGTAAA AAAAAAAAAC

4601 AAGCAGTTAT TTCGGATATA TGTCGGCTAC TCCTTGCGTC GGGCCCGAAG

4651 TCTTAGAGCC AGATATGCGA GCACCCGGAA GCTCACGATG AGAATGGCCA

4701 GACCCACGTA GTCCAGCGGC AGATCGGCGG CGGAGAAGTT AAGCGTCTCC

4751 AGGATGACCT TGCCCGAACT GGGGCACGTG GTGTTCGACG ATGTGCAGCT

4801 AATTTCGCCC GGCTCCACGT CCGCCCATTG GTTAATCAGC AGACCCTCGT

4851 TGGCGTAACG GAACCATGAG AGGTACGACA ACCATTTGAG GTATACTGGC

4901 ACCGAGCCCG AGTTCAAGAA GAAGCCGCCA AAGAGCAGGA ATGGTATGAT

4951 AACCGGCGGA CCCACAGACA GCGCCATCGA GGTCGAGGAG CTGGCGCAGG

5001 ATATTAGATA TCCGAAGGAC GTTGACACAT TGGCCACCAG AGTGACCAGC

5051 GCCAGGCAGT TGAAGAAGTG CAGCACTCCG GCCCGCAGTC CGATCATCGG

5101 ATAGGCAATC GCCGTGAAGA CCAGTGGCAC TGTGAGAAAA AGCGGCAATT

5151 CGGCAATCGT TTTGCCCAGA AAGTATGTGT CACAGCGATA AAGTCGACTT

5201 CGGGCCTCCC TCATAAAAAC TGGCAGCTCT GAGGTGAACA CCTAAATCGA

5251 ATCGATTCAT TAGAAAGTTA GTAAATTATT GAAATGCAAA TGTATTCTAA

5301 ACATGACTTA CATTTATCGT GGCAAAGACG TTTTGAAAGG TCATGTTGGT

5351 CAGGAAGAGG AAGATGGCTC CGTTGATATT CATCACACCC ACTTGCGTGA

5401 GTTGTTGGCC CAAAAAGATG AGGCCAATCA AGATGGCAAC CATCTGCAAA

5451 TTAAAATGTT ACTCGCATCT CATTAATATT CGCGAGTTAA ATGAAATTTA

5501 TTTATCTTCT GCAAAACTAT AAACTATACA TCTCATTGAA AAAAACTAAG

5551 AAGGGTGTGG AATCAGGCAA TTCTATCTAA AATCTAGCGA ATTTGTTTCC

5601 AAGAATTGTA AGCGTTATAT CATTTGTTTC CACTGGAACC ACTCACCGTT

5651 GTCTGAATAA GTCGCACTTT TACGAGGAGT GGTTCCTTGA GCACCGACAG

5701 CCAGGATCGC CACAGGACCG CCCGGAACTG CATGAACCAG GTGGCCTTGT

5751 AGGTGTACCC ATTCTCCGGC TGCTCCAGTG GCTTCTCCAG ATTTTTGGTG

5801 GCCAACAACT GCTCCATATC CCGGGCTACT TTGCTAATGG CAAAATTGTC

5851 GCATATCTTG GCGATCCGAT CACGGGACTC GATCTCCCGT CCGGGCACAA

5901 CGGCCAACAC CTGTACGTAA AAGTCCGCCG GATTGTAGTT GGTAGGACAC

5951 TGGGCACCCA CGCTGGATAG GAGTTGAGAT GTTATGTAAT ACTAGATACC

6001 CTTAATAAAC ACATCGAACT CACTAGGAAA AGAAGTCGAC GGCTTCGCTG

6051 GGAGTGCCCA AGAAAGCTAC CCTGCCCTCG GCCATCAGAA GGATCTTGTC

6101 AAAGAGCTCA AACAGCTCGG AAGACGGCTG ATGAATGGTC AGGATGACGG

6151 TCTTGCCCTT CTGCGACAGC TTCTTCAGCA CCTGGACGAC GCTGTGGGCG

6201 GTAAAGGAGT CCAGTCCGGA GGTGGGCTCA TCGCAGATCA GAAGCGGCGG

6251 ATCGGTTAGA GCCTCGGAGG CGAATGCCAG ACGCTTCCTT TCTCCGCCGG

6301 ACAGACCTTT CACCCTGCCG GGCACACCGA TGATCGTGTG CTGACATTTG

6351 CTGAGCGAAA GCTCCTGGAT CACCTGATCC ACGCGGGCCA CTCGCTGCCG

6401 ATAGGTCAGA TGTCGTGGCA TCCGCACCAT GGCTTGGAAA ATCAGGTGTT

6451 CCCTGGCCGT TAGGGAGCCG ATAAAGAGGT CATCCTGCTG GACATAGGCG

6501 CACCTGGCCT GCATCTCCTT GGCGTCCACA GGTTGGCCAT TGAGCAGTCG

6551 CATCCCGGAT GGCGATACTT GGATGCCCTG CGGCGATCGA AAGGCAAGGG

6601 CATTCAGCAG GGTCGTCTTT CCGGCACCGG AACTGCCCAT CACGGCCAAA

6651 AGTTCGCCCG GATAGGCCAC GCCGCAAACT GAGTTTCAAA TTGGTAATTG

6701 GACCCTTTAT TAAGATTTCA CACAGATCAG CCGACTGCGA ATAGAAACTC

6751 ACCGTTCTTG AGCAAATGTT TCCTGGGCGC CGGTATGTGT CGCTCGTTGC

6801 AGAATAGTCC GCGTGTCCGG TTGACCAGCT GCCGCCATCC GGAGCCCGGC

6851 TGATTGACCG CCCCAAAGAT GTCCATATTG TGCCAGGCAT AGGTGAGGTT

6901 CTCGGCTAGT TGGCCGCTCC CTGAACCGGA GTCCTCCGGC GGACTGGGTG

6951 GCCGGAGCGT GCCGTAGTTT TTGGCCTGCC CGAAGCCCTG GTTAATGCAG

7001 CTCTGCGAAG CCGCTCCGCT GTCACCCTGC AATGATAGGG GATCTCAAAT

7051 ATCAACTACT AGCGTTATGC TCATCTAACC CCGAACAAAA AGTACCCCGA

7101 AGTATCCTAC GAAGTAGGTT TATACTTTTA TTTATTTTTT GTGCATCTAG

7151 GATCAGCTTA AAATATCTGG TTGTTATATT TTTTGTAAAA AAGAATATAG

7201 TCGAAAATGA ATGCCTTTAG ATGTCTTGAT CATGATATGA TCTCAAAAAT

7251 TGTCTTATAT AGCGAGAACA GCTACCAGAA TAATCTGTTT CGTGTCACTA

7301 TTTGTTTGTG CAATTGCGGT TTGGGATTTT TGTGGGTCGC AGTTCTCACG

7351 CCGCATACAA TTTGATGTTG CAATCGCAGT TCCTATAGAT CAAGTGAACT

7401 TAAGATGTAT GCACATGTAC TACTCACATT GTTCAGATGC TCGGCAGATG

7451 GGTGTTTGCT GCCTCCGCGA ATTAATAGCT CCTGATCCTC TTGGCCCATT

7501 GCCGGGATTT TTCACACTTT CCCCTGCTTA CCCACCCAAA ACCAATCACC

7551 ACCCCAATCA CTCAAAAAAC AAACAAAAAT AAGAAGCGAG AGGAGTTTTG

7601 GCACAGCACT TTGTGTTTAA TTGATGGCGT AAACCGCTTG GAGCTTCGTC

7651 ACGAAACCGC TGACAAAATG CAACTGAAGG CGGACATTGA CGCTACGTAA

7701 CGCTACAAAC GGTGGCGAAA GAGATAGCGG ACGCAGCGGC GAAAGAGACG

7751 GCGATATTTC TGTGGACAGA GAAGGAGGCA AACAGCGCTG ACTTTGAGTG

7801 GAATGTCATT TTGAGTGAGA GGTAATCGAA AGAACCTGGT ACATCAAATA

7851 CCCTTGGATC GAAGTAAATT TAAAACTGAT CAGATAAGTT CAATGATATC

7901 CAGTGCAGTA AAAAAAAAAA ATGTTTTTTT TATCTACTTT CCGCAAAAAT

7951 GGGTTTTATT AACTTACATA CATACTAGGC GCGCCCATAT GTTCGGCTTG

8001 TCGACATGCC CGCCGTGACC GTCGAGAACC CGCTGACGCT GCCCCGCGTA

8051 TCCGCACCCG CCGACGCCGT CGCACGTCCC GTGCTCACCG TGACCACCGC

8101 GCCCAGCGGT TTCGAGGGCG AGGGCTTCCC GGTGCGCCGC GCGTTCGCCG

8151 GGATCAACTA CCGCCACCTC GACCCGTTCA TCATGATGGA CCAGATGGGT

8201 GAGGTGGAGT ACGCGCCCGG GGAGCCCAAG GGCACGCCCT GGCACCCGCA

8251 CCGCGGCTTC GAGACCGTGA CCTACATCGT CGACGGTAAC ATGTGAGCAA

8301 AAGGCCAGCA AAAGGCCAGG AACCGTAAAA AGGCCGCGTT GCTGGCGTTT

8351 TTCCATAGGC TCCGCCCCCC TGACGAGCAT CACAAAAATC GACGCTCAAG

8401 TCAGAGGTGG CGAAACCCGA CAGGACTATA AAGATACCAG GCGTTTCCCC

8451 CTGGAAGCTC CCTCGTGCGC TCTCCTGTTC CGACCCTGCC GCTTACCGGA

8501 TACCTGTCCG CCTTTCTCCC TTCGGGAAGC GTGGCGCTTT CTCATAGCTC

8551 ACGCTGTAGG TATCTCAGTT CGGTGTAGGT CGTTCGCTCC AAGCTGGGCT

8601 GTGTGCACGA ACCCCCCGTT CAGCCCGACC GCTGCGCCTT ATCCGGTAAC

8651 TATCGTCTTG AGTCCAACCC GGTAAGACAC GACTTATCGC CACTGGCAGC

8701 AGCCACTGGT AACAGGATTA GCAGAGCGAG GTATGTAGGC GGTGCTACAG

8751 AGTTCTTGAA GTGGTGGCCT AACTACGGCT ACACTAGAAG AACAGTATTT

8801 GGTATCTGCG CTCTGCTGAA GCCAGTTACC TTCGGAAAAA GAGTTGGTAG

8851 CTCTTGATCC GGCAAACAAA CCACCGCTGG TAGCGGTGGT TTTTTTGTTT

8901 GCAAGCAGCA GATTACGCGC AGAAAAAAAG GATCTCAAGA AGATCCTTTG

8951 ATCTTTTCTA CGGGGTCTGA CGCTCAGTGG AACGAAAACT CACGTTAAGG

9001 GATTTTGGTC ATGAGATTAT CAAAAAGGAT CTTCACCTAG ATCCTTTTAA

9051 ATTAAAAATG AAGTTTTAAA TCAATCTAAA GTATATATGA GTAAACTTGG

9101 TCTGACAGTT ACCAATGCTT AATCAGTGAG GCACCTATCT CAGCGATCTG

9151 TCTATTTCGT TCATCCATAG TTGCCTGACT CCCCGTCGTG TAGATAACTA

9201 CGATACGGGA GGGCTTACCA TCTGGCCCCA GTGCTGCAAT GATACCGCGA

9251 GACCCACGCT CACCGGCTCC AGATTTATCA GCAATAAACC AGCCAGCCGG

9301 AAGGGCCGAG CGCAGAAGTG GTCCTGCAAC TTTATCCGCC TCCATCCAGT

9351 CTATTAATTG TTGCCGGGAA GCTAGAGTAA GTAGTTCGCC AGTTAATAGT

9401 TTGCGCAACG TTGTTGCCAT TGCTACAGGC ATCGTGGTGT CACGCTCGTC

9451 GTTTGGTATG GCTTCATTCA GCTCCGGTTC CCAACGATCA AGGCGAGTTA

9501 CATGATCCCC CATGTTGTGC AAAAAAGCGG TTAGCTCCTT CGGTCCTCCG

9551 ATCGTTGTCA GAAGTAAGTT GGCCGCAGTG TTATCACTCA TGGTTATGGC

9601 AGCACTGCAT AATTCTCTTA CTGTCATGCC ATCCGTAAGA TGCTTTTCTG

9651 TGACTGGTGA GTACTCAACC AAGTCATTCT GAGAATAGTG TATGCGGCGA

9701 CCGAGTTGCT CTTGCCCGGC GTCAATACGG GATAATACCG CGCCACATAG

9751 CAGAACTTTA AAAGTGCTCA TCATTGGAAA ACGTTCTTCG GGGCGAAAAC

9801 TCTCAAGGAT CTTACCGCTG TTGAGATCCA GTTCGATGTA ACCCACTCGT

9851 GCACCCAACT GATCTTCAGC ATCTTTTACT TTCACCAGCG TTTCTGGGTG

9901 AGCAAAAACA GGAAGGCAAA ATGCCGCAAA AAAGGGAATA AGGGCGACAC

9951 GGAAATGTTG AATACTCATA CTCTTCCTTT TTCAATATTA TTGAAGCATT

10001 TATCAGGGTT ATTGTCTCAT GAGCGGATAC ATATTTGAAT GTATTTAGAA

10051 AAATAAACAA ATAGGGGTTC CGCGCACATT TCCCCGAAAA GTGCCACCTG

10101 AGTC

**HJP-194-IVS-Syn21-Cre_A_-gp41-1^N^-WPREUw**

1 TCTAAGAAAC CATTATTATC ATGACATTAA CCTATAAAAA TAGGCGTATC

51 ACGAGGCCCT TTCGTCTTCA AGAATTCGTT TATCACAAGT TTGTACAAAA

101 AAGCTGAACG AGAAACGTAA AATGATATAA ATATCAATAT ATTAAATTAG

151 ATTTTGCATA AAAAACAGAC TACATAATAC TGTAAAACAC AACATATCCA

201 GTCACTATGG CGGCCGCATT AGGCACCCCA GGCTTTACAC TTTATGCTTC

251 CGGCTCGTAT AATGTGTGGA TTTTGAGTTA GGATCCGTCG AGATTTTCAG

301 GAGCTAAGGA AGCTAAAATG GAGAAAAAAA TCACTGGATA TACCACCGTT

351 GATATATCCC AATGGCATCG TAAAGAACAT TTTGAGGCAT TTCAGTCAGT

401 TGCTCAATGT ACCTATAACC AGACCGTTCA GCTGGATATT ACGGCCTTTT

451 TAAAGACCGT AAAGAAAAAT AAGCACAAGT TTTATCCGGC CTTTATTCAC

501 ATTCTTGCCC GCCTGATGAA TGCTCATCCG GAATTCCGTA TGGCAATGAA

551 AGACGGTGAG CTGGTGATAT GGGATAGTGT TCACCCTTGT TACACCGTTT

601 TCCATGAGCA AACTGAAACG TTTTCATCGC TCTGGAGTGA ATACCACGAC

651 GATTTCCGGC AGTTTCTACA CATATATTCG CAAGATGTGG CGTGTTACGG

701 TGAAAACCTG GCCTATTTCC CTAAAGGGTT TATTGAGAAT ATGTTTTTCG

751 TCTCAGCCAA TCCCTGGGTG AGTTTCACCA GTTTTGATTT AAACGTGGCC

801 AATATGGACA ACTTCTTCGC CCCCGTTTTC ACCATGGGCA AATATTATAC

851 GCAAGGCGAC AAGGTGCTGA TGCCGCTGGC GATTCAGGTT CATCATGCCG

901 TTTGTGATGG CTTCCATGTC GGCAGAATGC TTAATGAATT ACAACAGTAC

951 TGCGATGAGT GGCAGGGCGG GGCGTAAACG CGTGGATCCG GCTTACTAAA

1001 AGCCAGATAA CAGTATGCGT ATTTGCGCGC TGATTTTTGC GGTATAAGAA

1051 TATATACTGA TATGTATACC CGAAGTATGT CAAAAAGAGG TATGCTATGA

1101 AGCAGCGTAT TACAGTGACA GTTGACAGCG ACAGCTATCA GTTGCTCAAG

1151 GCATATATGA TGTCAATATC TCCGGTCTGG TAAGCACAAC CATGCAGAAT

1201 GAAGCCCGTC GTCTGCGTGC CGAACGCTGG AAAGCGGAAA ATCAGGAAGG

1251 GATGGCTGAG GTCGCCCGGT TTATTGAAAT GAACGGCTCT TTTGCTGACG

1301 AGAACAGGGG CTGGTGAAAT GCAGTTTAAG GTTTACACCT ATAAAAGAGA

1351 GAGCCGTTAT CGTCTGTTTG TGGATGTACA GAGTGATATT ATTGACACGC

1401 CCGGGCGACG GATGGTGATC CCCCTGGCCA GTGCACGTCT GCTGTCAGAT

1451 AAAGTCTCCC GTGAACTTTA CCCGGTGGTG CATATCGGGG ATGAAAGCTG

1501 GCGCATGATG ACCACCGATA TGGCCAGTGT GCCGGTCTCC GTTATCGGGG

1551 AAGAAGTGGC TGATCTCAGC CACCGCGAAA ATGACATCAA AAACGCCATT

1601 AACCTGATGT TCTGGGGAAT ATAAATGTCA GGCTCCCTTA TACACAGCCA

1651 GTCTGCAGGT CGACCATAGT GACTGGATAT GTTGTGTTTT ACAGTATTAT

1701 GTAGTCTGTT TTTTATGCAA AATCTAATTT AATATATTGA TATTTATATC

1751 ATTTTACGTT TCTCGTTCAG CTTTCTTGTA CAAAGTGGTG ATAAACGGCC

1801 GGCCGAGCTC GCCCGGGGAT CGAGCGCAGC GGTATAAAAG GGCGCGGGGT

1851 GGCTGAGAGC ATCAGTTGTG AATGAATGTT CGAGCCGAGC AGACGTGCCG

1901 CTGCCTTCGT TAATATCCTT TGAATAAGCC AACTTTGAAT CACAAGACGC

1951 ATACCAAACG GTACCGCTAG AAAAAGGTAG GTTCAACCAC TGATGCCTAG

2001 GCACACCGAA ACGACTAACC CTAATTCTTA TCCTTTACTT CAGGGCTAGC

2051 AACTTAAAAA AAAAAATCAA AATGAGTAAC TTGCTCACGG TACACCAAAA

2101 TTTGCCGGCC CTGCCTGTGG ACGCCACGTC CGACGAGGTC CGAAAGAATC

2151 TGATGGACAT GTTCCGTGAC AGGCAGGCCT TTAGCGAGCA CACCTGGAAG

2201 ATGCTGCTCT CGGTGTGCAG GTCCTGGGCC GCTTGGTGCA AGCTGAACAA

2251 CCGAAAGTGG TTTCCCGCGG AGCCCGAGGA CGTCCGCGAT TATCTGCTGT

2301 ACCTGCAGGC TCGCGGACTG GCCGTCAAAA CAATACAGCA ACATTTGGGT

2351 CAGCTGAACA TGCTCCACAG GCGCAGCGGA CTGCCCCGGC CATCCGATTG

2401 CCTGGATCTG AAGACCCAGG TGCAGACCCC CCAGGGCATG AAGGAGATCA

2451 GCAACATCCA GGTGGGCGAT CTGGTGCTGA GCAACACCGG CTACAACGAG

2501 GTGCTGAACG TGTTCCCCAA GAGCAAGAAG AAGAGCTACA AGATCACCCT

2551 GGAGGATGGC AAGGAGATCA TCTGCAGCGA GGAGCACCTG TTCCCCACCC

2601 AGACCGGCGA GATGAACATC AGCGGCGGCC TGAAGGAGGG CATGTGCCTG

2651 TACGTGAAGG AGTAAACCGG TGTTTAAACA ATCAACCTCT GGATTACAAA

2701 ATTTGTGAAA GATTGACTGG TATTCTTAAC TATGTTGCTC CTTTTACGCT

2751 ATGTGGATAC GCTGCTTTAA TGCCTTTGTA TCATGCTATT GCTTCCCGTA

2801 TGGCTTTCAT TTTCTCCTCC TTGTATAAAT CCTGGTTGCT GTCTCTTTAT

2851 GAGGAGTTGT GGCCCGTTGT CAGGCAACGT GGCGTGGTGT GCACTGTGTT

2901 TGCTGACGCA ACCCCCACTG GTTGGGGCAT TGCCACCACC TGTCAGCTCC

2951 TTTCCGGGAC TTTCGCTTTC CCCCTCCCTA TTGCCACGGC GGAACTCATC

3001 GCCGCCTGCC TTGCCCGCTG CTGGACAGGG GCTCGGCTGT TGGGCACTGA

3051 CAATTCCGTG GTGTTGTCGG GGAAATCATC GTCCTTTCCT TGGCTGCTCG

3101 CCTGTGTTGC CACCTGGATT CTGCGCGGGA CGTCCTTCTG CTACGTCCCT

3151 TCGGCCCTCA ATCCAGCGGA CCTTCCTTCC CGCGGCCTGC TGCCGGCTCT

3201 GCGGCCTCTT CCGCGTCTTC GCCTTCGCCC TCAGACGAGT CGGATCTCCC

3251 TTTGGGCCGC CTCCCCGCCT GAAGCTTATC GATACCGTCG ACTAAAGCCA

3301 AATAGAAAAT TATTCAGTTC CTGGCTTAAG TTTTTAAAAG TGATATTATT

3351 TATTTGGTTG TAACCAACCA AAAGAATGTA AATAACTAAT ACATAATTAT

3401 GTTAGTTTTA AGTTAGCAAC AAATTGATTT TAGCTATATT AGCTACTTGG

3451 TTAATAAATA GAATATATTT ATTTAAAGAT AATTGCGTTT TTATTGTCAG

3501 GGAGTGAGTT TGCTTAAAAA CTCGTTTAGA TCCACTAGTT CTAGAGCGGC

3551 CGCTGGCCAC GGGTGCGCAT GATCGTGCTC CTGTCGTTGA GGACCCGGCT

3601 AGGCTGGCGG GGTTGCCTTA CTGGTTAGCA GAATGAATCA CCGATACGCG

3651 AGCGAACGTG AAGCGACTGC TGCTGCAAAA CGTCTGCGAC CTGAGCAACA

3701 ACATGAATGG TCTTCGGTTT CCGTGTTTCG TAAAGTCTGG AAACGCGGAA

3751 GTCAGCGCCC TGCACCATTA TGTTCCGGAG GCGCGCCCTA GTTCCAGTGA

3801 AATCCAAGCA TTTTCTAAAT TAAATGTATT CTTATTATTA TAGTTGTTAT

3851 TTTTGATATA TATAAACAAC ACTATTATGC CCACCATTTT TTTGAGATGC

3901 ATCTACACAA GGAACAAACA CTGGATGTCA CTTTCAGTTC AAATTGTAAC

3951 GCTAATCACT CCGAACAGGT CACAAAAAAT TACCTTAAAA AGTCATAATA

4001 TTAAATTAGA ATAAATATAG CTGTGAGGGA AATATATACA AATATATTGG

4051 AGCAAATAAA TTGTACATAC AAATATTTAT TACTAATTTC TATTGAGACG

4101 AAATGAACCA CTCGGAACCA TTTGAGCGAA CCGAATCGCG CGGAACTAAC

4151 GACAGTCGCT CCAAGGTCGT CGAACAAAAG GTGAATGTGT TGCGGAGAGC

4201 GGGTGGGAGA CAGCGAAAGA GCAACTACGA AACGTGGTGT GGTGGAGGTG

4251 AATTATGAAG AGGGCGCGCG ATTTGAAAAG TATGTATATA AAAAATATAT

4301 CCCGGTGTTT TATGTAGCGA TAAACGAGTT TTTGATGTAA GGTATGCAGG

4351 TGTGTAAGTC TTTTGGTTAG AAGACAAATC CAAAGTCTAC TTGTGGGGAT

4401 GTTCGAAGGG GAAATACTTG TATTCTATAG GTCATATCTT GTTTTTATTG

4451 GCACAAATAT AATTACATTA GCTTTTTGAG GGGGCAATAA ACAGTAAACA

4501 CGATGGTAAT AATGGTAAAA AAAAAAAACA AGCAGTTATT TCGGATATAT

4551 GTCGGCTACT CCTTGCGTCG GGCCCGAAGT CTTAGAGCCA GATATGCGAG

4601 CACCCGGAAG CTCACGATGA GAATGGCCAG ACCCACGTAG TCCAGCGGCA

4651 GATCGGCGGC GGAGAAGTTA AGCGTCTCCA GGATGACCTT GCCCGAACTG

4701 GGGCACGTGG TGTTCGACGA TGTGCAGCTA ATTTCGCCCG GCTCCACGTC

4751 CGCCCATTGG TTAATCAGCA GACCCTCGTT GGCGTAACGG AACCATGAGA

4801 GGTACGACAA CCATTTGAGG TATACTGGCA CCGAGCCCGA GTTCAAGAAG

4851 AAGCCGCCAA AGAGCAGGAA TGGTATGATA ACCGGCGGAC CCACAGACAG

4901 CGCCATCGAG GTCGAGGAGC TGGCGCAGGA TATTAGATAT CCGAAGGACG

4951 TTGACACATT GGCCACCAGA GTGACCAGCG CCAGGCAGTT GAAGAAGTGC

5001 AGCACTCCGG CCCGCAGTCC GATCATCGGA TAGGCAATCG CCGTGAAGAC

5051 CAGTGGCACT GTGAGAAAAA GCGGCAATTC GGCAATCGTT TTGCCCAGAA

5101 AGTATGTGTC ACAGCGATAA AGTCGACTTC GGGCCTCCCT CATAAAAACT

5151 GGCAGCTCTG AGGTGAACAC CTAAATCGAA TCGATTCATT AGAAAGTTAG

5201 TAAATTATTG AAATGCAAAT GTATTCTAAA CATGACTTAC ATTTATCGTG

5251 GCAAAGACGT TTTGAAAGGT CATGTTGGTC AGGAAGAGGA AGATGGCTCC

5301 GTTGATATTC ATCACACCCA CTTGCGTGAG TTGTTGGCCC AAAAAGATGA

5351 GGCCAATCAA GATGGCAACC ATCTGCAAAT TAAAATGTTA CTCGCATCTC

5401 ATTAATATTC GCGAGTTAAA TGAAATTTAT TTATCTTCTG CAAAACTATA

5451 AACTATACAT CTCATTGAAA AAAACTAAGA AGGGTGTGGA ATCAGGCAAT

5501 TCTATCTAAA ATCTAGCGAA TTTGTTTCCA AGAATTGTAA GCGTTATATC

5551 ATTTGTTTCC ACTGGAACCA CTCACCGTTG TCTGAATAAG TCGCACTTTT

5601 ACGAGGAGTG GTTCCTTGAG CACCGACAGC CAGGATCGCC ACAGGACCGC

5651 CCGGAACTGC ATGAACCAGG TGGCCTTGTA GGTGTACCCA TTCTCCGGCT

5701 GCTCCAGTGG CTTCTCCAGA TTTTTGGTGG CCAACAACTG CTCCATATCC

5751 CGGGCTACTT TGCTAATGGC AAAATTGTCG CATATCTTGG CGATCCGATC

5801 ACGGGACTCG ATCTCCCGTC CGGGCACAAC GGCCAACACC TGTACGTAAA

5851 AGTCCGCCGG ATTGTAGTTG GTAGGACACT GGGCACCCAC GCTGGATAGG

5901 AGTTGAGATG TTATGTAATA CTAGATACCC TTAATAAACA CATCGAACTC

5951 ACTAGGAAAA GAAGTCGACG GCTTCGCTGG GAGTGCCCAA GAAAGCTACC

6001 CTGCCCTCGG CCATCAGAAG GATCTTGTCA AAGAGCTCAA ACAGCTCGGA

6051 AGACGGCTGA TGAATGGTCA GGATGACGGT CTTGCCCTTC TGCGACAGCT

6101 TCTTCAGCAC CTGGACGACG CTGTGGGCGG TAAAGGAGTC CAGTCCGGAG

6151 GTGGGCTCAT CGCAGATCAG AAGCGGCGGA TCGGTTAGAG CCTCGGAGGC

6201 GAATGCCAGA CGCTTCCTTT CTCCGCCGGA CAGACCTTTC ACCCTGCCGG

6251 GCACACCGAT GATCGTGTGC TGACATTTGC TGAGCGAAAG CTCCTGGATC

6301 ACCTGATCCA CGCGGGCCAC TCGCTGCCGA TAGGTCAGAT GTCGTGGCAT

6351 CCGCACCATG GCTTGGAAAA TCAGGTGTTC CCTGGCCGTT AGGGAGCCGA

6401 TAAAGAGGTC ATCCTGCTGG ACATAGGCGC ACCTGGCCTG CATCTCCTTG

6451 GCGTCCACAG GTTGGCCATT GAGCAGTCGC ATCCCGGATG GCGATACTTG

6501 GATGCCCTGC GGCGATCGAA AGGCAAGGGC ATTCAGCAGG GTCGTCTTTC

6551 CGGCACCGGA ACTGCCCATC ACGGCCAAAA GTTCGCCCGG ATAGGCCACG

6601 CCGCAAACTG AGTTTCAAAT TGGTAATTGG ACCCTTTATT AAGATTTCAC

6651 ACAGATCAGC CGACTGCGAA TAGAAACTCA CCGTTCTTGA GCAAATGTTT

6701 CCTGGGCGCC GGTATGTGTC GCTCGTTGCA GAATAGTCCG CGTGTCCGGT

6751 TGACCAGCTG CCGCCATCCG GAGCCCGGCT GATTGACCGC CCCAAAGATG

6801 TCCATATTGT GCCAGGCATA GGTGAGGTTC TCGGCTAGTT GGCCGCTCCC

6851 TGAACCGGAG TCCTCCGGCG GACTGGGTGG CCGGAGCGTG CCGTAGTTTT

6901 TGGCCTGCCC GAAGCCCTGG TTAATGCAGC TCTGCGAAGC CGCTCCGCTG

6951 TCACCCTGCA ATGATAGGGG ATCTCAAATA TCAACTACTA GCGTTATGCT

7001 CATCTAACCC CGAACAAAAA GTACCCCGAA GTATCCTACG AAGTAGGTTT

7051 ATACTTTTAT TTATTTTTTG TGCATCTAGG ATCAGCTTAA AATATCTGGT

7101 TGTTATATTT TTTGTAAAAA AGAATATAGT CGAAAATGAA TGCCTTTAGA

7151 TGTCTTGATC ATGATATGAT CTCAAAAATT GTCTTATATA GCGAGAACAG

7201 CTACCAGAAT AATCTGTTTC GTGTCACTAT TTGTTTGTGC AATTGCGGTT

7251 TGGGATTTTT GTGGGTCGCA GTTCTCACGC CGCATACAAT TTGATGTTGC

7301 AATCGCAGTT CCTATAGATC AAGTGAACTT AAGATGTATG CACATGTACT

7351 ACTCACATTG TTCAGATGCT CGGCAGATGG GTGTTTGCTG CCTCCGCGAA

7401 TTAATAGCTC CTGATCCTCT TGGCCCATTG CCGGGATTTT TCACACTTTC

7451 CCCTGCTTAC CCACCCAAAA CCAATCACCA CCCCAATCAC TCAAAAAACA

7501 AACAAAAATA AGAAGCGAGA GGAGTTTTGG CACAGCACTT TGTGTTTAAT

7551 TGATGGCGTA AACCGCTTGG AGCTTCGTCA CGAAACCGCT GACAAAATGC

7601 AACTGAAGGC GGACATTGAC GCTACGTAAC GCTACAAACG GTGGCGAAAG

7651 AGATAGCGGA CGCAGCGGCG AAAGAGACGG CGATATTTCT GTGGACAGAG

7701 AAGGAGGCAA ACAGCGCTGA CTTTGAGTGG AATGTCATTT TGAGTGAGAG

7751 GTAATCGAAA GAACCTGGTA CATCAAATAC CCTTGGATCG AAGTAAATTT

7801 AAAACTGATC AGATAAGTTC AATGATATCC AGTGCAGTAA AAAAAAAAAA

7851 TGTTTTTTTT ATCTACTTTC CGCAAAAATG GGTTTTATTA ACTTACATAC

7901 ATACTAGGCG CGCCCATATG TTCGGCTTGT CGACATGCCC GCCGTGACCG

7951 TCGAGAACCC GCTGACGCTG CCCCGCGTAT CCGCACCCGC CGACGCCGTC

8001 GCACGTCCCG TGCTCACCGT GACCACCGCG CCCAGCGGTT TCGAGGGCGA

8051 GGGCTTCCCG GTGCGCCGCG CGTTCGCCGG GATCAACTAC CGCCACCTCG

8101 ACCCGTTCAT CATGATGGAC CAGATGGGTG AGGTGGAGTA CGCGCCCGGG

8151 GAGCCCAAGG GCACGCCCTG GCACCCGCAC CGCGGCTTCG AGACCGTGAC

8201 CTACATCGTC GACGGTAACA TGTGAGCAAA AGGCCAGCAA AAGGCCAGGA

8251 ACCGTAAAAA GGCCGCGTTG CTGGCGTTTT TCCATAGGCT CCGCCCCCCT

8301 GACGAGCATC ACAAAAATCG ACGCTCAAGT CAGAGGTGGC GAAACCCGAC

8351 AGGACTATAA AGATACCAGG CGTTTCCCCC TGGAAGCTCC CTCGTGCGCT

8401 CTCCTGTTCC GACCCTGCCG CTTACCGGAT ACCTGTCCGC CTTTCTCCCT

8451 TCGGGAAGCG TGGCGCTTTC TCATAGCTCA CGCTGTAGGT ATCTCAGTTC

8501 GGTGTAGGTC GTTCGCTCCA AGCTGGGCTG TGTGCACGAA CCCCCCGTTC

8551 AGCCCGACCG CTGCGCCTTA TCCGGTAACT ATCGTCTTGA GTCCAACCCG

8601 GTAAGACACG ACTTATCGCC ACTGGCAGCA GCCACTGGTA ACAGGATTAG

8651 CAGAGCGAGG TATGTAGGCG GTGCTACAGA GTTCTTGAAG TGGTGGCCTA

8701 ACTACGGCTA CACTAGAAGA ACAGTATTTG GTATCTGCGC TCTGCTGAAG

8751 CCAGTTACCT TCGGAAAAAG AGTTGGTAGC TCTTGATCCG GCAAACAAAC

8801 CACCGCTGGT AGCGGTGGTT TTTTTGTTTG CAAGCAGCAG ATTACGCGCA

8851 GAAAAAAAGG ATCTCAAGAA GATCCTTTGA TCTTTTCTAC GGGGTCTGAC

8901 GCTCAGTGGA ACGAAAACTC ACGTTAAGGG ATTTTGGTCA TGAGATTATC

8951 AAAAAGGATC TTCACCTAGA TCCTTTTAAA TTAAAAATGA AGTTTTAAAT

9001 CAATCTAAAG TATATATGAG TAAACTTGGT CTGACAGTTA CCAATGCTTA

9051 ATCAGTGAGG CACCTATCTC AGCGATCTGT CTATTTCGTT CATCCATAGT

9101 TGCCTGACTC CCCGTCGTGT AGATAACTAC GATACGGGAG GGCTTACCAT

9151 CTGGCCCCAG TGCTGCAATG ATACCGCGAG ACCCACGCTC ACCGGCTCCA

9201 GATTTATCAG CAATAAACCA GCCAGCCGGA AGGGCCGAGC GCAGAAGTGG

9251 TCCTGCAACT TTATCCGCCT CCATCCAGTC TATTAATTGT TGCCGGGAAG

9301 CTAGAGTAAG TAGTTCGCCA GTTAATAGTT TGCGCAACGT TGTTGCCATT

9351 GCTACAGGCA TCGTGGTGTC ACGCTCGTCG TTTGGTATGG CTTCATTCAG

9401 CTCCGGTTCC CAACGATCAA GGCGAGTTAC ATGATCCCCC ATGTTGTGCA

9451 AAAAAGCGGT TAGCTCCTTC GGTCCTCCGA TCGTTGTCAG AAGTAAGTTG

9501 GCCGCAGTGT TATCACTCAT GGTTATGGCA GCACTGCATA ATTCTCTTAC

9551 TGTCATGCCA TCCGTAAGAT GCTTTTCTGT GACTGGTGAG TACTCAACCA

9601 AGTCATTCTG AGAATAGTGT ATGCGGCGAC CGAGTTGCTC TTGCCCGGCG

9651 TCAATACGGG ATAATACCGC GCCACATAGC AGAACTTTAA AAGTGCTCAT

9701 CATTGGAAAA CGTTCTTCGG GGCGAAAACT CTCAAGGATC TTACCGCTGT

9751 TGAGATCCAG TTCGATGTAA CCCACTCGTG CACCCAACTG ATCTTCAGCA

9801 TCTTTTACTT TCACCAGCGT TTCTGGGTGA GCAAAAACAG GAAGGCAAAA

9851 TGCCGCAAAA AAGGGAATAA GGGCGACACG GAAATGTTGA ATACTCATAC

9901 TCTTCCTTTT TCAATATTAT TGAAGCATTT ATCAGGGTTA TTGTCTCATG

9951 AGCGGATACA TATTTGAATG TATTTAGAAA AATAAACAAA TAGGGGTTCC

10001 GCGCACATTT CCCCGAAAAG TGCCACCTGA G

**HJP-195-IVS-Syn21-gp41-1^C^-Cre_B_-NrdJ-1^N^-WPREUw**

1 TAAGAAACCA TTATTATCAT GACATTAACC TATAAAAATA GGCGTATCAC

51 GAGGCCCTTT CGTCTTCAAG AATTCGTTTA TCACAAGTTT GTACAAAAAA

101 GCTGAACGAG AAACGTAAAA TGATATAAAT ATCAATATAT TAAATTAGAT

151 TTTGCATAAA AAACAGACTA CATAATACTG TAAAACACAA CATATCCAGT

201 CACTATGGCG GCCGCATTAG GCACCCCAGG CTTTACACTT TATGCTTCCG

251 GCTCGTATAA TGTGTGGATT TTGAGTTAGG ATCCGTCGAG ATTTTCAGGA

301 GCTAAGGAAG CTAAAATGGA GAAAAAAATC ACTGGATATA CCACCGTTGA

351 TATATCCCAA TGGCATCGTA AAGAACATTT TGAGGCATTT CAGTCAGTTG

401 CTCAATGTAC CTATAACCAG ACCGTTCAGC TGGATATTAC GGCCTTTTTA

451 AAGACCGTAA AGAAAAATAA GCACAAGTTT TATCCGGCCT TTATTCACAT

501 TCTTGCCCGC CTGATGAATG CTCATCCGGA ATTCCGTATG GCAATGAAAG

551 ACGGTGAGCT GGTGATATGG GATAGTGTTC ACCCTTGTTA CACCGTTTTC

601 CATGAGCAAA CTGAAACGTT TTCATCGCTC TGGAGTGAAT ACCACGACGA

651 TTTCCGGCAG TTTCTACACA TATATTCGCA AGATGTGGCG TGTTACGGTG

701 AAAACCTGGC CTATTTCCCT AAAGGGTTTA TTGAGAATAT GTTTTTCGTC

751 TCAGCCAATC CCTGGGTGAG TTTCACCAGT TTTGATTTAA ACGTGGCCAA

801 TATGGACAAC TTCTTCGCCC CCGTTTTCAC CATGGGCAAA TATTATACGC

851 AAGGCGACAA GGTGCTGATG CCGCTGGCGA TTCAGGTTCA TCATGCCGTT

901 TGTGATGGCT TCCATGTCGG CAGAATGCTT AATGAATTAC AACAGTACTG

951 CGATGAGTGG CAGGGCGGGG CGTAAACGCG TGGATCCGGC TTACTAAAAG

1001 CCAGATAACA GTATGCGTAT TTGCGCGCTG ATTTTTGCGG TATAAGAATA

1051 TATACTGATA TGTATACCCG AAGTATGTCA AAAAGAGGTA TGCTATGAAG

1101 CAGCGTATTA CAGTGACAGT TGACAGCGAC AGCTATCAGT TGCTCAAGGC

1151 ATATATGATG TCAATATCTC CGGTCTGGTA AGCACAACCA TGCAGAATGA

1201 AGCCCGTCGT CTGCGTGCCG AACGCTGGAA AGCGGAAAAT CAGGAAGGGA

1251 TGGCTGAGGT CGCCCGGTTT ATTGAAATGA ACGGCTCTTT TGCTGACGAG

1301 AACAGGGGCT GGTGAAATGC AGTTTAAGGT TTACACCTAT AAAAGAGAGA

1351 GCCGTTATCG TCTGTTTGTG GATGTACAGA GTGATATTAT TGACACGCCC

1401 GGGCGACGGA TGGTGATCCC CCTGGCCAGT GCACGTCTGC TGTCAGATAA

1451 AGTCTCCCGT GAACTTTACC CGGTGGTGCA TATCGGGGAT GAAAGCTGGC

1501 GCATGATGAC CACCGATATG GCCAGTGTGC CGGTCTCCGT TATCGGGGAA

1551 GAAGTGGCTG ATCTCAGCCA CCGCGAAAAT GACATCAAAA ACGCCATTAA

1601 CCTGATGTTC TGGGGAATAT AAATGTCAGG CTCCCTTATA CACAGCCAGT

1651 CTGCAGGTCG ACCATAGTGA CTGGATATGT TGTGTTTTAC AGTATTATGT

1701 AGTCTGTTTT TTATGCAAAA TCTAATTTAA TATATTGATA TTTATATCAT

1751 TTTACGTTTC TCGTTCAGCT TTCTTGTACA AAGTGGTGAT AAACGGCCGG

1801 CCGAGCTCGC CCGGGGATCG AGCGCAGCGG TATAAAAGGG CGCGGGGTGG

1851 CTGAGAGCAT CAGTTGTGAA TGAATGTTCG AGCCGAGCAG ACGTGCCGCT

1901 GCCTTCGTTA ATATCCTTTG AATAAGCCAA CTTTGAATCA CAAGACGCAT

1951 ACCAAACGGT ACCGCTAGAA AAAGGTAGGT TCAACCACTG ATGCCTAGGC

2001 ACACCGAAAC GACTAACCCT AATTCTTATC CTTTACTTCA GGGCTAGCAA

2051 CTTAAAAAAA AAAATCAAAA TGATGCTGAA GAAGATCCTG AAGATCGAGG

2101 AGCTGGATGA GCGCGAGCTG ATCGATATCG AGGTGAGCGG CAACCACCTG

2151 TTCTACGCCA ACGATATCCT GACCCACAAC AGCAATGCAG TGAGCCTGGT

2201 AATGCGTCGA ATACGCAAGG AAAACGTGGA CGCCGGCGAG AGGGCCAAAC

2251 AGGCGTTGGC TTTCGAACGC ACCGATTTCG ATCAGGTGCG GTCGTTGATG

2301 GAGAACAGCG ATCGATGCCA GGACATCCGG AACTTGGCGT TTTTGGGCAT

2351 TGCCTATAAC ACCCTCCTTC GCATCGCCGA GATAGCGCGT ATCCGAGTTA

2401 AAGACATTTC CCGTACAGAC GGAGGCCGCA TGCTTATTCA CATCGGACGC

2451 ACGAAAACCC TGGTCAGCAC TGCTGGCGTG GAGAAAGCAC TTTCCCTGGG

2501 TGTGACGAAA TTGGTCGAGC GATGGATCAG TGTGAGTGGC GTCGCCGATG

2551 ACCCCAACAA TTATCTGTTC TGCAGGGTGC GCAAAAATGG AGTGGCAGCC

2601 CCCTGCCTGG TGGGCAGCAG CGAGATCATC ACCCGCAACT ACGGCAAGAC

2651 CACCATCAAG GAGGTGGTGG AGATCTTCGA TAACGATAAG AACATCCAGG

2701 TGCTGGCCTT CAACACCCAC ACCGATAACA TCGAGTGGGC CCCCATCAAG

2751 GCCGCCCAGC TGACCCGCCC CAACGCCGAG CTGGTGGAGC TGGAGATCGA

2801 TACCCTGCAC GGCGTGAAGA CCATCCGCTG CACCCCCGAT CACCCCGTGT

2851 ACACCAAGAA CCGCGGCTAC GTGCGCGCCG ATGAGCTGAC CGATGATGAT

2901 GAGCTGGTGG TGGCCATCTA AACCGGTGTT TAAACAATCA ACCTCTGGAT

2951 TACAAAATTT GTGAAAGATT GACTGGTATT CTTAACTATG TTGCTCCTTT

3001 TACGCTATGT GGATACGCTG CTTTAATGCC TTTGTATCAT GCTATTGCTT

3051 CCCGTATGGC TTTCATTTTC TCCTCCTTGT ATAAATCCTG GTTGCTGTCT

3101 CTTTATGAGG AGTTGTGGCC CGTTGTCAGG CAACGTGGCG TGGTGTGCAC

3151 TGTGTTTGCT GACGCAACCC CCACTGGTTG GGGCATTGCC ACCACCTGTC

3201 AGCTCCTTTC CGGGACTTTC GCTTTCCCCC TCCCTATTGC CACGGCGGAA

3251 CTCATCGCCG CCTGCCTTGC CCGCTGCTGG ACAGGGGCTC GGCTGTTGGG

3301 CACTGACAAT TCCGTGGTGT TGTCGGGGAA ATCATCGTCC TTTCCTTGGC

3351 TGCTCGCCTG TGTTGCCACC TGGATTCTGC GCGGGACGTC CTTCTGCTAC

3401 GTCCCTTCGG CCCTCAATCC AGCGGACCTT CCTTCCCGCG GCCTGCTGCC

3451 GGCTCTGCGG CCTCTTCCGC GTCTTCGCCT TCGCCCTCAG ACGAGTCGGA

3501 TCTCCCTTTG GGCCGCCTCC CCGCCTGAAG CTTATCGATA CCGTCGACTA

3551 AAGCCAAATA GAAAATTATT CAGTTCCTGG CTTAAGTTTT TAAAAGTGAT

3601 ATTATTTATT TGGTTGTAAC CAACCAAAAG AATGTAAATA ACTAATACAT

3651 AATTATGTTA GTTTTAAGTT AGCAACAAAT TGATTTTAGC TATATTAGCT

3701 ACTTGGTTAA TAAATAGAAT ATATTTATTT AAAGATAATT GCGTTTTTAT

3751 TGTCAGGGAG TGAGTTTGCT TAAAAACTCG TTTAGATCCA CTAGTTCTAG

3801 AGCGGCCGCT GGCCACGGGT GCGCATGATC GTGCTCCTGT CGTTGAGGAC

3851 CCGGCTAGGC TGGCGGGGTT GCCTTACTGG TTAGCAGAAT GAATCACCGA

3901 TACGCGAGCG AACGTGAAGC GACTGCTGCT GCAAAACGTC TGCGACCTGA

3951 GCAACAACAT GAATGGTCTT CGGTTTCCGT GTTTCGTAAA GTCTGGAAAC

4001 GCGGAAGTCA GCGCCCTGCA CCATTATGTT CCGGAGGCGC GCCCTAGTTC

4051 CAGTGAAATC CAAGCATTTT CTAAATTAAA TGTATTCTTA TTATTATAGT

4101 TGTTATTTTT GATATATATA AACAACACTA TTATGCCCAC CATTTTTTTG

4151 AGATGCATCT ACACAAGGAA CAAACACTGG ATGTCACTTT CAGTTCAAAT

4201 TGTAACGCTA ATCACTCCGA ACAGGTCACA AAAAATTACC TTAAAAAGTC

4251 ATAATATTAA ATTAGAATAA ATATAGCTGT GAGGGAAATA TATACAAATA

4301 TATTGGAGCA AATAAATTGT ACATACAAAT ATTTATTACT AATTTCTATT

4351 GAGACGAAAT GAACCACTCG GAACCATTTG AGCGAACCGA ATCGCGCGGA

4401 ACTAACGACA GTCGCTCCAA GGTCGTCGAA CAAAAGGTGA ATGTGTTGCG

4451 GAGAGCGGGT GGGAGACAGC GAAAGAGCAA CTACGAAACG TGGTGTGGTG

4501 GAGGTGAATT ATGAAGAGGG CGCGCGATTT GAAAAGTATG TATATAAAAA

4551 ATATATCCCG GTGTTTTATG TAGCGATAAA CGAGTTTTTG ATGTAAGGTA

4601 TGCAGGTGTG TAAGTCTTTT GGTTAGAAGA CAAATCCAAA GTCTACTTGT

4651 GGGGATGTTC GAAGGGGAAA TACTTGTATT CTATAGGTCA TATCTTGTTT

4701 TTATTGGCAC AAATATAATT ACATTAGCTT TTTGAGGGGG CAATAAACAG

4751 TAAACACGAT GGTAATAATG GTAAAAAAAA AAAACAAGCA GTTATTTCGG

4801 ATATATGTCG GCTACTCCTT GCGTCGGGCC CGAAGTCTTA GAGCCAGATA

4851 TGCGAGCACC CGGAAGCTCA CGATGAGAAT GGCCAGACCC ACGTAGTCCA

4901 GCGGCAGATC GGCGGCGGAG AAGTTAAGCG TCTCCAGGAT GACCTTGCCC

4951 GAACTGGGGC ACGTGGTGTT CGACGATGTG CAGCTAATTT CGCCCGGCTC

5001 CACGTCCGCC CATTGGTTAA TCAGCAGACC CTCGTTGGCG TAACGGAACC

5051 ATGAGAGGTA CGACAACCAT TTGAGGTATA CTGGCACCGA GCCCGAGTTC

5101 AAGAAGAAGC CGCCAAAGAG CAGGAATGGT ATGATAACCG GCGGACCCAC

5151 AGACAGCGCC ATCGAGGTCG AGGAGCTGGC GCAGGATATT AGATATCCGA

5201 AGGACGTTGA CACATTGGCC ACCAGAGTGA CCAGCGCCAG GCAGTTGAAG

5251 AAGTGCAGCA CTCCGGCCCG CAGTCCGATC ATCGGATAGG CAATCGCCGT

5301 GAAGACCAGT GGCACTGTGA GAAAAAGCGG CAATTCGGCA ATCGTTTTGC

5351 CCAGAAAGTA TGTGTCACAG CGATAAAGTC GACTTCGGGC CTCCCTCATA

5401 AAAACTGGCA GCTCTGAGGT GAACACCTAA ATCGAATCGA TTCATTAGAA

5451 AGTTAGTAAA TTATTGAAAT GCAAATGTAT TCTAAACATG ACTTACATTT

5501 ATCGTGGCAA AGACGTTTTG AAAGGTCATG TTGGTCAGGA AGAGGAAGAT

5551 GGCTCCGTTG ATATTCATCA CACCCACTTG CGTGAGTTGT TGGCCCAAAA

5601 AGATGAGGCC AATCAAGATG GCAACCATCT GCAAATTAAA ATGTTACTCG

5651 CATCTCATTA ATATTCGCGA GTTAAATGAA ATTTATTTAT CTTCTGCAAA

5701 ACTATAAACT ATACATCTCA TTGAAAAAAA CTAAGAAGGG TGTGGAATCA

5751 GGCAATTCTA TCTAAAATCT AGCGAATTTG TTTCCAAGAA TTGTAAGCGT

5801 TATATCATTT GTTTCCACTG GAACCACTCA CCGTTGTCTG AATAAGTCGC

5851 ACTTTTACGA GGAGTGGTTC CTTGAGCACC GACAGCCAGG ATCGCCACAG

5901 GACCGCCCGG AACTGCATGA ACCAGGTGGC CTTGTAGGTG TACCCATTCT

5951 CCGGCTGCTC CAGTGGCTTC TCCAGATTTT TGGTGGCCAA CAACTGCTCC

6001 ATATCCCGGG CTACTTTGCT AATGGCAAAA TTGTCGCATA TCTTGGCGAT

6051 CCGATCACGG GACTCGATCT CCCGTCCGGG CACAACGGCC AACACCTGTA

6101 CGTAAAAGTC CGCCGGATTG TAGTTGGTAG GACACTGGGC ACCCACGCTG

6151 GATAGGAGTT GAGATGTTAT GTAATACTAG ATACCCTTAA TAAACACATC

6201 GAACTCACTA GGAAAAGAAG TCGACGGCTT CGCTGGGAGT GCCCAAGAAA

6251 GCTACCCTGC CCTCGGCCAT CAGAAGGATC TTGTCAAAGA GCTCAAACAG

6301 CTCGGAAGAC GGCTGATGAA TGGTCAGGAT GACGGTCTTG CCCTTCTGCG

6351 ACAGCTTCTT CAGCACCTGG ACGACGCTGT GGGCGGTAAA GGAGTCCAGT

6401 CCGGAGGTGG GCTCATCGCA GATCAGAAGC GGCGGATCGG TTAGAGCCTC

6451 GGAGGCGAAT GCCAGACGCT TCCTTTCTCC GCCGGACAGA CCTTTCACCC

6501 TGCCGGGCAC ACCGATGATC GTGTGCTGAC ATTTGCTGAG CGAAAGCTCC

6551 TGGATCACCT GATCCACGCG GGCCACTCGC TGCCGATAGG TCAGATGTCG

6601 TGGCATCCGC ACCATGGCTT GGAAAATCAG GTGTTCCCTG GCCGTTAGGG

6651 AGCCGATAAA GAGGTCATCC TGCTGGACAT AGGCGCACCT GGCCTGCATC

6701 TCCTTGGCGT CCACAGGTTG GCCATTGAGC AGTCGCATCC CGGATGGCGA

6751 TACTTGGATG CCCTGCGGCG ATCGAAAGGC AAGGGCATTC AGCAGGGTCG

6801 TCTTTCCGGC ACCGGAACTG CCCATCACGG CCAAAAGTTC GCCCGGATAG

6851 GCCACGCCGC AAACTGAGTT TCAAATTGGT AATTGGACCC TTTATTAAGA

6901 TTTCACACAG ATCAGCCGAC TGCGAATAGA AACTCACCGT TCTTGAGCAA

6951 ATGTTTCCTG GGCGCCGGTA TGTGTCGCTC GTTGCAGAAT AGTCCGCGTG

7001 TCCGGTTGAC CAGCTGCCGC CATCCGGAGC CCGGCTGATT GACCGCCCCA

7051 AAGATGTCCA TATTGTGCCA GGCATAGGTG AGGTTCTCGG CTAGTTGGCC

7101 GCTCCCTGAA CCGGAGTCCT CCGGCGGACT GGGTGGCCGG AGCGTGCCGT

7151 AGTTTTTGGC CTGCCCGAAG CCCTGGTTAA TGCAGCTCTG CGAAGCCGCT

7201 CCGCTGTCAC CCTGCAATGA TAGGGGATCT CAAATATCAA CTACTAGCGT

7251 TATGCTCATC TAACCCCGAA CAAAAAGTAC CCCGAAGTAT CCTACGAAGT

7301 AGGTTTATAC TTTTATTTAT TTTTTGTGCA TCTAGGATCA GCTTAAAATA

7351 TCTGGTTGTT ATATTTTTTG TAAAAAAGAA TATAGTCGAA AATGAATGCC

7401 TTTAGATGTC TTGATCATGA TATGATCTCA AAAATTGTCT TATATAGCGA

7451 GAACAGCTAC CAGAATAATC TGTTTCGTGT CACTATTTGT TTGTGCAATT

7501 GCGGTTTGGG ATTTTTGTGG GTCGCAGTTC TCACGCCGCA TACAATTTGA

7551 TGTTGCAATC GCAGTTCCTA TAGATCAAGT GAACTTAAGA TGTATGCACA

7601 TGTACTACTC ACATTGTTCA GATGCTCGGC AGATGGGTGT TTGCTGCCTC

7651 CGCGAATTAA TAGCTCCTGA TCCTCTTGGC CCATTGCCGG GATTTTTCAC

7701 ACTTTCCCCT GCTTACCCAC CCAAAACCAA TCACCACCCC AATCACTCAA

7751 AAAACAAACA AAAATAAGAA GCGAGAGGAG TTTTGGCACA GCACTTTGTG

7801 TTTAATTGAT GGCGTAAACC GCTTGGAGCT TCGTCACGAA ACCGCTGACA

7851 AAATGCAACT GAAGGCGGAC ATTGACGCTA CGTAACGCTA CAAACGGTGG

7901 CGAAAGAGAT AGCGGACGCA GCGGCGAAAG AGACGGCGAT ATTTCTGTGG

7951 ACAGAGAAGG AGGCAAACAG CGCTGACTTT GAGTGGAATG TCATTTTGAG

8001 TGAGAGGTAA TCGAAAGAAC CTGGTACATC AAATACCCTT GGATCGAAGT

8051 AAATTTAAAA CTGATCAGAT AAGTTCAATG ATATCCAGTG CAGTAAAAAA

8101 AAAAAATGTT TTTTTTATCT ACTTTCCGCA AAAATGGGTT TTATTAACTT

8151 ACATACATAC TAGGCGCGCC CATATGTTCG GCTTGTCGAC ATGCCCGCCG

8201 TGACCGTCGA GAACCCGCTG ACGCTGCCCC GCGTATCCGC ACCCGCCGAC

8251 GCCGTCGCAC GTCCCGTGCT CACCGTGACC ACCGCGCCCA GCGGTTTCGA

8301 GGGCGAGGGC TTCCCGGTGC GCCGCGCGTT CGCCGGGATC AACTACCGCC

8351 ACCTCGACCC GTTCATCATG ATGGACCAGA TGGGTGAGGT GGAGTACGCG

8401 CCCGGGGAGC CCAAGGGCAC GCCCTGGCAC CCGCACCGCG GCTTCGAGAC

8451 CGTGACCTAC ATCGTCGACG GTAACATGTG AGCAAAAGGC CAGCAAAAGG

8501 CCAGGAACCG TAAAAAGGCC GCGTTGCTGG CGTTTTTCCA TAGGCTCCGC

8551 CCCCCTGACG AGCATCACAA AAATCGACGC TCAAGTCAGA GGTGGCGAAA

8601 CCCGACAGGA CTATAAAGAT ACCAGGCGTT TCCCCCTGGA AGCTCCCTCG

8651 TGCGCTCTCC TGTTCCGACC CTGCCGCTTA CCGGATACCT GTCCGCCTTT

8701 CTCCCTTCGG GAAGCGTGGC GCTTTCTCAT AGCTCACGCT GTAGGTATCT

8751 CAGTTCGGTG TAGGTCGTTC GCTCCAAGCT GGGCTGTGTG CACGAACCCC

8801 CCGTTCAGCC CGACCGCTGC GCCTTATCCG GTAACTATCG TCTTGAGTCC

8851 AACCCGGTAA GACACGACTT ATCGCCACTG GCAGCAGCCA CTGGTAACAG

8901 GATTAGCAGA GCGAGGTATG TAGGCGGTGC TACAGAGTTC TTGAAGTGGT

8951 GGCCTAACTA CGGCTACACT AGAAGAACAG TATTTGGTAT CTGCGCTCTG

9001 CTGAAGCCAG TTACCTTCGG AAAAAGAGTT GGTAGCTCTT GATCCGGCAA

9051 ACAAACCACC GCTGGTAGCG GTGGTTTTTT TGTTTGCAAG CAGCAGATTA

9101 CGCGCAGAAA AAAAGGATCT CAAGAAGATC CTTTGATCTT TTCTACGGGG

9151 TCTGACGCTC AGTGGAACGA AAACTCACGT TAAGGGATTT TGGTCATGAG

9201 ATTATCAAAA AGGATCTTCA CCTAGATCCT TTTAAATTAA AAATGAAGTT

9251 TTAAATCAAT CTAAAGTATA TATGAGTAAA CTTGGTCTGA CAGTTACCAA

9301 TGCTTAATCA GTGAGGCACC TATCTCAGCG ATCTGTCTAT TTCGTTCATC

9351 CATAGTTGCC TGACTCCCCG TCGTGTAGAT AACTACGATA CGGGAGGGCT

9401 TACCATCTGG CCCCAGTGCT GCAATGATAC CGCGAGACCC ACGCTCACCG

9451 GCTCCAGATT TATCAGCAAT AAACCAGCCA GCCGGAAGGG CCGAGCGCAG

9501 AAGTGGTCCT GCAACTTTAT CCGCCTCCAT CCAGTCTATT AATTGTTGCC

9551 GGGAAGCTAG AGTAAGTAGT TCGCCAGTTA ATAGTTTGCG CAACGTTGTT

9601 GCCATTGCTA CAGGCATCGT GGTGTCACGC TCGTCGTTTG GTATGGCTTC

9651 ATTCAGCTCC GGTTCCCAAC GATCAAGGCG AGTTACATGA TCCCCCATGT

9701 TGTGCAAAAA AGCGGTTAGC TCCTTCGGTC CTCCGATCGT TGTCAGAAGT

9751 AAGTTGGCCG CAGTGTTATC ACTCATGGTT ATGGCAGCAC TGCATAATTC

9801 TCTTACTGTC ATGCCATCCG TAAGATGCTT TTCTGTGACT GGTGAGTACT

9851 CAACCAAGTC ATTCTGAGAA TAGTGTATGC GGCGACCGAG TTGCTCTTGC

9901 CCGGCGTCAA TACGGGATAA TACCGCGCCA CATAGCAGAA CTTTAAAAGT

9951 GCTCATCATT GGAAAACGTT CTTCGGGGCG AAAACTCTCA AGGATCTTAC

10001 CGCTGTTGAG ATCCAGTTCG ATGTAACCCA CTCGTGCACC CAACTGATCT

10051 TCAGCATCTT TTACTTTCAC CAGCGTTTCT GGGTGAGCAA AAACAGGAAG

10101 GCAAAATGCC GCAAAAAAGG GAATAAGGGC GACACGGAAA TGTTGAATAC

10151 TCATACTCTT CCTTTTTCAA TATTATTGAA GCATTTATCA GGGTTATTGT

10201 CTCATGAGCG GATACATATT TGAATGTATT TAGAAAAATA AACAAATAGG

10251 GGTTCCGCGC ACATTTCCCC GAAAAGTGCC ACCTGAGTC

**HJP-196-IVS-Syn21-NrdJ-1^C^-Cre_C_-WPREUw**

1 TAAGAAACCA TTATTATCAT GACATTAACC TATAAAAATA GGCGTATCAC

51 GAGGCCCTTT CGTCTTCAAG AATTCGTTTA TCACAAGTTT GTACAAAAAA

101 GCTGAACGAG AAACGTAAAA TGATATAAAT ATCAATATAT TAAATTAGAT

151 TTTGCATAAA AAACAGACTA CATAATACTG TAAAACACAA CATATCCAGT

201 CACTATGGCG GCCGCATTAG GCACCCCAGG CTTTACACTT TATGCTTCCG

251 GCTCGTATAA TGTGTGGATT TTGAGTTAGG ATCCGTCGAG ATTTTCAGGA

301 GCTAAGGAAG CTAAAATGGA GAAAAAAATC ACTGGATATA CCACCGTTGA

351 TATATCCCAA TGGCATCGTA AAGAACATTT TGAGGCATTT CAGTCAGTTG

401 CTCAATGTAC CTATAACCAG ACCGTTCAGC TGGATATTAC GGCCTTTTTA

451 AAGACCGTAA AGAAAAATAA GCACAAGTTT TATCCGGCCT TTATTCACAT

501 TCTTGCCCGC CTGATGAATG CTCATCCGGA ATTCCGTATG GCAATGAAAG

551 ACGGTGAGCT GGTGATATGG GATAGTGTTC ACCCTTGTTA CACCGTTTTC

601 CATGAGCAAA CTGAAACGTT TTCATCGCTC TGGAGTGAAT ACCACGACGA

651 TTTCCGGCAG TTTCTACACA TATATTCGCA AGATGTGGCG TGTTACGGTG

701 AAAACCTGGC CTATTTCCCT AAAGGGTTTA TTGAGAATAT GTTTTTCGTC

751 TCAGCCAATC CCTGGGTGAG TTTCACCAGT TTTGATTTAA ACGTGGCCAA

801 TATGGACAAC TTCTTCGCCC CCGTTTTCAC CATGGGCAAA TATTATACGC

851 AAGGCGACAA GGTGCTGATG CCGCTGGCGA TTCAGGTTCA TCATGCCGTT

901 TGTGATGGCT TCCATGTCGG CAGAATGCTT AATGAATTAC AACAGTACTG

951 CGATGAGTGG CAGGGCGGGG CGTAAACGCG TGGATCCGGC TTACTAAAAG

1001 CCAGATAACA GTATGCGTAT TTGCGCGCTG ATTTTTGCGG TATAAGAATA

1051 TATACTGATA TGTATACCCG AAGTATGTCA AAAAGAGGTA TGCTATGAAG

1101 CAGCGTATTA CAGTGACAGT TGACAGCGAC AGCTATCAGT TGCTCAAGGC

1151 ATATATGATG TCAATATCTC CGGTCTGGTA AGCACAACCA TGCAGAATGA

1201 AGCCCGTCGT CTGCGTGCCG AACGCTGGAA AGCGGAAAAT CAGGAAGGGA

1251 TGGCTGAGGT CGCCCGGTTT ATTGAAATGA ACGGCTCTTT TGCTGACGAG

1301 AACAGGGGCT GGTGAAATGC AGTTTAAGGT TTACACCTAT AAAAGAGAGA

1351 GCCGTTATCG TCTGTTTGTG GATGTACAGA GTGATATTAT TGACACGCCC

1401 GGGCGACGGA TGGTGATCCC CCTGGCCAGT GCACGTCTGC TGTCAGATAA

1451 AGTCTCCCGT GAACTTTACC CGGTGGTGCA TATCGGGGAT GAAAGCTGGC

1501 GCATGATGAC CACCGATATG GCCAGTGTGC CGGTCTCCGT TATCGGGGAA

1551 GAAGTGGCTG ATCTCAGCCA CCGCGAAAAT GACATCAAAA ACGCCATTAA

1601 CCTGATGTTC TGGGGAATAT AAATGTCAGG CTCCCTTATA CACAGCCAGT

1651 CTGCAGGTCG ACCATAGTGA CTGGATATGT TGTGTTTTAC AGTATTATGT

1701 AGTCTGTTTT TTATGCAAAA TCTAATTTAA TATATTGATA TTTATATCAT

1751 TTTACGTTTC TCGTTCAGCT TTCTTGTACA AAGTGGTGAT AAACGGCCGG

1801 CCGAGCTCGC CCGGGGATCG AGCGCAGCGG TATAAAAGGG CGCGGGGTGG

1851 CTGAGAGCAT CAGTTGTGAA TGAATGTTCG AGCCGAGCAG ACGTGCCGCT

1901 GCCTTCGTTA ATATCCTTTG AATAAGCCAA CTTTGAATCA CAAGACGCAT

1951 ACCAAACGGT ACCGCTAGAA AAAGGTAGGT TCAACCACTG ATGCCTAGGC

2001 ACACCGAAAC GACTAACCCT AATTCTTATC CTTTACTTCA GGGCTAGCAA

2051 CTTAAAAAAA AAAATCAAAA TGGAGGCCAA GACCTACATC GGCAAGCTGA

2101 AGAGCCGCAA GATCGTGAGC AACGAGGATA CCTACGATAT CCAGACCAGC

2151 ACCCACAACT TCTTCGCCAA CGATATCCTG GTGCACAACT CGGCAACAAG

2201 TCAGTTGAGT ACGCGCGCGT TGGAGGGAAT CTTCGAGGCC ACTCACCGAT

2251 TGATTTACGG TGCAAAAGAT GACTCCGGCC AACGTTACTT GGCCTGGTCC

2301 GGTCACAGCG CACGTGTGGG TGCCGCCCGT GACATGGCGA GGGCCGGTGT

2351 TTCCATCCCA GAAATCATGC AAGCGGGCGG ATGGACCAAC GTGAATATTG

2401 TAATGAACTA CATTCGTAAT CTGGATAGTG AAACAGGCGC AATGGTGCGC

2451 CTGCTGGAGG ACGGAGATTA AACCGGTGTT TAAACAATCA ACCTCTGGAT

2501 TACAAAATTT GTGAAAGATT GACTGGTATT CTTAACTATG TTGCTCCTTT

2551 TACGCTATGT GGATACGCTG CTTTAATGCC TTTGTATCAT GCTATTGCTT

2601 CCCGTATGGC TTTCATTTTC TCCTCCTTGT ATAAATCCTG GTTGCTGTCT

2651 CTTTATGAGG AGTTGTGGCC CGTTGTCAGG CAACGTGGCG TGGTGTGCAC

2701 TGTGTTTGCT GACGCAACCC CCACTGGTTG GGGCATTGCC ACCACCTGTC

2751 AGCTCCTTTC CGGGACTTTC GCTTTCCCCC TCCCTATTGC CACGGCGGAA

2801 CTCATCGCCG CCTGCCTTGC CCGCTGCTGG ACAGGGGCTC GGCTGTTGGG

2851 CACTGACAAT TCCGTGGTGT TGTCGGGGAA ATCATCGTCC TTTCCTTGGC

2901 TGCTCGCCTG TGTTGCCACC TGGATTCTGC GCGGGACGTC CTTCTGCTAC

2951 GTCCCTTCGG CCCTCAATCC AGCGGACCTT CCTTCCCGCG GCCTGCTGCC

3001 GGCTCTGCGG CCTCTTCCGC GTCTTCGCCT TCGCCCTCAG ACGAGTCGGA

3051 TCTCCCTTTG GGCCGCCTCC CCGCCTGAAG CTTATCGATA CCGTCGACTA

3101 AAGCCAAATA GAAAATTATT CAGTTCCTGG CTTAAGTTTT TAAAAGTGAT

3151 ATTATTTATT TGGTTGTAAC CAACCAAAAG AATGTAAATA ACTAATACAT

3201 AATTATGTTA GTTTTAAGTT AGCAACAAAT TGATTTTAGC TATATTAGCT

3251 ACTTGGTTAA TAAATAGAAT ATATTTATTT AAAGATAATT GCGTTTTTAT

3301 TGTCAGGGAG TGAGTTTGCT TAAAAACTCG TTTAGATCCA CTAGTTCTAG

3351 AGCGGCCGCT GGCCACGGGT GCGCATGATC GTGCTCCTGT CGTTGAGGAC

3401 CCGGCTAGGC TGGCGGGGTT GCCTTACTGG TTAGCAGAAT GAATCACCGA

3451 TACGCGAGCG AACGTGAAGC GACTGCTGCT GCAAAACGTC TGCGACCTGA

3501 GCAACAACAT GAATGGTCTT CGGTTTCCGT GTTTCGTAAA GTCTGGAAAC

3551 GCGGAAGTCA GCGCCCTGCA CCATTATGTT CCGGAGGCGC GCCCTAGTTC

3601 CAGTGAAATC CAAGCATTTT CTAAATTAAA TGTATTCTTA TTATTATAGT

3651 TGTTATTTTT GATATATATA AACAACACTA TTATGCCCAC CATTTTTTTG

3701 AGATGCATCT ACACAAGGAA CAAACACTGG ATGTCACTTT CAGTTCAAAT

3751 TGTAACGCTA ATCACTCCGA ACAGGTCACA AAAAATTACC TTAAAAAGTC

3801 ATAATATTAA ATTAGAATAA ATATAGCTGT GAGGGAAATA TATACAAATA

3851 TATTGGAGCA AATAAATTGT ACATACAAAT ATTTATTACT AATTTCTATT

3901 GAGACGAAAT GAACCACTCG GAACCATTTG AGCGAACCGA ATCGCGCGGA

3951 ACTAACGACA GTCGCTCCAA GGTCGTCGAA CAAAAGGTGA ATGTGTTGCG

4001 GAGAGCGGGT GGGAGACAGC GAAAGAGCAA CTACGAAACG TGGTGTGGTG

4051 GAGGTGAATT ATGAAGAGGG CGCGCGATTT GAAAAGTATG TATATAAAAA

4101 ATATATCCCG GTGTTTTATG TAGCGATAAA CGAGTTTTTG ATGTAAGGTA

4151 TGCAGGTGTG TAAGTCTTTT GGTTAGAAGA CAAATCCAAA GTCTACTTGT

4201 GGGGATGTTC GAAGGGGAAA TACTTGTATT CTATAGGTCA TATCTTGTTT

4251 TTATTGGCAC AAATATAATT ACATTAGCTT TTTGAGGGGG CAATAAACAG

4301 TAAACACGAT GGTAATAATG GTAAAAAAAA AAAACAAGCA GTTATTTCGG

4351 ATATATGTCG GCTACTCCTT GCGTCGGGCC CGAAGTCTTA GAGCCAGATA

4401 TGCGAGCACC CGGAAGCTCA CGATGAGAAT GGCCAGACCC ACGTAGTCCA

4451 GCGGCAGATC GGCGGCGGAG AAGTTAAGCG TCTCCAGGAT GACCTTGCCC

4501 GAACTGGGGC ACGTGGTGTT CGACGATGTG CAGCTAATTT CGCCCGGCTC

4551 CACGTCCGCC CATTGGTTAA TCAGCAGACC CTCGTTGGCG TAACGGAACC

4601 ATGAGAGGTA CGACAACCAT TTGAGGTATA CTGGCACCGA GCCCGAGTTC

4651 AAGAAGAAGC CGCCAAAGAG CAGGAATGGT ATGATAACCG GCGGACCCAC

4701 AGACAGCGCC ATCGAGGTCG AGGAGCTGGC GCAGGATATT AGATATCCGA

4751 AGGACGTTGA CACATTGGCC ACCAGAGTGA CCAGCGCCAG GCAGTTGAAG

4801 AAGTGCAGCA CTCCGGCCCG CAGTCCGATC ATCGGATAGG CAATCGCCGT

4851 GAAGACCAGT GGCACTGTGA GAAAAAGCGG CAATTCGGCA ATCGTTTTGC

4901 CCAGAAAGTA TGTGTCACAG CGATAAAGTC GACTTCGGGC CTCCCTCATA

4951 AAAACTGGCA GCTCTGAGGT GAACACCTAA ATCGAATCGA TTCATTAGAA

5001 AGTTAGTAAA TTATTGAAAT GCAAATGTAT TCTAAACATG ACTTACATTT

5051 ATCGTGGCAA AGACGTTTTG AAAGGTCATG TTGGTCAGGA AGAGGAAGAT

5101 GGCTCCGTTG ATATTCATCA CACCCACTTG CGTGAGTTGT TGGCCCAAAA

5151 AGATGAGGCC AATCAAGATG GCAACCATCT GCAAATTAAA ATGTTACTCG

5201 CATCTCATTA ATATTCGCGA GTTAAATGAA ATTTATTTAT CTTCTGCAAA

5251 ACTATAAACT ATACATCTCA TTGAAAAAAA CTAAGAAGGG TGTGGAATCA

5301 GGCAATTCTA TCTAAAATCT AGCGAATTTG TTTCCAAGAA TTGTAAGCGT

5351 TATATCATTT GTTTCCACTG GAACCACTCA CCGTTGTCTG AATAAGTCGC

5401 ACTTTTACGA GGAGTGGTTC CTTGAGCACC GACAGCCAGG ATCGCCACAG

5451 GACCGCCCGG AACTGCATGA ACCAGGTGGC CTTGTAGGTG TACCCATTCT

5501 CCGGCTGCTC CAGTGGCTTC TCCAGATTTT TGGTGGCCAA CAACTGCTCC

5551 ATATCCCGGG CTACTTTGCT AATGGCAAAA TTGTCGCATA TCTTGGCGAT

5601 CCGATCACGG GACTCGATCT CCCGTCCGGG CACAACGGCC AACACCTGTA

5651 CGTAAAAGTC CGCCGGATTG TAGTTGGTAG GACACTGGGC ACCCACGCTG

5701 GATAGGAGTT GAGATGTTAT GTAATACTAG ATACCCTTAA TAAACACATC

5751 GAACTCACTA GGAAAAGAAG TCGACGGCTT CGCTGGGAGT GCCCAAGAAA

5801 GCTACCCTGC CCTCGGCCAT CAGAAGGATC TTGTCAAAGA GCTCAAACAG

5851 CTCGGAAGAC GGCTGATGAA TGGTCAGGAT GACGGTCTTG CCCTTCTGCG

5901 ACAGCTTCTT CAGCACCTGG ACGACGCTGT GGGCGGTAAA GGAGTCCAGT

5951 CCGGAGGTGG GCTCATCGCA GATCAGAAGC GGCGGATCGG TTAGAGCCTC

6001 GGAGGCGAAT GCCAGACGCT TCCTTTCTCC GCCGGACAGA CCTTTCACCC

6051 TGCCGGGCAC ACCGATGATC GTGTGCTGAC ATTTGCTGAG CGAAAGCTCC

6101 TGGATCACCT GATCCACGCG GGCCACTCGC TGCCGATAGG TCAGATGTCG

6151 TGGCATCCGC ACCATGGCTT GGAAAATCAG GTGTTCCCTG GCCGTTAGGG

6201 AGCCGATAAA GAGGTCATCC TGCTGGACAT AGGCGCACCT GGCCTGCATC

6251 TCCTTGGCGT CCACAGGTTG GCCATTGAGC AGTCGCATCC CGGATGGCGA

6301 TACTTGGATG CCCTGCGGCG ATCGAAAGGC AAGGGCATTC AGCAGGGTCG

6351 TCTTTCCGGC ACCGGAACTG CCCATCACGG CCAAAAGTTC GCCCGGATAG

6401 GCCACGCCGC AAACTGAGTT TCAAATTGGT AATTGGACCC TTTATTAAGA

6451 TTTCACACAG ATCAGCCGAC TGCGAATAGA AACTCACCGT TCTTGAGCAA

6501 ATGTTTCCTG GGCGCCGGTA TGTGTCGCTC GTTGCAGAAT AGTCCGCGTG

6551 TCCGGTTGAC CAGCTGCCGC CATCCGGAGC CCGGCTGATT GACCGCCCCA

6601 AAGATGTCCA TATTGTGCCA GGCATAGGTG AGGTTCTCGG CTAGTTGGCC

6651 GCTCCCTGAA CCGGAGTCCT CCGGCGGACT GGGTGGCCGG AGCGTGCCGT

6701 AGTTTTTGGC CTGCCCGAAG CCCTGGTTAA TGCAGCTCTG CGAAGCCGCT

6751 CCGCTGTCAC CCTGCAATGA TAGGGGATCT CAAATATCAA CTACTAGCGT

6801 TATGCTCATC TAACCCCGAA CAAAAAGTAC CCCGAAGTAT CCTACGAAGT

6851 AGGTTTATAC TTTTATTTAT TTTTTGTGCA TCTAGGATCA GCTTAAAATA

6901 TCTGGTTGTT ATATTTTTTG TAAAAAAGAA TATAGTCGAA AATGAATGCC

6951 TTTAGATGTC TTGATCATGA TATGATCTCA AAAATTGTCT TATATAGCGA

7001 GAACAGCTAC CAGAATAATC TGTTTCGTGT CACTATTTGT TTGTGCAATT

7051 GCGGTTTGGG ATTTTTGTGG GTCGCAGTTC TCACGCCGCA TACAATTTGA

7101 TGTTGCAATC GCAGTTCCTA TAGATCAAGT GAACTTAAGA TGTATGCACA

7151 TGTACTACTC ACATTGTTCA GATGCTCGGC AGATGGGTGT TTGCTGCCTC

7201 CGCGAATTAA TAGCTCCTGA TCCTCTTGGC CCATTGCCGG GATTTTTCAC

7251 ACTTTCCCCT GCTTACCCAC CCAAAACCAA TCACCACCCC AATCACTCAA

7301 AAAACAAACA AAAATAAGAA GCGAGAGGAG TTTTGGCACA GCACTTTGTG

7351 TTTAATTGAT GGCGTAAACC GCTTGGAGCT TCGTCACGAA ACCGCTGACA

7401 AAATGCAACT GAAGGCGGAC ATTGACGCTA CGTAACGCTA CAAACGGTGG

7451 CGAAAGAGAT AGCGGACGCA GCGGCGAAAG AGACGGCGAT ATTTCTGTGG

7501 ACAGAGAAGG AGGCAAACAG CGCTGACTTT GAGTGGAATG TCATTTTGAG

7551 TGAGAGGTAA TCGAAAGAAC CTGGTACATC AAATACCCTT GGATCGAAGT

7601 AAATTTAAAA CTGATCAGAT AAGTTCAATG ATATCCAGTG CAGTAAAAAA

7651 AAAAAATGTT TTTTTTATCT ACTTTCCGCA AAAATGGGTT TTATTAACTT

7701 ACATACATAC TAGGCGCGCC CATATGTTCG GCTTGTCGAC ATGCCCGCCG

7751 TGACCGTCGA GAACCCGCTG ACGCTGCCCC GCGTATCCGC ACCCGCCGAC

7801 GCCGTCGCAC GTCCCGTGCT CACCGTGACC ACCGCGCCCA GCGGTTTCGA

7851 GGGCGAGGGC TTCCCGGTGC GCCGCGCGTT CGCCGGGATC AACTACCGCC

7901 ACCTCGACCC GTTCATCATG ATGGACCAGA TGGGTGAGGT GGAGTACGCG

7951 CCCGGGGAGC CCAAGGGCAC GCCCTGGCAC CCGCACCGCG GCTTCGAGAC

8001 CGTGACCTAC ATCGTCGACG GTAACATGTG AGCAAAAGGC CAGCAAAAGG

8051 CCAGGAACCG TAAAAAGGCC GCGTTGCTGG CGTTTTTCCA TAGGCTCCGC

8101 CCCCCTGACG AGCATCACAA AAATCGACGC TCAAGTCAGA GGTGGCGAAA

8151 CCCGACAGGA CTATAAAGAT ACCAGGCGTT TCCCCCTGGA AGCTCCCTCG

8201 TGCGCTCTCC TGTTCCGACC CTGCCGCTTA CCGGATACCT GTCCGCCTTT

8251 CTCCCTTCGG GAAGCGTGGC GCTTTCTCAT AGCTCACGCT GTAGGTATCT

8301 CAGTTCGGTG TAGGTCGTTC GCTCCAAGCT GGGCTGTGTG CACGAACCCC

8351 CCGTTCAGCC CGACCGCTGC GCCTTATCCG GTAACTATCG TCTTGAGTCC

8401 AACCCGGTAA GACACGACTT ATCGCCACTG GCAGCAGCCA CTGGTAACAG

8451 GATTAGCAGA GCGAGGTATG TAGGCGGTGC TACAGAGTTC TTGAAGTGGT

8501 GGCCTAACTA CGGCTACACT AGAAGAACAG TATTTGGTAT CTGCGCTCTG

8551 CTGAAGCCAG TTACCTTCGG AAAAAGAGTT GGTAGCTCTT GATCCGGCAA

8601 ACAAACCACC GCTGGTAGCG GTGGTTTTTT TGTTTGCAAG CAGCAGATTA

8651 CGCGCAGAAA AAAAGGATCT CAAGAAGATC CTTTGATCTT TTCTACGGGG

8701 TCTGACGCTC AGTGGAACGA AAACTCACGT TAAGGGATTT TGGTCATGAG

8751 ATTATCAAAA AGGATCTTCA CCTAGATCCT TTTAAATTAA AAATGAAGTT

8801 TTAAATCAAT CTAAAGTATA TATGAGTAAA CTTGGTCTGA CAGTTACCAA

8851 TGCTTAATCA GTGAGGCACC TATCTCAGCG ATCTGTCTAT TTCGTTCATC

8901 CATAGTTGCC TGACTCCCCG TCGTGTAGAT AACTACGATA CGGGAGGGCT

8951 TACCATCTGG CCCCAGTGCT GCAATGATAC CGCGAGACCC ACGCTCACCG

9001 GCTCCAGATT TATCAGCAAT AAACCAGCCA GCCGGAAGGG CCGAGCGCAG

9051 AAGTGGTCCT GCAACTTTAT CCGCCTCCAT CCAGTCTATT AATTGTTGCC

9101 GGGAAGCTAG AGTAAGTAGT TCGCCAGTTA ATAGTTTGCG CAACGTTGTT

9151 GCCATTGCTA CAGGCATCGT GGTGTCACGC TCGTCGTTTG GTATGGCTTC

9201 ATTCAGCTCC GGTTCCCAAC GATCAAGGCG AGTTACATGA TCCCCCATGT

9251 TGTGCAAAAA AGCGGTTAGC TCCTTCGGTC CTCCGATCGT TGTCAGAAGT

9301 AAGTTGGCCG CAGTGTTATC ACTCATGGTT ATGGCAGCAC TGCATAATTC

9351 TCTTACTGTC ATGCCATCCG TAAGATGCTT TTCTGTGACT GGTGAGTACT

9401 CAACCAAGTC ATTCTGAGAA TAGTGTATGC GGCGACCGAG TTGCTCTTGC

9451 CCGGCGTCAA TACGGGATAA TACCGCGCCA CATAGCAGAA CTTTAAAAGT

9501 GCTCATCATT GGAAAACGTT CTTCGGGGCG AAAACTCTCA AGGATCTTAC

9551 CGCTGTTGAG ATCCAGTTCG ATGTAACCCA CTCGTGCACC CAACTGATCT

9601 TCAGCATCTT TTACTTTCAC CAGCGTTTCT GGGTGAGCAA AAACAGGAAG

9651 GCAAAATGCC GCAAAAAAGG GAATAAGGGC GACACGGAAA TGTTGAATAC

9701 TCATACTCTT CCTTTTTCAA TATTATTGAA GCATTTATCA GGGTTATTGT

9751 CTCATGAGCG GATACATATT TGAATGTATT TAGAAAAATA AACAAATAGG

9801 GGTTCCGCGC ACATTTCCCC GAAAAGTGCC ACCTGAGTC

**HJP-208-IVS-Syn21-Cre_AB_-NrdJ-1^N^-WPREUw**

1 TAAGAAACCA TTATTATCAT GACATTAACC TATAAAAATA GGCGTATCAC

51 GAGGCCCTTT CGTCTTCAAG AATTCGTTTA TCACAAGTTT GTACAAAAAA

101 GCTGAACGAG AAACGTAAAA TGATATAAAT ATCAATATAT TAAATTAGAT

151 TTTGCATAAA AAACAGACTA CATAATACTG TAAAACACAA CATATCCAGT

201 CACTATGGCG GCCGCATTAG GCACCCCAGG CTTTACACTT TATGCTTCCG

251 GCTCGTATAA TGTGTGGATT TTGAGTTAGG ATCCGTCGAG ATTTTCAGGA

301 GCTAAGGAAG CTAAAATGGA GAAAAAAATC ACTGGATATA CCACCGTTGA

351 TATATCCCAA TGGCATCGTA AAGAACATTT TGAGGCATTT CAGTCAGTTG

401 CTCAATGTAC CTATAACCAG ACCGTTCAGC TGGATATTAC GGCCTTTTTA

451 AAGACCGTAA AGAAAAATAA GCACAAGTTT TATCCGGCCT TTATTCACAT

501 TCTTGCCCGC CTGATGAATG CTCATCCGGA ATTCCGTATG GCAATGAAAG

551 ACGGTGAGCT GGTGATATGG GATAGTGTTC ACCCTTGTTA CACCGTTTTC

601 CATGAGCAAA CTGAAACGTT TTCATCGCTC TGGAGTGAAT ACCACGACGA

651 TTTCCGGCAG TTTCTACACA TATATTCGCA AGATGTGGCG TGTTACGGTG

701 AAAACCTGGC CTATTTCCCT AAAGGGTTTA TTGAGAATAT GTTTTTCGTC

751 TCAGCCAATC CCTGGGTGAG TTTCACCAGT TTTGATTTAA ACGTGGCCAA

801 TATGGACAAC TTCTTCGCCC CCGTTTTCAC CATGGGCAAA TATTATACGC

851 AAGGCGACAA GGTGCTGATG CCGCTGGCGA TTCAGGTTCA TCATGCCGTT

901 TGTGATGGCT TCCATGTCGG CAGAATGCTT AATGAATTAC AACAGTACTG

951 CGATGAGTGG CAGGGCGGGG CGTAAACGCG TGGATCCGGC TTACTAAAAG

1001 CCAGATAACA GTATGCGTAT TTGCGCGCTG ATTTTTGCGG TATAAGAATA

1051 TATACTGATA TGTATACCCG AAGTATGTCA AAAAGAGGTA TGCTATGAAG

1101 CAGCGTATTA CAGTGACAGT TGACAGCGAC AGCTATCAGT TGCTCAAGGC

1151 ATATATGATG TCAATATCTC CGGTCTGGTA AGCACAACCA TGCAGAATGA

1201 AGCCCGTCGT CTGCGTGCCG AACGCTGGAA AGCGGAAAAT CAGGAAGGGA

1251 TGGCTGAGGT CGCCCGGTTT ATTGAAATGA ACGGCTCTTT TGCTGACGAG

1301 AACAGGGGCT GGTGAAATGC AGTTTAAGGT TTACACCTAT AAAAGAGAGA

1351 GCCGTTATCG TCTGTTTGTG GATGTACAGA GTGATATTAT TGACACGCCC

1401 GGGCGACGGA TGGTGATCCC CCTGGCCAGT GCACGTCTGC TGTCAGATAA

1451 AGTCTCCCGT GAACTTTACC CGGTGGTGCA TATCGGGGAT GAAAGCTGGC

1501 GCATGATGAC CACCGATATG GCCAGTGTGC CGGTCTCCGT TATCGGGGAA

1551 GAAGTGGCTG ATCTCAGCCA CCGCGAAAAT GACATCAAAA ACGCCATTAA

1601 CCTGATGTTC TGGGGAATAT AAATGTCAGG CTCCCTTATA CACAGCCAGT

1651 CTGCAGGTCG ACCATAGTGA CTGGATATGT TGTGTTTTAC AGTATTATGT

1701 AGTCTGTTTT TTATGCAAAA TCTAATTTAA TATATTGATA TTTATATCAT

1751 TTTACGTTTC TCGTTCAGCT TTCTTGTACA AAGTGGTGAT AAACGGCCGG

1801 CCGAGCTCGC CCGGGGATCG AGCGCAGCGG TATAAAAGGG CGCGGGGTGG

1851 CTGAGAGCAT CAGTTGTGAA TGAATGTTCG AGCCGAGCAG ACGTGCCGCT

1901 GCCTTCGTTA ATATCCTTTG AATAAGCCAA CTTTGAATCA CAAGACGCAT

1951 ACCAAACGGT ACCGCTAGAA AAAGGTAGGT TCAACCACTG ATGCCTAGGC

2001 ACACCGAAAC GACTAACCCT AATTCTTATC CTTTACTTCA GGGCTAGCAA

2051 CTTAAAAAAA AAAATCAAAA TGAGTAACTT GCTCACGGTA CACCAAAATT

2101 TGCCGGCCCT GCCTGTGGAC GCCACGTCCG ACGAGGTCCG AAAGAATCTG

2151 ATGGACATGT TCCGTGACAG GCAGGCCTTT AGCGAGCACA CCTGGAAGAT

2201 GCTGCTCTCG GTGTGCAGGT CCTGGGCCGC TTGGTGCAAG CTGAACAACC

2251 GAAAGTGGTT TCCCGCGGAG CCCGAGGACG TCCGCGATTA TCTGCTGTAC

2301 CTGCAGGCTC GCGGACTGGC CGTCAAAACA ATACAGCAAC ATTTGGGTCA

2351 GCTGAACATG CTCCACAGGC GCAGCGGACT GCCCCGGCCA TCCGATAGCA

2401 ATGCAGTGAG CCTGGTAATG CGTCGAATAC GCAAGGAAAA CGTGGACGCC

2451 GGCGAGAGGG CCAAACAGGC GTTGGCTTTC GAACGCACCG ATTTCGATCA

2501 GGTGCGGTCG TTGATGGAGA ACAGCGATCG ATGCCAGGAC ATCCGGAACT

2551 TGGCGTTTTT GGGCATTGCC TATAACACCC TCCTTCGCAT CGCCGAGATA

2601 GCGCGTATCC GAGTTAAAGA CATTTCCCGT ACAGACGGAG GCCGCATGCT

2651 TATTCACATC GGACGCACGA AAACCCTGGT CAGCACTGCT GGCGTGGAGA

2701 AAGCACTTTC CCTGGGTGTG ACGAAATTGG TCGAGCGATG GATCAGTGTG

2751 AGTGGCGTCG CCGATGACCC CAACAATTAT CTGTTCTGCA GGGTGCGCAA

2801 AAATGGAGTG GCAGCCCCCT GCCTGGTGGG CAGCAGCGAG ATCATCACCC

2851 GCAACTACGG CAAGACCACC ATCAAGGAGG TGGTGGAGAT CTTCGATAAC

2901 GATAAGAACA TCCAGGTGCT GGCCTTCAAC ACCCACACCG ATAACATCGA

2951 GTGGGCCCCC ATCAAGGCCG CCCAGCTGAC CCGCCCCAAC GCCGAGCTGG

3001 TGGAGCTGGA GATCGATACC CTGCACGGCG TGAAGACCAT CCGCTGCACC

3051 CCCGATCACC CCGTGTACAC CAAGAACCGC GGCTACGTGC GCGCCGATGA

3101 GCTGACCGAT GATGATGAGC TGGTGGTGGC CATCTAAACC GGTGTTTAAA

3151 CAATCAACCT CTGGATTACA AAATTTGTGA AAGATTGACT GGTATTCTTA

3201 ACTATGTTGC TCCTTTTACG CTATGTGGAT ACGCTGCTTT AATGCCTTTG

3251 TATCATGCTA TTGCTTCCCG TATGGCTTTC ATTTTCTCCT CCTTGTATAA

3301 ATCCTGGTTG CTGTCTCTTT ATGAGGAGTT GTGGCCCGTT GTCAGGCAAC

3351 GTGGCGTGGT GTGCACTGTG TTTGCTGACG CAACCCCCAC TGGTTGGGGC

3401 ATTGCCACCA CCTGTCAGCT CCTTTCCGGG ACTTTCGCTT TCCCCCTCCC

3451 TATTGCCACG GCGGAACTCA TCGCCGCCTG CCTTGCCCGC TGCTGGACAG

3501 GGGCTCGGCT GTTGGGCACT GACAATTCCG TGGTGTTGTC GGGGAAATCA

3551 TCGTCCTTTC CTTGGCTGCT CGCCTGTGTT GCCACCTGGA TTCTGCGCGG

3601 GACGTCCTTC TGCTACGTCC CTTCGGCCCT CAATCCAGCG GACCTTCCTT

3651 CCCGCGGCCT GCTGCCGGCT CTGCGGCCTC TTCCGCGTCT TCGCCTTCGC

3701 CCTCAGACGA GTCGGATCTC CCTTTGGGCC GCCTCCCCGC CTGAAGCTTA

3751 TCGATACCGT CGACTAAAGC CAAATAGAAA ATTATTCAGT TCCTGGCTTA

3801 AGTTTTTAAA AGTGATATTA TTTATTTGGT TGTAACCAAC CAAAAGAATG

3851 TAAATAACTA ATACATAATT ATGTTAGTTT TAAGTTAGCA ACAAATTGAT

3901 TTTAGCTATA TTAGCTACTT GGTTAATAAA TAGAATATAT TTATTTAAAG

3951 ATAATTGCGT TTTTATTGTC AGGGAGTGAG TTTGCTTAAA AACTCGTTTA

4001 GATCCACTAG TTCTAGAGCG GCCGCTGGCC ACGGGTGCGC ATGATCGTGC

4051 TCCTGTCGTT GAGGACCCGG CTAGGCTGGC GGGGTTGCCT TACTGGTTAG

4101 CAGAATGAAT CACCGATACG CGAGCGAACG TGAAGCGACT GCTGCTGCAA

4151 AACGTCTGCG ACCTGAGCAA CAACATGAAT GGTCTTCGGT TTCCGTGTTT

4201 CGTAAAGTCT GGAAACGCGG AAGTCAGCGC CCTGCACCAT TATGTTCCGG

4251 AGGCGCGCCC TAGTTCCAGT GAAATCCAAG CATTTTCTAA ATTAAATGTA

4301 TTCTTATTAT TATAGTTGTT ATTTTTGATA TATATAAACA ACACTATTAT

4351 GCCCACCATT TTTTTGAGAT GCATCTACAC AAGGAACAAA CACTGGATGT

4401 CACTTTCAGT TCAAATTGTA ACGCTAATCA CTCCGAACAG GTCACAAAAA

4451 ATTACCTTAA AAAGTCATAA TATTAAATTA GAATAAATAT AGCTGTGAGG

4501 GAAATATATA CAAATATATT GGAGCAAATA AATTGTACAT ACAAATATTT

4551 ATTACTAATT TCTATTGAGA CGAAATGAAC CACTCGGAAC CATTTGAGCG

4601 AACCGAATCG CGCGGAACTA ACGACAGTCG CTCCAAGGTC GTCGAACAAA

4651 AGGTGAATGT GTTGCGGAGA GCGGGTGGGA GACAGCGAAA GAGCAACTAC

4701 GAAACGTGGT GTGGTGGAGG TGAATTATGA AGAGGGCGCG CGATTTGAAA

4751 AGTATGTATA TAAAAAATAT ATCCCGGTGT TTTATGTAGC GATAAACGAG

4801 TTTTTGATGT AAGGTATGCA GGTGTGTAAG TCTTTTGGTT AGAAGACAAA

4851 TCCAAAGTCT ACTTGTGGGG ATGTTCGAAG GGGAAATACT TGTATTCTAT

4901 AGGTCATATC TTGTTTTTAT TGGCACAAAT ATAATTACAT TAGCTTTTTG

4951 AGGGGGCAAT AAACAGTAAA CACGATGGTA ATAATGGTAA AAAAAAAAAA

5001 CAAGCAGTTA TTTCGGATAT ATGTCGGCTA CTCCTTGCGT CGGGCCCGAA

5051 GTCTTAGAGC CAGATATGCG AGCACCCGGA AGCTCACGAT GAGAATGGCC

5101 AGACCCACGT AGTCCAGCGG CAGATCGGCG GCGGAGAAGT TAAGCGTCTC

5151 CAGGATGACC TTGCCCGAAC TGGGGCACGT GGTGTTCGAC GATGTGCAGC

5201 TAATTTCGCC CGGCTCCACG TCCGCCCATT GGTTAATCAG CAGACCCTCG

5251 TTGGCGTAAC GGAACCATGA GAGGTACGAC AACCATTTGA GGTATACTGG

5301 CACCGAGCCC GAGTTCAAGA AGAAGCCGCC AAAGAGCAGG AATGGTATGA

5351 TAACCGGCGG ACCCACAGAC AGCGCCATCG AGGTCGAGGA GCTGGCGCAG

5401 GATATTAGAT ATCCGAAGGA CGTTGACACA TTGGCCACCA GAGTGACCAG

5451 CGCCAGGCAG TTGAAGAAGT GCAGCACTCC GGCCCGCAGT CCGATCATCG

5501 GATAGGCAAT CGCCGTGAAG ACCAGTGGCA CTGTGAGAAA AAGCGGCAAT

5551 TCGGCAATCG TTTTGCCCAG AAAGTATGTG TCACAGCGAT AAAGTCGACT

5601 TCGGGCCTCC CTCATAAAAA CTGGCAGCTC TGAGGTGAAC ACCTAAATCG

5651 AATCGATTCA TTAGAAAGTT AGTAAATTAT TGAAATGCAA ATGTATTCTA

5701 AACATGACTT ACATTTATCG TGGCAAAGAC GTTTTGAAAG GTCATGTTGG

5751 TCAGGAAGAG GAAGATGGCT CCGTTGATAT TCATCACACC CACTTGCGTG

5801 AGTTGTTGGC CCAAAAAGAT GAGGCCAATC AAGATGGCAA CCATCTGCAA

5851 ATTAAAATGT TACTCGCATC TCATTAATAT TCGCGAGTTA AATGAAATTT

5901 ATTTATCTTC TGCAAAACTA TAAACTATAC ATCTCATTGA AAAAAACTAA

5951 GAAGGGTGTG GAATCAGGCA ATTCTATCTA AAATCTAGCG AATTTGTTTC

6001 CAAGAATTGT AAGCGTTATA TCATTTGTTT CCACTGGAAC CACTCACCGT

6051 TGTCTGAATA AGTCGCACTT TTACGAGGAG TGGTTCCTTG AGCACCGACA

6101 GCCAGGATCG CCACAGGACC GCCCGGAACT GCATGAACCA GGTGGCCTTG

6151 TAGGTGTACC CATTCTCCGG CTGCTCCAGT GGCTTCTCCA GATTTTTGGT

6201 GGCCAACAAC TGCTCCATAT CCCGGGCTAC TTTGCTAATG GCAAAATTGT

6251 CGCATATCTT GGCGATCCGA TCACGGGACT CGATCTCCCG TCCGGGCACA

6301 ACGGCCAACA CCTGTACGTA AAAGTCCGCC GGATTGTAGT TGGTAGGACA

6351 CTGGGCACCC ACGCTGGATA GGAGTTGAGA TGTTATGTAA TACTAGATAC

6401 CCTTAATAAA CACATCGAAC TCACTAGGAA AAGAAGTCGA CGGCTTCGCT

6451 GGGAGTGCCC AAGAAAGCTA CCCTGCCCTC GGCCATCAGA AGGATCTTGT

6501 CAAAGAGCTC AAACAGCTCG GAAGACGGCT GATGAATGGT CAGGATGACG

6551 GTCTTGCCCT TCTGCGACAG CTTCTTCAGC ACCTGGACGA CGCTGTGGGC

6601 GGTAAAGGAG TCCAGTCCGG AGGTGGGCTC ATCGCAGATC AGAAGCGGCG

6651 GATCGGTTAG AGCCTCGGAG GCGAATGCCA GACGCTTCCT TTCTCCGCCG

6701 GACAGACCTT TCACCCTGCC GGGCACACCG ATGATCGTGT GCTGACATTT

6751 GCTGAGCGAA AGCTCCTGGA TCACCTGATC CACGCGGGCC ACTCGCTGCC

6801 GATAGGTCAG ATGTCGTGGC ATCCGCACCA TGGCTTGGAA AATCAGGTGT

6851 TCCCTGGCCG TTAGGGAGCC GATAAAGAGG TCATCCTGCT GGACATAGGC

6901 GCACCTGGCC TGCATCTCCT TGGCGTCCAC AGGTTGGCCA TTGAGCAGTC

6951 GCATCCCGGA TGGCGATACT TGGATGCCCT GCGGCGATCG AAAGGCAAGG

7001 GCATTCAGCA GGGTCGTCTT TCCGGCACCG GAACTGCCCA TCACGGCCAA

7051 AAGTTCGCCC GGATAGGCCA CGCCGCAAAC TGAGTTTCAA ATTGGTAATT

7101 GGACCCTTTA TTAAGATTTC ACACAGATCA GCCGACTGCG AATAGAAACT

7151 CACCGTTCTT GAGCAAATGT TTCCTGGGCG CCGGTATGTG TCGCTCGTTG

7201 CAGAATAGTC CGCGTGTCCG GTTGACCAGC TGCCGCCATC CGGAGCCCGG

7251 CTGATTGACC GCCCCAAAGA TGTCCATATT GTGCCAGGCA TAGGTGAGGT

7301 TCTCGGCTAG TTGGCCGCTC CCTGAACCGG AGTCCTCCGG CGGACTGGGT

7351 GGCCGGAGCG TGCCGTAGTT TTTGGCCTGC CCGAAGCCCT GGTTAATGCA

7401 GCTCTGCGAA GCCGCTCCGC TGTCACCCTG CAATGATAGG GGATCTCAAA

7451 TATCAACTAC TAGCGTTATG CTCATCTAAC CCCGAACAAA AAGTACCCCG

7501 AAGTATCCTA CGAAGTAGGT TTATACTTTT ATTTATTTTT TGTGCATCTA

7551 GGATCAGCTT AAAATATCTG GTTGTTATAT TTTTTGTAAA AAAGAATATA

7601 GTCGAAAATG AATGCCTTTA GATGTCTTGA TCATGATATG ATCTCAAAAA

7651 TTGTCTTATA TAGCGAGAAC AGCTACCAGA ATAATCTGTT TCGTGTCACT

7701 ATTTGTTTGT GCAATTGCGG TTTGGGATTT TTGTGGGTCG CAGTTCTCAC

7751 GCCGCATACA ATTTGATGTT GCAATCGCAG TTCCTATAGA TCAAGTGAAC

7801 TTAAGATGTA TGCACATGTA CTACTCACAT TGTTCAGATG CTCGGCAGAT

7851 GGGTGTTTGC TGCCTCCGCG AATTAATAGC TCCTGATCCT CTTGGCCCAT

7901 TGCCGGGATT TTTCACACTT TCCCCTGCTT ACCCACCCAA AACCAATCAC

7951 CACCCCAATC ACTCAAAAAA CAAACAAAAA TAAGAAGCGA GAGGAGTTTT

8001 GGCACAGCAC TTTGTGTTTA ATTGATGGCG TAAACCGCTT GGAGCTTCGT

8051 CACGAAACCG CTGACAAAAT GCAACTGAAG GCGGACATTG ACGCTACGTA

8101 ACGCTACAAA CGGTGGCGAA AGAGATAGCG GACGCAGCGG CGAAAGAGAC

8151 GGCGATATTT CTGTGGACAG AGAAGGAGGC AAACAGCGCT GACTTTGAGT

8201 GGAATGTCAT TTTGAGTGAG AGGTAATCGA AAGAACCTGG TACATCAAAT

8251 ACCCTTGGAT CGAAGTAAAT TTAAAACTGA TCAGATAAGT TCAATGATAT

8301 CCAGTGCAGT AAAAAAAAAA AATGTTTTTT TTATCTACTT TCCGCAAAAA

8351 TGGGTTTTAT TAACTTACAT ACATACTAGG CGCGCCCATA TGTTCGGCTT

8401 GTCGACATGC CCGCCGTGAC CGTCGAGAAC CCGCTGACGC TGCCCCGCGT

8451 ATCCGCACCC GCCGACGCCG TCGCACGTCC CGTGCTCACC GTGACCACCG

8501 CGCCCAGCGG TTTCGAGGGC GAGGGCTTCC CGGTGCGCCG CGCGTTCGCC

8551 GGGATCAACT ACCGCCACCT CGACCCGTTC ATCATGATGG ACCAGATGGG

8601 TGAGGTGGAG TACGCGCCCG GGGAGCCCAA GGGCACGCCC TGGCACCCGC

8651 ACCGCGGCTT CGAGACCGTG ACCTACATCG TCGACGGTAA CATGTGAGCA

8701 AAAGGCCAGC AAAAGGCCAG GAACCGTAAA AAGGCCGCGT TGCTGGCGTT

8751 TTTCCATAGG CTCCGCCCCC CTGACGAGCA TCACAAAAAT CGACGCTCAA

8801 GTCAGAGGTG GCGAAACCCG ACAGGACTAT AAAGATACCA GGCGTTTCCC

8851 CCTGGAAGCT CCCTCGTGCG CTCTCCTGTT CCGACCCTGC CGCTTACCGG

8901 ATACCTGTCC GCCTTTCTCC CTTCGGGAAG CGTGGCGCTT TCTCATAGCT

8951 CACGCTGTAG GTATCTCAGT TCGGTGTAGG TCGTTCGCTC CAAGCTGGGC

9001 TGTGTGCACG AACCCCCCGT TCAGCCCGAC CGCTGCGCCT TATCCGGTAA

9051 CTATCGTCTT GAGTCCAACC CGGTAAGACA CGACTTATCG CCACTGGCAG

9101 CAGCCACTGG TAACAGGATT AGCAGAGCGA GGTATGTAGG CGGTGCTACA

9151 GAGTTCTTGA AGTGGTGGCC TAACTACGGC TACACTAGAA GAACAGTATT

9201 TGGTATCTGC GCTCTGCTGA AGCCAGTTAC CTTCGGAAAA AGAGTTGGTA

9251 GCTCTTGATC CGGCAAACAA ACCACCGCTG GTAGCGGTGG TTTTTTTGTT

9301 TGCAAGCAGC AGATTACGCG CAGAAAAAAA GGATCTCAAG AAGATCCTTT

9351 GATCTTTTCT ACGGGGTCTG ACGCTCAGTG GAACGAAAAC TCACGTTAAG

9401 GGATTTTGGT CATGAGATTA TCAAAAAGGA TCTTCACCTA GATCCTTTTA

9451 AATTAAAAAT GAAGTTTTAA ATCAATCTAA AGTATATATG AGTAAACTTG

9501 GTCTGACAGT TACCAATGCT TAATCAGTGA GGCACCTATC TCAGCGATCT

9551 GTCTATTTCG TTCATCCATA GTTGCCTGAC TCCCCGTCGT GTAGATAACT

9601 ACGATACGGG AGGGCTTACC ATCTGGCCCC AGTGCTGCAA TGATACCGCG

9651 AGACCCACGC TCACCGGCTC CAGATTTATC AGCAATAAAC CAGCCAGCCG

9701 GAAGGGCCGA GCGCAGAAGT GGTCCTGCAA CTTTATCCGC CTCCATCCAG

9751 TCTATTAATT GTTGCCGGGA AGCTAGAGTA AGTAGTTCGC CAGTTAATAG

9801 TTTGCGCAAC GTTGTTGCCA TTGCTACAGG CATCGTGGTG TCACGCTCGT

9851 CGTTTGGTAT GGCTTCATTC AGCTCCGGTT CCCAACGATC AAGGCGAGTT

9901 ACATGATCCC CCATGTTGTG CAAAAAAGCG GTTAGCTCCT TCGGTCCTCC

9951 GATCGTTGTC AGAAGTAAGT TGGCCGCAGT GTTATCACTC ATGGTTATGG

10001 CAGCACTGCA TAATTCTCTT ACTGTCATGC CATCCGTAAG ATGCTTTTCT

10051 GTGACTGGTG AGTACTCAAC CAAGTCATTC TGAGAATAGT GTATGCGGCG

10101 ACCGAGTTGC TCTTGCCCGG CGTCAATACG GGATAATACC GCGCCACATA

10151 GCAGAACTTT AAAAGTGCTC ATCATTGGAA AACGTTCTTC GGGGCGAAAA

10201 CTCTCAAGGA TCTTACCGCT GTTGAGATCC AGTTCGATGT AACCCACTCG

10251 TGCACCCAAC TGATCTTCAG CATCTTTTAC TTTCACCAGC GTTTCTGGGT

10301 GAGCAAAAAC AGGAAGGCAA AATGCCGCAA AAAAGGGAAT AAGGGCGACA

10351 CGGAAATGTT GAATACTCAT ACTCTTCCTT TTTCAATATT ATTGAAGCAT

10401 TTATCAGGGT TATTGTCTCA TGAGCGGATA CATATTTGAA TGTATTTAGA

10451 AAAATAAACA AATAGGGGTT CCGCGCACAT TTCCCCGAAA AGTGCCACCT

10501 GAGTC

**HJP-207-IVS-Syn21-gp41-1^C^-Cre_BC-_WPREUw**

1 TAAGAAACCA TTATTATCAT GACATTAACC TATAAAAATA GGCGTATCAC

51 GAGGCCCTTT CGTCTTCAAG AATTCGTTTA TCACAAGTTT GTACAAAAAA

101 GCTGAACGAG AAACGTAAAA TGATATAAAT ATCAATATAT TAAATTAGAT

151 TTTGCATAAA AAACAGACTA CATAATACTG TAAAACACAA CATATCCAGT

201 CACTATGGCG GCCGCATTAG GCACCCCAGG CTTTACACTT TATGCTTCCG

251 GCTCGTATAA TGTGTGGATT TTGAGTTAGG ATCCGTCGAG ATTTTCAGGA

301 GCTAAGGAAG CTAAAATGGA GAAAAAAATC ACTGGATATA CCACCGTTGA

351 TATATCCCAA TGGCATCGTA AAGAACATTT TGAGGCATTT CAGTCAGTTG

401 CTCAATGTAC CTATAACCAG ACCGTTCAGC TGGATATTAC GGCCTTTTTA

451 AAGACCGTAA AGAAAAATAA GCACAAGTTT TATCCGGCCT TTATTCACAT

501 TCTTGCCCGC CTGATGAATG CTCATCCGGA ATTCCGTATG GCAATGAAAG

551 ACGGTGAGCT GGTGATATGG GATAGTGTTC ACCCTTGTTA CACCGTTTTC

601 CATGAGCAAA CTGAAACGTT TTCATCGCTC TGGAGTGAAT ACCACGACGA

651 TTTCCGGCAG TTTCTACACA TATATTCGCA AGATGTGGCG TGTTACGGTG

701 AAAACCTGGC CTATTTCCCT AAAGGGTTTA TTGAGAATAT GTTTTTCGTC

751 TCAGCCAATC CCTGGGTGAG TTTCACCAGT TTTGATTTAA ACGTGGCCAA

801 TATGGACAAC TTCTTCGCCC CCGTTTTCAC CATGGGCAAA TATTATACGC

851 AAGGCGACAA GGTGCTGATG CCGCTGGCGA TTCAGGTTCA TCATGCCGTT

901 TGTGATGGCT TCCATGTCGG CAGAATGCTT AATGAATTAC AACAGTACTG

951 CGATGAGTGG CAGGGCGGGG CGTAAACGCG TGGATCCGGC TTACTAAAAG

1001 CCAGATAACA GTATGCGTAT TTGCGCGCTG ATTTTTGCGG TATAAGAATA

1051 TATACTGATA TGTATACCCG AAGTATGTCA AAAAGAGGTA TGCTATGAAG

1101 CAGCGTATTA CAGTGACAGT TGACAGCGAC AGCTATCAGT TGCTCAAGGC

1151 ATATATGATG TCAATATCTC CGGTCTGGTA AGCACAACCA TGCAGAATGA

1201 AGCCCGTCGT CTGCGTGCCG AACGCTGGAA AGCGGAAAAT CAGGAAGGGA

1251 TGGCTGAGGT CGCCCGGTTT ATTGAAATGA ACGGCTCTTT TGCTGACGAG

1301 AACAGGGGCT GGTGAAATGC AGTTTAAGGT TTACACCTAT AAAAGAGAGA

1351 GCCGTTATCG TCTGTTTGTG GATGTACAGA GTGATATTAT TGACACGCCC

1401 GGGCGACGGA TGGTGATCCC CCTGGCCAGT GCACGTCTGC TGTCAGATAA

1451 AGTCTCCCGT GAACTTTACC CGGTGGTGCA TATCGGGGAT GAAAGCTGGC

1501 GCATGATGAC CACCGATATG GCCAGTGTGC CGGTCTCCGT TATCGGGGAA

1551 GAAGTGGCTG ATCTCAGCCA CCGCGAAAAT GACATCAAAA ACGCCATTAA

1601 CCTGATGTTC TGGGGAATAT AAATGTCAGG CTCCCTTATA CACAGCCAGT

1651 CTGCAGGTCG ACCATAGTGA CTGGATATGT TGTGTTTTAC AGTATTATGT

1701 AGTCTGTTTT TTATGCAAAA TCTAATTTAA TATATTGATA TTTATATCAT

1751 TTTACGTTTC TCGTTCAGCT TTCTTGTACA AAGTGGTGAT AAACGGCCGG

1801 CCGAGCTCGC CCGGGGATCG AGCGCAGCGG TATAAAAGGG CGCGGGGTGG

1851 CTGAGAGCAT CAGTTGTGAA TGAATGTTCG AGCCGAGCAG ACGTGCCGCT

1901 GCCTTCGTTA ATATCCTTTG AATAAGCCAA CTTTGAATCA CAAGACGCAT

1951 ACCAAACGGT ACCGCTAGAA AAAGGTAGGT TCAACCACTG ATGCCTAGGC

2001 ACACCGAAAC GACTAACCCT AATTCTTATC CTTTACTTCA GGGCTAGCAA

2051 CTTAAAAAAA AAAATCAAAA TGATGCTGAA GAAGATCCTG AAGATCGAGG

2101 AGCTGGATGA GCGCGAGCTG ATCGATATCG AGGTGAGCGG CAACCACCTG

2151 TTCTACGCCA ACGATATCCT GACCCACAAC AGCAATGCAG TGAGCCTGGT

2201 AATGCGTCGA ATACGCAAGG AAAACGTGGA CGCCGGCGAG AGGGCCAAAC

2251 AGGCGTTGGC TTTCGAACGC ACCGATTTCG ATCAGGTGCG GTCGTTGATG

2301 GAGAACAGCG ATCGATGCCA GGACATCCGG AACTTGGCGT TTTTGGGCAT

2351 TGCCTATAAC ACCCTCCTTC GCATCGCCGA GATAGCGCGT ATCCGAGTTA

2401 AAGACATTTC CCGTACAGAC GGAGGCCGCA TGCTTATTCA CATCGGACGC

2451 ACGAAAACCC TGGTCAGCAC TGCTGGCGTG GAGAAAGCAC TTTCCCTGGG

2501 TGTGACGAAA TTGGTCGAGC GATGGATCAG TGTGAGTGGC GTCGCCGATG

2551 ACCCCAACAA TTATCTGTTC TGCAGGGTGC GCAAAAATGG AGTGGCAGCC

2601 CCCTCGGCAA CAAGTCAGTT GAGTACGCGC GCGTTGGAGG GAATCTTCGA

2651 GGCCACTCAC CGATTGATTT ACGGTGCAAA AGATGACTCC GGCCAACGTT

2701 ACTTGGCCTG GTCCGGTCAC AGCGCACGTG TGGGTGCCGC CCGTGACATG

2751 GCGAGGGCCG GTGTTTCCAT CCCAGAAATC ATGCAAGCGG GCGGATGGAC

2801 CAACGTGAAT ATTGTAATGA ACTACATTCG TAATCTGGAT AGTGAAACAG

2851 GCGCAATGGT GCGCCTGCTG GAGGACGGAG ATTAAACCGG TGTTTAAACA

2901 ATCAACCTCT GGATTACAAA ATTTGTGAAA GATTGACTGG TATTCTTAAC

2951 TATGTTGCTC CTTTTACGCT ATGTGGATAC GCTGCTTTAA TGCCTTTGTA

3001 TCATGCTATT GCTTCCCGTA TGGCTTTCAT TTTCTCCTCC TTGTATAAAT

3051 CCTGGTTGCT GTCTCTTTAT GAGGAGTTGT GGCCCGTTGT CAGGCAACGT

3101 GGCGTGGTGT GCACTGTGTT TGCTGACGCA ACCCCCACTG GTTGGGGCAT

3151 TGCCACCACC TGTCAGCTCC TTTCCGGGAC TTTCGCTTTC CCCCTCCCTA

3201 TTGCCACGGC GGAACTCATC GCCGCCTGCC TTGCCCGCTG CTGGACAGGG

3251 GCTCGGCTGT TGGGCACTGA CAATTCCGTG GTGTTGTCGG GGAAATCATC

3301 GTCCTTTCCT TGGCTGCTCG CCTGTGTTGC CACCTGGATT CTGCGCGGGA

3351 CGTCCTTCTG CTACGTCCCT TCGGCCCTCA ATCCAGCGGA CCTTCCTTCC

3401 CGCGGCCTGC TGCCGGCTCT GCGGCCTCTT CCGCGTCTTC GCCTTCGCCC

3451 TCAGACGAGT CGGATCTCCC TTTGGGCCGC CTCCCCGCCT GAAGCTTATC

3501 GATACCGTCG ACTAAAGCCA AATAGAAAAT TATTCAGTTC CTGGCTTAAG

3551 TTTTTAAAAG TGATATTATT TATTTGGTTG TAACCAACCA AAAGAATGTA

3601 AATAACTAAT ACATAATTAT GTTAGTTTTA AGTTAGCAAC AAATTGATTT

3651 TAGCTATATT AGCTACTTGG TTAATAAATA GAATATATTT ATTTAAAGAT

3701 AATTGCGTTT TTATTGTCAG GGAGTGAGTT TGCTTAAAAA CTCGTTTAGA

3751 TCCACTAGTT CTAGAGCGGC CGCTGGCCAC GGGTGCGCAT GATCGTGCTC

3801 CTGTCGTTGA GGACCCGGCT AGGCTGGCGG GGTTGCCTTA CTGGTTAGCA

3851 GAATGAATCA CCGATACGCG AGCGAACGTG AAGCGACTGC TGCTGCAAAA

3901 CGTCTGCGAC CTGAGCAACA ACATGAATGG TCTTCGGTTT CCGTGTTTCG

3951 TAAAGTCTGG AAACGCGGAA GTCAGCGCCC TGCACCATTA TGTTCCGGAG

4001 GCGCGCCCTA GTTCCAGTGA AATCCAAGCA TTTTCTAAAT TAAATGTATT

4051 CTTATTATTA TAGTTGTTAT TTTTGATATA TATAAACAAC ACTATTATGC

4101 CCACCATTTT TTTGAGATGC ATCTACACAA GGAACAAACA CTGGATGTCA

4151 CTTTCAGTTC AAATTGTAAC GCTAATCACT CCGAACAGGT CACAAAAAAT

4201 TACCTTAAAA AGTCATAATA TTAAATTAGA ATAAATATAG CTGTGAGGGA

4251 AATATATACA AATATATTGG AGCAAATAAA TTGTACATAC AAATATTTAT

4301 TACTAATTTC TATTGAGACG AAATGAACCA CTCGGAACCA TTTGAGCGAA

4351 CCGAATCGCG CGGAACTAAC GACAGTCGCT CCAAGGTCGT CGAACAAAAG

4401 GTGAATGTGT TGCGGAGAGC GGGTGGGAGA CAGCGAAAGA GCAACTACGA

4451 AACGTGGTGT GGTGGAGGTG AATTATGAAG AGGGCGCGCG ATTTGAAAAG

4501 TATGTATATA AAAAATATAT CCCGGTGTTT TATGTAGCGA TAAACGAGTT

4551 TTTGATGTAA GGTATGCAGG TGTGTAAGTC TTTTGGTTAG AAGACAAATC

4601 CAAAGTCTAC TTGTGGGGAT GTTCGAAGGG GAAATACTTG TATTCTATAG

4651 GTCATATCTT GTTTTTATTG GCACAAATAT AATTACATTA GCTTTTTGAG

4701 GGGGCAATAA ACAGTAAACA CGATGGTAAT AATGGTAAAA AAAAAAAACA

4751 AGCAGTTATT TCGGATATAT GTCGGCTACT CCTTGCGTCG GGCCCGAAGT

4801 CTTAGAGCCA GATATGCGAG CACCCGGAAG CTCACGATGA GAATGGCCAG

4851 ACCCACGTAG TCCAGCGGCA GATCGGCGGC GGAGAAGTTA AGCGTCTCCA

4901 GGATGACCTT GCCCGAACTG GGGCACGTGG TGTTCGACGA TGTGCAGCTA

4951 ATTTCGCCCG GCTCCACGTC CGCCCATTGG TTAATCAGCA GACCCTCGTT

5001 GGCGTAACGG AACCATGAGA GGTACGACAA CCATTTGAGG TATACTGGCA

5051 CCGAGCCCGA GTTCAAGAAG AAGCCGCCAA AGAGCAGGAA TGGTATGATA

5101 ACCGGCGGAC CCACAGACAG CGCCATCGAG GTCGAGGAGC TGGCGCAGGA

5151 TATTAGATAT CCGAAGGACG TTGACACATT GGCCACCAGA GTGACCAGCG

5201 CCAGGCAGTT GAAGAAGTGC AGCACTCCGG CCCGCAGTCC GATCATCGGA

5251 TAGGCAATCG CCGTGAAGAC CAGTGGCACT GTGAGAAAAA GCGGCAATTC

5301 GGCAATCGTT TTGCCCAGAA AGTATGTGTC ACAGCGATAA AGTCGACTTC

5351 GGGCCTCCCT CATAAAAACT GGCAGCTCTG AGGTGAACAC CTAAATCGAA

5401 TCGATTCATT AGAAAGTTAG TAAATTATTG AAATGCAAAT GTATTCTAAA

5451 CATGACTTAC ATTTATCGTG GCAAAGACGT TTTGAAAGGT CATGTTGGTC

5501 AGGAAGAGGA AGATGGCTCC GTTGATATTC ATCACACCCA CTTGCGTGAG

5551 TTGTTGGCCC AAAAAGATGA GGCCAATCAA GATGGCAACC ATCTGCAAAT

5601 TAAAATGTTA CTCGCATCTC ATTAATATTC GCGAGTTAAA TGAAATTTAT

5651 TTATCTTCTG CAAAACTATA AACTATACAT CTCATTGAAA AAAACTAAGA

5701 AGGGTGTGGA ATCAGGCAAT TCTATCTAAA ATCTAGCGAA TTTGTTTCCA

5751 AGAATTGTAA GCGTTATATC ATTTGTTTCC ACTGGAACCA CTCACCGTTG

5801 TCTGAATAAG TCGCACTTTT ACGAGGAGTG GTTCCTTGAG CACCGACAGC

5851 CAGGATCGCC ACAGGACCGC CCGGAACTGC ATGAACCAGG TGGCCTTGTA

5901 GGTGTACCCA TTCTCCGGCT GCTCCAGTGG CTTCTCCAGA TTTTTGGTGG

5951 CCAACAACTG CTCCATATCC CGGGCTACTT TGCTAATGGC AAAATTGTCG

6001 CATATCTTGG CGATCCGATC ACGGGACTCG ATCTCCCGTC CGGGCACAAC

6051 GGCCAACACC TGTACGTAAA AGTCCGCCGG ATTGTAGTTG GTAGGACACT

6101 GGGCACCCAC GCTGGATAGG AGTTGAGATG TTATGTAATA CTAGATACCC

6151 TTAATAAACA CATCGAACTC ACTAGGAAAA GAAGTCGACG GCTTCGCTGG

6201 GAGTGCCCAA GAAAGCTACC CTGCCCTCGG CCATCAGAAG GATCTTGTCA

6251 AAGAGCTCAA ACAGCTCGGA AGACGGCTGA TGAATGGTCA GGATGACGGT

6301 CTTGCCCTTC TGCGACAGCT TCTTCAGCAC CTGGACGACG CTGTGGGCGG

6351 TAAAGGAGTC CAGTCCGGAG GTGGGCTCAT CGCAGATCAG AAGCGGCGGA

6401 TCGGTTAGAG CCTCGGAGGC GAATGCCAGA CGCTTCCTTT CTCCGCCGGA

6451 CAGACCTTTC ACCCTGCCGG GCACACCGAT GATCGTGTGC TGACATTTGC

6501 TGAGCGAAAG CTCCTGGATC ACCTGATCCA CGCGGGCCAC TCGCTGCCGA

6551 TAGGTCAGAT GTCGTGGCAT CCGCACCATG GCTTGGAAAA TCAGGTGTTC

6601 CCTGGCCGTT AGGGAGCCGA TAAAGAGGTC ATCCTGCTGG ACATAGGCGC

6651 ACCTGGCCTG CATCTCCTTG GCGTCCACAG GTTGGCCATT GAGCAGTCGC

6701 ATCCCGGATG GCGATACTTG GATGCCCTGC GGCGATCGAA AGGCAAGGGC

6751 ATTCAGCAGG GTCGTCTTTC CGGCACCGGA ACTGCCCATC ACGGCCAAAA

6801 GTTCGCCCGG ATAGGCCACG CCGCAAACTG AGTTTCAAAT TGGTAATTGG

6851 ACCCTTTATT AAGATTTCAC ACAGATCAGC CGACTGCGAA TAGAAACTCA

6901 CCGTTCTTGA GCAAATGTTT CCTGGGCGCC GGTATGTGTC GCTCGTTGCA

6951 GAATAGTCCG CGTGTCCGGT TGACCAGCTG CCGCCATCCG GAGCCCGGCT

7001 GATTGACCGC CCCAAAGATG TCCATATTGT GCCAGGCATA GGTGAGGTTC

7051 TCGGCTAGTT GGCCGCTCCC TGAACCGGAG TCCTCCGGCG GACTGGGTGG

7101 CCGGAGCGTG CCGTAGTTTT TGGCCTGCCC GAAGCCCTGG TTAATGCAGC

7151 TCTGCGAAGC CGCTCCGCTG TCACCCTGCA ATGATAGGGG ATCTCAAATA

7201 TCAACTACTA GCGTTATGCT CATCTAACCC CGAACAAAAA GTACCCCGAA

7251 GTATCCTACG AAGTAGGTTT ATACTTTTAT TTATTTTTTG TGCATCTAGG

7301 ATCAGCTTAA AATATCTGGT TGTTATATTT TTTGTAAAAA AGAATATAGT

7351 CGAAAATGAA TGCCTTTAGA TGTCTTGATC ATGATATGAT CTCAAAAATT

7401 GTCTTATATA GCGAGAACAG CTACCAGAAT AATCTGTTTC GTGTCACTAT

7451 TTGTTTGTGC AATTGCGGTT TGGGATTTTT GTGGGTCGCA GTTCTCACGC

7501 CGCATACAAT TTGATGTTGC AATCGCAGTT CCTATAGATC AAGTGAACTT

7551 AAGATGTATG CACATGTACT ACTCACATTG TTCAGATGCT CGGCAGATGG

7601 GTGTTTGCTG CCTCCGCGAA TTAATAGCTC CTGATCCTCT TGGCCCATTG

7651 CCGGGATTTT TCACACTTTC CCCTGCTTAC CCACCCAAAA CCAATCACCA

7701 CCCCAATCAC TCAAAAAACA AACAAAAATA AGAAGCGAGA GGAGTTTTGG

7751 CACAGCACTT TGTGTTTAAT TGATGGCGTA AACCGCTTGG AGCTTCGTCA

7801 CGAAACCGCT GACAAAATGC AACTGAAGGC GGACATTGAC GCTACGTAAC

7851 GCTACAAACG GTGGCGAAAG AGATAGCGGA CGCAGCGGCG AAAGAGACGG

7901 CGATATTTCT GTGGACAGAG AAGGAGGCAA ACAGCGCTGA CTTTGAGTGG

7951 AATGTCATTT TGAGTGAGAG GTAATCGAAA GAACCTGGTA CATCAAATAC

8001 CCTTGGATCG AAGTAAATTT AAAACTGATC AGATAAGTTC AATGATATCC

8051 AGTGCAGTAA AAAAAAAAAA TGTTTTTTTT ATCTACTTTC CGCAAAAATG

8101 GGTTTTATTA ACTTACATAC ATACTAGGCG CGCCCATATG TTCGGCTTGT

8151 CGACATGCCC GCCGTGACCG TCGAGAACCC GCTGACGCTG CCCCGCGTAT

8201 CCGCACCCGC CGACGCCGTC GCACGTCCCG TGCTCACCGT GACCACCGCG

8251 CCCAGCGGTT TCGAGGGCGA GGGCTTCCCG GTGCGCCGCG CGTTCGCCGG

8301 GATCAACTAC CGCCACCTCG ACCCGTTCAT CATGATGGAC CAGATGGGTG

8351 AGGTGGAGTA CGCGCCCGGG GAGCCCAAGG GCACGCCCTG GCACCCGCAC

8401 CGCGGCTTCG AGACCGTGAC CTACATCGTC GACGGTAACA TGTGAGCAAA

8451 AGGCCAGCAA AAGGCCAGGA ACCGTAAAAA GGCCGCGTTG CTGGCGTTTT

8501 TCCATAGGCT CCGCCCCCCT GACGAGCATC ACAAAAATCG ACGCTCAAGT

8551 CAGAGGTGGC GAAACCCGAC AGGACTATAA AGATACCAGG CGTTTCCCCC

8601 TGGAAGCTCC CTCGTGCGCT CTCCTGTTCC GACCCTGCCG CTTACCGGAT

8651 ACCTGTCCGC CTTTCTCCCT TCGGGAAGCG TGGCGCTTTC TCATAGCTCA

8701 CGCTGTAGGT ATCTCAGTTC GGTGTAGGTC GTTCGCTCCA AGCTGGGCTG

8751 TGTGCACGAA CCCCCCGTTC AGCCCGACCG CTGCGCCTTA TCCGGTAACT

8801 ATCGTCTTGA GTCCAACCCG GTAAGACACG ACTTATCGCC ACTGGCAGCA

8851 GCCACTGGTA ACAGGATTAG CAGAGCGAGG TATGTAGGCG GTGCTACAGA

8901 GTTCTTGAAG TGGTGGCCTA ACTACGGCTA CACTAGAAGA ACAGTATTTG

8951 GTATCTGCGC TCTGCTGAAG CCAGTTACCT TCGGAAAAAG AGTTGGTAGC

9001 TCTTGATCCG GCAAACAAAC CACCGCTGGT AGCGGTGGTT TTTTTGTTTG

9051 CAAGCAGCAG ATTACGCGCA GAAAAAAAGG ATCTCAAGAA GATCCTTTGA

9101 TCTTTTCTAC GGGGTCTGAC GCTCAGTGGA ACGAAAACTC ACGTTAAGGG

9151 ATTTTGGTCA TGAGATTATC AAAAAGGATC TTCACCTAGA TCCTTTTAAA

9201 TTAAAAATGA AGTTTTAAAT CAATCTAAAG TATATATGAG TAAACTTGGT

9251 CTGACAGTTA CCAATGCTTA ATCAGTGAGG CACCTATCTC AGCGATCTGT

9301 CTATTTCGTT CATCCATAGT TGCCTGACTC CCCGTCGTGT AGATAACTAC

9351 GATACGGGAG GGCTTACCAT CTGGCCCCAG TGCTGCAATG ATACCGCGAG

9401 ACCCACGCTC ACCGGCTCCA GATTTATCAG CAATAAACCA GCCAGCCGGA

9451 AGGGCCGAGC GCAGAAGTGG TCCTGCAACT TTATCCGCCT CCATCCAGTC

9501 TATTAATTGT TGCCGGGAAG CTAGAGTAAG TAGTTCGCCA GTTAATAGTT

9551 TGCGCAACGT TGTTGCCATT GCTACAGGCA TCGTGGTGTC ACGCTCGTCG

9601 TTTGGTATGG CTTCATTCAG CTCCGGTTCC CAACGATCAA GGCGAGTTAC

9651 ATGATCCCCC ATGTTGTGCA AAAAAGCGGT TAGCTCCTTC GGTCCTCCGA

9701 TCGTTGTCAG AAGTAAGTTG GCCGCAGTGT TATCACTCAT GGTTATGGCA

9751 GCACTGCATA ATTCTCTTAC TGTCATGCCA TCCGTAAGAT GCTTTTCTGT

9801 GACTGGTGAG TACTCAACCA AGTCATTCTG AGAATAGTGT ATGCGGCGAC

9851 CGAGTTGCTC TTGCCCGGCG TCAATACGGG ATAATACCGC GCCACATAGC

9901 AGAACTTTAA AAGTGCTCAT CATTGGAAAA CGTTCTTCGG GGCGAAAACT

9951 CTCAAGGATC TTACCGCTGT TGAGATCCAG TTCGATGTAA CCCACTCGTG

10001 CACCCAACTG ATCTTCAGCA TCTTTTACTT TCACCAGCGT TTCTGGGTGA

10051 GCAAAAACAG GAAGGCAAAA TGCCGCAAAA AAGGGAATAA GGGCGACACG

10101 GAAATGTTGA ATACTCATAC TCTTCCTTTT TCAATATTAT TGAAGCATTT

10151 ATCAGGGTTA TTGTCTCATG AGCGGATACA TATTTGAATG TATTTAGAAA

10201 AATAAACAAA TAGGGGTTCC GCGCACATTT CCCCGAAAAG TGCCACCTGA

10251 GTC

**HJP-225-actin5C-Flox-IVS-Syn21myr-tdTomato-p10w**

1 TAAGAAACCA TTATTATCAT GACATTAACC TATAAAAATA GGCGTATCAC

51 GAGGCCCTTT CGTCTTCAAG AATTCGTTTA TCACAAGTTT GTACAAAAAA

101 GCAGGCTCCG CGGCCGCAAT TCTATATTCT AAAAACACAA ATGATACTTC

151 TAAAAAAAAA TCATGAATGG CATCAACTCT GAATCAAATC TTTGCAGATG

201 CACCTACTTC TCATTTCCAC TGTCACATCA TTTTTCCAGA TCTCGCTGCC

251 TGTTATGTGG CCCACAAACC AAGACACGTT TTATGGCCAT TAAAGCTGGC

301 TGATCGTCGC CAAACACCAA ATACATAATG AATATGTACA CATTCGAGAA

351 AGAAGCGATC AAAGAAGCGT CTTCGGGCGG AGTAGGAGAA TGCGGAGGAG

401 AAGGAGAACG AGCTGATCTA GTATCTCTCC ACAATCCAAT GCCAACTGAC

451 CAACTGGCCA TATTCGGAGC AATTTGAAGC CAATTTCCAT CGCCTGGCGA

501 TCGCTCCATT CTTGGCTATA TGTTTTTCAC CGTTACCCGG GGCCATTTTC

551 AAAGACTCGT CGGCAAGATA AGATTGTGTC ACTCGCTGTC TCTCTTCATT

601 TGTCGAAGAA TGCTGAGGAA TTTCGCGATG ACGTCGGCGA GTATTTTGAA

651 GAATGAGAAT AATTTGTATT TATACGAAAA TCAGTTAGTG GAATTTTCTA

701 CAAAAACATG TTATCTATAG ATAATTTTGT TGCAAAATAT GTTGACTATG

751 ACAAAGATTG TATGTATATA CCTTTAATGT ATTCTCATTT TCTTATGTAT

801 TTATAATGGC AATGATGATA CTGATGATAT TTTAAGATGA TGCCAGACCA

851 AAAGGCTTGA ATTTCTGCGT CTTTTGCCGA ACGCAGTGCA TGTGCAATTG

901 TTGTTTTTTG GAATATTCAA TTTTCGGACT GTCCGCTTTG ATTTCAGTTT

951 CTTGGCTTAT TCAAAAAGCA AAGTAAAGCC AAAAAAGCGA GATGGCAATA

1001 CCAAATGCGG CAAAACGGTA GTGGAAGGAA AGGGGTGCGG GGCAGCGGAA

1051 GGAAGGGTGG GGCGGGGCGT GGCGGGGTCT GTGGCTGGGC GCGACGTCAC

1101 CGACGTTGGA GCCACTCCTT TGACCATGTG TGCGTGTGTG TATTATTCGT

1151 GTCTCGCCAC TCGCCGGTTG TTTTTTTCTT TTTATGCTGC GCTCTCTCTA

1201 GCGCCATCTC GCTTACGCAT GCTCAACGCA CCGCATGTTG CCGTTTCCTT

1251 TTATGCGTCA TTTTGGCTCG AAATAGGCAA TTATTTAAAC AAAGATTAGT

1301 CAACGAAAAC GCTAAAATAA ATAAGTCTAC AATATGGTTA CTTATTGCCA

1351 TGTGTGTGCA GCCAACGATA GCAACAAAAG CAACAACACA GGTGGCTTTC

1401 CCTCTTTCAC TTTTTGTTTG CAAGCCGCGT GCGAGCAAGA CGGCACGACC

1451 GGCAAACGCA ATTACGCTGA CAAAGAGCAG ACGAAGTTTT GGCGAAAAAC

1501 ATCAAGGCGC CTGATACGAA TGCATTTGCA ATAACAATTG CGATATTTAA

1551 TATTGTTTAT GAAGCTGTTT GACTTCAAAA CACACAAAAA AAAAAATAAA

1601 ACAAATTATT TGAAAGAGAA TTAGGAATCG GACGCTTATC GTTAGGGTAA

1651 CAACAAGAAA TGCTTACTGA GTCACAGCCT CTGGAAAACT GCCGCAAGCC

1701 AGAGAGAGAG AGAAAAAGAG GGAGAGCAGC TTAGACCGCA TGTGCTTGTG

1751 TGTGAGGCGT CTCTCTCTTC GTCTCTGTTG CGCAAACGCA TAGACTGCAC

1801 TGAAAAAATC GATTACCTAT TTTTTATGAA TGAATATTTG CACTATTACT

1851 ATTCAAAACT ATTAAGATAG CAATCACATT CAATAGCCAA ATACTATACC

1901 ACCTGAGCGA TGCAACGAAA TGATCAATTT GAGCAAAAAT GCTGCATATT

1951 TAGGACGGCA TCATTATAGA AATGCTTCTT GCTGTGTACT TTTCTCTCGT

2001 CTGGCAGCTG TTTCGCCGTT ATTGTTAAAA CCGGCTTAAG TTAGGTGTGT

2051 TTTCTACGAC TAGTGAATGC CCTACTAGAA GATGTGTGTT GCACAAAATG

2101 TCCCTGGAAT AACCAATTTG AAGTGCAGAT AGCAGTAAAC GTAAGCTAAT

2151 ATGAATATTA TTTAACTGTA ATGTTTTAAT ATCGCTGGAC ATTACTAATA

2201 AACCCACTAT AAACACATGT ACATATGTAT GTTTTGGCAT ACAATGAGTA

2251 GTTGGGGAAA AAATGTGTAA AAGCACCGTG ACCATCACAG CATAAAGATA

2301 ACCAGCTGAA GTATCGAATA TGAGTAACCC CCAAATTGAA TCACATGCCG

2351 CAACTGATAG GACCCATGGA AGTACACTCT TCATGGCGAT ATACAAGACA

2401 CACACAAGCA CGAACACCCA GTTGCGGAGG AAATTCTCCG TAAATGAAAA

2451 CCCAATCGGC GAACAATTCA TACCCATATA TGGTAAAAGT TTTGAACGCG

2501 ACTTGAGAGC GGAGAGCATT GCGGCTGATA AGGTTTTAGC GCTAAGCGGG

2551 CTTTATAAAA CGGGCTGCGG GACCAGTTTT CATATCACTA CCGTTTGAGT

2601 TCTTGTGCTG TGTGGATACT CCTCCCGACA CAAAGCCGCT CCATCAGCCA

2651 GCAGTCGTCT AATCCAGAGA CACCAAACCG AAAGACTTAA TTTATATTTA

2701 TTTAATTAAT TTTAATAAAA CACACCAAAT GTAAGTAGCT TTCCCCTTCC

2751 CAACAACAAA ACACCATCGA ACCACTCCCA CCAAGAAAAA GCAATAATCG

2801 AGAAAAGCCG CGGAAAATGT GTGATTTTTT TTGTAAACAA AACATTTTTT

2851 TATGTGCCAG TGCTGAAAGT GATCAAAAAA TACTAGCCAC GAGCTAAAGA

2901 GTTATTGTAT TGACCAAAAC TCCAAAAATA CCCAAGTTTG GCCCTAAATT

2951 GTCAATCAAA ATACCAATAG GTCGAAAGAC ATCAAAATTA ACAAAACCAG

3001 GGTTTCAAAT ACCATAACTC AAGAATCAGG ATTACAACTG CAGATTTCAG

3051 GATATATACA TACAAATTAT ACGAAATTAT AAAAACCAAA GCAATTCAAT

3101 AGCCCCAACT CAAATGTTAG GATCTAATAT AGTGTTTAAA GCCAAGCTCG

3151 CTGATGTGGG CGTGTCACGA TTTCACCCAA AGATATGCCA AATTACGAAT

3201 TGCAAATCAA TTCGCCAACA CTTTCTTTTT TTCCCACGCC CTAAAACACC

3251 AGATCATCCA TAAATGTACA TACATACAGT ATATGCATAT TATAATCTGT

3301 AAAACTAGAT CAGGTTCTTG AAAATAGTGA CGTAGGCAGC CGTTTTGGCT

3351 GAAGCAGAAA TTTTTGCCGG TTTTTCAAAG TTGTAGTTGC AAAAATGGAG

3401 AAAACCTTCG AGCATTCGTT CATATACACA CACTCACGCG CAAAATAACG

3451 AGAGAGAGTG TATGTGTGTG TGAGAGAGCG AAAGCCAGAC GACGGTTTGC

3501 TTTTCGCCTC GAAACATGAC CATATATGGT CACAAAACTT GGCCGCCGCA

3551 ATTCAACACA CCAGCGCTCT CCTTCGCACC CATAGCGACC ATGGCGCGGA

3601 GCGAGCGAGA TGGCGAGAGC GAGCGACGCC TATGGCGACG TCGACGCAGG

3651 CAGCGATTGA AAAACGCAGT TAACTGGCAT TCAACATTCA CCAGCCACTT

3701 TCAGTCGGTT TATTCCAGTC ATTCCTTTCA AACCGTGCGG TCGCTTAGCT

3751 CAGCCTCGCC ACTTGCGTTT ACAGTAGTTT TCACGCCTTG AATTTGTTAA

3801 ATCGAACAAA AAGGTAAAGT TTAACTAGCT TTGAAAAGTT TCGTGGCTCT

3851 TAATTGTTAA ATTTTCTAGA GTGCGTTTAG TGTTTTTTTT TTTTTTTATT

3901 TTGTAATGTT AATTTCGGGT TCCAATTCGA GTTTTAGGCA GCCGCCATTT

3951 TAAGGGCGCA TACACACAGG CAACTGTGCT CTCTTTGCGG CTTTCTTTTG

4001 CACCGGCATT CGTTAAGTGT CGTCTAGAAG CTTCTCCCCT CCCTTTTCGG

4051 CATATTCGTA TTGTGGTTTT AATTTTTCGG GGCGGGGCCT TCTATTTTGT

4101 AACTGTTCTT TTAATTTCTT ATTACAATTC GATCGCAAGT GAAAATCAGT

4151 TTTCAATCGG AAAAGTATTT TTTTATGAAA TTTTTTTTTG TCCAAGATTA

4201 AAATTTTGTA CTAAAAAAAC GTACATTGCA TTGAGTGATT TTTAATTGTA

4251 CACGAAAAAC AAGTTAGTTT GTTATGACAA TTGTACTTTG GTAGACCAGC

4301 GCAGTCCAAG GAAACCACGC AAATTCTCAG TTTTTTTTTT GCCATTTCTA

4351 CATTACCAAA TAAGGTAACC AAAAACTAAT GGGAAATCCG CATTCTTTCC

4401 ATTGCAGCTT ACAGGATCGA TCCCCGGGTA CCGCGCCGAC CCAGCTTTCT

4451 TGTACAAAGT GGTGATAAAC GGCCGGCATA ACTTCGTATA ATGTATGCTA

4501 TACGAAGTTA TGTCGACTAA AGCCAAATAG AAAATTATTC AGTTCCTGGC

4551 TTAAGTTTTT AAAAGTGATA TTATTTATTT GGTTGTAACC AACCAAAAGA

4601 ATGTAAATAA CTAATACATA ATTATGTTAG TTTTAAGTTA GCAACAAATT

4651 GATTTTAGCT ATATTAGCTA CTTGGTTAAT AAATAGAATA TATTTATTTA

4701 AAGATAATTG CGTTTTTATT GTCAGGGAGT GAGTTTGCTT AAAAACTCGT

4751 TTAGGTTTGT CCTCCCGAAA TTATTTATTT AAATGCGATG GAGAGTTGGC

4801 GCCGAATCGA AAACTTTACG CGCTTAAAAG CACGAGTTGG CATCCCTAAC

4851 GCGTAGGATC TTTGTGAAGG AACCTTACTT CTGTGGTGTG ACATAATTGG

4901 ACAAACTACC TACAGAGATT TAAAGCTCTA AGGTAAATAT AAAATTTTTA

4951 AGTGTATAAT GTGTTAAACT ACTGATTCTA ATTGTTTGTG TATTTTAGAT

5001 TCCAACCTAT GGAACTGATG AATGGGAGCA GTGGTGGAAT GCCTTTAATG

5051 AGGAAAACCT GTTTTGCTCA GAAGAAATGC CATCTAGTGA TGATGAGGCT

5101 ACTGCTGACT CTCAACATTC TACTCCTCCA AAAAAGAAGA GAAAGGTAGA

5151 AGACCCCAAG GACTTTCCTT CAGAATTGCT AAGTTTTTTG AGTCATGCTG

5201 TGTTTAGTAA TAGAACTCTT GCTTGCTTTG CTATTTACAC CACAAAGGAA

5251 AAAGCTGCAC TGCTATACAA GAAAATTATG GAAAAATATT TGATGTATAG

5301 TGCCTTGACT AGAGATCATA ATCAGCCATA CCACATTTGT AGAGGTTTTA

5351 CTTGCTTTAA AAAACCTCCC ACACCTCCCC CTGAACCTGA AACATAAAAT

5401 GAATGCAATT GTTGTTGTTA ACTTGTTTAT TGCAGCTTAT AATGGTTACA

5451 AATAAAGCAA TAGCATCACA AATTTCACAA ATAAAGCATT TTTTTCACTG

5501 CATTCTAGTT GTGGTTTGTC CAAACTCATC AATGTATCTT ATCATGTCTG

5551 GATCCCTAGG ATAACTTCGT ATAATGTATG CTATACGAAG TTATGGTACC

5601 AAAAGGTAGG TTCAACCACT GATGCCTAGG CACACCGAAA CGACTAACCC

5651 TAATTCTTAT CCTTTACTTC AGGAGGCCTA CCGGTGCTAG AACTTAAAAA

5701 AAAAAATCAA AATGGGCAAC AAATGCTGCA GCAAGCGACA GGATCAGGAA

5751 CTGGCACTGG CCTATCCCAC TGGGGGCTAC AAGAAATCCG ACTACACCTT

5801 TGGCCAGACG CACATCAACA GCAGCGGCGG CGGCAACATG GGCGGCGTTC

5851 TTGGCCAGAA GCATAACAAC GGTGGCTCGC TGGACTCGCG CTACACGCCC

5901 GATCCCAATC ATCGGGGTCC GTTGAAAATC GGCGGAAAGG GCGGCGTTGA

5951 CATCATCAGA CCACGCGGAT CCATGGTGTC GAAAGGCGAG GAGGTTATCA

6001 AGGAGTTTAT GCGATTTAAG GTCCGAATGG AGGGCAGTAT GAATGGTCAT

6051 GAATTTGAGA TTGAGGGTGA AGGAGAGGGC CGACCGTACG AAGGTACGCA

6101 GACGGCGAAG TTGAAGGTCA CGAAAGGAGG ACCTCTGCCA TTCGCCTGGG

6151 ATATCCTGTC CCCTCAGTTT ATGTACGGAA GCAAGGCATA CGTGAAGCAC

6201 CCCGCCGATA TTCCAGATTA TAAGAAACTC TCCTTCCCCG AGGGATTCAA

6251 GTGGGAGCGC GTTATGAACT TTGAGGATGG AGGACTTGTC ACGGTCACTC

6301 AGGACAGCAG TCTGCAGGAC GGAACCCTGA TCTACAAGGT TAAGATGCGA

6351 GGTACGAACT TCCCACCGGA TGGTCCGGTG ATGCAGAAAA AGACGATGGG

6401 ATGGGAGGCG AGCACCGAGC GACTGTACCC GCGTGATGGA GTCCTCAAGG

6451 GCGAAATTCA TCAGGCCCTG AAATTGAAAG ATGGTGGCCA CTATTTGGTG

6501 GAGTTCAAGA CTATCTACAT GGCAAAGAAG CCCGTACAGC TGCCGGGCTA

6551 TTATTACGTT GACACGAAGC TCGATATCAC GAGTCATAAC GAGGACTACA

6601 CAATCGTTGA GCAATACGAG CGCAGCGAGG GTCGCCACCA CCTTTTCCTG

6651 GGACATGGCA CTGGCTCGAC CGGCTCGGGC AGCAGCGGTA CAGCAAGCAG

6701 TGAGGACAAC AACATGGCCG TGATTAAGGA ATTTATGAGG TTCAAGGTAC

6751 GTATGGAGGG TTCCATGAAC GGCCATGAGT TCGAGATTGA GGGAGAGGGT

6801 GAAGGCCGAC CGTACGAAGG CACGCAGACT GCGAAACTGA AGGTGACAAA

6851 GGGTGGCCCG CTGCCATTTG CCTGGGATAT TCTGAGCCCT CAGTTCATGT

6901 ACGGTAGCAA GGCATACGTC AAACACCCTG CAGATATCCC CGATTACAAG

6951 AAGTTGTCCT TTCCCGAGGG TTTTAAGTGG GAGCGGGTGA TGAACTTCGA

7001 AGACGGAGGT CTTGTCACGG TGACACAGGA TAGTTCCCTG CAGGACGGCA

7051 CTCTGATCTA CAAAGTCAAG ATGCGAGGCA CCAATTTTCC ACCGGATGGA

7101 CCAGTGATGC AAAAGAAGAC CATGGGTTGG GAAGCTAGTA CGGAACGCCT

7151 GTATCCGAGG GATGGTGTGC TCAAGGGCGA GATCCACCAG GCATTGAAAC

7201 TTAAGGACGG TGGACATTAC TTGGTCGAGT TCAAAACTAT TTACATGGCT

7251 AAGAAACCCG TCCAGCTGCC CGGCTACTAT TATGTCGATA CCAAGCTGGA

7301 CATCACCTCC CACAATGAAG ACTACACCAT CGTGGAGCAG TACGAACGCA

7351 GTGAGGGCCG CCACCACTTG TTCCTGTACG GTATGGACGA GCTTTACAAG

7401 TAAAAACATG AATCGTTTTT AAAATAACAA ATCAATTGTT TTATAATATT

7451 CGTACGATTC TTTGATTATG TAATAAAATG TGATCATTAG GAAGATTACG

7501 AAAAATATAA AAAATATGAG TTCTGTGTGT ATAACAAATG CTGTAAACGC

7551 CACAATTGTG TTTGTTGCAA ATAAACCCAT GATTATTTGA TTAAAATTGT

7601 TGTTTTCTTT GTTCATAGAC AATAGTGTGT TTTGCCTAAA CGTGTACTGC

7651 ATAAACTCCA TGCGAGTGTA TAGCGAGCTA GTGGCTAACG CTTGCCCCAC

7701 CAAAGTAGAT TCGTCAAAAT CCTCAATTTC ATCACCCTCC TCCAAGTTTA

7751 ACATTTGGCC GTCGGAATTA ACTTCTAAAG ATGCCACATA ATCTAATAAA

7801 TGAAATAGAG ATTCAAACGT GGCGTCATCG TCCGTTTCGA CCATTTCCGA

7851 AAAGAACTCG GGCATAAACT CTATGATTTC TCTGGACGTG GTGTTGTCGA

7901 AACTCTCAAA GTACGCAGTC AGGAACGTGC GCGACATGTC GTCGGGAAAC

7951 TCGCGCGGAA ACATGTTGTT GTAACCGAAC GGGTCCCATA GCGCCAAAAC

8001 CAAATCTGCC AGCGTCAATA GAATGAGCAC GATGCCGACA ATGGAGCTGG

8051 CTTGGATAGC GATTCGAGTT AACTCTAGAG CGGCCGCTGG CCACGGGTGC

8101 GCATGATCGT GCTCCTGTCG TTGAGGACCC GGCTAGGCTG GCGGGGTTGC

8151 CTTACTGGTT AGCAGAATGA ATCACCGATA CGCGAGCGAA CGTGAAGCGA

8201 CTGCTGCTGC AAAACGTCTG CGACCTGAGC AACAACATGA ATGGTCTTCG

8251 GTTTCCGTGT TTCGTAAAGT CTGGAAACGC GGAAGTCAGC GCCCTGCACC

8301 ATTATGTTCC GGAGGCGCGC CCTAGTTCCA GTGAAATCCA AGCATTTTCT

8351 AAATTAAATG TATTCTTATT ATTATAGTTG TTATTTTTGA TATATATAAA

8401 CAACACTATT ATGCCCACCA TTTTTTTGAG ATGCATCTAC ACAAGGAACA

8451 AACACTGGAT GTCACTTTCA GTTCAAATTG TAACGCTAAT CACTCCGAAC

8501 AGGTCACAAA AAATTACCTT AAAAAGTCAT AATATTAAAT TAGAATAAAT

8551 ATAGCTGTGA GGGAAATATA TACAAATATA TTGGAGCAAA TAAATTGTAC

8601 ATACAAATAT TTATTACTAA TTTCTATTGA GACGAAATGA ACCACTCGGA

8651 ACCATTTGAG CGAACCGAAT CGCGCGGAAC TAACGACAGT CGCTCCAAGG

8701 TCGTCGAACA AAAGGTGAAT GTGTTGCGGA GAGCGGGTGG GAGACAGCGA

8751 AAGAGCAACT ACGAAACGTG GTGTGGTGGA GGTGAATTAT GAAGAGGGCG

8801 CGCGATTTGA AAAGTATGTA TATAAAAAAT ATATCCCGGT GTTTTATGTA

8851 GCGATAAACG AGTTTTTGAT GTAAGGTATG CAGGTGTGTA AGTCTTTTGG

8901 TTAGAAGACA AATCCAAAGT CTACTTGTGG GGATGTTCGA AGGGGAAATA

8951 CTTGTATTCT ATAGGTCATA TCTTGTTTTT ATTGGCACAA ATATAATTAC

9001 ATTAGCTTTT TGAGGGGGCA ATAAACAGTA AACACGATGG TAATAATGGT

9051 AAAAAAAAAA AACAAGCAGT TATTTCGGAT ATATGTCGGC TACTCCTTGC

9101 GTCGGGCCCG AAGTCTTAGA GCCAGATATG CGAGCACCCG GAAGCTCACG

9151 ATGAGAATGG CCAGACCCAC GTAGTCCAGC GGCAGATCGG CGGCGGAGAA

9201 GTTAAGCGTC TCCAGGATGA CCTTGCCCGA ACTGGGGCAC GTGGTGTTCG

9251 ACGATGTGCA GCTAATTTCG CCCGGCTCCA CGTCCGCCCA TTGGTTAATC

9301 AGCAGACCCT CGTTGGCGTA ACGGAACCAT GAGAGGTACG ACAACCATTT

9351 GAGGTATACT GGCACCGAGC CCGAGTTCAA GAAGAAGCCG CCAAAGAGCA

9401 GGAATGGTAT GATAACCGGC GGACCCACAG ACAGCGCCAT CGAGGTCGAG

9451 GAGCTGGCGC AGGATATTAG ATATCCGAAG GACGTTGACA CATTGGCCAC

9501 CAGAGTGACC AGCGCCAGGC AGTTGAAGAA GTGCAGCACT CCGGCCCGCA

9551 GTCCGATCAT CGGATAGGCA ATCGCCGTGA AGACCAGTGG CACTGTGAGA

9601 AAAAGCGGCA ATTCGGCAAT CGTTTTGCCC AGAAAGTATG TGTCACAGCG

9651 ATAAAGTCGA CTTCGGGCCT CCCTCATAAA AACTGGCAGC TCTGAGGTGA

9701 ACACCTAAAT CGAATCGATT CATTAGAAAG TTAGTAAATT ATTGAAATGC

9751 AAATGTATTC TAAACATGAC TTACATTTAT CGTGGCAAAG ACGTTTTGAA

9801 AGGTCATGTT GGTCAGGAAG AGGAAGATGG CTCCGTTGAT ATTCATCACA

9851 CCCACTTGCG TGAGTTGTTG GCCCAAAAAG ATGAGGCCAA TCAAGATGGC

9901 AACCATCTGC AAATTAAAAT GTTACTCGCA TCTCATTAAT ATTCGCGAGT

9951 TAAATGAAAT TTATTTATCT TCTGCAAAAC TATAAACTAT ACATCTCATT

10001 GAAAAAAACT AAGAAGGGTG TGGAATCAGG CAATTCTATC TAAAATCTAG

10051 CGAATTTGTT TCCAAGAATT GTAAGCGTTA TATCATTTGT TTCCACTGGA

10101 ACCACTCACC GTTGTCTGAA TAAGTCGCAC TTTTACGAGG AGTGGTTCCT

10151 TGAGCACCGA CAGCCAGGAT CGCCACAGGA CCGCCCGGAA CTGCATGAAC

10201 CAGGTGGCCT TGTAGGTGTA CCCATTCTCC GGCTGCTCCA GTGGCTTCTC

10251 CAGATTTTTG GTGGCCAACA ACTGCTCCAT ATCCCGGGCT ACTTTGCTAA

10301 TGGCAAAATT GTCGCATATC TTGGCGATCC GATCACGGGA CTCGATCTCC

10351 CGTCCGGGCA CAACGGCCAA CACCTGTACG TAAAAGTCCG CCGGATTGTA

10401 GTTGGTAGGA CACTGGGCAC CCACGCTGGA TAGGAGTTGA GATGTTATGT

10451 AATACTAGAT ACCCTTAATA AACACATCGA ACTCACTAGG AAAAGAAGTC

10501 GACGGCTTCG CTGGGAGTGC CCAAGAAAGC TACCCTGCCC TCGGCCATCA

10551 GAAGGATCTT GTCAAAGAGC TCAAACAGCT CGGAAGACGG CTGATGAATG

10601 GTCAGGATGA CGGTCTTGCC CTTCTGCGAC AGCTTCTTCA GCACCTGGAC

10651 GACGCTGTGG GCGGTAAAGG AGTCCAGTCC GGAGGTGGGC TCATCGCAGA

10701 TCAGAAGCGG CGGATCGGTT AGAGCCTCGG AGGCGAATGC CAGACGCTTC

10751 CTTTCTCCGC CGGACAGACC TTTCACCCTG CCGGGCACAC CGATGATCGT

10801 GTGCTGACAT TTGCTGAGCG AAAGCTCCTG GATCACCTGA TCCACGCGGG

10851 CCACTCGCTG CCGATAGGTC AGATGTCGTG GCATCCGCAC CATGGCTTGG

10901 AAAATCAGGT GTTCCCTGGC CGTTAGGGAG CCGATAAAGA GGTCATCCTG

10951 CTGGACATAG GCGCACCTGG CCTGCATCTC CTTGGCGTCC ACAGGTTGGC

11001 CATTGAGCAG TCGCATCCCG GATGGCGATA CTTGGATGCC CTGCGGCGAT

11051 CGAAAGGCAA GGGCATTCAG CAGGGTCGTC TTTCCGGCAC CGGAACTGCC

11101 CATCACGGCC AAAAGTTCGC CCGGATAGGC CACGCCGCAA ACTGAGTTTC

11151 AAATTGGTAA TTGGACCCTT TATTAAGATT TCACACAGAT CAGCCGACTG

11201 CGAATAGAAA CTCACCGTTC TTGAGCAAAT GTTTCCTGGG CGCCGGTATG

11251 TGTCGCTCGT TGCAGAATAG TCCGCGTGTC CGGTTGACCA GCTGCCGCCA

11301 TCCGGAGCCC GGCTGATTGA CCGCCCCAAA GATGTCCATA TTGTGCCAGG

11351 CATAGGTGAG GTTCTCGGCT AGTTGGCCGC TCCCTGAACC GGAGTCCTCC

11401 GGCGGACTGG GTGGCCGGAG CGTGCCGTAG TTTTTGGCCT GCCCGAAGCC

11451 CTGGTTAATG CAGCTCTGCG AAGCCGCTCC GCTGTCACCC TGCAATGATA

11501 GGGGATCTCA AATATCAACT ACTAGCGTTA TGCTCATCTA ACCCCGAACA

11551 AAAAGTACCC CGAAGTATCC TACGAAGTAG GTTTATACTT TTATTTATTT

11601 TTTGTGCATC TAGGATCAGC TTAAAATATC TGGTTGTTAT ATTTTTTGTA

11651 AAAAAGAATA TAGTCGAAAA TGAATGCCTT TAGATGTCTT GATCATGATA

11701 TGATCTCAAA AATTGTCTTA TATAGCGAGA ACAGCTACCA GAATAATCTG

11751 TTTCGTGTCA CTATTTGTTT GTGCAATTGC GGTTTGGGAT TTTTGTGGGT

11801 CGCAGTTCTC ACGCCGCATA CAATTTGATG TTGCAATCGC AGTTCCTATA

11851 GATCAAGTGA ACTTAAGATG TATGCACATG TACTACTCAC ATTGTTCAGA

11901 TGCTCGGCAG ATGGGTGTTT GCTGCCTCCG CGAATTAATA GCTCCTGATC

11951 CTCTTGGCCC ATTGCCGGGA TTTTTCACAC TTTCCCCTGC TTACCCACCC

12001 AAAACCAATC ACCACCCCAA TCACTCAAAA AACAAACAAA AATAAGAAGC

12051 GAGAGGAGTT TTGGCACAGC ACTTTGTGTT TAATTGATGG CGTAAACCGC

12101 TTGGAGCTTC GTCACGAAAC CGCTGACAAA ATGCAACTGA AGGCGGACAT

12151 TGACGCTACG TAACGCTACA AACGGTGGCG AAAGAGATAG CGGACGCAGC

12201 GGCGAAAGAG ACGGCGATAT TTCTGTGGAC AGAGAAGGAG GCAAACAGCG

12251 CTGACTTTGA GTGGAATGTC ATTTTGAGTG AGAGGTAATC GAAAGAACCT

12301 GGTACATCAA ATACCCTTGG ATCGAAGTAA ATTTAAAACT GATCAGATAA

12351 GTTCAATGAT ATCCAGTGCA GTAAAAAAAA AAAATGTTTT TTTTATCTAC

12401 TTTCCGCAAA AATGGGTTTT ATTAACTTAC ATACATACTA GGCGCGCCCA

12451 TATGTTCGGC TTGTCGACAT GCCCGCCGTG ACCGTCGAGA ACCCGCTGAC

12501 GCTGCCCCGC GTATCCGCAC CCGCCGACGC CGTCGCACGT CCCGTGCTCA

12551 CCGTGACCAC CGCGCCCAGC GGTTTCGAGG GCGAGGGCTT CCCGGTGCGC

12601 CGCGCGTTCG CCGGGATCAA CTACCGCCAC CTCGACCCGT TCATCATGAT

12651 GGACCAGATG GGTGAGGTGG AGTACGCGCC CGGGGAGCCC AAGGGCACGC

12701 CCTGGCACCC GCACCGCGGC TTCGAGACCG TGACCTACAT CGTCGACGGT

12751 AACATGTGAG CAAAAGGCCA GCAAAAGGCC AGGAACCGTA AAAAGGCCGC

12801 GTTGCTGGCG TTTTTCCATA GGCTCCGCCC CCCTGACGAG CATCACAAAA

12851 ATCGACGCTC AAGTCAGAGG TGGCGAAACC CGACAGGACT ATAAAGATAC

12901 CAGGCGTTTC CCCCTGGAAG CTCCCTCGTG CGCTCTCCTG TTCCGACCCT

12951 GCCGCTTACC GGATACCTGT CCGCCTTTCT CCCTTCGGGA AGCGTGGCGC

13001 TTTCTCATAG CTCACGCTGT AGGTATCTCA GTTCGGTGTA GGTCGTTCGC

13051 TCCAAGCTGG GCTGTGTGCA CGAACCCCCC GTTCAGCCCG ACCGCTGCGC

13101 CTTATCCGGT AACTATCGTC TTGAGTCCAA CCCGGTAAGA CACGACTTAT

13151 CGCCACTGGC AGCAGCCACT GGTAACAGGA TTAGCAGAGC GAGGTATGTA

13201 GGCGGTGCTA CAGAGTTCTT GAAGTGGTGG CCTAACTACG GCTACACTAG

13251 AAGAACAGTA TTTGGTATCT GCGCTCTGCT GAAGCCAGTT ACCTTCGGAA

13301 AAAGAGTTGG TAGCTCTTGA TCCGGCAAAC AAACCACCGC TGGTAGCGGT

13351 GGTTTTTTTG TTTGCAAGCA GCAGATTACG CGCAGAAAAA AAGGATCTCA

13401 AGAAGATCCT TTGATCTTTT CTACGGGGTC TGACGCTCAG TGGAACGAAA

13451 ACTCACGTTA AGGGATTTTG GTCATGAGAT TATCAAAAAG GATCTTCACC

13501 TAGATCCTTT TAAATTAAAA ATGAAGTTTT AAATCAATCT AAAGTATATA

13551 TGAGTAAACT TGGTCTGACA GTTACCAATG CTTAATCAGT GAGGCACCTA

13601 TCTCAGCGAT CTGTCTATTT CGTTCATCCA TAGTTGCCTG ACTCCCCGTC

13651 GTGTAGATAA CTACGATACG GGAGGGCTTA CCATCTGGCC CCAGTGCTGC

13701 AATGATACCG CGAGACCCAC GCTCACCGGC TCCAGATTTA TCAGCAATAA

13751 ACCAGCCAGC CGGAAGGGCC GAGCGCAGAA GTGGTCCTGC AACTTTATCC

13801 GCCTCCATCC AGTCTATTAA TTGTTGCCGG GAAGCTAGAG TAAGTAGTTC

13851 GCCAGTTAAT AGTTTGCGCA ACGTTGTTGC CATTGCTACA GGCATCGTGG

13901 TGTCACGCTC GTCGTTTGGT ATGGCTTCAT TCAGCTCCGG TTCCCAACGA

13951 TCAAGGCGAG TTACATGATC CCCCATGTTG TGCAAAAAAG CGGTTAGCTC

14001 CTTCGGTCCT CCGATCGTTG TCAGAAGTAA GTTGGCCGCA GTGTTATCAC

14051 TCATGGTTAT GGCAGCACTG CATAATTCTC TTACTGTCAT GCCATCCGTA

14101 AGATGCTTTT CTGTGACTGG TGAGTACTCA ACCAAGTCAT TCTGAGAATA

14151 GTGTATGCGG CGACCGAGTT GCTCTTGCCC GGCGTCAATA CGGGATAATA

14201 CCGCGCCACA TAGCAGAACT TTAAAAGTGC TCATCATTGG AAAACGTTCT

14251 TCGGGGCGAA AACTCTCAAG GATCTTACCG CTGTTGAGAT CCAGTTCGAT

14301 GTAACCCACT CGTGCACCCA ACTGATCTTC AGCATCTTTT ACTTTCACCA

14351 GCGTTTCTGG GTGAGCAAAA ACAGGAAGGC AAAATGCCGC AAAAAAGGGA

14401 ATAAGGGCGA CACGGAAATG TTGAATACTC ATACTCTTCC TTTTTCAATA

14451 TTATTGAAGC ATTTATCAGG GTTATTGTCT CATGAGCGGA TACATATTTG

14501 AATGTATTTA GAAAAATAAA CAAATAGGGG TTCCGCGCAC ATTTCCCCGA

14551 AAAGTGCCAC CTGACGTC

**HJP-223-actin5C^Syn21Gal80^IVS-Syn21myr-tdTomato-p10w**

1 TAAGAAACCA TTATTATCAT GACATTAACC TATAAAAATA GGCGTATCAC

51 GAGGCCCTTT CGTCTTCAAG AATTCGTTTA TCACAAGTTT GTACAAAAAA

101 GCAGGCTCCG CGGCCGCAAT TCTATATTCT AAAAACACAA ATGATACTTC

151 TAAAAAAAAA TCATGAATGG CATCAACTCT GAATCAAATC TTTGCAGATG

201 CACCTACTTC TCATTTCCAC TGTCACATCA TTTTTCCAGA TCTCGCTGCC

251 TGTTATGTGG CCCACAAACC AAGACACGTT TTATGGCCAT TAAAGCTGGC

301 TGATCGTCGC CAAACACCAA ATACATAATG AATATGTACA CATTCGAGAA

351 AGAAGCGATC AAAGAAGCGT CTTCGGGCGG AGTAGGAGAA TGCGGAGGAG

401 AAGGAGAACG AGCTGATCTA GTATCTCTCC ACAATCCAAT GCCAACTGAC

451 CAACTGGCCA TATTCGGAGC AATTTGAAGC CAATTTCCAT CGCCTGGCGA

501 TCGCTCCATT CTTGGCTATA TGTTTTTCAC CGTTACCCGG GGCCATTTTC

551 AAAGACTCGT CGGCAAGATA AGATTGTGTC ACTCGCTGTC TCTCTTCATT

601 TGTCGAAGAA TGCTGAGGAA TTTCGCGATG ACGTCGGCGA GTATTTTGAA

651 GAATGAGAAT AATTTGTATT TATACGAAAA TCAGTTAGTG GAATTTTCTA

701 CAAAAACATG TTATCTATAG ATAATTTTGT TGCAAAATAT GTTGACTATG

751 ACAAAGATTG TATGTATATA CCTTTAATGT ATTCTCATTT TCTTATGTAT

801 TTATAATGGC AATGATGATA CTGATGATAT TTTAAGATGA TGCCAGACCA

851 AAAGGCTTGA ATTTCTGCGT CTTTTGCCGA ACGCAGTGCA TGTGCAATTG

901 TTGTTTTTTG GAATATTCAA TTTTCGGACT GTCCGCTTTG ATTTCAGTTT

951 CTTGGCTTAT TCAAAAAGCA AAGTAAAGCC AAAAAAGCGA GATGGCAATA

1001 CCAAATGCGG CAAAACGGTA GTGGAAGGAA AGGGGTGCGG GGCAGCGGAA

1051 GGAAGGGTGG GGCGGGGCGT GGCGGGGTCT GTGGCTGGGC GCGACGTCAC

1101 CGACGTTGGA GCCACTCCTT TGACCATGTG TGCGTGTGTG TATTATTCGT

1151 GTCTCGCCAC TCGCCGGTTG TTTTTTTCTT TTTATGCTGC GCTCTCTCTA

1201 GCGCCATCTC GCTTACGCAT GCTCAACGCA CCGCATGTTG CCGTTTCCTT

1251 TTATGCGTCA TTTTGGCTCG AAATAGGCAA TTATTTAAAC AAAGATTAGT

1301 CAACGAAAAC GCTAAAATAA ATAAGTCTAC AATATGGTTA CTTATTGCCA

1351 TGTGTGTGCA GCCAACGATA GCAACAAAAG CAACAACACA GGTGGCTTTC

1401 CCTCTTTCAC TTTTTGTTTG CAAGCCGCGT GCGAGCAAGA CGGCACGACC

1451 GGCAAACGCA ATTACGCTGA CAAAGAGCAG ACGAAGTTTT GGCGAAAAAC

1501 ATCAAGGCGC CTGATACGAA TGCATTTGCA ATAACAATTG CGATATTTAA

1551 TATTGTTTAT GAAGCTGTTT GACTTCAAAA CACACAAAAA AAAAAATAAA

1601 ACAAATTATT TGAAAGAGAA TTAGGAATCG GACGCTTATC GTTAGGGTAA

1651 CAACAAGAAA TGCTTACTGA GTCACAGCCT CTGGAAAACT GCCGCAAGCC

1701 AGAGAGAGAG AGAAAAAGAG GGAGAGCAGC TTAGACCGCA TGTGCTTGTG

1751 TGTGAGGCGT CTCTCTCTTC GTCTCTGTTG CGCAAACGCA TAGACTGCAC

1801 TGAAAAAATC GATTACCTAT TTTTTATGAA TGAATATTTG CACTATTACT

1851 ATTCAAAACT ATTAAGATAG CAATCACATT CAATAGCCAA ATACTATACC

1901 ACCTGAGCGA TGCAACGAAA TGATCAATTT GAGCAAAAAT GCTGCATATT

1951 TAGGACGGCA TCATTATAGA AATGCTTCTT GCTGTGTACT TTTCTCTCGT

2001 CTGGCAGCTG TTTCGCCGTT ATTGTTAAAA CCGGCTTAAG TTAGGTGTGT

2051 TTTCTACGAC TAGTGAATGC CCTACTAGAA GATGTGTGTT GCACAAAATG

2101 TCCCTGGAAT AACCAATTTG AAGTGCAGAT AGCAGTAAAC GTAAGCTAAT

2151 ATGAATATTA TTTAACTGTA ATGTTTTAAT ATCGCTGGAC ATTACTAATA

2201 AACCCACTAT AAACACATGT ACATATGTAT GTTTTGGCAT ACAATGAGTA

2251 GTTGGGGAAA AAATGTGTAA AAGCACCGTG ACCATCACAG CATAAAGATA

2301 ACCAGCTGAA GTATCGAATA TGAGTAACCC CCAAATTGAA TCACATGCCG

2351 CAACTGATAG GACCCATGGA AGTACACTCT TCATGGCGAT ATACAAGACA

2401 CACACAAGCA CGAACACCCA GTTGCGGAGG AAATTCTCCG TAAATGAAAA

2451 CCCAATCGGC GAACAATTCA TACCCATATA TGGTAAAAGT TTTGAACGCG

2501 ACTTGAGAGC GGAGAGCATT GCGGCTGATA AGGTTTTAGC GCTAAGCGGG

2551 CTTTATAAAA CGGGCTGCGG GACCAGTTTT CATATCACTA CCGTTTGAGT

2601 TCTTGTGCTG TGTGGATACT CCTCCCGACA CAAAGCCGCT CCATCAGCCA

2651 GCAGTCGTCT AATCCAGAGA CACCAAACCG AAAGACTTAA TTTATATTTA

2701 TTTAATTAAT TTTAATAAAA CACACCAAAT GTAAGTAGCT TTCCCCTTCC

2751 CAACAACAAA ACACCATCGA ACCACTCCCA CCAAGAAAAA GCAATAATCG

2801 AGAAAAGCCG CGGAAAATGT GTGATTTTTT TTGTAAACAA AACATTTTTT

2851 TATGTGCCAG TGCTGAAAGT GATCAAAAAA TACTAGCCAC GAGCTAAAGA

2901 GTTATTGTAT TGACCAAAAC TCCAAAAATA CCCAAGTTTG GCCCTAAATT

2951 GTCAATCAAA ATACCAATAG GTCGAAAGAC ATCAAAATTA ACAAAACCAG

3001 GGTTTCAAAT ACCATAACTC AAGAATCAGG ATTACAACTG CAGATTTCAG

3051 GATATATACA TACAAATTAT ACGAAATTAT AAAAACCAAA GCAATTCAAT

3101 AGCCCCAACT CAAATGTTAG GATCTAATAT AGTGTTTAAA GCCAAGCTCG

3151 CTGATGTGGG CGTGTCACGA TTTCACCCAA AGATATGCCA AATTACGAAT

3201 TGCAAATCAA TTCGCCAACA CTTTCTTTTT TTCCCACGCC CTAAAACACC

3251 AGATCATCCA TAAATGTACA TACATACAGT ATATGCATAT TATAATCTGT

3301 AAAACTAGAT CAGGTTCTTG AAAATAGTGA CGTAGGCAGC CGTTTTGGCT

3351 GAAGCAGAAA TTTTTGCCGG TTTTTCAAAG TTGTAGTTGC AAAAATGGAG

3401 AAAACCTTCG AGCATTCGTT CATATACACA CACTCACGCG CAAAATAACG

3451 AGAGAGAGTG TATGTGTGTG TGAGAGAGCG AAAGCCAGAC GACGGTTTGC

3501 TTTTCGCCTC GAAACATGAC CATATATGGT CACAAAACTT GGCCGCCGCA

3551 ATTCAACACA CCAGCGCTCT CCTTCGCACC CATAGCGACC ATGGCGCGGA

3601 GCGAGCGAGA TGGCGAGAGC GAGCGACGCC TATGGCGACG TCGACGCAGG

3651 CAGCGATTGA AAAACGCAGT TAACTGGCAT TCAACATTCA CCAGCCACTT

3701 TCAGTCGGTT TATTCCAGTC ATTCCTTTCA AACCGTGCGG TCGCTTAGCT

3751 CAGCCTCGCC ACTTGCGTTT ACAGTAGTTT TCACGCCTTG AATTTGTTAA

3801 ATCGAACAAA AAGGTAAAGT TTAACTAGCT TTGAAAAGTT TCGTGGCTCT

3851 TAATTGTTAA ATTTTCTAGA GTGCGTTTAG TGTTTTTTTT TTTTTTTATT

3901 TTGTAATGTT AATTTCGGGT TCCAATTCGA GTTTTAGGCA GCCGCCATTT

3951 TAAGGGCGCA TACACACAGG CAACTGTGCT CTCTTTGCGG CTTTCTTTTG

4001 CACCGGCATT CGTTAAGTGT CGTCTAGAAG CTTCTCCCCT CCCTTTTCGG

4051 CATATTCGTA TTGTGGTTTT AATTTTTCGG GGCGGGGCCT TCTATTTTGT

4101 AACTGTTCTT TTAATTTCTT ATTACAATTC GATCGCAAGT GAAAATCAGT

4151 TTTCAATCGG AAAAGTATTT TTTTATGAAA TTTTTTTTTG TCCAAGATTA

4201 AAATTTTGTA CTAAAAAAAC GTACATTGCA TTGAGTGATT TTTAATTGTA

4251 CACGAAAAAC AAGTTAGTTT GTTATGACAA TTGTACTTTG GTAGACCAGC

4301 GCAGTCCAAG GAAACCACGC AAATTCTCAG TTTTTTTTTT GCCATTTCTA

4351 CATTACCAAA TAAGGTAACC AAAAACTAAT GGGAAATCCG CATTCTTTCC

4401 ATTGCAGCTT ACAGGATCGA TCCCCGGGTA CCGCGCCGAC CCAGCTTTCT

4451 TGTACAAAGT GGTGATAAAC GGCCGGCATA ACTTCGTATA ATGTATGCTA

4501 TACGAAGTTA TCCTGCAGGA ACTTAAAAAA AAAAATCAAA ATGGATTACA

4551 ACAAAAGGAG TAGTGTGAGT ACGGTGCCGA ATGCTGCTCC CATTCGCGTG

4601 GGCTTCGTGG GATTGAACGC GGCTAAGGGT TGGGCCATTA AAACGCATTA

4651 TCCAGCCATA CTGCAGCTGA GCTCCCAGTT CCAAATAACA GCGCTTTATT

4701 CCCCCAAGAT CGAGACGTCC ATTGCGACAA TTCAGAGGCT GAAGTTGTCC

4751 AACGCCACAG CTTTCCCAAC ACTCGAGAGC TTCGCCTCGA GCTCCACGAT

4801 CGATATGATT GTGATCGCAA TCCAGGTGGC TTCCCACTAC GAGGTGGTAA

4851 TGCCCCTGCT CGAGTTTTCG AAGAACAATC CTAATCTGAA GTATTTGTTT

4901 GTGGAATGGG CACTGGCCTG CTCGCTCGAT CAGGCGGAGT CGATCTATAA

4951 GGCCGCTGCA GAGCGGGGCG TGCAAACAAT AATCAGTCTG CAGGGACGAA

5001 AGAGCCCGTA CATTTTGCGG GCAAAAGAGC TTATCTCCCA GGGCTACATC

5051 GGCGATATAA ATAGCATCGA GATTGCAGGT AACGGAGGTT GGTACGGTTA

5101 CGAGCGGCCG GTGAAAAGCC CGAAATACAT TTACGAGATC GGAAACGGAG

5151 TTGATCTGGT GACCACCACG TTCGGTCACA CGATAGATAT ATTGCAGTAC

5201 ATGACCAGTT CGTACTTCAG CCGTATCAAT GCCATGGTGT TTAACAACAT

5251 TCCAGAACAG GAGCTCATTG ATGAGAGGGG CAATCGCCTG GGCCAGCGGG

5301 TCCCAAAGAC TGTCCCAGAT CATCTCTTGT TCCAGGGCAC GCTGCTCAAC

5351 GGAAATGTTC CTGTGTCCTG TTCCTTTAAG GGAGGCAAGC CTACGAAAAA

5401 GTTCACCAAG AATCTGGTCA TAGATATCCA TGGTACAAAG GGTGATCTGA

5451 AGCTGGAGGG TGACGCTGGA TTTGCGGAAA TCTCGAATCT CGTTCTGTAT

5501 TATTCGGGCA CCCGCGCCAA TGATTTTCCC CTGGCCAATG GTCAGCAGGC

5551 ACCCTTGGAC CCCGGCTACG ACGCAGGCAA GGAGATTATG GAGGTGTATC

5601 ACCTCCGAAA CTACAACGCG ATCGTTGGTA ATATACACCG GCTCTACCAG

5651 AGTATCAGCG ATTTCCACTT CAACACCAAG AAAATCCCCG AGCTTCCATC

5701 GCAGTTCGTA ATGCAAGGCT TCGATTTCGA AGGCTTTCCC ACCCTGATGG

5751 ATGCCTTGAT CTTGCATCGC CTGATTGAAA GTGTCTACAA GTCGAATATG

5801 ATGGGCAGTA CCCTGAATGT TTCCAACATC TCCCACTACT CGCTTTAAAC

5851 GCGTAGGATC TTTGTGAAGG AACCTTACTT CTGTGGTGTG ACATAATTGG

5901 ACAAACTACC TACAGAGATT TAAAGCTCTA AGGTAAATAT AAAATTTTTA

5951 AGTGTATAAT GTGTTAAACT ACTGATTCTA ATTGTTTGTG TATTTTAGAT

6001 TCCAACCTAT GGAACTGATG AATGGGAGCA GTGGTGGAAT GCCTTTAATG

6051 AGGAAAACCT GTTTTGCTCA GAAGAAATGC CATCTAGTGA TGATGAGGCT

6101 ACTGCTGACT CTCAACATTC TACTCCTCCA AAAAAGAAGA GAAAGGTAGA

6151 AGACCCCAAG GACTTTCCTT CAGAATTGCT AAGTTTTTTG AGTCATGCTG

6201 TGTTTAGTAA TAGAACTCTT GCTTGCTTTG CTATTTACAC CACAAAGGAA

6251 AAAGCTGCAC TGCTATACAA GAAAATTATG GAAAAATATT TGATGTATAG

6301 TGCCTTGACT AGAGATCATA ATCAGCCATA CCACATTTGT AGAGGTTTTA

6351 CTTGCTTTAA AAAACCTCCC ACACCTCCCC CTGAACCTGA AACATAAAAT

6401 GAATGCAATT GTTGTTGTTA ACTTGTTTAT TGCAGCTTAT AATGGTTACA

6451 AATAAAGCAA TAGCATCACA AATTTCACAA ATAAAGCATT TTTTTCACTG

6501 CATTCTAGTT GTGGTTTGTC CAAACTCATC AATGTATCTT ATCATGTCTG

6551 GATCCCTAGG ATAACTTCGT ATAATGTATG CTATACGAAG TTATGGTACC

6601 AAAAGGTAGG TTCAACCACT GATGCCTAGG CACACCGAAA CGACTAACCC

6651 TAATTCTTAT CCTTTACTTC AGGAGGCCTA CCGGTGCTAG AACTTAAAAA

6701 AAAAAATCAA AATGGGCAAC AAATGCTGCA GCAAGCGACA GGATCAGGAA

6751 CTGGCACTGG CCTATCCCAC TGGGGGCTAC AAGAAATCCG ACTACACCTT

6801 TGGCCAGACG CACATCAACA GCAGCGGCGG CGGCAACATG GGCGGCGTTC

6851 TTGGCCAGAA GCATAACAAC GGTGGCTCGC TGGACTCGCG CTACACGCCC

6901 GATCCCAATC ATCGGGGTCC GTTGAAAATC GGCGGAAAGG GCGGCGTTGA

6951 CATCATCAGA CCACGCGGAT CCATGGTGTC GAAAGGCGAG GAGGTTATCA

7001 AGGAGTTTAT GCGATTTAAG GTCCGAATGG AGGGCAGTAT GAATGGTCAT

7051 GAATTTGAGA TTGAGGGTGA AGGAGAGGGC CGACCGTACG AAGGTACGCA

7101 GACGGCGAAG TTGAAGGTCA CGAAAGGAGG ACCTCTGCCA TTCGCCTGGG

7151 ATATCCTGTC CCCTCAGTTT ATGTACGGAA GCAAGGCATA CGTGAAGCAC

7201 CCCGCCGATA TTCCAGATTA TAAGAAACTC TCCTTCCCCG AGGGATTCAA

7251 GTGGGAGCGC GTTATGAACT TTGAGGATGG AGGACTTGTC ACGGTCACTC

7301 AGGACAGCAG TCTGCAGGAC GGAACCCTGA TCTACAAGGT TAAGATGCGA

7351 GGTACGAACT TCCCACCGGA TGGTCCGGTG ATGCAGAAAA AGACGATGGG

7401 ATGGGAGGCG AGCACCGAGC GACTGTACCC GCGTGATGGA GTCCTCAAGG

7451 GCGAAATTCA TCAGGCCCTG AAATTGAAAG ATGGTGGCCA CTATTTGGTG

7501 GAGTTCAAGA CTATCTACAT GGCAAAGAAG CCCGTACAGC TGCCGGGCTA

7551 TTATTACGTT GACACGAAGC TCGATATCAC GAGTCATAAC GAGGACTACA

7601 CAATCGTTGA GCAATACGAG CGCAGCGAGG GTCGCCACCA CCTTTTCCTG

7651 GGACATGGCA CTGGCTCGAC CGGCTCGGGC AGCAGCGGTA CAGCAAGCAG

7701 TGAGGACAAC AACATGGCCG TGATTAAGGA ATTTATGAGG TTCAAGGTAC

7751 GTATGGAGGG TTCCATGAAC GGCCATGAGT TCGAGATTGA GGGAGAGGGT

7801 GAAGGCCGAC CGTACGAAGG CACGCAGACT GCGAAACTGA AGGTGACAAA

7851 GGGTGGCCCG CTGCCATTTG CCTGGGATAT TCTGAGCCCT CAGTTCATGT

7901 ACGGTAGCAA GGCATACGTC AAACACCCTG CAGATATCCC CGATTACAAG

7951 AAGTTGTCCT TTCCCGAGGG TTTTAAGTGG GAGCGGGTGA TGAACTTCGA

8001 AGACGGAGGT CTTGTCACGG TGACACAGGA TAGTTCCCTG CAGGACGGCA

8051 CTCTGATCTA CAAAGTCAAG ATGCGAGGCA CCAATTTTCC ACCGGATGGA

8101 CCAGTGATGC AAAAGAAGAC CATGGGTTGG GAAGCTAGTA CGGAACGCCT

8151 GTATCCGAGG GATGGTGTGC TCAAGGGCGA GATCCACCAG GCATTGAAAC

8201 TTAAGGACGG TGGACATTAC TTGGTCGAGT TCAAAACTAT TTACATGGCT

8251 AAGAAACCCG TCCAGCTGCC CGGCTACTAT TATGTCGATA CCAAGCTGGA

8301 CATCACCTCC CACAATGAAG ACTACACCAT CGTGGAGCAG TACGAACGCA

8351 GTGAGGGCCG CCACCACTTG TTCCTGTACG GTATGGACGA GCTTTACAAG

8401 TAAAAACATG AATCGTTTTT AAAATAACAA ATCAATTGTT TTATAATATT

8451 CGTACGATTC TTTGATTATG TAATAAAATG TGATCATTAG GAAGATTACG

8501 AAAAATATAA AAAATATGAG TTCTGTGTGT ATAACAAATG CTGTAAACGC

8551 CACAATTGTG TTTGTTGCAA ATAAACCCAT GATTATTTGA TTAAAATTGT

8601 TGTTTTCTTT GTTCATAGAC AATAGTGTGT TTTGCCTAAA CGTGTACTGC

8651 ATAAACTCCA TGCGAGTGTA TAGCGAGCTA GTGGCTAACG CTTGCCCCAC

8701 CAAAGTAGAT TCGTCAAAAT CCTCAATTTC ATCACCCTCC TCCAAGTTTA

8751 ACATTTGGCC GTCGGAATTA ACTTCTAAAG ATGCCACATA ATCTAATAAA

8801 TGAAATAGAG ATTCAAACGT GGCGTCATCG TCCGTTTCGA CCATTTCCGA

8851 AAAGAACTCG GGCATAAACT CTATGATTTC TCTGGACGTG GTGTTGTCGA

8901 AACTCTCAAA GTACGCAGTC AGGAACGTGC GCGACATGTC GTCGGGAAAC

8951 TCGCGCGGAA ACATGTTGTT GTAACCGAAC GGGTCCCATA GCGCCAAAAC

9001 CAAATCTGCC AGCGTCAATA GAATGAGCAC GATGCCGACA ATGGAGCTGG

9051 CTTGGATAGC GATTCGAGTT AACTCTAGAG CGGCCGCTGG CCACGGGTGC

9101 GCATGATCGT GCTCCTGTCG TTGAGGACCC GGCTAGGCTG GCGGGGTTGC

9151 CTTACTGGTT AGCAGAATGA ATCACCGATA CGCGAGCGAA CGTGAAGCGA

9201 CTGCTGCTGC AAAACGTCTG CGACCTGAGC AACAACATGA ATGGTCTTCG

9251 GTTTCCGTGT TTCGTAAAGT CTGGAAACGC GGAAGTCAGC GCCCTGCACC

9301 ATTATGTTCC GGAGGCGCGC CCTAGTTCCA GTGAAATCCA AGCATTTTCT

9351 AAATTAAATG TATTCTTATT ATTATAGTTG TTATTTTTGA TATATATAAA

9401 CAACACTATT ATGCCCACCA TTTTTTTGAG ATGCATCTAC ACAAGGAACA

9451 AACACTGGAT GTCACTTTCA GTTCAAATTG TAACGCTAAT CACTCCGAAC

9501 AGGTCACAAA AAATTACCTT AAAAAGTCAT AATATTAAAT TAGAATAAAT

9551 ATAGCTGTGA GGGAAATATA TACAAATATA TTGGAGCAAA TAAATTGTAC

9601 ATACAAATAT TTATTACTAA TTTCTATTGA GACGAAATGA ACCACTCGGA

9651 ACCATTTGAG CGAACCGAAT CGCGCGGAAC TAACGACAGT CGCTCCAAGG

9701 TCGTCGAACA AAAGGTGAAT GTGTTGCGGA GAGCGGGTGG GAGACAGCGA

9751 AAGAGCAACT ACGAAACGTG GTGTGGTGGA GGTGAATTAT GAAGAGGGCG

9801 CGCGATTTGA AAAGTATGTA TATAAAAAAT ATATCCCGGT GTTTTATGTA

9851 GCGATAAACG AGTTTTTGAT GTAAGGTATG CAGGTGTGTA AGTCTTTTGG

9901 TTAGAAGACA AATCCAAAGT CTACTTGTGG GGATGTTCGA AGGGGAAATA

9951 CTTGTATTCT ATAGGTCATA TCTTGTTTTT ATTGGCACAA ATATAATTAC

10001 ATTAGCTTTT TGAGGGGGCA ATAAACAGTA AACACGATGG TAATAATGGT

10051 AAAAAAAAAA AACAAGCAGT TATTTCGGAT ATATGTCGGC TACTCCTTGC

10101 GTCGGGCCCG AAGTCTTAGA GCCAGATATG CGAGCACCCG GAAGCTCACG

10151 ATGAGAATGG CCAGACCCAC GTAGTCCAGC GGCAGATCGG CGGCGGAGAA

10201 GTTAAGCGTC TCCAGGATGA CCTTGCCCGA ACTGGGGCAC GTGGTGTTCG

10251 ACGATGTGCA GCTAATTTCG CCCGGCTCCA CGTCCGCCCA TTGGTTAATC

10301 AGCAGACCCT CGTTGGCGTA ACGGAACCAT GAGAGGTACG ACAACCATTT

10351 GAGGTATACT GGCACCGAGC CCGAGTTCAA GAAGAAGCCG CCAAAGAGCA

10401 GGAATGGTAT GATAACCGGC GGACCCACAG ACAGCGCCAT CGAGGTCGAG

10451 GAGCTGGCGC AGGATATTAG ATATCCGAAG GACGTTGACA CATTGGCCAC

10501 CAGAGTGACC AGCGCCAGGC AGTTGAAGAA GTGCAGCACT CCGGCCCGCA

10551 GTCCGATCAT CGGATAGGCA ATCGCCGTGA AGACCAGTGG CACTGTGAGA

10601 AAAAGCGGCA ATTCGGCAAT CGTTTTGCCC AGAAAGTATG TGTCACAGCG

10651 ATAAAGTCGA CTTCGGGCCT CCCTCATAAA AACTGGCAGC TCTGAGGTGA

10701 ACACCTAAAT CGAATCGATT CATTAGAAAG TTAGTAAATT ATTGAAATGC

10751 AAATGTATTC TAAACATGAC TTACATTTAT CGTGGCAAAG ACGTTTTGAA

10801 AGGTCATGTT GGTCAGGAAG AGGAAGATGG CTCCGTTGAT ATTCATCACA

10851 CCCACTTGCG TGAGTTGTTG GCCCAAAAAG ATGAGGCCAA TCAAGATGGC

10901 AACCATCTGC AAATTAAAAT GTTACTCGCA TCTCATTAAT ATTCGCGAGT

10951 TAAATGAAAT TTATTTATCT TCTGCAAAAC TATAAACTAT ACATCTCATT

11001 GAAAAAAACT AAGAAGGGTG TGGAATCAGG CAATTCTATC TAAAATCTAG

11051 CGAATTTGTT TCCAAGAATT GTAAGCGTTA TATCATTTGT TTCCACTGGA

11101 ACCACTCACC GTTGTCTGAA TAAGTCGCAC TTTTACGAGG AGTGGTTCCT

11151 TGAGCACCGA CAGCCAGGAT CGCCACAGGA CCGCCCGGAA CTGCATGAAC

11201 CAGGTGGCCT TGTAGGTGTA CCCATTCTCC GGCTGCTCCA GTGGCTTCTC

11251 CAGATTTTTG GTGGCCAACA ACTGCTCCAT ATCCCGGGCT ACTTTGCTAA

11301 TGGCAAAATT GTCGCATATC TTGGCGATCC GATCACGGGA CTCGATCTCC

11351 CGTCCGGGCA CAACGGCCAA CACCTGTACG TAAAAGTCCG CCGGATTGTA

11401 GTTGGTAGGA CACTGGGCAC CCACGCTGGA TAGGAGTTGA GATGTTATGT

11451 AATACTAGAT ACCCTTAATA AACACATCGA ACTCACTAGG AAAAGAAGTC

11501 GACGGCTTCG CTGGGAGTGC CCAAGAAAGC TACCCTGCCC TCGGCCATCA

11551 GAAGGATCTT GTCAAAGAGC TCAAACAGCT CGGAAGACGG CTGATGAATG

11601 GTCAGGATGA CGGTCTTGCC CTTCTGCGAC AGCTTCTTCA GCACCTGGAC

11651 GACGCTGTGG GCGGTAAAGG AGTCCAGTCC GGAGGTGGGC TCATCGCAGA

11701 TCAGAAGCGG CGGATCGGTT AGAGCCTCGG AGGCGAATGC CAGACGCTTC

11751 CTTTCTCCGC CGGACAGACC TTTCACCCTG CCGGGCACAC CGATGATCGT

11801 GTGCTGACAT TTGCTGAGCG AAAGCTCCTG GATCACCTGA TCCACGCGGG

11851 CCACTCGCTG CCGATAGGTC AGATGTCGTG GCATCCGCAC CATGGCTTGG

11901 AAAATCAGGT GTTCCCTGGC CGTTAGGGAG CCGATAAAGA GGTCATCCTG

11951 CTGGACATAG GCGCACCTGG CCTGCATCTC CTTGGCGTCC ACAGGTTGGC

12001 CATTGAGCAG TCGCATCCCG GATGGCGATA CTTGGATGCC CTGCGGCGAT

12051 CGAAAGGCAA GGGCATTCAG CAGGGTCGTC TTTCCGGCAC CGGAACTGCC

12101 CATCACGGCC AAAAGTTCGC CCGGATAGGC CACGCCGCAA ACTGAGTTTC

12151 AAATTGGTAA TTGGACCCTT TATTAAGATT TCACACAGAT CAGCCGACTG

12201 CGAATAGAAA CTCACCGTTC TTGAGCAAAT GTTTCCTGGG CGCCGGTATG

12251 TGTCGCTCGT TGCAGAATAG TCCGCGTGTC CGGTTGACCA GCTGCCGCCA

12301 TCCGGAGCCC GGCTGATTGA CCGCCCCAAA GATGTCCATA TTGTGCCAGG

12351 CATAGGTGAG GTTCTCGGCT AGTTGGCCGC TCCCTGAACC GGAGTCCTCC

12401 GGCGGACTGG GTGGCCGGAG CGTGCCGTAG TTTTTGGCCT GCCCGAAGCC

12451 CTGGTTAATG CAGCTCTGCG AAGCCGCTCC GCTGTCACCC TGCAATGATA

12501 GGGGATCTCA AATATCAACT ACTAGCGTTA TGCTCATCTA ACCCCGAACA

12551 AAAAGTACCC CGAAGTATCC TACGAAGTAG GTTTATACTT TTATTTATTT

12601 TTTGTGCATC TAGGATCAGC TTAAAATATC TGGTTGTTAT ATTTTTTGTA

12651 AAAAAGAATA TAGTCGAAAA TGAATGCCTT TAGATGTCTT GATCATGATA

12701 TGATCTCAAA AATTGTCTTA TATAGCGAGA ACAGCTACCA GAATAATCTG

12751 TTTCGTGTCA CTATTTGTTT GTGCAATTGC GGTTTGGGAT TTTTGTGGGT

12801 CGCAGTTCTC ACGCCGCATA CAATTTGATG TTGCAATCGC AGTTCCTATA

12851 GATCAAGTGA ACTTAAGATG TATGCACATG TACTACTCAC ATTGTTCAGA

12901 TGCTCGGCAG ATGGGTGTTT GCTGCCTCCG CGAATTAATA GCTCCTGATC

12951 CTCTTGGCCC ATTGCCGGGA TTTTTCACAC TTTCCCCTGC TTACCCACCC

13001 AAAACCAATC ACCACCCCAA TCACTCAAAA AACAAACAAA AATAAGAAGC

13051 GAGAGGAGTT TTGGCACAGC ACTTTGTGTT TAATTGATGG CGTAAACCGC

13101 TTGGAGCTTC GTCACGAAAC CGCTGACAAA ATGCAACTGA AGGCGGACAT

13151 TGACGCTACG TAACGCTACA AACGGTGGCG AAAGAGATAG CGGACGCAGC

13201 GGCGAAAGAG ACGGCGATAT TTCTGTGGAC AGAGAAGGAG GCAAACAGCG

13251 CTGACTTTGA GTGGAATGTC ATTTTGAGTG AGAGGTAATC GAAAGAACCT

13301 GGTACATCAA ATACCCTTGG ATCGAAGTAA ATTTAAAACT GATCAGATAA

13351 GTTCAATGAT ATCCAGTGCA GTAAAAAAAA AAAATGTTTT TTTTATCTAC

13401 TTTCCGCAAA AATGGGTTTT ATTAACTTAC ATACATACTA GGCGCGCCCA

13451 TATGTTCGGC TTGTCGACAT GCCCGCCGTG ACCGTCGAGA ACCCGCTGAC

13501 GCTGCCCCGC GTATCCGCAC CCGCCGACGC CGTCGCACGT CCCGTGCTCA

13551 CCGTGACCAC CGCGCCCAGC GGTTTCGAGG GCGAGGGCTT CCCGGTGCGC

13601 CGCGCGTTCG CCGGGATCAA CTACCGCCAC CTCGACCCGT TCATCATGAT

13651 GGACCAGATG GGTGAGGTGG AGTACGCGCC CGGGGAGCCC AAGGGCACGC

13701 CCTGGCACCC GCACCGCGGC TTCGAGACCG TGACCTACAT CGTCGACGGT

13751 AACATGTGAG CAAAAGGCCA GCAAAAGGCC AGGAACCGTA AAAAGGCCGC

13801 GTTGCTGGCG TTTTTCCATA GGCTCCGCCC CCCTGACGAG CATCACAAAA

13851 ATCGACGCTC AAGTCAGAGG TGGCGAAACC CGACAGGACT ATAAAGATAC

13901 CAGGCGTTTC CCCCTGGAAG CTCCCTCGTG CGCTCTCCTG TTCCGACCCT

13951 GCCGCTTACC GGATACCTGT CCGCCTTTCT CCCTTCGGGA AGCGTGGCGC

14001 TTTCTCATAG CTCACGCTGT AGGTATCTCA GTTCGGTGTA GGTCGTTCGC

14051 TCCAAGCTGG GCTGTGTGCA CGAACCCCCC GTTCAGCCCG ACCGCTGCGC

14101 CTTATCCGGT AACTATCGTC TTGAGTCCAA CCCGGTAAGA CACGACTTAT

14151 CGCCACTGGC AGCAGCCACT GGTAACAGGA TTAGCAGAGC GAGGTATGTA

14201 GGCGGTGCTA CAGAGTTCTT GAAGTGGTGG CCTAACTACG GCTACACTAG

14251 AAGAACAGTA TTTGGTATCT GCGCTCTGCT GAAGCCAGTT ACCTTCGGAA

14301 AAAGAGTTGG TAGCTCTTGA TCCGGCAAAC AAACCACCGC TGGTAGCGGT

14351 GGTTTTTTTG TTTGCAAGCA GCAGATTACG CGCAGAAAAA AAGGATCTCA

14401 AGAAGATCCT TTGATCTTTT CTACGGGGTC TGACGCTCAG TGGAACGAAA

14451 ACTCACGTTA AGGGATTTTG GTCATGAGAT TATCAAAAAG GATCTTCACC

14501 TAGATCCTTT TAAATTAAAA ATGAAGTTTT AAATCAATCT AAAGTATATA

14551 TGAGTAAACT TGGTCTGACA GTTACCAATG CTTAATCAGT GAGGCACCTA

14601 TCTCAGCGAT CTGTCTATTT CGTTCATCCA TAGTTGCCTG ACTCCCCGTC

14651 GTGTAGATAA CTACGATACG GGAGGGCTTA CCATCTGGCC CCAGTGCTGC

14701 AATGATACCG CGAGACCCAC GCTCACCGGC TCCAGATTTA TCAGCAATAA

14751 ACCAGCCAGC CGGAAGGGCC GAGCGCAGAA GTGGTCCTGC AACTTTATCC

14801 GCCTCCATCC AGTCTATTAA TTGTTGCCGG GAAGCTAGAG TAAGTAGTTC

14851 GCCAGTTAAT AGTTTGCGCA ACGTTGTTGC CATTGCTACA GGCATCGTGG

14901 TGTCACGCTC GTCGTTTGGT ATGGCTTCAT TCAGCTCCGG TTCCCAACGA

14951 TCAAGGCGAG TTACATGATC CCCCATGTTG TGCAAAAAAG CGGTTAGCTC

15001 CTTCGGTCCT CCGATCGTTG TCAGAAGTAA GTTGGCCGCA GTGTTATCAC

15051 TCATGGTTAT GGCAGCACTG CATAATTCTC TTACTGTCAT GCCATCCGTA

15101 AGATGCTTTT CTGTGACTGG TGAGTACTCA ACCAAGTCAT TCTGAGAATA

15151 GTGTATGCGG CGACCGAGTT GCTCTTGCCC GGCGTCAATA CGGGATAATA

15201 CCGCGCCACA TAGCAGAACT TTAAAAGTGC TCATCATTGG AAAACGTTCT

15251 TCGGGGCGAA AACTCTCAAG GATCTTACCG CTGTTGAGAT CCAGTTCGAT

15301 GTAACCCACT CGTGCACCCA ACTGATCTTC AGCATCTTTT ACTTTCACCA

15351 GCGTTTCTGG GTGAGCAAAA ACAGGAAGGC AAAATGCCGC AAAAAAGGGA

15401 ATAAGGGCGA CACGGAAATG TTGAATACTC ATACTCTTCC TTTTTCAATA

15451 TTATTGAAGC ATTTATCAGG GTTATTGTCT CATGAGCGGA TACATATTTG

15501 AATGTATTTA GAAAAATAAA CAAATAGGGG TTCCGCGCAC ATTTCCCCGA

15551 AAAGTGCCAC CTGACGTC

**HJP-224-actin5C^Syn21Gal80^IVS-Syn21myr-tdTomato-10UAS-mCD8GFP-p10w**

1 TAAGAAACCA TTATTATCAT GACATTAACC TATAAAAATA GGCGTATCAC

51 GAGGCCCTTT CGTCTTCAAG AATTCGTTTA TCACAAGTTT GTACAAAAAA

101 GCAGGCTCCG CGGCCGCAAT TCTATATTCT AAAAACACAA ATGATACTTC

151 TAAAAAAAAA TCATGAATGG CATCAACTCT GAATCAAATC TTTGCAGATG

201 CACCTACTTC TCATTTCCAC TGTCACATCA TTTTTCCAGA TCTCGCTGCC

251 TGTTATGTGG CCCACAAACC AAGACACGTT TTATGGCCAT TAAAGCTGGC

301 TGATCGTCGC CAAACACCAA ATACATAATG AATATGTACA CATTCGAGAA

351 AGAAGCGATC AAAGAAGCGT CTTCGGGCGG AGTAGGAGAA TGCGGAGGAG

401 AAGGAGAACG AGCTGATCTA GTATCTCTCC ACAATCCAAT GCCAACTGAC

451 CAACTGGCCA TATTCGGAGC AATTTGAAGC CAATTTCCAT CGCCTGGCGA

501 TCGCTCCATT CTTGGCTATA TGTTTTTCAC CGTTACCCGG GGCCATTTTC

551 AAAGACTCGT CGGCAAGATA AGATTGTGTC ACTCGCTGTC TCTCTTCATT

601 TGTCGAAGAA TGCTGAGGAA TTTCGCGATG ACGTCGGCGA GTATTTTGAA

651 GAATGAGAAT AATTTGTATT TATACGAAAA TCAGTTAGTG GAATTTTCTA

701 CAAAAACATG TTATCTATAG ATAATTTTGT TGCAAAATAT GTTGACTATG

751 ACAAAGATTG TATGTATATA CCTTTAATGT ATTCTCATTT TCTTATGTAT

801 TTATAATGGC AATGATGATA CTGATGATAT TTTAAGATGA TGCCAGACCA

851 AAAGGCTTGA ATTTCTGCGT CTTTTGCCGA ACGCAGTGCA TGTGCAATTG

901 TTGTTTTTTG GAATATTCAA TTTTCGGACT GTCCGCTTTG ATTTCAGTTT

951 CTTGGCTTAT TCAAAAAGCA AAGTAAAGCC AAAAAAGCGA GATGGCAATA

1001 CCAAATGCGG CAAAACGGTA GTGGAAGGAA AGGGGTGCGG GGCAGCGGAA

1051 GGAAGGGTGG GGCGGGGCGT GGCGGGGTCT GTGGCTGGGC GCGACGTCAC

1101 CGACGTTGGA GCCACTCCTT TGACCATGTG TGCGTGTGTG TATTATTCGT

1151 GTCTCGCCAC TCGCCGGTTG TTTTTTTCTT TTTATGCTGC GCTCTCTCTA

1201 GCGCCATCTC GCTTACGCAT GCTCAACGCA CCGCATGTTG CCGTTTCCTT

1251 TTATGCGTCA TTTTGGCTCG AAATAGGCAA TTATTTAAAC AAAGATTAGT

1301 CAACGAAAAC GCTAAAATAA ATAAGTCTAC AATATGGTTA CTTATTGCCA

1351 TGTGTGTGCA GCCAACGATA GCAACAAAAG CAACAACACA GGTGGCTTTC

1401 CCTCTTTCAC TTTTTGTTTG CAAGCCGCGT GCGAGCAAGA CGGCACGACC

1451 GGCAAACGCA ATTACGCTGA CAAAGAGCAG ACGAAGTTTT GGCGAAAAAC

1501 ATCAAGGCGC CTGATACGAA TGCATTTGCA ATAACAATTG CGATATTTAA

1551 TATTGTTTAT GAAGCTGTTT GACTTCAAAA CACACAAAAA AAAAAATAAA

1601 ACAAATTATT TGAAAGAGAA TTAGGAATCG GACGCTTATC GTTAGGGTAA

1651 CAACAAGAAA TGCTTACTGA GTCACAGCCT CTGGAAAACT GCCGCAAGCC

1701 AGAGAGAGAG AGAAAAAGAG GGAGAGCAGC TTAGACCGCA TGTGCTTGTG

1751 TGTGAGGCGT CTCTCTCTTC GTCTCTGTTG CGCAAACGCA TAGACTGCAC

1801 TGAAAAAATC GATTACCTAT TTTTTATGAA TGAATATTTG CACTATTACT

1851 ATTCAAAACT ATTAAGATAG CAATCACATT CAATAGCCAA ATACTATACC

1901 ACCTGAGCGA TGCAACGAAA TGATCAATTT GAGCAAAAAT GCTGCATATT

1951 TAGGACGGCA TCATTATAGA AATGCTTCTT GCTGTGTACT TTTCTCTCGT

2001 CTGGCAGCTG TTTCGCCGTT ATTGTTAAAA CCGGCTTAAG TTAGGTGTGT

2051 TTTCTACGAC TAGTGAATGC CCTACTAGAA GATGTGTGTT GCACAAAATG

2101 TCCCTGGAAT AACCAATTTG AAGTGCAGAT AGCAGTAAAC GTAAGCTAAT

2151 ATGAATATTA TTTAACTGTA ATGTTTTAAT ATCGCTGGAC ATTACTAATA

2201 AACCCACTAT AAACACATGT ACATATGTAT GTTTTGGCAT ACAATGAGTA

2251 GTTGGGGAAA AAATGTGTAA AAGCACCGTG ACCATCACAG CATAAAGATA

2301 ACCAGCTGAA GTATCGAATA TGAGTAACCC CCAAATTGAA TCACATGCCG

2351 CAACTGATAG GACCCATGGA AGTACACTCT TCATGGCGAT ATACAAGACA

2401 CACACAAGCA CGAACACCCA GTTGCGGAGG AAATTCTCCG TAAATGAAAA

2451 CCCAATCGGC GAACAATTCA TACCCATATA TGGTAAAAGT TTTGAACGCG

2501 ACTTGAGAGC GGAGAGCATT GCGGCTGATA AGGTTTTAGC GCTAAGCGGG

2551 CTTTATAAAA CGGGCTGCGG GACCAGTTTT CATATCACTA CCGTTTGAGT

2601 TCTTGTGCTG TGTGGATACT CCTCCCGACA CAAAGCCGCT CCATCAGCCA

2651 GCAGTCGTCT AATCCAGAGA CACCAAACCG AAAGACTTAA TTTATATTTA

2701 TTTAATTAAT TTTAATAAAA CACACCAAAT GTAAGTAGCT TTCCCCTTCC

2751 CAACAACAAA ACACCATCGA ACCACTCCCA CCAAGAAAAA GCAATAATCG

2801 AGAAAAGCCG CGGAAAATGT GTGATTTTTT TTGTAAACAA AACATTTTTT

2851 TATGTGCCAG TGCTGAAAGT GATCAAAAAA TACTAGCCAC GAGCTAAAGA

2901 GTTATTGTAT TGACCAAAAC TCCAAAAATA CCCAAGTTTG GCCCTAAATT

2951 GTCAATCAAA ATACCAATAG GTCGAAAGAC ATCAAAATTA ACAAAACCAG

3001 GGTTTCAAAT ACCATAACTC AAGAATCAGG ATTACAACTG CAGATTTCAG

3051 GATATATACA TACAAATTAT ACGAAATTAT AAAAACCAAA GCAATTCAAT

3101 AGCCCCAACT CAAATGTTAG GATCTAATAT AGTGTTTAAA GCCAAGCTCG

3151 CTGATGTGGG CGTGTCACGA TTTCACCCAA AGATATGCCA AATTACGAAT

3201 TGCAAATCAA TTCGCCAACA CTTTCTTTTT TTCCCACGCC CTAAAACACC

3251 AGATCATCCA TAAATGTACA TACATACAGT ATATGCATAT TATAATCTGT

3301 AAAACTAGAT CAGGTTCTTG AAAATAGTGA CGTAGGCAGC CGTTTTGGCT

3351 GAAGCAGAAA TTTTTGCCGG TTTTTCAAAG TTGTAGTTGC AAAAATGGAG

3401 AAAACCTTCG AGCATTCGTT CATATACACA CACTCACGCG CAAAATAACG

3451 AGAGAGAGTG TATGTGTGTG TGAGAGAGCG AAAGCCAGAC GACGGTTTGC

3501 TTTTCGCCTC GAAACATGAC CATATATGGT CACAAAACTT GGCCGCCGCA

3551 ATTCAACACA CCAGCGCTCT CCTTCGCACC CATAGCGACC ATGGCGCGGA

3601 GCGAGCGAGA TGGCGAGAGC GAGCGACGCC TATGGCGACG TCGACGCAGG

3651 CAGCGATTGA AAAACGCAGT TAACTGGCAT TCAACATTCA CCAGCCACTT

3701 TCAGTCGGTT TATTCCAGTC ATTCCTTTCA AACCGTGCGG TCGCTTAGCT

3751 CAGCCTCGCC ACTTGCGTTT ACAGTAGTTT TCACGCCTTG AATTTGTTAA

3801 ATCGAACAAA AAGGTAAAGT TTAACTAGCT TTGAAAAGTT TCGTGGCTCT

3851 TAATTGTTAA ATTTTCTAGA GTGCGTTTAG TGTTTTTTTT TTTTTTTATT

3901 TTGTAATGTT AATTTCGGGT TCCAATTCGA GTTTTAGGCA GCCGCCATTT

3951 TAAGGGCGCA TACACACAGG CAACTGTGCT CTCTTTGCGG CTTTCTTTTG

4001 CACCGGCATT CGTTAAGTGT CGTCTAGAAG CTTCTCCCCT CCCTTTTCGG

4051 CATATTCGTA TTGTGGTTTT AATTTTTCGG GGCGGGGCCT TCTATTTTGT

4101 AACTGTTCTT TTAATTTCTT ATTACAATTC GATCGCAAGT GAAAATCAGT

4151 TTTCAATCGG AAAAGTATTT TTTTATGAAA TTTTTTTTTG TCCAAGATTA

4201 AAATTTTGTA CTAAAAAAAC GTACATTGCA TTGAGTGATT TTTAATTGTA

4251 CACGAAAAAC AAGTTAGTTT GTTATGACAA TTGTACTTTG GTAGACCAGC

4301 GCAGTCCAAG GAAACCACGC AAATTCTCAG TTTTTTTTTT GCCATTTCTA

4351 CATTACCAAA TAAGGTAACC AAAAACTAAT GGGAAATCCG CATTCTTTCC

4401 ATTGCAGCTT ACAGGATCGA TCCCCGGGTA CCGCGCCGAC CCAGCTTTCT

4451 TGTACAAAGT GGTGATAAAC GGCCGGCATA ACTTCGTATA ATGTATGCTA

4501 TACGAAGTTA TCCTGCAGGA ACTTAAAAAA AAAAATCAAA ATGGATTACA

4551 ACAAAAGGAG TAGTGTGAGT ACGGTGCCGA ATGCTGCTCC CATTCGCGTG

4601 GGCTTCGTGG GATTGAACGC GGCTAAGGGT TGGGCCATTA AAACGCATTA

4651 TCCAGCCATA CTGCAGCTGA GCTCCCAGTT CCAAATAACA GCGCTTTATT

4701 CCCCCAAGAT CGAGACGTCC ATTGCGACAA TTCAGAGGCT GAAGTTGTCC

4751 AACGCCACAG CTTTCCCAAC ACTCGAGAGC TTCGCCTCGA GCTCCACGAT

4801 CGATATGATT GTGATCGCAA TCCAGGTGGC TTCCCACTAC GAGGTGGTAA

4851 TGCCCCTGCT CGAGTTTTCG AAGAACAATC CTAATCTGAA GTATTTGTTT

4901 GTGGAATGGG CACTGGCCTG CTCGCTCGAT CAGGCGGAGT CGATCTATAA

4951 GGCCGCTGCA GAGCGGGGCG TGCAAACAAT AATCAGTCTG CAGGGACGAA

5001 AGAGCCCGTA CATTTTGCGG GCAAAAGAGC TTATCTCCCA GGGCTACATC

5051 GGCGATATAA ATAGCATCGA GATTGCAGGT AACGGAGGTT GGTACGGTTA

5101 CGAGCGGCCG GTGAAAAGCC CGAAATACAT TTACGAGATC GGAAACGGAG

5151 TTGATCTGGT GACCACCACG TTCGGTCACA CGATAGATAT ATTGCAGTAC

5201 ATGACCAGTT CGTACTTCAG CCGTATCAAT GCCATGGTGT TTAACAACAT

5251 TCCAGAACAG GAGCTCATTG ATGAGAGGGG CAATCGCCTG GGCCAGCGGG

5301 TCCCAAAGAC TGTCCCAGAT CATCTCTTGT TCCAGGGCAC GCTGCTCAAC

5351 GGAAATGTTC CTGTGTCCTG TTCCTTTAAG GGAGGCAAGC CTACGAAAAA

5401 GTTCACCAAG AATCTGGTCA TAGATATCCA TGGTACAAAG GGTGATCTGA

5451 AGCTGGAGGG TGACGCTGGA TTTGCGGAAA TCTCGAATCT CGTTCTGTAT

5501 TATTCGGGCA CCCGCGCCAA TGATTTTCCC CTGGCCAATG GTCAGCAGGC

5551 ACCCTTGGAC CCCGGCTACG ACGCAGGCAA GGAGATTATG GAGGTGTATC

5601 ACCTCCGAAA CTACAACGCG ATCGTTGGTA ATATACACCG GCTCTACCAG

5651 AGTATCAGCG ATTTCCACTT CAACACCAAG AAAATCCCCG AGCTTCCATC

5701 GCAGTTCGTA ATGCAAGGCT TCGATTTCGA AGGCTTTCCC ACCCTGATGG

5751 ATGCCTTGAT CTTGCATCGC CTGATTGAAA GTGTCTACAA GTCGAATATG

5801 ATGGGCAGTA CCCTGAATGT TTCCAACATC TCCCACTACT CGCTTTAAAC

5851 GCGTAGGATC TTTGTGAAGG AACCTTACTT CTGTGGTGTG ACATAATTGG

5901 ACAAACTACC TACAGAGATT TAAAGCTCTA AGGTAAATAT AAAATTTTTA

5951 AGTGTATAAT GTGTTAAACT ACTGATTCTA ATTGTTTGTG TATTTTAGAT

6001 TCCAACCTAT GGAACTGATG AATGGGAGCA GTGGTGGAAT GCCTTTAATG

6051 AGGAAAACCT GTTTTGCTCA GAAGAAATGC CATCTAGTGA TGATGAGGCT

6101 ACTGCTGACT CTCAACATTC TACTCCTCCA AAAAAGAAGA GAAAGGTAGA

6151 AGACCCCAAG GACTTTCCTT CAGAATTGCT AAGTTTTTTG AGTCATGCTG

6201 TGTTTAGTAA TAGAACTCTT GCTTGCTTTG CTATTTACAC CACAAAGGAA

6251 AAAGCTGCAC TGCTATACAA GAAAATTATG GAAAAATATT TGATGTATAG

6301 TGCCTTGACT AGAGATCATA ATCAGCCATA CCACATTTGT AGAGGTTTTA

6351 CTTGCTTTAA AAAACCTCCC ACACCTCCCC CTGAACCTGA AACATAAAAT

6401 GAATGCAATT GTTGTTGTTA ACTTGTTTAT TGCAGCTTAT AATGGTTACA

6451 AATAAAGCAA TAGCATCACA AATTTCACAA ATAAAGCATT TTTTTCACTG

6501 CATTCTAGTT GTGGTTTGTC CAAACTCATC AATGTATCTT ATCATGTCTG

6551 GATCCCTAGG ATAACTTCGT ATAATGTATG CTATACGAAG TTATGGTACC

6601 AAAAGGTAGG TTCAACCACT GATGCCTAGG CACACCGAAA CGACTAACCC

6651 TAATTCTTAT CCTTTACTTC AGGAACTTAA AAAAAAAAAT CAAAATGGGC

6701 AACAAATGCT GCAGCAAGCG ACAGGATCAG GAACTGGCAC TGGCCTATCC

6751 CACTGGGGGC TACAAGAAAT CCGACTACAC CTTTGGCCAG ACGCACATCA

6801 ACAGCAGCGG CGGCGGCAAC ATGGGCGGCG TTCTTGGCCA GAAGCATAAC

6851 AACGGTGGCT CGCTGGACTC GCGCTACACG CCCGATCCCA ATCATCGGGG

6901 TCCGTTGAAA ATCGGCGGAA AGGGCGGCGT TGACATCATC AGACCACGCG

6951 GATCCATGGT GTCGAAAGGC GAGGAGGTTA TCAAGGAGTT TATGCGATTT

7001 AAGGTCCGAA TGGAGGGCAG TATGAATGGT CATGAATTTG AGATTGAGGG

7051 TGAAGGAGAG GGCCGACCGT ACGAAGGTAC GCAGACGGCG AAGTTGAAGG

7101 TCACGAAAGG AGGACCTCTG CCATTCGCCT GGGATATCCT GTCCCCTCAG

7151 TTTATGTACG GAAGCAAGGC ATACGTGAAG CACCCCGCCG ATATTCCAGA

7201 TTATAAGAAA CTCTCCTTCC CCGAGGGATT CAAGTGGGAG CGCGTTATGA

7251 ACTTTGAGGA TGGAGGACTT GTCACGGTCA CTCAGGACAG CAGTCTGCAG

7301 GACGGAACCC TGATCTACAA GGTTAAGATG CGAGGTACGA ACTTCCCACC

7351 GGATGGTCCG GTGATGCAGA AAAAGACGAT GGGATGGGAG GCGAGCACCG

7401 AGCGACTGTA CCCGCGTGAT GGAGTCCTCA AGGGCGAAAT TCATCAGGCC

7451 CTGAAATTGA AAGATGGTGG CCACTATTTG GTGGAGTTCA AGACTATCTA

7501 CATGGCAAAG AAGCCCGTAC AGCTGCCGGG CTATTATTAC GTTGACACGA

7551 AGCTCGATAT CACGAGTCAT AACGAGGACT ACACAATCGT TGAGCAATAC

7601 GAGCGCAGCG AGGGTCGCCA CCACCTTTTC CTGGGACATG GCACTGGCTC

7651 GACCGGCTCG GGCAGCAGCG GTACAGCAAG CAGTGAGGAC AACAACATGG

7701 CCGTGATTAA GGAATTTATG AGGTTCAAGG TACGTATGGA GGGTTCCATG

7751 AACGGCCATG AGTTCGAGAT TGAGGGAGAG GGTGAAGGCC GACCGTACGA

7801 AGGCACGCAG ACTGCGAAAC TGAAGGTGAC AAAGGGTGGC CCGCTGCCAT

7851 TTGCCTGGGA TATTCTGAGC CCTCAGTTCA TGTACGGTAG CAAGGCATAC

7901 GTCAAACACC CTGCAGATAT CCCCGATTAC AAGAAGTTGT CCTTTCCCGA

7951 GGGTTTTAAG TGGGAGCGGG TGATGAACTT CGAAGACGGA GGTCTTGTCA

8001 CGGTGACACA GGATAGTTCC CTGCAGGACG GCACTCTGAT CTACAAAGTC

8051 AAGATGCGAG GCACCAATTT TCCACCGGAT GGACCAGTGA TGCAAAAGAA

8101 GACCATGGGT TGGGAAGCTA GTACGGAACG CCTGTATCCG AGGGATGGTG

8151 TGCTCAAGGG CGAGATCCAC CAGGCATTGA AACTTAAGGA CGGTGGACAT

8201 TACTTGGTCG AGTTCAAAAC TATTTACATG GCTAAGAAAC CCGTCCAGCT

8251 GCCCGGCTAC TATTATGTCG ATACCAAGCT GGACATCACC TCCCACAATG

8301 AAGACTACAC CATCGTGGAG CAGTACGAAC GCAGTGAGGG CCGCCACCAC

8351 TTGTTCCTGT ACGGTATGGA CGAGCTTTAC AAGTAAAGGC CTATGAATCG

8401 TTTTTAAAAT AACAAATCAA TTGTTTTATA ATATTCGTAC GATTCTTTGA

8451 TTATGTAATA AAATGTGATC ATTAGGAAGA TTACGAAAAA TATAAAAAAT

8501 ATGAGTTCTG TGTGTATAAC AAATGCTGTA AACGCCACAA TTGTGTTTGT

8551 TGCAAATAAA CCCATGATTA TTTGATTAAA ATTGTTGTTT TCTTTGTTCA

8601 TAGACAATAG TGTGTTTTGC CTAAACGTGT ACTGCATAAA CTCCATGCGA

8651 GTGTATAGCG AGCTAGTGGC TAACGCTTGC CCCACCAAAG TAGATTCGTC

8701 AAAATCCTCA ATTTCATCAC CCTCCTCCAA GTTTAACATT TGGCCGTCGG

8751 AATTAACTTC TAAAGATGCC ACATAATCTA ATAAATGAAA TAGAGATTCA

8801 AACGTGGCGT CATCGTCCGT TTCGACCATT TCCGAAAAGA ACTCGGGCAT

8851 AAACTCTATG ATTTCTCTGG ACGTGGTGTT GTCGAAACTC TCAAAGTACG

8901 CAGTCAGGAA CGTGCGCGAC ATGTCGTCGG GAAACTCGCG CGGAAACATG

8951 TTGTTGTAAC CGAACGGGTC CCATAGCGCC AAAACCAAAT CTGCCAGCGT

9001 CAATAGAATG AGCACGATGC CGACAATGGA GCTGGCTTGG ATAGCGATTC

9051 GAGTTAACCT AGTTCTAGAG CGGCCGCTGG CCACGGGTGC GCATGATCGT

9101 GCTCCTGTCG TTGAGGACCC GGCTAGGCTG GCGGGGTTGC CTTACTGGTT

9151 AGCAGAATGA ATCACCGATA CGCGAGCGAA CGTGAAGCGA CTGCTGCTGC

9201 AAAACGTCTG CGACCTGAGC AACAACATGA ATGGTCTTCG GTTTCCGTGT

9251 TTCGTAAAGT CTGGAAACGC GGAAGTCAGC GCCCTGCACC ATTATGTTCC

9301 GGAGGCGCGC CCTAGTTCCA GTGAAATCCA AGCATTTTCT AAATTAAATG

9351 TATTCTTATT ATTATAGTTG TTATTTTTGA TATATATAAA CAACACTATT

9401 ATGCCCACCA TTTTTTTGAG ATGCATCTAC ACAAGGAACA AACACTGGAT

9451 GTCACTTTCA GTTCAAATTG TAACGCTAAT CACTCCGAAC AGGTCACAAA

9501 AAATTACCTT AAAAAGTCAT AATATTAAAT TAGAATAAAT ATAGCTGTGA

9551 GGGAAATATA TACAAATATA TTGGAGCAAA TAAATTGTAC ATACAAATAT

9601 TTATTACTAA TTTCTATTGA GACGAAATGA ACCACTCGGA ACCATTTGAG

9651 CGAACCGAAT CGCGCGGAAC TAACGACAGT CGCTCCAAGG TCGTCGAACA

9701 AAAGGTGAAT GTGTTGCGGA GAGCGGGTGG GAGACAGCGA AAGAGCAACT

9751 ACGAAACGTG GTGTGGTGGA GGTGAATTAT GAAGAGGGCG CGCGATTTGA

9801 AAAGTATGTA TATAAAAAAT ATATCCCGGT GTTTTATGTA GCGATAAACG

9851 AGTTTTTGAT GTAAGGTATG CAGGTGTGTA AGTCTTTTGG TTAGAAGACA

9901 AATCCAAAGT CTACTTGTGG GGATGTTCGA AGGGGAAATA CTTGTATTCT

9951 ATAGGTCATA TCTTGTTTTT ATTGGCACAA ATATAATTAC ATTAGCTTTT

10001 TGAGGGGGCA ATAAACAGTA AACACGATGG TAATAATGGT AAAAAAAAAA

10051 AACAAGCAGT TATTTCGGAT ATATGTCGGC TACTCCTTGC GTCGGGCCCG

10101 AAGTCTTAGA GCCAGATATG CGAGCACCCG GAAGCTCACG ATGAGAATGG

10151 CCAGACCCAC GTAGTCCAGC GGCAGATCGG CGGCGGAGAA GTTAAGCGTC

10201 TCCAGGATGA CCTTGCCCGA ACTGGGGCAC GTGGTGTTCG ACGATGTGCA

10251 GCTAATTTCG CCCGGCTCCA CGTCCGCCCA TTGGTTAATC AGCAGACCCT

10301 CGTTGGCGTA ACGGAACCAT GAGAGGTACG ACAACCATTT GAGGTATACT

10351 GGCACCGAGC CCGAGTTCAA GAAGAAGCCG CCAAAGAGCA GGAATGGTAT

10401 GATAACCGGC GGACCCACAG ACAGCGCCAT CGAGGTCGAG GAGCTGGCGC

10451 AGGATATTAG ATATCCGAAG GACGTTGACA CATTGGCCAC CAGAGTGACC

10501 AGCGCCAGGC AGTTGAAGAA GTGCAGCACT CCGGCCCGCA GTCCGATCAT

10551 CGGATAGGCA ATCGCCGTGA AGACCAGTGG CACTGTGAGA AAAAGCGGCA

10601 ATTCGGCAAT CGTTTTGCCC AGAAAGTATG TGTCACAGCG ATAAAGTCGA

10651 CTTCGGGCCT CCCTCATAAA AACTGGCAGC TCTGAGGTGA ACACCTAAAT

10701 CGAATCGATT CATTAGAAAG TTAGTAAATT ATTGAAATGC AAATGTATTC

10751 TAAACATGAC TTACATTTAT CGTGGCAAAG ACGTTTTGAA AGGTCATGTT

10801 GGTCAGGAAG AGGAAGATGG CTCCGTTGAT ATTCATCACA CCCACTTGCG

10851 TGAGTTGTTG GCCCAAAAAG ATGAGGCCAA TCAAGATGGC AACCATCTGC

10901 AAATTAAAAT GTTACTCGCA TCTCATTAAT ATTCGCGAGT TAAATGAAAT

10951 TTATTTATCT TCTGCAAAAC TATAAACTAT ACATCTCATT GAAAAAAACT

11001 AAGAAGGGTG TGGAATCAGG CAATTCTATC TAAAATCTAG CGAATTTGTT

11051 TCCAAGAATT GTAAGCGTTA TATCATTTGT TTCCACTGGA ACCACTCACC

11101 GTTGTCTGAA TAAGTCGCAC TTTTACGAGG AGTGGTTCCT TGAGCACCGA

11151 CAGCCAGGAT CGCCACAGGA CCGCCCGGAA CTGCATGAAC CAGGTGGCCT

11201 TGTAGGTGTA CCCATTCTCC GGCTGCTCCA GTGGCTTCTC CAGATTTTTG

11251 GTGGCCAACA ACTGCTCCAT ATCCCGGGCT ACTTTGCTAA TGGCAAAATT

11301 GTCGCATATC TTGGCGATCC GATCACGGGA CTCGATCTCC CGTCCGGGCA

11351 CAACGGCCAA CACCTGTACG TAAAAGTCCG CCGGATTGTA GTTGGTAGGA

11401 CACTGGGCAC CCACGCTGGA TAGGAGTTGA GATGTTATGT AATACTAGAT

11451 ACCCTTAATA AACACATCGA ACTCACTAGG AAAAGAAGTC GACGGCTTCG

11501 CTGGGAGTGC CCAAGAAAGC TACCCTGCCC TCGGCCATCA GAAGGATCTT

11551 GTCAAAGAGC TCAAACAGCT CGGAAGACGG CTGATGAATG GTCAGGATGA

11601 CGGTCTTGCC CTTCTGCGAC AGCTTCTTCA GCACCTGGAC GACGCTGTGG

11651 GCGGTAAAGG AGTCCAGTCC GGAGGTGGGC TCATCGCAGA TCAGAAGCGG

11701 CGGATCGGTT AGAGCCTCGG AGGCGAATGC CAGACGCTTC CTTTCTCCGC

11751 CGGACAGACC TTTCACCCTG CCGGGCACAC CGATGATCGT GTGCTGACAT

11801 TTGCTGAGCG AAAGCTCCTG GATCACCTGA TCCACGCGGG CCACTCGCTG

11851 CCGATAGGTC AGATGTCGTG GCATCCGCAC CATGGCTTGG AAAATCAGGT

11901 GTTCCCTGGC CGTTAGGGAG CCGATAAAGA GGTCATCCTG CTGGACATAG

11951 GCGCACCTGG CCTGCATCTC CTTGGCGTCC ACAGGTTGGC CATTGAGCAG

12001 TCGCATCCCG GATGGCGATA CTTGGATGCC CTGCGGCGAT CGAAAGGCAA

12051 GGGCATTCAG CAGGGTCGTC TTTCCGGCAC CGGAACTGCC CATCACGGCC

12101 AAAAGTTCGC CCGGATAGGC CACGCCGCAA ACTGAGTTTC AAATTGGTAA

12151 TTGGACCCTT TATTAAGATT TCACACAGAT CAGCCGACTG CGAATAGAAA

12201 CTCACCGTTC TTGAGCAAAT GTTTCCTGGG CGCCGGTATG TGTCGCTCGT

12251 TGCAGAATAG TCCGCGTGTC CGGTTGACCA GCTGCCGCCA TCCGGAGCCC

12301 GGCTGATTGA CCGCCCCAAA GATGTCCATA TTGTGCCAGG CATAGGTGAG

12351 GTTCTCGGCT AGTTGGCCGC TCCCTGAACC GGAGTCCTCC GGCGGACTGG

12401 GTGGCCGGAG CGTGCCGTAG TTTTTGGCCT GCCCGAAGCC CTGGTTAATG

12451 CAGCTCTGCG AAGCCGCTCC GCTGTCACCC TGCAATGATA GGGGATCTCA

12501 AATATCAACT ACTAGCGTTA TGCTCATCTA ACCCCGAACA AAAAGTACCC

12551 CGAAGTATCC TACGAAGTAG GTTTATACTT TTATTTATTT TTTGTGCATC

12601 TAGGATCAGC TTAAAATATC TGGTTGTTAT ATTTTTTGTA AAAAAGAATA

12651 TAGTCGAAAA TGAATGCCTT TAGATGTCTT GATCATGATA TGATCTCAAA

12701 AATTGTCTTA TATAGCGAGA ACAGCTACCA GAATAATCTG TTTCGTGTCA

12751 CTATTTGTTT GTGCAATTGC GGTTTGGGAT TTTTGTGGGT CGCAGTTCTC

12801 ACGCCGCATA CAATTTGATG TTGCAATCGC AGTTCCTATA GATCAAGTGA

12851 ACTTAAGATG TATGCACATG TACTACTCAC ATTGTTCAGA TGCTCGGCAG

12901 ATGGGTGTTT GCTGCCTCCG CGAATTAATA GCTCCTGATC CTCTTGGCCC

12951 ATTGCCGGGA TTTTTCACAC TTTCCCCTGC TTACCCACCC AAAACCAATC

13001 ACCACCCCAA TCACTCAAAA AACAAACAAA AATAAGAAGC GAGAGGAGTT

13051 TTGGCACAGC ACTTTGTGTT TAATTGATGG CGTAAACCGC TTGGAGCTTC

13101 GTCACGAAAC CGCTGACAAA ATGCAACTGA AGGCGGACAT TGACGCTACG

13151 TAACGCTACA AACGGTGGCG AAAGAGATAG CGGACGCAGC GGCGAAAGAG

13201 ACGGCGATAT TTCTGTGGAC AGAGAAGGAG GCAAACAGCG CTGACTTTGA

13251 GTGGAATGTC ATTTTGAGTG AGAGGTAATC GAAAGAACCT GGTACATCAA

13301 ATACCCTTGG ATCGAAGTAA ATTTAAAACT GATCAGATAA GTTCAATGAT

13351 ATCCAGTGCA GTAAAAAAAA AAAATGTTTT TTTTATCTAC TTTCCGCAAA

13401 AATGGGTTTT ATTAACTTAC ATACATACTA GGCGCGCCCA TAAGCTTGCA

13451 TGCCTGCAGG TCGGAGTACT GTCCTCCGAG CGGAGTACTG TCCTCCGAGC

13501 GGAGTACTGT CCTCCGAGCG GAGTACTGTC CTCCGAGCGG AGTACTGTCC

13551 TCCGAGCGGA GACTCTAGCC CTAGGGCATG CCTGCAGGTC GGAGTACTGT

13601 CCTCCGAGCG GAGTACTGTC CTCCGAGCGG AGTACTGTCC TCCGAGCGGA

13651 GTACTGTCCT CCGAGCGGAG TACTGTCCTC CGAGCGGAGA CTCTAGCGCT

13701 AGCGACGTCG AGCGCCGGAG TATAAATAGA GGCGCTTCGT CTACGGAGCG

13751 ACAATTCAAT TCAAACAAGC AAAGTGAACA CGTCGCTAAG CGAAAGCTAA

13801 GCAAATAAAC AAGCGCAGCT GAACAAGCTA AACAATCTGC AGTAAAGTGC

13851 AAGTTAAAGT GAATCAATTA AAAGTAACCA GCAACCAAGT AAATCAACTG

13901 CAACTACTGA AATCTGCCAA GAAGTAATTA TTGAATACAA GAAGAGAACT

13951 CTGAATAGAT CTGCGGCCGC GGCTCGAGCA AAATGGCCTC ACCGTTGACC

14001 CGCTTTCTGT CGCTGAACCT GCTGCTGCTG GGTGAGTCGA TTATCCTGGG

14051 GAGTGGAGAA GCTAAGCCAC AGGCACCCGA ACTCCGAATC TTTCCAAAGA

14101 AAATGGACGC CGAACTTGGT CAGAAGGTGG ACCTGGTATG TGAAGTGTTG

14151 GGGTCCGTTT CGCAAGGATG CTCTTGGCTC TTCCAGAACT CCAGCTCCAA

14201 ACTCCCCCAG CCCACCTTCG TTGTCTATAT GGCTTCATCC CACAACAAGA

14251 TAACGTGGGA CGAGAAGCTG AATTCGTCGA AACTGTTTTC TGCCATGAGG

14301 GACACGAATA ATAAGTACGT TCTCACCCTG AACAAGTTCA GCAAGGAAAA

14351 CGAAGGCTAC TATTTCTGCT CAGTCATCAG CAACTCGGTG ATGTACTTCA

14401 GTTCTGTCGT GCCAGTCCTT CAGAAAGTGA ACTCTACTAC TACCAAGCCA

14451 GTGCTGCGAA CTCCCTCACC TGTGCACCCT ACCGGGACAT CTCAGCCCCA

14501 GAGACCAGAA GATTGTCGGC CCCGTGGCTC AGTGAAGGGG ACCGGATTGG

14551 ACTTCGCCTG TGATATTTAC ATCTGGGCAC CCTTGGCCGG AATCTGCGTG

14601 GCCCTTCTGC TGTCCTTGAT CATCACTCTC ATCTGCTACC ACAGCCGCGG

14651 ATCCATGAGT AAAGGAGAAG AACTTTTCAC TGGAGTTGTC CCAATTCTTG

14701 TTGAATTAGA TGGTGATGTT AATGGGCACA AATTTTCTGT CAGTGGAGAG

14751 GGTGAAGGTG ATGCAACATA CGGAAAACTT ACCCTTAAAT TTATTTGCAC

14801 TACTGGAAAA CTACCTGTTC CATGGCCAAC ACTTGTCACT ACTTTAACTT

14851 ATGGTGTTCA ATGCTTTTCA AGATACCCAG ATCATATGAA ACAGCATGAC

14901 TTTTTCAAGA GTGCCATGCC CGAAGGTTAT GTCCAGGAAA GAACTATATT

14951 TTTCAAAGAT GACGGGAACT ACAAGACACG TGCTGAAGTC AAGTTTGAAG

15001 GTGATACCCT TGTTAATAGA ATCGAGTTAA AAGGTATTGA TTTTAAAGAA

15051 GATGGAAACA TTCTTGGACA CAAATTGGAA TACAACTATA ACTCACACAA

15101 TGTATACATC ATGGCAGACA AACAAAAGAA TGGAATCAAA GTTAACTTCA

15151 AAATTAGACA CAACATTGAA GATGGAAGCG TTCAACTAGC AGACCATTAT

15201 CAACAAAATA CTCCAATTGG CGATGGCCCT GTCCTTTTAC CAGACAACCA

15251 TTACCTGTCC ACACAATCTG CCCTTTCGAA AGATCCCAAC GAAAAGAGAG

15301 ACCACATGGT CCTTCTTGAG TTTGTAACAG CTGCTGGGAT TACACATGGC

15351 ATGGATGAAC TATACAAATA ATCTAGAGGA TCTTTGTGAA GGAACCTTAC

15401 TTCTGTGGTG TGACATAATT GGACAAACTA CCTACAGAGA TTTAAAGCTC

15451 TAAGGTAAAT ATAAAATTTT TAAGTGTATA ATGTGTTAAA CTACTGATTC

15501 TAATTGTTTG TGTATTTTAG ATTCCAACCT ATGGAACTGA TGAATGGGAG

15551 CAGTGGTGGA ATGCCTTTAA TGAGGAAAAC CTGTTTTGCT CAGAAGAAAT

15601 GCCATCTAGT GATGATGAGG CTACTGCTGA CTCTCAACAT TCTACTCCTC

15651 CAAAAAAGAA GAGAAAGGTA GAAGACCCCA AGGACTTTCC TTCAGAATTG

15701 CTAAGTTTTT TGAGTCATGC TGTGTTTAGT AATAGAACTC TTGCTTGCTT

15751 TGCTATTTAC ACCACAAAGG AAAAAGCTGC ACTGCTATAC AAGAAAATTA

15801 TGGAAAAATA TTTGATGTAT AGTGCCTTGA CTAGAGATCA TAATCAGCCA

15851 TACCACATTT GTAGAGGTTT TACTTGCTTT AAAAAACCTC CCACACCTCC

15901 CCCTGAACCT GAAACATAAA ATGAATGCAA TTGTTGTTGT TAACTTGTTT

15951 ATTGCAGCTT ATAATGGTTA CAAATAAAGC AATAGCATCA CAAATTTCAC

16001 AAATAAAGCA TTTTTTTCAC TGCATTCTAG TTGTGGTTTG TCCAAACTCA

16051 TCAATGTATC TTATCATGTC TGGATCACCG GTTATGTTCG GCTTGTCGAC

16101 ATGCCCGCCG TGACCGTCGA GAACCCGCTG ACGCTGCCCC GCGTATCCGC

16151 ACCCGCCGAC GCCGTCGCAC GTCCCGTGCT CACCGTGACC ACCGCGCCCA

16201 GCGGTTTCGA GGGCGAGGGC TTCCCGGTGC GCCGCGCGTT CGCCGGGATC

16251 AACTACCGCC ACCTCGACCC GTTCATCATG ATGGACCAGA TGGGTGAGGT

16301 GGAGTACGCG CCCGGGGAGC CCAAGGGCAC GCCCTGGCAC CCGCACCGCG

16351 GCTTCGAGAC CGTGACCTAC ATCGTCGACG GTAACATGTG AGCAAAAGGC

16401 CAGCAAAAGG CCAGGAACCG TAAAAAGGCC GCGTTGCTGG CGTTTTTCCA

16451 TAGGCTCCGC CCCCCTGACG AGCATCACAA AAATCGACGC TCAAGTCAGA

16501 GGTGGCGAAA CCCGACAGGA CTATAAAGAT ACCAGGCGTT TCCCCCTGGA

16551 AGCTCCCTCG TGCGCTCTCC TGTTCCGACC CTGCCGCTTA CCGGATACCT

16601 GTCCGCCTTT CTCCCTTCGG GAAGCGTGGC GCTTTCTCAT AGCTCACGCT

16651 GTAGGTATCT CAGTTCGGTG TAGGTCGTTC GCTCCAAGCT GGGCTGTGTG

16701 CACGAACCCC CCGTTCAGCC CGACCGCTGC GCCTTATCCG GTAACTATCG

16751 TCTTGAGTCC AACCCGGTAA GACACGACTT ATCGCCACTG GCAGCAGCCA

16801 CTGGTAACAG GATTAGCAGA GCGAGGTATG TAGGCGGTGC TACAGAGTTC

16851 TTGAAGTGGT GGCCTAACTA CGGCTACACT AGAAGAACAG TATTTGGTAT

16901 CTGCGCTCTG CTGAAGCCAG TTACCTTCGG AAAAAGAGTT GGTAGCTCTT

16951 GATCCGGCAA ACAAACCACC GCTGGTAGCG GTGGTTTTTT TGTTTGCAAG

17001 CAGCAGATTA CGCGCAGAAA AAAAGGATCT CAAGAAGATC CTTTGATCTT

17051 TTCTACGGGG TCTGACGCTC AGTGGAACGA AAACTCACGT TAAGGGATTT

17101 TGGTCATGAG ATTATCAAAA AGGATCTTCA CCTAGATCCT TTTAAATTAA

17151 AAATGAAGTT TTAAATCAAT CTAAAGTATA TATGAGTAAA CTTGGTCTGA

17201 CAGTTACCAA TGCTTAATCA GTGAGGCACC TATCTCAGCG ATCTGTCTAT

17251 TTCGTTCATC CATAGTTGCC TGACTCCCCG TCGTGTAGAT AACTACGATA

17301 CGGGAGGGCT TACCATCTGG CCCCAGTGCT GCAATGATAC CGCGAGACCC

17351 ACGCTCACCG GCTCCAGATT TATCAGCAAT AAACCAGCCA GCCGGAAGGG

17401 CCGAGCGCAG AAGTGGTCCT GCAACTTTAT CCGCCTCCAT CCAGTCTATT

17451 AATTGTTGCC GGGAAGCTAG AGTAAGTAGT TCGCCAGTTA ATAGTTTGCG

17501 CAACGTTGTT GCCATTGCTA CAGGCATCGT GGTGTCACGC TCGTCGTTTG

17551 GTATGGCTTC ATTCAGCTCC GGTTCCCAAC GATCAAGGCG AGTTACATGA

17601 TCCCCCATGT TGTGCAAAAA AGCGGTTAGC TCCTTCGGTC CTCCGATCGT

17651 TGTCAGAAGT AAGTTGGCCG CAGTGTTATC ACTCATGGTT ATGGCAGCAC

17701 TGCATAATTC TCTTACTGTC ATGCCATCCG TAAGATGCTT TTCTGTGACT

17751 GGTGAGTACT CAACCAAGTC ATTCTGAGAA TAGTGTATGC GGCGACCGAG

17801 TTGCTCTTGC CCGGCGTCAA TACGGGATAA TACCGCGCCA CATAGCAGAA

17851 CTTTAAAAGT GCTCATCATT GGAAAACGTT CTTCGGGGCG AAAACTCTCA

17901 AGGATCTTAC CGCTGTTGAG ATCCAGTTCG ATGTAACCCA CTCGTGCACC

17951 CAACTGATCT TCAGCATCTT TTACTTTCAC CAGCGTTTCT GGGTGAGCAA

18001 AAACAGGAAG GCAAAATGCC GCAAAAAAGG GAATAAGGGC GACACGGAAA

18051 TGTTGAATAC TCATACTCTT CCTTTTTCAA TATTATTGAA GCATTTATCA

18101 GGGTTATTGT CTCATGAGCG GATACATATT TGAATGTATT TAGAAAAATA

18151 AACAAATAGG GGTTCCGCGC ACATTTCCCC GAAAAGTGCC ACCTGAGTC

**HJP-226-10XUAS-NrdJ-1^C^-Cre_C_**

1 CGACAGGAGC ACGATCATGC GCACCCGTGG CCAGGGCCGC AAGCTTGCAT

51 GCCTGCAGGT CGGAGTACTG TCCTCCGAGC GGAGTACTGT CCTCCGAGCG

101 GAGTACTGTC CTCCGAGCGG AGTACTGTCC TCCGAGCGGA GTACTGTCCT

151 CCGAGCGGAG ACTCTAGCCC TAGGGCATGC CTGCAGGTCG GAGTACTGTC

201 CTCCGAGCGG AGTACTGTCC TCCGAGCGGA GTACTGTCCT CCGAGCGGAG

251 TACTGTCCTC CGAGCGGAGT ACTGTCCTCC GAGCGGAGAC TCTAGCGCTA

301 GCGACGTCGA GCGCCGGAGT ATAAATAGAG GCGCTTCGTC TACGGAGCGA

351 CAATTCAATT CAAACAAGCA AAGTGAACAC GTCGCTAAGC GAAAGCTAAG

401 CAAATAAACA AGCGCAGCTG AACAAGCTAA ACAATCTGCA GTAAAGTGCA

451 AGTTAAAGTG AATCAATTAA AAGTAACCAG CAACCAAGTA AATCAACTGC

501 AACTACTGAA ATCTGCCAAG AAGTAATTAT TGAATACAAG AAGAGAACTC

551 TGAATAGATC TGCGGCCGCA ATCAAAATGG AGGCCAAGAC CTACATCGGC

601 AAGCTGAAGA GCCGCAAGAT CGTGAGCAAC GAGGATACCT ACGATATCCA

651 GACCAGCACC CACAACTTCT TCGCCAACGA TATCCTGGTG CACAACTCGG

701 CAACAAGTCA GTTGAGTACG CGCGCGTTGG AGGGAATCTT CGAGGCCACT

751 CACCGATTGA TTTACGGTGC AAAAGATGAC TCCGGCCAAC GTTACTTGGC

801 CTGGTCCGGT CACAGCGCAC GTGTGGGTGC CGCCCGTGAC ATGGCGAGGG

851 CCGGTGTTTC CATCCCAGAA ATCATGCAAG CGGGCGGATG GACCAACGTG

901 AATATTGTAA TGAACTACAT TCGTAATCTG GATAGTGAAA CAGGCGCAAT

951 GGTGCGCCTG CTGGAGGACG GAGATTAATC TAGAGGATCT TTGTGAAGGA

1001 ACCTTACTTC TGTGGTGTGA CATAATTGGA CAAACTACCT ACAGAGATTT

1051 AAAGCTCTAA GGTAAATATA AAATTTTTAA GTGTATAATG TGTTAAACTA

1101 CTGATTCTAA TTGTTTGTGT ATTTTAGATT CCAACCTATG GAACTGATGA

1151 ATGGGAGCAG TGGTGGAATG CCTTTAATGA GGAAAACCTG TTTTGCTCAG

1201 AAGAAATGCC ATCTAGTGAT GATGAGGCTA CTGCTGACTC TCAACATTCT

1251 ACTCCTCCAA AAAAGAAGAG AAAGGTAGAA GACCCCAAGG ACTTTCCTTC

1301 AGAATTGCTA AGTTTTTTGA GTCATGCTGT GTTTAGTAAT AGAACTCTTG

1351 CTTGCTTTGC TATTTACACC ACAAAGGAAA AAGCTGCACT GCTATACAAG

1401 AAAATTATGG AAAAATATTT GATGTATAGT GCCTTGACTA GAGATCATAA

1451 TCAGCCATAC CACATTTGTA GAGGTTTTAC TTGCTTTAAA AAACCTCCCA

1501 CACCTCCCCC TGAACCTGAA ACATAAAATG AATGCAATTG TTGTTGTTAA

1551 CTTGTTTATT GCAGCTTATA ATGGTTACAA ATAAAGCAAT AGCATCACAA

1601 ATTTCACAAA TAAAGCATTT TTTTCACTGC ATTCTAGTTG TGGTTTGTCC

1651 AAACTCATCA ATGTATCTTA TCATGTCTGG ATCGATCTGG CCGGCCGTTT

1701 AAACGAATTC TTGAAGACGA AAGGGCCTCG TGATACGCCT ATTTTTATAG

1751 GTTAATGTCA TGATAATAAT GGTTTCTTAG ACTCAGGTGG CACTTTTCGG

1801 GGAAATGTGC GCGGAACCCC TATTTGTTTA TTTTTCTAAA TACATTCAAA

1851 TATGTATCCG CTCATGAGAC AATAACCCTG ATAAATGCTT CAATAATATT

1901 GAAAAAGGAA GAGTATGAGT ATTCAACATT TCCGTGTCGC CCTTATTCCC

1951 TTTTTTGCGG CATTTTGCCT TCCTGTTTTT GCTCACCCAG AAACGCTGGT

2001 GAAAGTAAAA GATGCTGAAG ATCAGTTGGG TGCACGAGTG GGTTACATCG

2051 AACTGGATCT CAACAGCGGT AAGATCCTTG AGAGTTTTCG CCCCGAAGAA

2101 CGTTTTCCAA TGATGAGCAC TTTTAAAGTT CTGCTATGTG GCGCGGTATT

2151 ATCCCGTATT GACGCCGGGC AAGAGCAACT CGGTCGCCGC ATACACTATT

2201 CTCAGAATGA CTTGGTTGAG TACTCACCAG TCACAGAAAA GCATCTTACG

2251 GATGGCATGA CAGTAAGAGA ATTATGCAGT GCTGCCATAA CCATGAGTGA

2301 TAACACTGCG GCCAACTTAC TTCTGACAAC GATCGGAGGA CCGAAGGAGC

2351 TAACCGCTTT TTTGCACAAC ATGGGGGATC ATGTAACTCG CCTTGATCGT

2401 TGGGAACCGG AGCTGAATGA AGCCATACCA AACGACGAGC GTGACACCAC

2451 GATGCCTGTA GCAATGGCAA CAACGTTGCG CAAACTATTA ACTGGCGAAC

2501 TACTTACTCT AGCTTCCCGG CAACAATTAA TAGACTGGAT GGAGGCGGAT

2551 AAAGTTGCAG GACCACTTCT GCGCTCGGCC CTTCCGGCTG GCTGGTTTAT

2601 TGCTGATAAA TCTGGAGCCG GTGAGCGTGG GTCTCGCGGT ATCATTGCAG

2651 CACTGGGGCC AGATGGTAAG CCCTCCCGTA TCGTAGTTAT CTACACGACG

2701 GGGAGTCAGG CAACTATGGA TGAACGAAAT AGACAGATCG CTGAGATAGG

2751 TGCCTCACTG ATTAAGCATT GGTAACTGTC AGACCAAGTT TACTCATATA

2801 TACTTTAGAT TGATTTAAAA CTTCATTTTT AATTTAAAAG GATCTAGGTG

2851 AAGATCCTTT TTGATAATCT CATGACCAAA ATCCCTTAAC GTGAGTTTTC

2901 GTTCCACTGA GCGTCAGACC CCGTAGAAAA GATCAAAGGA TCTTCTTGAG

2951 ATCCTTTTTT TCTGCGCGTA ATCTGCTGCT TGCAAACAAA AAAACCACCG

3001 CTACCAGCGG TGGTTTGTTT GCCGGATCAA GAGCTACCAA CTCTTTTTCC

3051 GAAGGTAACT GGCTTCAGCA GAGCGCAGAT ACCAAATACT GTTCTTCTAG

3101 TGTAGCCGTA GTTAGGCCAC CACTTCAAGA ACTCTGTAGC ACCGCCTACA

3151 TACCTCGCTC TGCTAATCCT GTTACCAGTG GCTGCTGCCA GTGGCGATAA

3201 GTCGTGTCTT ACCGGGTTGG ACTCAAGACG ATAGTTACCG GATAAGGCGC

3251 AGCGGTCGGG CTGAACGGGG GGTTCGTGCA CACAGCCCAG CTTGGAGCGA

3301 ACGACCTACA CCGAACTGAG ATACCTACAG CGTGAGCTAT GAGAAAGCGC

3351 CACGCTTCCC GAAGGGAGAA AGGCGGACAG GTATCCGGTA AGCGGCAGGG

3401 TCGGAACAGG AGAGCGCACG AGGGAGCTTC CAGGGGGAAA CGCCTGGTAT

3451 CTTTATAGTC CTGTCGGGTT TCGCCACCTC TGACTTGAGC GTCGATTTTT

3501 GTGATGCTCG TCAGGGGGGC GGAGCCTATG GAAAAACGCC AGCAACGCGG

3551 CCTTTTTACG GTTCCTGGCC TTTTGCTGGC CTTTTGCTCA CATGTTACCG

3601 TCGACGATGT AGGTCACGGT CTCGAAGCCG CGGTGCGGGT GCCAGGGCGT

3651 GCCCTTGGGC TCCCCGGGCG CGTACTCCAC CTCACCCATC TGGTCCATCA

3701 TGATGAACGG GTCGAGGTGG CGGTAGTTGA TCCCGGCGAA CGCGCGGCGC

3751 ACCGGGAAGC CCTCGCCCTC GAAACCGCTG GGCGCGGTGG TCACGGTGAG

3801 CACGGGACGT GCGACGGCGT CGGCGGGTGC GGATACGCGG GGCAGCGTCA

3851 GCGGGTTCTC GACGGTCACG GCGGGCATGT CGACAAGCCG AACATATGGG

3901 CGCGCCTAGT ATGTATGTAA GTTAATAAAA CCCATTTTTG CGGAAAGTAG

3951 ATAAAAAAAA CATTTTTTTT TTTTACTGCA CTGGATATCA TTGAACTTAT

4001 CTGATCAGTT TTAAATTTAC TTCGATCCAA GGGTATTTGA TGTACCAGGT

4051 TCTTTCGATT ACCTCTCACT CAAAATGACA TTCCACTCAA AGTCAGCGCT

4101 GTTTGCCTCC TTCTCTGTCC ACAGAAATAT CGCCGTCTCT TTCGCCGCTG

4151 CGTCCGCTAT CTCTTTCGCC ACCGTTTGTA GCGTTACGTA GCGTCAATGT

4201 CCGCCTTCAG TTGCATTTTG TCAGCGGTTT CGTGACGAAG CTCCAAGCGG

4251 TTTACGCCAT CAATTAAACA CAAAGTGCTG TGCCAAAACT CCTCTCGCTT

4301 CTTATTTTTG TTTGTTTTTT GAGTGATTGG GGTGGTGATT GGTTTTGGGT

4351 GGGTAAGCAG GGGAAAGTGT GAAAAATCCC GGCAATGGGC CAAGAGGATC

4401 AGGAGCTATT AATTCGCGGA GGCAGCAAAC ACCCATCTGC CGAGCATCTG

4451 AACAATGTGA GTAGTACATG TGCATACATC TTAAGTTCAC TTGATCTATA

4501 GGAACTGCGA TTGCAACATC AAATTGTATG CGGCGTGAGA ACTGCGACCC

4551 ACAAAAATCC CAAACCGCAA TTGCACAAAC AAATAGTGAC ACGAAACAGA

4601 TTATTCTGGT AGCTGTTCTC GCTATATAAG ACAATTTTTG AGATCATATC

4651 ATGATCAAGA CATCTAAAGG CATTCATTTT CGACTATATT CTTTTTTACA

4701 AAAAATATAA CAACCAGATA TTTTAAGCTG ATCCTAGATG CACAAAAAAT

4751 AAATAAAAGT ATAAACCTAC TTCGTAGGAT ACTTCGGGGT ACTTTTTGTT

4801 CGGGGTTAGA TGAGCATAAC GCTAGTAGTT GATATTTGAG ATCCCCTATC

4851 ATTGCAGGGT GACAGCGGAG CGGCTTCGCA GAGCTGCATT AACCAGGGCT

4901 TCGGGCAGGC CAAAAACTAC GGCACGCTCC GGCCACCCAG TCCGCCGGAG

4951 GACTCCGGTT CAGGGAGCGG CCAACTAGCC GAGAACCTCA CCTATGCCTG

5001 GCACAATATG GACATCTTTG GGGCGGTCAA TCAGCCGGGC TCCGGATGGC

5051 GGCAGCTGGT CAACCGGACA CGCGGACTAT TCTGCAACGA GCGACACATA

5101 CCGGCGCCCA GGAAACATTT GCTCAAGAAC GGTGAGTTTC TATTCGCAGT

5151 CGGCTGATCT GTGTGAAATC TTAATAAAGG GTCCAATTAC CAATTTGAAA

5201 CTCAGTTTGC GGCGTGGCCT ATCCGGGCGA ACTTTTGGCC GTGATGGGCA

5251 GTTCCGGTGC CGGAAAGACG ACCCTGCTGA ATGCCCTTGC CTTTCGATCG

5301 CCGCAGGGCA TCCAAGTATC GCCATCCGGG ATGCGACTGC TCAATGGCCA

5351 ACCTGTGGAC GCCAAGGAGA TGCAGGCCAG GTGCGCCTAT GTCCAGCAGG

5401 ATGACCTCTT TATCGGCTCC CTAACGGCCA GGGAACACCT GATTTTCCAA

5451 GCCATGGTGC GGATGCCACG ACATCTGACC TATCGGCAGC GAGTGGCCCG

5501 CGTGGATCAG GTGATCCAGG AGCTTTCGCT CAGCAAATGT CAGCACACGA

5551 TCATCGGTGT GCCCGGCAGG GTGAAAGGTC TGTCCGGCGG AGAAAGGAAG

5601 CGTCTGGCAT TCGCCTCCGA GGCTCTAACC GATCCGCCGC TTCTGATCTG

5651 CGATGAGCCC ACCTCCGGAC TGGACTCCTT TACCGCCCAC AGCGTCGTCC

5701 AGGTGCTGAA GAAGCTGTCG CAGAAGGGCA AGACCGTCAT CCTGACCATT

5751 CATCAGCCGT CTTCCGAGCT GTTTGAGCTC TTTGACAAGA TCCTTCTGAT

5801 GGCCGAGGGC AGGGTAGCTT TCTTGGGCAC TCCCAGCGAA GCCGTCGACT

5851 TCTTTTCCTA GTGAGTTCGA TGTGTTTATT AAGGGTATCT AGTATTACAT

5901 AACATCTCAA CTCCTATCCA GCGTGGGTGC CCAGTGTCCT ACCAACTACA

5951 ATCCGGCGGA CTTTTACGTA CAGGTGTTGG CCGTTGTGCC CGGACGGGAG

6001 ATCGAGTCCC GTGATCGGAT CGCCAAGATA TGCGACAATT TTGCCATTAG

6051 CAAAGTAGCC CGGGATATGG AGCAGTTGTT GGCCACCAAA AATCTGGAGA

6101 AGCCACTGGA GCAGCCGGAG AATGGGTACA CCTACAAGGC CACCTGGTTC

6151 ATGCAGTTCC GGGCGGTCCT GTGGCGATCC TGGCTGTCGG TGCTCAAGGA

6201 ACCACTCCTC GTAAAAGTGC GACTTATTCA GACAACGGTG AGTGGTTCCA

6251 GTGGAAACAA ATGATATAAC GCTTACAATT CTTGGAAACA AATTCGCTAG

6301 ATTTTAGATA GAATTGCCTG ATTCCACACC CTTCTTAGTT TTTTTCAATG

6351 AGATGTATAG TTTATAGTTT TGCAGAAGAT AAATAAATTT CATTTAACTC

6401 GCGAATATTA ATGAGATGCG AGTAACATTT TAATTTGCAG ATGGTTGCCA

6451 TCTTGATTGG CCTCATCTTT TTGGGCCAAC AACTCACGCA AGTGGGTGTG

6501 ATGAATATCA ACGGAGCCAT CTTCCTCTTC CTGACCAACA TGACCTTTCA

6551 AAACGTCTTT GCCACGATAA ATGTAAGTCA TGTTTAGAAT ACATTTGCAT

6601 TTCAATAATT TACTAACTTT CTAATGAATC GATTCGATTT AGGTGTTCAC

6651 CTCAGAGCTG CCAGTTTTTA TGAGGGAGGC CCGAAGTCGA CTTTATCGCT

6701 GTGACACATA CTTTCTGGGC AAAACGATTG CCGAATTGCC GCTTTTTCTC

6751 ACAGTGCCAC TGGTCTTCAC GGCGATTGCC TATCCGATGA TCGGACTGCG

6801 GGCCGGAGTG CTGCACTTCT TCAACTGCCT GGCGCTGGTC ACTCTGGTGG

6851 CCAATGTGTC AACGTCCTTC GGATATCTAA TATCCTGCGC CAGCTCCTCG

6901 ACCTCGATGG CGCTGTCTGT GGGTCCGCCG GTTATCATAC CATTCCTGCT

6951 CTTTGGCGGC TTCTTCTTGA ACTCGGGCTC GGTGCCAGTA TACCTCAAAT

7001 GGTTGTCGTA CCTCTCATGG TTCCGTTACG CCAACGAGGG TCTGCTGATT

7051 AACCAATGGG CGGACGTGGA GCCGGGCGAA ATTAGCTGCA CATCGTCGAA

7101 CACCACGTGC CCCAGTTCGG GCAAGGTCAT CCTGGAGACG CTTAACTTCT

7151 CCGCCGCCGA TCTGCCGCTG GACTACGTGG GTCTGGCCAT TCTCATCGTG

7201 AGCTTCCGGG TGCTCGCATA TCTGGCTCTA AGACTTCGGG CCCGACGCAA

7251 GGAGTAGCCG ACATATATCC GAAATAACTG CTTGTTTTTT TTTTTTACCA

7301 TTATTACCAT CGTGTTTACT GTTTATTGCC CCCTCAAAAA GCTAATGTAA

7351 TTATATTTGT GCCAATAAAA ACAAGATATG ACCTATAGAA TACAAGTATT

7401 TCCCCTTCGA ACATCCCCAC AAGTAGACTT TGGATTTGTC TTCTAACCAA

7451 AAGACTTACA CACCTGCATA CCTTACATCA AAAACTCGTT TATCGCTACA

7501 TAAAACACCG GGATATATTT TTTATATACA TACTTTTCAA ATCGCGCGCC

7551 CTCTTCATAA TTCACCTCCA CCACACCACG TTTCGTAGTT GCTCTTTCGC

7601 TGTCTCCCAC CCGCTCTCCG CAACACATTC ACCTTTTGTT CGACGACCTT

7651 GGAGCGACTG TCGTTAGTTC CGCGCGATTC GGTTCGCTCA AATGGTTCCG

7701 AGTGGTTCAT TTCGTCTCAA TAGAAATTAG TAATAAATAT TTGTATGTAC

7751 AATTTATTTG CTCCAATATA TTTGTATATA TTTCCCTCAC AGCTATATTT

7801 ATTCTAATTT AATATTATGA CTTTTTAAGG TAATTTTTTG TGACCTGTTC

7851 GGAGTGATTA GCGTTACAAT TTGAACTGAA AGTGACATCC AGTGTTTGTT

7901 CCTTGTGTAG ATGCATCTCA AAAAAATGGT GGGCATAATA GTGTTGTTTA

7951 TATATATCAA AAATAACAAC TATAATAATA AGAATACATT TAATTTAGAA

8001 AATGCTTGGA TTTCACTGGA ACTAGGGCGC GCCTCCGGAA CATAATGGTG

8051 CAGGGCGCTG ACTTCCGCGT TTCCAGACTT TACGAAACAC GGAAACCGAA

8101 GACCATTCAT GTTGTTGCTC AGGTCGCAGA CGTTTTGCAG CAGCAGTCGC

8151 TTCACGTTCG CTCGCGTATC GGTGATTCAT TCTGCTAACC AGTAAGGCAA

8201 CCCCGCCAGC CTAGCCGGGT CCTCAA

**HJP-473-DNE>FLP-T2A-Cre_AB_-NrdJ-1^N^**

1 AAGAAACCAT TATTATCATG ACATTAACCT ATAAAAATAG GCGTATCACG

51 AGGCCCTTTC GTCTTCAAGA ATTCGTTTAT CACAAGTTTG TACAAAAAAG

101 CAGGCTCCGC GGCCGCCCCC TTCACCTCAC TTTGCACAAC CAGGGTTGCC

151 AGGCGGCGCA ATACGTTTAC GATCCCACCC AAAACTCCCC AAATCGGTCC

201 ATAGGTAGAT CTACGTCAGC TGTCAGCTGG CGCATGGCCC GCGTCAGTGG

251 TTATTGACAA CTCCTTTGGT CGTAATCCTG CCAACATCGT AAAGTAATAA

301 ATTTGCCTGA AAAAGGAAAA TCTGCCGGGG ATCGACTATA AAAATTGGCC

351 CAGCCAGGTA AGCTCCGTAA TGATTGACCC CGACCCTCAG TCCCAGACCC

401 ACGACCGATC CAGGTCCGAT TCCGATCCAT TGCAAGTCGC GATTCCGTCC

451 CGAAACCCAA GCAACCCCCT TAAGACGCTT CTGCCGATGA GCCGGCCAGG

501 CAAAAGCGAA GAATTTGAAA CTAAGGATTT CGCCTTGCAA GGGGGTTGGG

551 TTGTCACGGG GGTTGCGGCA GCTGCGCCGA ACAATTGCCA AGGAGTTCTC

601 CTTAATGAAT TGCAAATCAT TCGCCGGGCA TTGATCAAGT TTGTCCTTGC

651 CAAGGGTGAA ATCTGTGCGA CTAAATGTGC CATCAGCCAG CCACCCAAAA

701 GGGAAACGGA CACAAACGGA ACATCTGTCG CAAGAATTCG CCCTTGTGTC

751 TGCTAGTCTG TTAGTCTGTT ACCTACCCCA CCAAAAGCGA ACTTGACGAG

801 CTTAGTCGTC TGAGGCTGAT AAATGCAGTG CCGCAAAGTA CACCTCGCGA

851 AATTTGTGTA TGAGATGTAT GAAAAATGTA AAAATAGATA GCAATCCGAA

901 CTATGCTCCA TGCTAATGTT CTTCCCAGCC GTTATTACTT GTTACATTGT

951 TTACTTTTAT AACCATCTTG GCCAACAGCT GTTTAGCACC AAATGCCAAC

1001 AAAGAAACAA TTCACCTTTT GGCCCAAGGA TATTCCCGAT CTATTGATGA

1051 ACCTTATGCC TACATGCTCA ATTAACGACT CCATGGGTGT CAGCTGTTTT

1101 TGGCAAAAAT AAGGACAATG TACTGCTCAG ATTTCCGCTG AATACACAAA

1151 CATACATACA TATTTGCCTA GGAAGGTGCT GCGATTTTGG TGCGTGGCAA

1201 TTTTGGGTAC CGAAACAACA GCTGTCGCAC ATTGATTTCT CCGAGTGCAT

1251 TGTCCTGGCC GCAAAACGGG AGACTGTGTG TGTGTGTGCG TAGGACTTTC

1301 ACTCCGTGAA GGGTGGGCGC GCCGACCCAG CTTTCTTGTA CAAAGTGGTG

1351 ATAAACGGCC GGCCGAGCTC GCCCGGGGAT CGAGCGCAGC GGTATAAAAG

1401 GGCGCGGGGT GGCTGAGAGC ATCAGTTGTG AATGAATGTT CGAGCCGAGC

1451 AGACGTGCCG CTGCCTTCGT TAATATCCTT TGAATAAGCC AACTTTGAAT

1501 CACAAGACGC ATACCAAACG GTACCGAAGT TCCTATACTT TCTAGAGAAT

1551 AGGAACTTCG CTAGCAACTT AAAAAAAAAA ATCAAAATGC CCCAGTTCGG

1601 CATCCTGTGC AAGACCCCCC CAAAGGTGCT GGTGCGCCAG TTCGTGGAGC

1651 GCTTCGAGCG CCCCAGCGGC GAGAAGATTG CCCTGTGCGC CGCCGAGCTG

1701 ACCTACCTGT GCTGGATGAT CACCCACAAC GGCACCGCCA TCAAGCGCGC

1751 CACCTTCATG AGCTACAACA CCATCATCAG CAACAGCCTG AGCTTCGACA

1801 TCGTGAACAA GAGCCTGCAG TTCAAGTACA AGACCCAGAA GGCCACCATC

1851 CTGGAGGCCA GCCTGAAGAA GCTGATCCCC GCCTGGGAGT TCACCATCAT

1901 CCCGTACTAC GGCCAGAAGC ACCAGAGCGA TATCACCGAT ATCGTGTCCA

1951 GCCTGCAGCT GCAGTTCGAG AGCAGCGAGG AGGCCGATAA GGGCAACAGC

2001 CACAGCAAGA AGATGCTGAA GGCCCTGCTG AGCGAGGGCG AGAGCATCTG

2051 GGAGATCACC GAGAAGATCC TGAACAGCTT CGAGTACACC AGCCGCTTCA

2101 CCAAGACCAA GACCCTGTAC CAGTTCCTGT TCCTGGCCAC CTTCATCAAC

2151 TGCGGCCGCT TCAGCGATAT CAAGAACGTG GACCCCAAGA GCTTCAAGCT

2201 GGTGCAGAAC AAGTACCTGG GCGTGATTAT CCAGTGCCTG GTGACCGAGA

2251 CCAAGACGAG CGTGTCCCGC CACATCTACT TCTTCAGCGC CCGCGGACGC

2301 ATTGATCCGC TGGTGTACCT GGATGAGTTC CTGCGCAACA GCGAGCCAGT

2351 GCTGAAGCGC GTGAACCGCA CCGGCAACAG CAGCAGCAAC AAGCAGGAGT

2401 ACCAGCTGCT GAAGGATAAC CTGGTGCGCA GCTACAACAA GGCCCTGAAG

2451 AAGAACGCCC CCTACAGCAT CTTCGCCATC AAGAACGGCC CCAAGTCCCA

2501 CATTGGCCGC CACCTGATGA CCTCGTTCCT GAGCATGAAG GGCCTGACCG

2551 AGCTGACGAA CGTGGTGGGC AATTGGAGCG ATAAGCGCGC CAGCGCCGTG

2601 GCCCGCACCA CCTACACCCA CCAGATCACC GCCATCCCCG ATCACTACTT

2651 CGCCCTGGTG TCCCGCTACT ACGCCTACGA TCCCATCAGC AAGGAGATGA

2701 TCGCCCTGAA GGATGAGACC AACCCCATCG AGGAGTGGCA GCACATCGAG

2751 CAGCTGAAGG GCAGCGCCGA GGGCAGCATT CGCTACCCCG CCTGGAACGG

2801 CATCATCAGC CAGGAGGTGC TGGATTACCT GAGCAGCTAC ATCAACCGCC

2851 GCATTGAGGG CCGCGGCAGC CTGCTGACCT GCGGCGATGT GGAGGAGAAC

2901 CCCGGGCCCA TGAGTAACTT GCTCACGGTA CACCAAAATT TGCCGGCCCT

2951 GCCTGTGGAC GCCACGTCCG ACGAGGTCCG AAAGAATCTG ATGGACATGT

3001 TCCGTGACAG GCAGGCCTTT AGCGAGCACA CCTGGAAGAT GCTGCTCTCG

3051 GTGTGCAGGT CCTGGGCCGC TTGGTGCAAG CTGAACAACC GAAAGTGGTT

3101 TCCCGCGGAG CCCGAGGACG TCCGCGATTA TCTGCTGTAC CTGCAGGCTC

3151 GCGGACTGGC CGTCAAAACA ATACAGCAAC ATTTGGGTCA GCTGAACATG

3201 CTCCACAGGC GCAGCGGACT GCCCCGGCCA TCCGATAGCA ATGCAGTGAG

3251 CCTGGTAATG CGTCGAATAC GCAAGGAAAA CGTGGACGCC GGCGAGAGGG

3301 CCAAACAGGC GTTGGCTTTC GAACGCACCG ATTTCGATCA GGTGCGGTCG

3351 TTGATGGAGA ACAGCGATCG ATGCCAGGAC ATCCGGAACT TGGCGTTTTT

3401 GGGCATTGCC TATAACACCC TCCTTCGCAT CGCCGAGATA GCGCGTATCC

3451 GAGTTAAAGA CATTTCCCGT ACAGACGGAG GCCGCATGCT TATTCACATC

3501 GGACGCACGA AAACCCTGGT CAGCACTGCT GGCGTGGAGA AAGCACTTTC

3551 CCTGGGTGTG ACGAAATTGG TCGAGCGATG GATCAGTGTG AGTGGCGTCG

3601 CCGATGACCC CAACAATTAT CTGTTCTGCA GGGTGCGCAA AAATGGAGTG

3651 GCAGCCCCCT GCCTGGTGGG CAGCAGCGAG ATCATCACCC GCAACTACGG

3701 CAAGACCACC ATCAAGGAGG TGGTGGAGAT CTTCGATAAC GATAAGAACA

3751 TCCAGGTGCT GGCCTTCAAC ACCCACACCG ATAACATCGA GTGGGCCCCC

3801 ATCAAGGCCG CCCAGCTGAC CCGCCCCAAC GCCGAGCTGG TGGAGCTGGA

3851 GATCGATACC CTGCACGGCG TGAAGACCAT CCGCTGCACC CCCGATCACC

3901 CCGTGTACAC CAAGAACCGC GGCTACGTGC GCGCCGATGA GCTGACCGAT

3951 GATGATGAGC TGGTGGTGGC CATCTAAACC GGTGAAGTTC CTATACTTTC

4001 TAGAGAATAG GAACTTCGTT TAAACAATCA ACCTCTGGAT TACAAAATTT

4051 GTGAAAGATT GACTGGTATT CTTAACTATG TTGCTCCTTT TACGCTATGT

4101 GGATACGCTG CTTTAATGCC TTTGTATCAT GCTATTGCTT CCCGTATGGC

4151 TTTCATTTTC TCCTCCTTGT ATAAATCCTG GTTGCTGTCT CTTTATGAGG

4201 AGTTGTGGCC CGTTGTCAGG CAACGTGGCG TGGTGTGCAC TGTGTTTGCT

4251 GACGCAACCC CCACTGGTTG GGGCATTGCC ACCACCTGTC AGCTCCTTTC

4301 CGGGACTTTC GCTTTCCCCC TCCCTATTGC CACGGCGGAA CTCATCGCCG

4351 CCTGCCTTGC CCGCTGCTGG ACAGGGGCTC GGCTGTTGGG CACTGACAAT

4401 TCCGTGGTGT TGTCGGGGAA ATCATCGTCC TTTCCTTGGC TGCTCGCCTG

4451 TGTTGCCACC TGGATTCTGC GCGGGACGTC CTTCTGCTAC GTCCCTTCGG

4501 CCCTCAATCC AGCGGACCTT CCTTCCCGCG GCCTGCTGCC GGCTCTGCGG

4551 CCTCTTCCGC GTCTTCGCCT TCGCCCTCAG ACGAGTCGGA TCTCCCTTTG

4601 GGCCGCCTCC CCGCCTGAAG CTTATCGATA CCGTCGACTA AAGCCAAATA

4651 GAAAATTATT CAGTTCCTGG CTTAAGTTTT TAAAAGTGAT ATTATTTATT

4701 TGGTTGTAAC CAACCAAAAG AATGTAAATA ACTAATACAT AATTATGTTA

4751 GTTTTAAGTT AGCAACAAAT TGATTTTAGC TATATTAGCT ACTTGGTTAA

4801 TAAATAGAAT ATATTTATTT AAAGATAATT GCGTTTTTAT TGTCAGGGAG

4851 TGAGTTTGCT TAAAAACTCG TTTAGATCCA CTAGTTCTAG AGCGGCCGCT

4901 GGCCACGGGT GCGCATGATC GTGCTCCTGT CGTTGAGGAC CCGGCTAGGC

4951 TGGCGGGGTT GCCTTACTGG TTAGCAGAAT GAATCACCGA TACGCGAGCG

5001 AACGTGAAGC GACTGCTGCT GCAAAACGTC TGCGACCTGA GCAACAACAT

5051 GAATGGTCTT CGGTTTCCGT GTTTCGTAAA GTCTGGAAAC GCGGAAGTCA

5101 GCGCCCTGCA CCATTATGTT CCGGAGGCGC GCCCTAGTTC CAGTGAAATC

5151 CAAGCATTTT CTAAATTAAA TGTATTCTTA TTATTATAGT TGTTATTTTT

5201 GATATATATA AACAACACTA TTATGCCCAC CATTTTTTTG AGATGCATCT

5251 ACACAAGGAA CAAACACTGG ATGTCACTTT CAGTTCAAAT TGTAACGCTA

5301 ATCACTCCGA ACAGGTCACA AAAAATTACC TTAAAAAGTC ATAATATTAA

5351 ATTAGAATAA ATATAGCTGT GAGGGAAATA TATACAAATA TATTGGAGCA

5401 AATAAATTGT ACATACAAAT ATTTATTACT AATTTCTATT GAGACGAAAT

5451 GAACCACTCG GAACCATTTG AGCGAACCGA ATCGCGCGGA ACTAACGACA

5501 GTCGCTCCAA GGTCGTCGAA CAAAAGGTGA ATGTGTTGCG GAGAGCGGGT

5551 GGGAGACAGC GAAAGAGCAA CTACGAAACG TGGTGTGGTG GAGGTGAATT

5601 ATGAAGAGGG CGCGCGATTT GAAAAGTATG TATATAAAAA ATATATCCCG

5651 GTGTTTTATG TAGCGATAAA CGAGTTTTTG ATGTAAGGTA TGCAGGTGTG

5701 TAAGTCTTTT GGTTAGAAGA CAAATCCAAA GTCTACTTGT GGGGATGTTC

5751 GAAGGGGAAA TACTTGTATT CTATAGGTCA TATCTTGTTT TTATTGGCAC

5801 AAATATAATT ACATTAGCTT TTTGAGGGGG CAATAAACAG TAAACACGAT

5851 GGTAATAATG GTAAAAAAAA AAAACAAGCA GTTATTTCGG ATATATGTCG

5901 GCTACTCCTT GCGTCGGGCC CGAAGTCTTA GAGCCAGATA TGCGAGCACC

5951 CGGAAGCTCA CGATGAGAAT GGCCAGACCC ACGTAGTCCA GCGGCAGATC

6001 GGCGGCGGAG AAGTTAAGCG TCTCCAGGAT GACCTTGCCC GAACTGGGGC

6051 ACGTGGTGTT CGACGATGTG CAGCTAATTT CGCCCGGCTC CACGTCCGCC

6101 CATTGGTTAA TCAGCAGACC CTCGTTGGCG TAACGGAACC ATGAGAGGTA

6151 CGACAACCAT TTGAGGTATA CTGGCACCGA GCCCGAGTTC AAGAAGAAGC

6201 CGCCAAAGAG CAGGAATGGT ATGATAACCG GCGGACCCAC AGACAGCGCC

6251 ATCGAGGTCG AGGAGCTGGC GCAGGATATT AGATATCCGA AGGACGTTGA

6301 CACATTGGCC ACCAGAGTGA CCAGCGCCAG GCAGTTGAAG AAGTGCAGCA

6351 CTCCGGCCCG CAGTCCGATC ATCGGATAGG CAATCGCCGT GAAGACCAGT

6401 GGCACTGTGA GAAAAAGCGG CAATTCGGCA ATCGTTTTGC CCAGAAAGTA

6451 TGTGTCACAG CGATAAAGTC GACTTCGGGC CTCCCTCATA AAAACTGGCA

6501 GCTCTGAGGT GAACACCTAA ATCGAATCGA TTCATTAGAA AGTTAGTAAA

6551 TTATTGAAAT GCAAATGTAT TCTAAACATG ACTTACATTT ATCGTGGCAA

6601 AGACGTTTTG AAAGGTCATG TTGGTCAGGA AGAGGAAGAT GGCTCCGTTG

6651 ATATTCATCA CACCCACTTG CGTGAGTTGT TGGCCCAAAA AGATGAGGCC

6701 AATCAAGATG GCAACCATCT GCAAATTAAA ATGTTACTCG CATCTCATTA

6751 ATATTCGCGA GTTAAATGAA ATTTATTTAT CTTCTGCAAA ACTATAAACT

6801 ATACATCTCA TTGAAAAAAA CTAAGAAGGG TGTGGAATCA GGCAATTCTA

6851 TCTAAAATCT AGCGAATTTG TTTCCAAGAA TTGTAAGCGT TATATCATTT

6901 GTTTCCACTG GAACCACTCA CCGTTGTCTG AATAAGTCGC ACTTTTACGA

6951 GGAGTGGTTC CTTGAGCACC GACAGCCAGG ATCGCCACAG GACCGCCCGG

7001 AACTGCATGA ACCAGGTGGC CTTGTAGGTG TACCCATTCT CCGGCTGCTC

7051 CAGTGGCTTC TCCAGATTTT TGGTGGCCAA CAACTGCTCC ATATCCCGGG

7101 CTACTTTGCT AATGGCAAAA TTGTCGCATA TCTTGGCGAT CCGATCACGG

7151 GACTCGATCT CCCGTCCGGG CACAACGGCC AACACCTGTA CGTAAAAGTC

7201 CGCCGGATTG TAGTTGGTAG GACACTGGGC ACCCACGCTG GATAGGAGTT

7251 GAGATGTTAT GTAATACTAG ATACCCTTAA TAAACACATC GAACTCACTA

7301 GGAAAAGAAG TCGACGGCTT CGCTGGGAGT GCCCAAGAAA GCTACCCTGC

7351 CCTCGGCCAT CAGAAGGATC TTGTCAAAGA GCTCAAACAG CTCGGAAGAC

7401 GGCTGATGAA TGGTCAGGAT GACGGTCTTG CCCTTCTGCG ACAGCTTCTT

7451 CAGCACCTGG ACGACGCTGT GGGCGGTAAA GGAGTCCAGT CCGGAGGTGG

7501 GCTCATCGCA GATCAGAAGC GGCGGATCGG TTAGAGCCTC GGAGGCGAAT

7551 GCCAGACGCT TCCTTTCTCC GCCGGACAGA CCTTTCACCC TGCCGGGCAC

7601 ACCGATGATC GTGTGCTGAC ATTTGCTGAG CGAAAGCTCC TGGATCACCT

7651 GATCCACGCG GGCCACTCGC TGCCGATAGG TCAGATGTCG TGGCATCCGC

7701 ACCATGGCTT GGAAAATCAG GTGTTCCCTG GCCGTTAGGG AGCCGATAAA

7751 GAGGTCATCC TGCTGGACAT AGGCGCACCT GGCCTGCATC TCCTTGGCGT

7801 CCACAGGTTG GCCATTGAGC AGTCGCATCC CGGATGGCGA TACTTGGATG

7851 CCCTGCGGCG ATCGAAAGGC AAGGGCATTC AGCAGGGTCG TCTTTCCGGC

7901 ACCGGAACTG CCCATCACGG CCAAAAGTTC GCCCGGATAG GCCACGCCGC

7951 AAACTGAGTT TCAAATTGGT AATTGGACCC TTTATTAAGA TTTCACACAG

8001 ATCAGCCGAC TGCGAATAGA AACTCACCGT TCTTGAGCAA ATGTTTCCTG

8051 GGCGCCGGTA TGTGTCGCTC GTTGCAGAAT AGTCCGCGTG TCCGGTTGAC

8101 CAGCTGCCGC CATCCGGAGC CCGGCTGATT GACCGCCCCA AAGATGTCCA

8151 TATTGTGCCA GGCATAGGTG AGGTTCTCGG CTAGTTGGCC GCTCCCTGAA

8201 CCGGAGTCCT CCGGCGGACT GGGTGGCCGG AGCGTGCCGT AGTTTTTGGC

8251 CTGCCCGAAG CCCTGGTTAA TGCAGCTCTG CGAAGCCGCT CCGCTGTCAC

8301 CCTGCAATGA TAGGGGATCT CAAATATCAA CTACTAGCGT TATGCTCATC

8351 TAACCCCGAA CAAAAAGTAC CCCGAAGTAT CCTACGAAGT AGGTTTATAC

8401 TTTTATTTAT TTTTTGTGCA TCTAGGATCA GCTTAAAATA TCTGGTTGTT

8451 ATATTTTTTG TAAAAAAGAA TATAGTCGAA AATGAATGCC TTTAGATGTC

8501 TTGATCATGA TATGATCTCA AAAATTGTCT TATATAGCGA GAACAGCTAC

8551 CAGAATAATC TGTTTCGTGT CACTATTTGT TTGTGCAATT GCGGTTTGGG

8601 ATTTTTGTGG GTCGCAGTTC TCACGCCGCA TACAATTTGA TGTTGCAATC

8651 GCAGTTCCTA TAGATCAAGT GAACTTAAGA TGTATGCACA TGTACTACTC

8701 ACATTGTTCA GATGCTCGGC AGATGGGTGT TTGCTGCCTC CGCGAATTAA

8751 TAGCTCCTGA TCCTCTTGGC CCATTGCCGG GATTTTTCAC ACTTTCCCCT

8801 GCTTACCCAC CCAAAACCAA TCACCACCCC AATCACTCAA AAAACAAACA

8851 AAAATAAGAA GCGAGAGGAG TTTTGGCACA GCACTTTGTG TTTAATTGAT

8901 GGCGTAAACC GCTTGGAGCT TCGTCACGAA ACCGCTGACA AAATGCAACT

8951 GAAGGCGGAC ATTGACGCTA CGTAACGCTA CAAACGGTGG CGAAAGAGAT

9001 AGCGGACGCA GCGGCGAAAG AGACGGCGAT ATTTCTGTGG ACAGAGAAGG

9051 AGGCAAACAG CGCTGACTTT GAGTGGAATG TCATTTTGAG TGAGAGGTAA

9101 TCGAAAGAAC CTGGTACATC AAATACCCTT GGATCGAAGT AAATTTAAAA

9151 CTGATCAGAT AAGTTCAATG ATATCCAGTG CAGTAAAAAA AAAAAATGTT

9201 TTTTTTATCT ACTTTCCGCA AAAATGGGTT TTATTAACTT ACATACATAC

9251 TAGGCGCGCC CATATGTTCG GCTTGTCGAC ATGCCCGCCG TGACCGTCGA

9301 GAACCCGCTG ACGCTGCCCC GCGTATCCGC ACCCGCCGAC GCCGTCGCAC

9351 GTCCCGTGCT CACCGTGACC ACCGCGCCCA GCGGTTTCGA GGGCGAGGGC

9401 TTCCCGGTGC GCCGCGCGTT CGCCGGGATC AACTACCGCC ACCTCGACCC

9451 GTTCATCATG ATGGACCAGA TGGGTGAGGT GGAGTACGCG CCCGGGGAGC

9501 CCAAGGGCAC GCCCTGGCAC CCGCACCGCG GCTTCGAGAC CGTGACCTAC

9551 ATCGTCGACG GTAACATGTG AGCAAAAGGC CAGCAAAAGG CCAGGAACCG

9601 TAAAAAGGCC GCGTTGCTGG CGTTTTTCCA TAGGCTCCGC CCCCCTGACG

9651 AGCATCACAA AAATCGACGC TCAAGTCAGA GGTGGCGAAA CCCGACAGGA

9701 CTATAAAGAT ACCAGGCGTT TCCCCCTGGA AGCTCCCTCG TGCGCTCTCC

9751 TGTTCCGACC CTGCCGCTTA CCGGATACCT GTCCGCCTTT CTCCCTTCGG

9801 GAAGCGTGGC GCTTTCTCAT AGCTCACGCT GTAGGTATCT CAGTTCGGTG

9851 TAGGTCGTTC GCTCCAAGCT GGGCTGTGTG CACGAACCCC CCGTTCAGCC

9901 CGACCGCTGC GCCTTATCCG GTAACTATCG TCTTGAGTCC AACCCGGTAA

9951 GACACGACTT ATCGCCACTG GCAGCAGCCA CTGGTAACAG GATTAGCAGA

10001 GCGAGGTATG TAGGCGGTGC TACAGAGTTC TTGAAGTGGT GGCCTAACTA

10051 CGGCTACACT AGAAGAACAG TATTTGGTAT CTGCGCTCTG CTGAAGCCAG

10101 TTACCTTCGG AAAAAGAGTT GGTAGCTCTT GATCCGGCAA ACAAACCACC

10151 GCTGGTAGCG GTGGTTTTTT TGTTTGCAAG CAGCAGATTA CGCGCAGAAA

10201 AAAAGGATCT CAAGAAGATC CTTTGATCTT TTCTACGGGG TCTGACGCTC

10251 AGTGGAACGA AAACTCACGT TAAGGGATTT TGGTCATGAG ATTATCAAAA

10301 AGGATCTTCA CCTAGATCCT TTTAAATTAA AAATGAAGTT TTAAATCAAT

10351 CTAAAGTATA TATGAGTAAA CTTGGTCTGA CAGTTACCAA TGCTTAATCA

10401 GTGAGGCACC TATCTCAGCG ATCTGTCTAT TTCGTTCATC CATAGTTGCC

10451 TGACTCCCCG TCGTGTAGAT AACTACGATA CGGGAGGGCT TACCATCTGG

10501 CCCCAGTGCT GCAATGATAC CGCGAGACCC ACGCTCACCG GCTCCAGATT

10551 TATCAGCAAT AAACCAGCCA GCCGGAAGGG CCGAGCGCAG AAGTGGTCCT

10601 GCAACTTTAT CCGCCTCCAT CCAGTCTATT AATTGTTGCC GGGAAGCTAG

10651 AGTAAGTAGT TCGCCAGTTA ATAGTTTGCG CAACGTTGTT GCCATTGCTA

10701 CAGGCATCGT GGTGTCACGC TCGTCGTTTG GTATGGCTTC ATTCAGCTCC

10751 GGTTCCCAAC GATCAAGGCG AGTTACATGA TCCCCCATGT TGTGCAAAAA

10801 AGCGGTTAGC TCCTTCGGTC CTCCGATCGT TGTCAGAAGT AAGTTGGCCG

10851 CAGTGTTATC ACTCATGGTT ATGGCAGCAC TGCATAATTC TCTTACTGTC

10901 ATGCCATCCG TAAGATGCTT TTCTGTGACT GGTGAGTACT CAACCAAGTC

10951 ATTCTGAGAA TAGTGTATGC GGCGACCGAG TTGCTCTTGC CCGGCGTCAA

11001 TACGGGATAA TACCGCGCCA CATAGCAGAA CTTTAAAAGT GCTCATCATT

11051 GGAAAACGTT CTTCGGGGCG AAAACTCTCA AGGATCTTAC CGCTGTTGAG

11101 ATCCAGTTCG ATGTAACCCA CTCGTGCACC CAACTGATCT TCAGCATCTT

11151 TTACTTTCAC CAGCGTTTCT GGGTGAGCAA AAACAGGAAG GCAAAATGCC

11201 GCAAAAAAGG GAATAAGGGC GACACGGAAA TGTTGAATAC TCATACTCTT

11251 CCTTTTTCAA TATTATTGAA GCATTTATCA GGGTTATTGT CTCATGAGCG

11301 GATACATATT TGAATGTATT TAGAAAAATA AACAAATAGG GGTTCCGCGC

11351 ACATTTCCCC GAAAAGTGCC ACCTGAGTCT

**HJP-472-actin5C-FloxSyn21Gal80-10XUAS-EGFP-Kir2.1**

1 TAAGAAACCA TTATTATCAT GACATTAACC TATAAAAATA GGCGTATCAC

51 GAGGCCCTTT CGTCTTCAAG AATTCGTTTA TCACAAGTTT GTACAAAAAA

101 GCAGGCTCCG CGGCCGCAAT TCTATATTCT AAAAACACAA ATGATACTTC

151 TAAAAAAAAA TCATGAATGG CATCAACTCT GAATCAAATC TTTGCAGATG

201 CACCTACTTC TCATTTCCAC TGTCACATCA TTTTTCCAGA TCTCGCTGCC

251 TGTTATGTGG CCCACAAACC AAGACACGTT TTATGGCCAT TAAAGCTGGC

301 TGATCGTCGC CAAACACCAA ATACATAATG AATATGTACA CATTCGAGAA

351 AGAAGCGATC AAAGAAGCGT CTTCGGGCGG AGTAGGAGAA TGCGGAGGAG

401 AAGGAGAACG AGCTGATCTA GTATCTCTCC ACAATCCAAT GCCAACTGAC

451 CAACTGGCCA TATTCGGAGC AATTTGAAGC CAATTTCCAT CGCCTGGCGA

501 TCGCTCCATT CTTGGCTATA TGTTTTTCAC CGTTACCCGG GGCCATTTTC

551 AAAGACTCGT CGGCAAGATA AGATTGTGTC ACTCGCTGTC TCTCTTCATT

601 TGTCGAAGAA TGCTGAGGAA TTTCGCGATG ACGTCGGCGA GTATTTTGAA

651 GAATGAGAAT AATTTGTATT TATACGAAAA TCAGTTAGTG GAATTTTCTA

701 CAAAAACATG TTATCTATAG ATAATTTTGT TGCAAAATAT GTTGACTATG

751 ACAAAGATTG TATGTATATA CCTTTAATGT ATTCTCATTT TCTTATGTAT

801 TTATAATGGC AATGATGATA CTGATGATAT TTTAAGATGA TGCCAGACCA

851 AAAGGCTTGA ATTTCTGCGT CTTTTGCCGA ACGCAGTGCA TGTGCAATTG

901 TTGTTTTTTG GAATATTCAA TTTTCGGACT GTCCGCTTTG ATTTCAGTTT

951 CTTGGCTTAT TCAAAAAGCA AAGTAAAGCC AAAAAAGCGA GATGGCAATA

1001 CCAAATGCGG CAAAACGGTA GTGGAAGGAA AGGGGTGCGG GGCAGCGGAA

1051 GGAAGGGTGG GGCGGGGCGT GGCGGGGTCT GTGGCTGGGC GCGACGTCAC

1101 CGACGTTGGA GCCACTCCTT TGACCATGTG TGCGTGTGTG TATTATTCGT

1151 GTCTCGCCAC TCGCCGGTTG TTTTTTTCTT TTTATGCTGC GCTCTCTCTA

1201 GCGCCATCTC GCTTACGCAT GCTCAACGCA CCGCATGTTG CCGTTTCCTT

1251 TTATGCGTCA TTTTGGCTCG AAATAGGCAA TTATTTAAAC AAAGATTAGT

1301 CAACGAAAAC GCTAAAATAA ATAAGTCTAC AATATGGTTA CTTATTGCCA

1351 TGTGTGTGCA GCCAACGATA GCAACAAAAG CAACAACACA GGTGGCTTTC

1401 CCTCTTTCAC TTTTTGTTTG CAAGCCGCGT GCGAGCAAGA CGGCACGACC

1451 GGCAAACGCA ATTACGCTGA CAAAGAGCAG ACGAAGTTTT GGCGAAAAAC

1501 ATCAAGGCGC CTGATACGAA TGCATTTGCA ATAACAATTG CGATATTTAA

1551 TATTGTTTAT GAAGCTGTTT GACTTCAAAA CACACAAAAA AAAAAATAAA

1601 ACAAATTATT TGAAAGAGAA TTAGGAATCG GACGCTTATC GTTAGGGTAA

1651 CAACAAGAAA TGCTTACTGA GTCACAGCCT CTGGAAAACT GCCGCAAGCC

1701 AGAGAGAGAG AGAAAAAGAG GGAGAGCAGC TTAGACCGCA TGTGCTTGTG

1751 TGTGAGGCGT CTCTCTCTTC GTCTCTGTTG CGCAAACGCA TAGACTGCAC

1801 TGAAAAAATC GATTACCTAT TTTTTATGAA TGAATATTTG CACTATTACT

1851 ATTCAAAACT ATTAAGATAG CAATCACATT CAATAGCCAA ATACTATACC

1901 ACCTGAGCGA TGCAACGAAA TGATCAATTT GAGCAAAAAT GCTGCATATT

1951 TAGGACGGCA TCATTATAGA AATGCTTCTT GCTGTGTACT TTTCTCTCGT

2001 CTGGCAGCTG TTTCGCCGTT ATTGTTAAAA CCGGCTTAAG TTAGGTGTGT

2051 TTTCTACGAC TAGTGAATGC CCTACTAGAA GATGTGTGTT GCACAAAATG

2101 TCCCTGGAAT AACCAATTTG AAGTGCAGAT AGCAGTAAAC GTAAGCTAAT

2151 ATGAATATTA TTTAACTGTA ATGTTTTAAT ATCGCTGGAC ATTACTAATA

2201 AACCCACTAT AAACACATGT ACATATGTAT GTTTTGGCAT ACAATGAGTA

2251 GTTGGGGAAA AAATGTGTAA AAGCACCGTG ACCATCACAG CATAAAGATA

2301 ACCAGCTGAA GTATCGAATA TGAGTAACCC CCAAATTGAA TCACATGCCG

2351 CAACTGATAG GACCCATGGA AGTACACTCT TCATGGCGAT ATACAAGACA

2401 CACACAAGCA CGAACACCCA GTTGCGGAGG AAATTCTCCG TAAATGAAAA

2451 CCCAATCGGC GAACAATTCA TACCCATATA TGGTAAAAGT TTTGAACGCG

2501 ACTTGAGAGC GGAGAGCATT GCGGCTGATA AGGTTTTAGC GCTAAGCGGG

2551 CTTTATAAAA CGGGCTGCGG GACCAGTTTT CATATCACTA CCGTTTGAGT

2601 TCTTGTGCTG TGTGGATACT CCTCCCGACA CAAAGCCGCT CCATCAGCCA

2651 GCAGTCGTCT AATCCAGAGA CACCAAACCG AAAGACTTAA TTTATATTTA

2701 TTTAATTAAT TTTAATAAAA CACACCAAAT GTAAGTAGCT TTCCCCTTCC

2751 CAACAACAAA ACACCATCGA ACCACTCCCA CCAAGAAAAA GCAATAATCG

2801 AGAAAAGCCG CGGAAAATGT GTGATTTTTT TTGTAAACAA AACATTTTTT

2851 TATGTGCCAG TGCTGAAAGT GATCAAAAAA TACTAGCCAC GAGCTAAAGA

2901 GTTATTGTAT TGACCAAAAC TCCAAAAATA CCCAAGTTTG GCCCTAAATT

2951 GTCAATCAAA ATACCAATAG GTCGAAAGAC ATCAAAATTA ACAAAACCAG

3001 GGTTTCAAAT ACCATAACTC AAGAATCAGG ATTACAACTG CAGATTTCAG

3051 GATATATACA TACAAATTAT ACGAAATTAT AAAAACCAAA GCAATTCAAT

3101 AGCCCCAACT CAAATGTTAG GATCTAATAT AGTGTTTAAA GCCAAGCTCG

3151 CTGATGTGGG CGTGTCACGA TTTCACCCAA AGATATGCCA AATTACGAAT

3201 TGCAAATCAA TTCGCCAACA CTTTCTTTTT TTCCCACGCC CTAAAACACC

3251 AGATCATCCA TAAATGTACA TACATACAGT ATATGCATAT TATAATCTGT

3301 AAAACTAGAT CAGGTTCTTG AAAATAGTGA CGTAGGCAGC CGTTTTGGCT

3351 GAAGCAGAAA TTTTTGCCGG TTTTTCAAAG TTGTAGTTGC AAAAATGGAG

3401 AAAACCTTCG AGCATTCGTT CATATACACA CACTCACGCG CAAAATAACG

3451 AGAGAGAGTG TATGTGTGTG TGAGAGAGCG AAAGCCAGAC GACGGTTTGC

3501 TTTTCGCCTC GAAACATGAC CATATATGGT CACAAAACTT GGCCGCCGCA

3551 ATTCAACACA CCAGCGCTCT CCTTCGCACC CATAGCGACC ATGGCGCGGA

3601 GCGAGCGAGA TGGCGAGAGC GAGCGACGCC TATGGCGACG TCGACGCAGG

3651 CAGCGATTGA AAAACGCAGT TAACTGGCAT TCAACATTCA CCAGCCACTT

3701 TCAGTCGGTT TATTCCAGTC ATTCCTTTCA AACCGTGCGG TCGCTTAGCT

3751 CAGCCTCGCC ACTTGCGTTT ACAGTAGTTT TCACGCCTTG AATTTGTTAA

3801 ATCGAACAAA AAGGTAAAGT TTAACTAGCT TTGAAAAGTT TCGTGGCTCT

3851 TAATTGTTAA ATTTTCTAGA GTGCGTTTAG TGTTTTTTTT TTTTTTTATT

3901 TTGTAATGTT AATTTCGGGT TCCAATTCGA GTTTTAGGCA GCCGCCATTT

3951 TAAGGGCGCA TACACACAGG CAACTGTGCT CTCTTTGCGG CTTTCTTTTG

4001 CACCGGCATT CGTTAAGTGT CGTCTAGAAG CTTCTCCCCT CCCTTTTCGG

4051 CATATTCGTA TTGTGGTTTT AATTTTTCGG GGCGGGGCCT TCTATTTTGT

4101 AACTGTTCTT TTAATTTCTT ATTACAATTC GATCGCAAGT GAAAATCAGT

4151 TTTCAATCGG AAAAGTATTT TTTTATGAAA TTTTTTTTTG TCCAAGATTA

4201 AAATTTTGTA CTAAAAAAAC GTACATTGCA TTGAGTGATT TTTAATTGTA

4251 CACGAAAAAC AAGTTAGTTT GTTATGACAA TTGTACTTTG GTAGACCAGC

4301 GCAGTCCAAG GAAACCACGC AAATTCTCAG TTTTTTTTTT GCCATTTCTA

4351 CATTACCAAA TAAGGTAACC AAAAACTAAT GGGAAATCCG CATTCTTTCC

4401 ATTGCAGCTT ACAGGATCGA TCCCCGGGTA CCGCGCCGAC CCAGCTTTCT

4451 TGTACAAAGT GGTGATAAAC GGCCGGCATA ACTTCGTATA ATGTATGCTA

4501 TACGAAGTTA TCCTGCAGGA ACTTAAAAAA AAAAATCAAA ATGGATTACA

4551 ACAAAAGGAG TAGTGTGAGT ACGGTGCCGA ATGCTGCTCC CATTCGCGTG

4601 GGCTTCGTGG GATTGAACGC GGCTAAGGGT TGGGCCATTA AAACGCATTA

4651 TCCAGCCATA CTGCAGCTGA GCTCCCAGTT CCAAATAACA GCGCTTTATT

4701 CCCCCAAGAT CGAGACGTCC ATTGCGACAA TTCAGAGGCT GAAGTTGTCC

4751 AACGCCACAG CTTTCCCAAC ACTCGAGAGC TTCGCCTCGA GCTCCACGAT

4801 CGATATGATT GTGATCGCAA TCCAGGTGGC TTCCCACTAC GAGGTGGTAA

4851 TGCCCCTGCT CGAGTTTTCG AAGAACAATC CTAATCTGAA GTATTTGTTT

4901 GTGGAATGGG CACTGGCCTG CTCGCTCGAT CAGGCGGAGT CGATCTATAA

4951 GGCCGCTGCA GAGCGGGGCG TGCAAACAAT AATCAGTCTG CAGGGACGAA

5001 AGAGCCCGTA CATTTTGCGG GCAAAAGAGC TTATCTCCCA GGGCTACATC

5051 GGCGATATAA ATAGCATCGA GATTGCAGGT AACGGAGGTT GGTACGGTTA

5101 CGAGCGGCCG GTGAAAAGCC CGAAATACAT TTACGAGATC GGAAACGGAG

5151 TTGATCTGGT GACCACCACG TTCGGTCACA CGATAGATAT ATTGCAGTAC

5201 ATGACCAGTT CGTACTTCAG CCGTATCAAT GCCATGGTGT TTAACAACAT

5251 TCCAGAACAG GAGCTCATTG ATGAGAGGGG CAATCGCCTG GGCCAGCGGG

5301 TCCCAAAGAC TGTCCCAGAT CATCTCTTGT TCCAGGGCAC GCTGCTCAAC

5351 GGAAATGTTC CTGTGTCCTG TTCCTTTAAG GGAGGCAAGC CTACGAAAAA

5401 GTTCACCAAG AATCTGGTCA TAGATATCCA TGGTACAAAG GGTGATCTGA

5451 AGCTGGAGGG TGACGCTGGA TTTGCGGAAA TCTCGAATCT CGTTCTGTAT

5501 TATTCGGGCA CCCGCGCCAA TGATTTTCCC CTGGCCAATG GTCAGCAGGC

5551 ACCCTTGGAC CCCGGCTACG ACGCAGGCAA GGAGATTATG GAGGTGTATC

5601 ACCTCCGAAA CTACAACGCG ATCGTTGGTA ATATACACCG GCTCTACCAG

5651 AGTATCAGCG ATTTCCACTT CAACACCAAG AAAATCCCCG AGCTTCCATC

5701 GCAGTTCGTA ATGCAAGGCT TCGATTTCGA AGGCTTTCCC ACCCTGATGG

5751 ATGCCTTGAT CTTGCATCGC CTGATTGAAA GTGTCTACAA GTCGAATATG

5801 ATGGGCAGTA CCCTGAATGT TTCCAACATC TCCCACTACT CGCTTTAAAC

5851 GCGTAGGATC TTTGTGAAGG AACCTTACTT CTGTGGTGTG ACATAATTGG

5901 ACAAACTACC TACAGAGATT TAAAGCTCTA AGGTAAATAT AAAATTTTTA

5951 AGTGTATAAT GTGTTAAACT ACTGATTCTA ATTGTTTGTG TATTTTAGAT

6001 TCCAACCTAT GGAACTGATG AATGGGAGCA GTGGTGGAAT GCCTTTAATG

6051 AGGAAAACCT GTTTTGCTCA GAAGAAATGC CATCTAGTGA TGATGAGGCT

6101 ACTGCTGACT CTCAACATTC TACTCCTCCA AAAAAGAAGA GAAAGGTAGA

6151 AGACCCCAAG GACTTTCCTT CAGAATTGCT AAGTTTTTTG AGTCATGCTG

6201 TGTTTAGTAA TAGAACTCTT GCTTGCTTTG CTATTTACAC CACAAAGGAA

6251 AAAGCTGCAC TGCTATACAA GAAAATTATG GAAAAATATT TGATGTATAG

6301 TGCCTTGACT AGAGATCATA ATCAGCCATA CCACATTTGT AGAGGTTTTA

6351 CTTGCTTTAA AAAACCTCCC ACACCTCCCC CTGAACCTGA AACATAAAAT

6401 GAATGCAATT GTTGTTGTTA ACTTGTTTAT TGCAGCTTAT AATGGTTACA

6451 AATAAAGCAA TAGCATCACA AATTTCACAA ATAAAGCATT TTTTTCACTG

6501 CATTCTAGTT GTGGTTTGTC CAAACTCATC AATGTATCTT ATCATGTCTG

6551 GATCCCTAGG ATAACTTCGT ATAATGTATG CTATACGAAG TTATGGTACC

6601 GCTAGTTGGC CACGGGTGCG CATGATCGTG CTCCTGTCGT TGAGGACCCG

6651 GCTAGGCTGG CGGGGTTGCC TTACTGGTTA GCAGAATGAA TCACCGATAC

6701 GCGAGCGAAC GTGAAGCGAC TGCTGCTGCA AAACGTCTGC GACCTGAGCA

6751 ACAACATGAA TGGTCTTCGG TTTCCGTGTT TCGTAAAGTC TGGAAACGCG

6801 GAAGTCAGCG CCCTGCACCA TTATGTTCCG GACTAGTTCC AGTGAAATCC

6851 AAGCATTTTC TAAATTAAAT GTATTCTTAT TATTATAGTT GTTATTTTTG

6901 ATATATATAA ACAACACTAT TATGCCCACC ATTTTTTTGA GATGCATCTA

6951 CACAAGGAAC AAACACTGGA TGTCACTTTC AGTTCAAATT GTAACGCTAA

7001 TCACTCCGAA CAGGTCACAA AAAATTACCT TAAAAAGTCA TAATATTAAA

7051 TTAGAATAAA TATAGCTGTG AGGGAAATAT ATACAAATAT ATTGGAGCAA

7101 ATAAATTGTA CATACAAATA TTTATTACTA ATTTCTATTG AGACGAAATG

7151 AACCACTCGG AACCATTTGA GCGAACCGAA TCGCGCGGAA CTAACGACAG

7201 TCGCTCCAAG GTCGTCGAAC AAAAGGTGAA TGTGTTGCGG AGAGCGGGTG

7251 GGAGACAGCG AAAGAGCAAC TACGAAACGT GGTGTGGTGG AGGTGAATTA

7301 TGAAGAGGGC GCGCGATTTG AAAAGTATGT ATATAAAAAA TATATCCCGG

7351 TGTTTTATGT AGCGATAAAC GAGTTTTTGA TGTAAGGTAT GCAGGTGTGT

7401 AAGTCTTTTG GTTAGAAGAC AAATCCAAAG TCTACTTGTG GGGATGTTCG

7451 AAGGGGAAAT ACTTGTATTC TATAGGTCAT ATCTTGTTTT TATTGGCACA

7501 AATATAATTA CATTAGCTTT TTGAGGGGGC AATAAACAGT AAACACGATG

7551 GTAATAATGG TAAAAAAAAA AAACAAGCAG TTATTTCGGA TATATGTCGG

7601 CTACTCCTTG CGTCGGGCCC GAAGTCTTAG AGCCAGATAT GCGAGCACCC

7651 GGAAGCTCAC GATGAGAATG GCCAGACCCA CGTAGTCCAG CGGCAGATCG

7701 GCGGCGGAGA AGTTAAGCGT CTCCAGGATG ACCTTGCCCG AACTGGGGCA

7751 CGTGGTGTTC GACGATGTGC AGCTAATTTC GCCCGGCTCC ACGTCCGCCC

7801 ATTGGTTAAT CAGCAGACCC TCGTTGGCGT AACGGAACCA TGAGAGGTAC

7851 GACAACCATT TGAGGTATAC TGGCACCGAG CCCGAGTTCA AGAAGAAGCC

7901 GCCAAAGAGC AGGAATGGTA TGATAACCGG CGGACCCACA GACAGCGCCA

7951 TCGAGGTCGA GGAGCTGGCG CAGGATATTA GATATCCGAA GGACGTTGAC

8001 ACATTGGCCA CCAGAGTGAC CAGCGCCAGG CAGTTGAAGA AGTGCAGCAC

8051 TCCGGCCCGC AGTCCGATCA TCGGATAGGC AATCGCCGTG AAGACCAGTG

8101 GCACTGTGAG AAAAAGCGGC AATTCGGCAA TCGTTTTGCC CAGAAAGTAT

8151 GTGTCACAGC GATAAAGTCG ACTTCGGGCC TCCCTCATAA AAACTGGCAG

8201 CTCTGAGGTG AACACCTAAA TCGAATCGAT TCATTAGAAA GTTAGTAAAT

8251 TATTGAAATG CAAATGTATT CTAAACATGA CTTACATTTA TCGTGGCAAA

8301 GACGTTTTGA AAGGTCATGT TGGTCAGGAA GAGGAAGATG GCTCCGTTGA

8351 TATTCATCAC ACCCACTTGC GTGAGTTGTT GGCCCAAAAA GATGAGGCCA

8401 ATCAAGATGG CAACCATCTG CAAATTAAAA TGTTACTCGC ATCTCATTAA

8451 TATTCGCGAG TTAAATGAAA TTTATTTATC TTCTGCAAAA CTATAAACTA

8501 TACATCTCAT TGAAAAAAAC TAAGAAGGGT GTGGAATCAG GCAATTCTAT

8551 CTAAAATCTA GCGAATTTGT TTCCAAGAAT TGTAAGCGTT ATATCATTTG

8601 TTTCCACTGG AACCACTCAC CGTTGTCTGA ATAAGTCGCA CTTTTACGAG

8651 GAGTGGTTCC TTGAGCACCG ACAGCCAGGA TCGCCACAGG ACCGCCCGGA

8701 ACTGCATGAA CCAGGTGGCC TTGTAGGTGT ACCCATTCTC CGGCTGCTCC

8751 AGTGGCTTCT CCAGATTTTT GGTGGCCAAC AACTGCTCCA TATCCCGGGC

8801 TACTTTGCTA ATGGCAAAAT TGTCGCATAT CTTGGCGATC CGATCACGGG

8851 ACTCGATCTC CCGTCCGGGC ACAACGGCCA ACACCTGTAC GTAAAAGTCC

8901 GCCGGATTGT AGTTGGTAGG ACACTGGGCA CCCACGCTGG ATAGGAGTTG

8951 AGATGTTATG TAATACTAGA TACCCTTAAT AAACACATCG AACTCACTAG

9001 GAAAAGAAGT CGACGGCTTC GCTGGGAGTG CCCAAGAAAG CTACCCTGCC

9051 CTCGGCCATC AGAAGGATCT TGTCAAAGAG CTCAAACAGC TCGGAAGACG

9101 GCTGATGAAT GGTCAGGATG ACGGTCTTGC CCTTCTGCGA CAGCTTCTTC

9151 AGCACCTGGA CGACGCTGTG GGCGGTAAAG GAGTCCAGTC CGGAGGTGGG

9201 CTCATCGCAG ATCAGAAGCG GCGGATCGGT TAGAGCCTCG GAGGCGAATG

9251 CCAGACGCTT CCTTTCTCCG CCGGACAGAC CTTTCACCCT GCCGGGCACA

9301 CCGATGATCG TGTGCTGACA TTTGCTGAGC GAAAGCTCCT GGATCACCTG

9351 ATCCACGCGG GCCACTCGCT GCCGATAGGT CAGATGTCGT GGCATCCGCA

9401 CCATGGCTTG GAAAATCAGG TGTTCCCTGG CCGTTAGGGA GCCGATAAAG

9451 AGGTCATCCT GCTGGACATA GGCGCACCTG GCCTGCATCT CCTTGGCGTC

9501 CACAGGTTGG CCATTGAGCA GTCGCATCCC GGATGGCGAT ACTTGGATGC

9551 CCTGCGGCGA TCGAAAGGCA AGGGCATTCA GCAGGGTCGT CTTTCCGGCA

9601 CCGGAACTGC CCATCACGGC CAAAAGTTCG CCCGGATAGG CCACGCCGCA

9651 AACTGAGTTT CAAATTGGTA ATTGGACCCT TTATTAAGAT TTCACACAGA

9701 TCAGCCGACT GCGAATAGAA ACTCACCGTT CTTGAGCAAA TGTTTCCTGG

9751 GCGCCGGTAT GTGTCGCTCG TTGCAGAATA GTCCGCGTGT CCGGTTGACC

9801 AGCTGCCGCC ATCCGGAGCC CGGCTGATTG ACCGCCCCAA AGATGTCCAT

9851 ATTGTGCCAG GCATAGGTGA GGTTCTCGGC TAGTTGGCCG CTCCCTGAAC

9901 CGGAGTCCTC CGGCGGACTG GGTGGCCGGA GCGTGCCGTA GTTTTTGGCC

9951 TGCCCGAAGC CCTGGTTAAT GCAGCTCTGC GAAGCCGCTC CGCTGTCACC

10001 CTGCAATGAT AGGGGATCTC AAATATCAAC TACTAGCGTT ATGCTCATCT

10051 AACCCCGAAC AAAAAGTACC CCGAAGTATC CTACGAAGTA GGTTTATACT

10101 TTTATTTATT TTTTGTGCAT CTAGGATCAG CTTAAAATAT CTGGTTGTTA

10151 TATTTTTTGT AAAAAAGAAT ATAGTCGAAA ATGAATGCCT TTAGATGTCT

10201 TGATCATGAT ATGATCTCAA AAATTGTCTT ATATAGCGAG AACAGCTACC

10251 AGAATAATCT GTTTCGTGTC ACTATTTGTT TGTGCAATTG CGGTTTGGGA

10301 TTTTTGTGGG TCGCAGTTCT CACGCCGCAT ACAATTTGAT GTTGCAATCG

10351 CAGTTCCTAT AGATCAAGTG AACTTAAGAT GTATGCACAT GTACTACTCA

10401 CATTGTTCAG ATGCTCGGCA GATGGGTGTT TGCTGCCTCC GCGAATTAAT

10451 AGCTCCTGAT CCTCTTGGCC CATTGCCGGG ATTTTTCACA CTTTCCCCTG

10501 CTTACCCACC CAAAACCAAT CACCACCCCA ATCACTCAAA AAACAAACAA

10551 AAATAAGAAG CGAGAGGAGT TTTGGCACAG CACTTTGTGT TTAATTGATG

10601 GCGTAAACCG CTTGGAGCTT CGTCACGAAA CCGCTGACAA AATGCAACTG

10651 AAGGCGGACA TTGACGCTAC GTAACGCTAC AAACGGTGGC GAAAGAGATA

10701 GCGGACGCAG CGGCGAAAGA GACGGCGATA TTTCTGTGGA CAGAGAAGGA

10751 GGCAAACAGC GCTGACTTTG AGTGGAATGT CATTTTGAGT GAGAGGTAAT

10801 CGAAAGAACC TGGTACATCA AATACCCTTG GATCGAAGTA AATTTAAAAC

10851 TGATCAGATA AGTTCAATGA TATCCAGTGC AGTAAAAAAA AAAAATGTTT

10901 TTTTTATCTA CTTTCCGCAA AAATGGGTTT TATTAACTTA CATACATACT

10951 AGGCGCGCCC ATAAGCTTGC ATGCCTGCAG GTCGGAGTAC TGTCCTCCGA

11001 GCGGAGTACT GTCCTCCGAG CGGAGTACTG TCCTCCGAGC GGAGTACTGT

11051 CCTCCGAGCG GAGTACTGTC CTCCGAGCGG AGACTCTAGC CCTAGGGCAT

11101 GCCTGCAGGT CGGAGTACTG TCCTCCGAGC GGAGTACTGT CCTCCGAGCG

11151 GAGTACTGTC CTCCGAGCGG AGTACTGTCC TCCGAGCGGA GTACTGTCCT

11201 CCGAGCGGAG ACTCTAGCGC TAGCGACGTC GAGCGCCGGA GTATAAATAG

11251 AGGCGCTTCG TCTACGGAGC GACAATTCAA TTCAAACAAG CAAAGTGAAC

11301 ACGTCGCTAA GCGAAAGCTA AGCAAATAAA CAAGCGCAGC TGAACAAGCT

11351 AAACAATCTG CAGTAAAGTG CAAGTTAAAG TGAATCAATT AAAAGTAACC

11401 AGCAACCAAG TAAATCAACT GCAACTACTG AAATCTGCCA AGAAGTAATT

11451 ATTGAATACA AGAAGAGAAC TCTGAATAGA TCTAAAAGGT AGGTTCAACC

11501 ACTGATGCCT AGGCACACCG AAACGACTAA CCCTAATTCT TATCCTTTAC

11551 TTCAGGAACT TAAAAAAAAA AATCAAAATG GTGAGCAAGG GCGAGGAGCT

11601 GTTCACCGGG GTGGTGCCCA TCCTGGTCGA GCTGGACGGC GACGTAAACG

11651 GCCACAAGTT CAGCGTGTCC GGCGAGGGCG AGGGCGATGC CACCTACGGC

11701 AAGCTGACCC TGAAGTTCAT CTGCACCACC GGCAAGCTGC CCGTGCCCTG

11751 GCCCACCCTC GTGACCACCC TGACCTACGG CGTGCAGTGC TTCAGCCGCT

11801 ACCCCGACCA CATGAAGCAG CACGACTTCT TCAAGTCCGC CATGCCCGAA

11851 GGCTACGTCC AGGAGCGCAC CATCTTCTTC AAGGACGACG GCAACTACAA

11901 GACCCGCGCC GAGGTGAAGT TCGAGGGCGA CACCCTGGTG AACCGCATCG

11951 AGCTGAAGGG CATCGACTTC AAGGAGGACG GCAACATCCT GGGGCACAAG

12001 CTGGAGTACA ACTACAACAA CCACAACGTC TATATCATGG CCGACAAGCA

12051 GAAGAACGGC ATCAAGGTGA ACTTCAAGAT CCGCCACAAC ATCGAGGACG

12101 GCAGCGTGCA GCTCGCCGAC CACTACCAGC AGAACACCCC CATCGGCGAC

12151 GGCCCCGTGC TGCTGCCCGA CAACCACTAC CTGAGCACCC AGTCCGCCCT

12201 GAGCAAAGAC CCCAACGAGA AGCGCGATCA CATGGTCCTG CTGGAGTTCG

12251 TGACCGCCGC CGGGATCACT CTCGGCATGG ACGAGCTGTA CAAGTACTCA

12301 GATCTCGAGG CCATGGGCAG TGTGAGAACC AACCGCTACA GCATCGTCTC

12351 TTCAGAAGAA GACGGTATGA AGTTGGCCAC CATGGCAGTT GCAAATGGCT

12401 TTGGGAACGG GAAGAGTAAA GTCCACACCC GACAACAGTG CAGGAGCCGC

12451 TTTGTGAAGA AAGATGGCCA CTGTAATGTT CAGTTCATCA ATGTGGGTGA

12501 GAAGGGGCAA CGGTACCTCG CAGACATCTT CACCACGTGT GTGGACATTC

12551 GCTGGCGGTG GATGCTGGTT ATCTTCTGCC TGGCTTTCGT CCTGTCATGG

12601 CTGTTTTTTG GCTGTGTGTT TTGGTTGATA GCTCTGCTCC ATGGGGACCT

12651 GGATGCATCC AAAGAGGGCA AAGCTTGTGT GTCCGAGGTC AACAGCTTCA

12701 CGGCTGCCTT CCTCTTCTCC ATTGAGACCC AGACAACCAT AGGCTATGGT

12751 TTCAGATGTG TCACGGATGA ATGCCCAATT GCTGTTTTCA TGGTGGTGTT

12801 CCAGTCAATC GTGGGCTGCA TCATCGATGC TTTCATCATT GGCGCAGTCA

12851 TGGCCAAGAT GGCAAAGCCA AAGAAGAGAA ACGAGACTCT TGTCTTCAGT

12901 CACAATGCCG TGATTGCCAT GAGAGACGGC AAGCTGTGTT TGATGTGGCG

12951 AGTGGGCAAT CTTCGGAAAA GCCACTTGGT GGAAGCTCAT GTTCGAGCAC

13001 AGCTCCTCAA ATCCAGAATT ACTTCTGAAG GGGAGTATAT CCCTCTGGAT

13051 CAAATAGACA TCAATGTTGG GTTTGACAGT GGAATCGATC GTATATTTCT

13101 GGTGTCCCCA ATCACTATAG TCCATGAAAT AGATGAAGAC AGTCCTTTAT

13151 ATGATTTGAG TAAACAGGAC ATTGACAACG CAGACTTTGA AATCGTGGTC

13201 ATACTGGAAG GCATGGTGGA AGCCACTGCC ATGACGACAC AGTGCCGTAG

13251 CTCTTATCTA GCAAATGAAA TCCTGTGGGG CCACCGCTAT GAGCCTGTGC

13301 TCTTTGAAGA GAAGCACTAC TACAAAGTGG ACTATTCCAG GTTCCACAAA

13351 ACTTACGAAG TCCCCAACAC TCCCCTTTGT AGTGCCAGAG ACTTAGCAGA

13401 AAAGAAATAT ATCCTCTCAA ATGCAAATTC ATTTTGCTAT GAAAATGAAG

13451 TTGCCCTCAC AAGCAAAGAG GAAGACGACA GTGAAAATGG AGTTCCAGAA

13501 AGCACTAGTA CGGACACGCC CCCTGACATA GACCTTCACA ACCAGGCAAG

13551 TGTACCTCTA GAGCCCAGGC CCTTACGGCG AGAATCGGAG ATATGATCTA

13601 GAGGATCTTT GTGAAGGAAC CTTACTTCTG TGGTGTGACA TAATTGGACA

13651 AACTACCTAC AGAGATTTAA AGCTCTAAGG TAAATATAAA ATTTTTAAGT

13701 GTATAATGTG TTAAACTACT GATTCTAATT GTTTGTGTAT TTTAGATTCC

13751 AACCTATGGA ACTGATGAAT GGGAGCAGTG GTGGAATGCC TTTAATGAGG

13801 AAAACCTGTT TTGCTCAGAA GAAATGCCAT CTAGTGATGA TGAGGCTACT

13851 GCTGACTCTC AACATTCTAC TCCTCCAAAA AAGAAGAGAA AGGTAGAAGA

13901 CCCCAAGGAC TTTCCTTCAG AATTGCTAAG TTTTTTGAGT CATGCTGTGT

13951 TTAGTAATAG AACTCTTGCT TGCTTTGCTA TTTACACCAC AAAGGAAAAA

14001 GCTGCACTGC TATACAAGAA AATTATGGAA AAATATTTGA TGTATAGTGC

14051 CTTGACTAGA GATCATAATC AGCCATACCA CATTTGTAGA GGTTTTACTT

14101 GCTTTAAAAA ACCTCCCACA CCTCCCCCTG AACCTGAAAC ATAAAATGAA

14151 TGCAATTGTT GTTGTTAACT TGTTTATTGC AGCTTATAAT GGTTACAAAT

14201 AAAGCAATAG CATCACAAAT TTCACAAATA AAGCATTTTT TTCACTGCAT

14251 TCTAGTTGTG GTTTGTCCAA ACTCATCAAT GTATCTTATC ATGTCTGGAT

14301 CACCGGTTAT GTTCGGCTTG TCGACATGCC CGCCGTGACC GTCGAGAACC

14351 CGCTGACGCT GCCCCGCGTA TCCGCACCCG CCGACGCCGT CGCACGTCCC

14401 GTGCTCACCG TGACCACCGC GCCCAGCGGT TTCGAGGGCG AGGGCTTCCC

14451 GGTGCGCCGC GCGTTCGCCG GGATCAACTA CCGCCACCTC GACCCGTTCA

14501 TCATGATGGA CCAGATGGGT GAGGTGGAGT ACGCGCCCGG GGAGCCCAAG

14551 GGCACGCCCT GGCACCCGCA CCGCGGCTTC GAGACCGTGA CCTACATCGT

14601 CGACGGTAAC ATGTGAGCAA AAGGCCAGCA AAAGGCCAGG AACCGTAAAA

14651 AGGCCGCGTT GCTGGCGTTT TTCCATAGGC TCCGCCCCCC TGACGAGCAT

14701 CACAAAAATC GACGCTCAAG TCAGAGGTGG CGAAACCCGA CAGGACTATA

14751 AAGATACCAG GCGTTTCCCC CTGGAAGCTC CCTCGTGCGC TCTCCTGTTC

14801 CGACCCTGCC GCTTACCGGA TACCTGTCCG CCTTTCTCCC TTCGGGAAGC

14851 GTGGCGCTTT CTCATAGCTC ACGCTGTAGG TATCTCAGTT CGGTGTAGGT

14901 CGTTCGCTCC AAGCTGGGCT GTGTGCACGA ACCCCCCGTT CAGCCCGACC

14951 GCTGCGCCTT ATCCGGTAAC TATCGTCTTG AGTCCAACCC GGTAAGACAC

15001 GACTTATCGC CACTGGCAGC AGCCACTGGT AACAGGATTA GCAGAGCGAG

15051 GTATGTAGGC GGTGCTACAG AGTTCTTGAA GTGGTGGCCT AACTACGGCT

15101 ACACTAGAAG AACAGTATTT GGTATCTGCG CTCTGCTGAA GCCAGTTACC

15151 TTCGGAAAAA GAGTTGGTAG CTCTTGATCC GGCAAACAAA CCACCGCTGG

15201 TAGCGGTGGT TTTTTTGTTT GCAAGCAGCA GATTACGCGC AGAAAAAAAG

15251 GATCTCAAGA AGATCCTTTG ATCTTTTCTA CGGGGTCTGA CGCTCAGTGG

15301 AACGAAAACT CACGTTAAGG GATTTTGGTC ATGAGATTAT CAAAAAGGAT

15351 CTTCACCTAG ATCCTTTTAA ATTAAAAATG AAGTTTTAAA TCAATCTAAA

15401 GTATATATGA GTAAACTTGG TCTGACAGTT ACCAATGCTT AATCAGTGAG

15451 GCACCTATCT CAGCGATCTG TCTATTTCGT TCATCCATAG TTGCCTGACT

15501 CCCCGTCGTG TAGATAACTA CGATACGGGA GGGCTTACCA TCTGGCCCCA

15551 GTGCTGCAAT GATACCGCGA GACCCACGCT CACCGGCTCC AGATTTATCA

15601 GCAATAAACC AGCCAGCCGG AAGGGCCGAG CGCAGAAGTG GTCCTGCAAC

15651 TTTATCCGCC TCCATCCAGT CTATTAATTG TTGCCGGGAA GCTAGAGTAA

15701 GTAGTTCGCC AGTTAATAGT TTGCGCAACG TTGTTGCCAT TGCTACAGGC

15751 ATCGTGGTGT CACGCTCGTC GTTTGGTATG GCTTCATTCA GCTCCGGTTC

15801 CCAACGATCA AGGCGAGTTA CATGATCCCC CATGTTGTGC AAAAAAGCGG

15851 TTAGCTCCTT CGGTCCTCCG ATCGTTGTCA GAAGTAAGTT GGCCGCAGTG

15901 TTATCACTCA TGGTTATGGC AGCACTGCAT AATTCTCTTA CTGTCATGCC

15951 ATCCGTAAGA TGCTTTTCTG TGACTGGTGA GTACTCAACC AAGTCATTCT

16001 GAGAATAGTG TATGCGGCGA CCGAGTTGCT CTTGCCCGGC GTCAATACGG

16051 GATAATACCG CGCCACATAG CAGAACTTTA AAAGTGCTCA TCATTGGAAA

16101 ACGTTCTTCG GGGCGAAAAC TCTCAAGGAT CTTACCGCTG TTGAGATCCA

16151 GTTCGATGTA ACCCACTCGT GCACCCAACT GATCTTCAGC ATCTTTTACT

16201 TTCACCAGCG TTTCTGGGTG AGCAAAAACA GGAAGGCAAA ATGCCGCAAA

16251 AAAGGGAATA AGGGCGACAC GGAAATGTTG AATACTCATA CTCTTCCTTT

16301 TTCAATATTA TTGAAGCATT TATCAGGGTT ATTGTCTCAT GAGCGGATAC

16351 ATATTTGAAT GTATTTAGAA AAATAAACAA ATAGGGGTTC CGCGCACATT

16401 TCCCCGAAAA GTGCCACCTG AGTC

**HJP-381-actin5C-FloxSyn21Gal80-10XUAS-mCD8GFP**

1 TATCACGAGG CCCTTTCGTC TTCAAGAATT CGTTTATCAC AAGTTTGTAC

51 AAAAAAGCAG GCTCCGCGGC CGCAATTCTA TATTCTAAAA ACACAAATGA

101 TACTTCTAAA AAAAAATCAT GAATGGCATC AACTCTGAAT CAAATCTTTG

151 CAGATGCACC TACTTCTCAT TTCCACTGTC ACATCATTTT TCCAGATCTC

201 GCTGCCTGTT ATGTGGCCCA CAAACCAAGA CACGTTTTAT GGCCATTAAA

251 GCTGGCTGAT CGTCGCCAAA CACCAAATAC ATAATGAATA TGTACACATT

301 CGAGAAAGAA GCGATCAAAG AAGCGTCTTC GGGCGGAGTA GGAGAATGCG

351 GAGGAGAAGG AGAACGAGCT GATCTAGTAT CTCTCCACAA TCCAATGCCA

401 ACTGACCAAC TGGCCATATT CGGAGCAATT TGAAGCCAAT TTCCATCGCC

451 TGGCGATCGC TCCATTCTTG GCTATATGTT TTTCACCGTT ACCCGGGGCC

501 ATTTTCAAAG ACTCGTCGGC AAGATAAGAT TGTGTCACTC GCTGTCTCTC

551 TTCATTTGTC GAAGAATGCT GAGGAATTTC GCGATGACGT CGGCGAGTAT

601 TTTGAAGAAT GAGAATAATT TGTATTTATA CGAAAATCAG TTAGTGGAAT

651 TTTCTACAAA AACATGTTAT CTATAGATAA TTTTGTTGCA AAATATGTTG

701 ACTATGACAA AGATTGTATG TATATACCTT TAATGTATTC TCATTTTCTT

751 ATGTATTTAT AATGGCAATG ATGATACTGA TGATATTTTA AGATGATGCC

801 AGACCAAAAG GCTTGAATTT CTGCGTCTTT TGCCGAACGC AGTGCATGTG

851 CAATTGTTGT TTTTTGGAAT ATTCAATTTT CGGACTGTCC GCTTTGATTT

901 CAGTTTCTTG GCTTATTCAA AAAGCAAAGT AAAGCCAAAA AAGCGAGATG

951 GCAATACCAA ATGCGGCAAA ACGGTAGTGG AAGGAAAGGG GTGCGGGGCA

1001 GCGGAAGGAA GGGTGGGGCG GGGCGTGGCG GGGTCTGTGG CTGGGCGCGA

1051 CGTCACCGAC GTTGGAGCCA CTCCTTTGAC CATGTGTGCG TGTGTGTATT

1101 ATTCGTGTCT CGCCACTCGC CGGTTGTTTT TTTCTTTTTA TGCTGCGCTC

1151 TCTCTAGCGC CATCTCGCTT ACGCATGCTC AACGCACCGC ATGTTGCCGT

1201 TTCCTTTTAT GCGTCATTTT GGCTCGAAAT AGGCAATTAT TTAAACAAAG

1251 ATTAGTCAAC GAAAACGCTA AAATAAATAA GTCTACAATA TGGTTACTTA

1301 TTGCCATGTG TGTGCAGCCA ACGATAGCAA CAAAAGCAAC AACACAGGTG

1351 GCTTTCCCTC TTTCACTTTT TGTTTGCAAG CCGCGTGCGA GCAAGACGGC

1401 ACGACCGGCA AACGCAATTA CGCTGACAAA GAGCAGACGA AGTTTTGGCG

1451 AAAAACATCA AGGCGCCTGA TACGAATGCA TTTGCAATAA CAATTGCGAT

1501 ATTTAATATT GTTTATGAAG CTGTTTGACT TCAAAACACA CAAAAAAAAA

1551 AATAAAACAA ATTATTTGAA AGAGAATTAG GAATCGGACG CTTATCGTTA

1601 GGGTAACAAC AAGAAATGCT TACTGAGTCA CAGCCTCTGG AAAACTGCCG

1651 CAAGCCAGAG AGAGAGAGAA AAAGAGGGAG AGCAGCTTAG ACCGCATGTG

1701 CTTGTGTGTG AGGCGTCTCT CTCTTCGTCT CTGTTGCGCA AACGCATAGA

1751 CTGCACTGAA AAAATCGATT ACCTATTTTT TATGAATGAA TATTTGCACT

1801 ATTACTATTC AAAACTATTA AGATAGCAAT CACATTCAAT AGCCAAATAC

1851 TATACCACCT GAGCGATGCA ACGAAATGAT CAATTTGAGC AAAAATGCTG

1901 CATATTTAGG ACGGCATCAT TATAGAAATG CTTCTTGCTG TGTACTTTTC

1951 TCTCGTCTGG CAGCTGTTTC GCCGTTATTG TTAAAACCGG CTTAAGTTAG

2001 GTGTGTTTTC TACGACTAGT GAATGCCCTA CTAGAAGATG TGTGTTGCAC

2051 AAAATGTCCC TGGAATAACC AATTTGAAGT GCAGATAGCA GTAAACGTAA

2101 GCTAATATGA ATATTATTTA ACTGTAATGT TTTAATATCG CTGGACATTA

2151 CTAATAAACC CACTATAAAC ACATGTACAT ATGTATGTTT TGGCATACAA

2201 TGAGTAGTTG GGGAAAAAAT GTGTAAAAGC ACCGTGACCA TCACAGCATA

2251 AAGATAACCA GCTGAAGTAT CGAATATGAG TAACCCCCAA ATTGAATCAC

2301 ATGCCGCAAC TGATAGGACC CATGGAAGTA CACTCTTCAT GGCGATATAC

2351 AAGACACACA CAAGCACGAA CACCCAGTTG CGGAGGAAAT TCTCCGTAAA

2401 TGAAAACCCA ATCGGCGAAC AATTCATACC CATATATGGT AAAAGTTTTG

2451 AACGCGACTT GAGAGCGGAG AGCATTGCGG CTGATAAGGT TTTAGCGCTA

2501 AGCGGGCTTT ATAAAACGGG CTGCGGGACC AGTTTTCATA TCACTACCGT

2551 TTGAGTTCTT GTGCTGTGTG GATACTCCTC CCGACACAAA GCCGCTCCAT

2601 CAGCCAGCAG TCGTCTAATC CAGAGACACC AAACCGAAAG ACTTAATTTA

2651 TATTTATTTA ATTAATTTTA ATAAAACACA CCAAATGTAA GTAGCTTTCC

2701 CCTTCCCAAC AACAAAACAC CATCGAACCA CTCCCACCAA GAAAAAGCAA

2751 TAATCGAGAA AAGCCGCGGA AAATGTGTGA TTTTTTTTGT AAACAAAACA

2801 TTTTTTTATG TGCCAGTGCT GAAAGTGATC AAAAAATACT AGCCACGAGC

2851 TAAAGAGTTA TTGTATTGAC CAAAACTCCA AAAATACCCA AGTTTGGCCC

2901 TAAATTGTCA ATCAAAATAC CAATAGGTCG AAAGACATCA AAATTAACAA

2951 AACCAGGGTT TCAAATACCA TAACTCAAGA ATCAGGATTA CAACTGCAGA

3001 TTTCAGGATA TATACATACA AATTATACGA AATTATAAAA ACCAAAGCAA

3051 TTCAATAGCC CCAACTCAAA TGTTAGGATC TAATATAGTG TTTAAAGCCA

3101 AGCTCGCTGA TGTGGGCGTG TCACGATTTC ACCCAAAGAT ATGCCAAATT

3151 ACGAATTGCA AATCAATTCG CCAACACTTT CTTTTTTTCC CACGCCCTAA

3201 AACACCAGAT CATCCATAAA TGTACATACA TACAGTATAT GCATATTATA

3251 ATCTGTAAAA CTAGATCAGG TTCTTGAAAA TAGTGACGTA GGCAGCCGTT

3301 TTGGCTGAAG CAGAAATTTT TGCCGGTTTT TCAAAGTTGT AGTTGCAAAA

3351 ATGGAGAAAA CCTTCGAGCA TTCGTTCATA TACACACACT CACGCGCAAA

3401 ATAACGAGAG AGAGTGTATG TGTGTGTGAG AGAGCGAAAG CCAGACGACG

3451 GTTTGCTTTT CGCCTCGAAA CATGACCATA TATGGTCACA AAACTTGGCC

3501 GCCGCAATTC AACACACCAG CGCTCTCCTT CGCACCCATA GCGACCATGG

3551 CGCGGAGCGA GCGAGATGGC GAGAGCGAGC GACGCCTATG GCGACGTCGA

3601 CGCAGGCAGC GATTGAAAAA CGCAGTTAAC TGGCATTCAA CATTCACCAG

3651 CCACTTTCAG TCGGTTTATT CCAGTCATTC CTTTCAAACC GTGCGGTCGC

3701 TTAGCTCAGC CTCGCCACTT GCGTTTACAG TAGTTTTCAC GCCTTGAATT

3751 TGTTAAATCG AACAAAAAGG TAAAGTTTAA CTAGCTTTGA AAAGTTTCGT

3801 GGCTCTTAAT TGTTAAATTT TCTAGAGTGC GTTTAGTGTT TTTTTTTTTT

3851 TTTATTTTGT AATGTTAATT TCGGGTTCCA ATTCGAGTTT TAGGCAGCCG

3901 CCATTTTAAG GGCGCATACA CACAGGCAAC TGTGCTCTCT TTGCGGCTTT

3951 CTTTTGCACC GGCATTCGTT AAGTGTCGTC TAGAAGCTTC TCCCCTCCCT

4001 TTTCGGCATA TTCGTATTGT GGTTTTAATT TTTCGGGGCG GGGCCTTCTA

4051 TTTTGTAACT GTTCTTTTAA TTTCTTATTA CAATTCGATC GCAAGTGAAA

4101 ATCAGTTTTC AATCGGAAAA GTATTTTTTT ATGAAATTTT TTTTTGTCCA

4151 AGATTAAAAT TTTGTACTAA AAAAACGTAC ATTGCATTGA GTGATTTTTA

4201 ATTGTACACG AAAAACAAGT TAGTTTGTTA TGACAATTGT ACTTTGGTAG

4251 ACCAGCGCAG TCCAAGGAAA CCACGCAAAT TCTCAGTTTT TTTTTTGCCA

4301 TTTCTACATT ACCAAATAAG GTAACCAAAA ACTAATGGGA AATCCGCATT

4351 CTTTCCATTG CAGCTTACAG GATCGATCCC CGGGTACCGC GCCGACCCAG

4401 CTTTCTTGTA CAAAGTGGTG ATAAACGGCC GGCATAACTT CGTATAATGT

4451 ATGCTATACG AAGTTATCCT GCAGGAACTT AAAAAAAAAA ATCAAAATGG

4501 ATTACAACAA AAGGAGTAGT GTGAGTACGG TGCCGAATGC TGCTCCCATT

4551 CGCGTGGGCT TCGTGGGATT GAACGCGGCT AAGGGTTGGG CCATTAAAAC

4601 GCATTATCCA GCCATACTGC AGCTGAGCTC CCAGTTCCAA ATAACAGCGC

4651 TTTATTCCCC CAAGATCGAG ACGTCCATTG CGACAATTCA GAGGCTGAAG

4701 TTGTCCAACG CCACAGCTTT CCCAACACTC GAGAGCTTCG CCTCGAGCTC

4751 CACGATCGAT ATGATTGTGA TCGCAATCCA GGTGGCTTCC CACTACGAGG

4801 TGGTAATGCC CCTGCTCGAG TTTTCGAAGA ACAATCCTAA TCTGAAGTAT

4851 TTGTTTGTGG AATGGGCACT GGCCTGCTCG CTCGATCAGG CGGAGTCGAT

4901 CTATAAGGCC GCTGCAGAGC GGGGCGTGCA AACAATAATC AGTCTGCAGG

4951 GACGAAAGAG CCCGTACATT TTGCGGGCAA AAGAGCTTAT CTCCCAGGGC

5001 TACATCGGCG ATATAAATAG CATCGAGATT GCAGGTAACG GAGGTTGGTA

5051 CGGTTACGAG CGGCCGGTGA AAAGCCCGAA ATACATTTAC GAGATCGGAA

5101 ACGGAGTTGA TCTGGTGACC ACCACGTTCG GTCACACGAT AGATATATTG

5151 CAGTACATGA CCAGTTCGTA CTTCAGCCGT ATCAATGCCA TGGTGTTTAA

5201 CAACATTCCA GAACAGGAGC TCATTGATGA GAGGGGCAAT CGCCTGGGCC

5251 AGCGGGTCCC AAAGACTGTC CCAGATCATC TCTTGTTCCA GGGCACGCTG

5301 CTCAACGGAA ATGTTCCTGT GTCCTGTTCC TTTAAGGGAG GCAAGCCTAC

5351 GAAAAAGTTC ACCAAGAATC TGGTCATAGA TATCCATGGT ACAAAGGGTG

5401 ATCTGAAGCT GGAGGGTGAC GCTGGATTTG CGGAAATCTC GAATCTCGTT

5451 CTGTATTATT CGGGCACCCG CGCCAATGAT TTTCCCCTGG CCAATGGTCA

5501 GCAGGCACCC TTGGACCCCG GCTACGACGC AGGCAAGGAG ATTATGGAGG

5551 TGTATCACCT CCGAAACTAC AACGCGATCG TTGGTAATAT ACACCGGCTC

5601 TACCAGAGTA TCAGCGATTT CCACTTCAAC ACCAAGAAAA TCCCCGAGCT

5651 TCCATCGCAG TTCGTAATGC AAGGCTTCGA TTTCGAAGGC TTTCCCACCC

5701 TGATGGATGC CTTGATCTTG CATCGCCTGA TTGAAAGTGT CTACAAGTCG

5751 AATATGATGG GCAGTACCCT GAATGTTTCC AACATCTCCC ACTACTCGCT

5801 TTAAACGCGT AGGATCTTTG TGAAGGAACC TTACTTCTGT GGTGTGACAT

5851 AATTGGACAA ACTACCTACA GAGATTTAAA GCTCTAAGGT AAATATAAAA

5901 TTTTTAAGTG TATAATGTGT TAAACTACTG ATTCTAATTG TTTGTGTATT

5951 TTAGATTCCA ACCTATGGAA CTGATGAATG GGAGCAGTGG TGGAATGCCT

6001 TTAATGAGGA AAACCTGTTT TGCTCAGAAG AAATGCCATC TAGTGATGAT

6051 GAGGCTACTG CTGACTCTCA ACATTCTACT CCTCCAAAAA AGAAGAGAAA

6101 GGTAGAAGAC CCCAAGGACT TTCCTTCAGA ATTGCTAAGT TTTTTGAGTC

6151 ATGCTGTGTT TAGTAATAGA ACTCTTGCTT GCTTTGCTAT TTACACCACA

6201 AAGGAAAAAG CTGCACTGCT ATACAAGAAA ATTATGGAAA AATATTTGAT

6251 GTATAGTGCC TTGACTAGAG ATCATAATCA GCCATACCAC ATTTGTAGAG

6301 GTTTTACTTG CTTTAAAAAA CCTCCCACAC CTCCCCCTGA ACCTGAAACA

6351 TAAAATGAAT GCAATTGTTG TTGTTAACTT GTTTATTGCA GCTTATAATG

6401 GTTACAAATA AAGCAATAGC ATCACAAATT TCACAAATAA AGCATTTTTT

6451 TCACTGCATT CTAGTTGTGG TTTGTCCAAA CTCATCAATG TATCTTATCA

6501 TGTCTGGATC CCTAGGATAA CTTCGTATAA TGTATGCTAT ACGAAGTTAT

6551 GGTACCGCTA GTTGGCCACG GGTGCGCATG ATCGTGCTCC TGTCGTTGAG

6601 GACCCGGCTA GGCTGGCGGG GTTGCCTTAC TGGTTAGCAG AATGAATCAC

6651 CGATACGCGA GCGAACGTGA AGCGACTGCT GCTGCAAAAC GTCTGCGACC

6701 TGAGCAACAA CATGAATGGT CTTCGGTTTC CGTGTTTCGT AAAGTCTGGA

6751 AACGCGGAAG TCAGCGCCCT GCACCATTAT GTTCCGGACT AGTTCCAGTG

6801 AAATCCAAGC ATTTTCTAAA TTAAATGTAT TCTTATTATT ATAGTTGTTA

6851 TTTTTGATAT ATATAAACAA CACTATTATG CCCACCATTT TTTTGAGATG

6901 CATCTACACA AGGAACAAAC ACTGGATGTC ACTTTCAGTT CAAATTGTAA

6951 CGCTAATCAC TCCGAACAGG TCACAAAAAA TTACCTTAAA AAGTCATAAT

7001 ATTAAATTAG AATAAATATA GCTGTGAGGG AAATATATAC AAATATATTG

7051 GAGCAAATAA ATTGTACATA CAAATATTTA TTACTAATTT CTATTGAGAC

7101 GAAATGAACC ACTCGGAACC ATTTGAGCGA ACCGAATCGC GCGGAACTAA

7151 CGACAGTCGC TCCAAGGTCG TCGAACAAAA GGTGAATGTG TTGCGGAGAG

7201 CGGGTGGGAG ACAGCGAAAG AGCAACTACG AAACGTGGTG TGGTGGAGGT

7251 GAATTATGAA GAGGGCGCGC GATTTGAAAA GTATGTATAT AAAAAATATA

7301 TCCCGGTGTT TTATGTAGCG ATAAACGAGT TTTTGATGTA AGGTATGCAG

7351 GTGTGTAAGT CTTTTGGTTA GAAGACAAAT CCAAAGTCTA CTTGTGGGGA

7401 TGTTCGAAGG GGAAATACTT GTATTCTATA GGTCATATCT TGTTTTTATT

7451 GGCACAAATA TAATTACATT AGCTTTTTGA GGGGGCAATA AACAGTAAAC

7501 ACGATGGTAA TAATGGTAAA AAAAAAAAAC AAGCAGTTAT TTCGGATATA

7551 TGTCGGCTAC TCCTTGCGTC GGGCCCGAAG TCTTAGAGCC AGATATGCGA

7601 GCACCCGGAA GCTCACGATG AGAATGGCCA GACCCACGTA GTCCAGCGGC

7651 AGATCGGCGG CGGAGAAGTT AAGCGTCTCC AGGATGACCT TGCCCGAACT

7701 GGGGCACGTG GTGTTCGACG ATGTGCAGCT AATTTCGCCC GGCTCCACGT

7751 CCGCCCATTG GTTAATCAGC AGACCCTCGT TGGCGTAACG GAACCATGAG

7801 AGGTACGACA ACCATTTGAG GTATACTGGC ACCGAGCCCG AGTTCAAGAA

7851 GAAGCCGCCA AAGAGCAGGA ATGGTATGAT AACCGGCGGA CCCACAGACA

7901 GCGCCATCGA GGTCGAGGAG CTGGCGCAGG ATATTAGATA TCCGAAGGAC

7951 GTTGACACAT TGGCCACCAG AGTGACCAGC GCCAGGCAGT TGAAGAAGTG

8001 CAGCACTCCG GCCCGCAGTC CGATCATCGG ATAGGCAATC GCCGTGAAGA

8051 CCAGTGGCAC TGTGAGAAAA AGCGGCAATT CGGCAATCGT TTTGCCCAGA

8101 AAGTATGTGT CACAGCGATA AAGTCGACTT CGGGCCTCCC TCATAAAAAC

8151 TGGCAGCTCT GAGGTGAACA CCTAAATCGA ATCGATTCAT TAGAAAGTTA

8201 GTAAATTATT GAAATGCAAA TGTATTCTAA ACATGACTTA CATTTATCGT

8251 GGCAAAGACG TTTTGAAAGG TCATGTTGGT CAGGAAGAGG AAGATGGCTC

8301 CGTTGATATT CATCACACCC ACTTGCGTGA GTTGTTGGCC CAAAAAGATG

8351 AGGCCAATCA AGATGGCAAC CATCTGCAAA TTAAAATGTT ACTCGCATCT

8401 CATTAATATT CGCGAGTTAA ATGAAATTTA TTTATCTTCT GCAAAACTAT

8451 AAACTATACA TCTCATTGAA AAAAACTAAG AAGGGTGTGG AATCAGGCAA

8501 TTCTATCTAA AATCTAGCGA ATTTGTTTCC AAGAATTGTA AGCGTTATAT

8551 CATTTGTTTC CACTGGAACC ACTCACCGTT GTCTGAATAA GTCGCACTTT

8601 TACGAGGAGT GGTTCCTTGA GCACCGACAG CCAGGATCGC CACAGGACCG

8651 CCCGGAACTG CATGAACCAG GTGGCCTTGT AGGTGTACCC ATTCTCCGGC

8701 TGCTCCAGTG GCTTCTCCAG ATTTTTGGTG GCCAACAACT GCTCCATATC

8751 CCGGGCTACT TTGCTAATGG CAAAATTGTC GCATATCTTG GCGATCCGAT

8801 CACGGGACTC GATCTCCCGT CCGGGCACAA CGGCCAACAC CTGTACGTAA

8851 AAGTCCGCCG GATTGTAGTT GGTAGGACAC TGGGCACCCA CGCTGGATAG

8901 GAGTTGAGAT GTTATGTAAT ACTAGATACC CTTAATAAAC ACATCGAACT

8951 CACTAGGAAA AGAAGTCGAC GGCTTCGCTG GGAGTGCCCA AGAAAGCTAC

9001 CCTGCCCTCG GCCATCAGAA GGATCTTGTC AAAGAGCTCA AACAGCTCGG

9051 AAGACGGCTG ATGAATGGTC AGGATGACGG TCTTGCCCTT CTGCGACAGC

9101 TTCTTCAGCA CCTGGACGAC GCTGTGGGCG GTAAAGGAGT CCAGTCCGGA

9151 GGTGGGCTCA TCGCAGATCA GAAGCGGCGG ATCGGTTAGA GCCTCGGAGG

9201 CGAATGCCAG ACGCTTCCTT TCTCCGCCGG ACAGACCTTT CACCCTGCCG

9251 GGCACACCGA TGATCGTGTG CTGACATTTG CTGAGCGAAA GCTCCTGGAT

9301 CACCTGATCC ACGCGGGCCA CTCGCTGCCG ATAGGTCAGA TGTCGTGGCA

9351 TCCGCACCAT GGCTTGGAAA ATCAGGTGTT CCCTGGCCGT TAGGGAGCCG

9401 ATAAAGAGGT CATCCTGCTG GACATAGGCG CACCTGGCCT GCATCTCCTT

9451 GGCGTCCACA GGTTGGCCAT TGAGCAGTCG CATCCCGGAT GGCGATACTT

9501 GGATGCCCTG CGGCGATCGA AAGGCAAGGG CATTCAGCAG GGTCGTCTTT

9551 CCGGCACCGG AACTGCCCAT CACGGCCAAA AGTTCGCCCG GATAGGCCAC

9601 GCCGCAAACT GAGTTTCAAA TTGGTAATTG GACCCTTTAT TAAGATTTCA

9651 CACAGATCAG CCGACTGCGA ATAGAAACTC ACCGTTCTTG AGCAAATGTT

9701 TCCTGGGCGC CGGTATGTGT CGCTCGTTGC AGAATAGTCC GCGTGTCCGG

9751 TTGACCAGCT GCCGCCATCC GGAGCCCGGC TGATTGACCG CCCCAAAGAT

9801 GTCCATATTG TGCCAGGCAT AGGTGAGGTT CTCGGCTAGT TGGCCGCTCC

9851 CTGAACCGGA GTCCTCCGGC GGACTGGGTG GCCGGAGCGT GCCGTAGTTT

9901 TTGGCCTGCC CGAAGCCCTG GTTAATGCAG CTCTGCGAAG CCGCTCCGCT

9951 GTCACCCTGC AATGATAGGG GATCTCAAAT ATCAACTACT AGCGTTATGC

10001 TCATCTAACC CCGAACAAAA AGTACCCCGA AGTATCCTAC GAAGTAGGTT

10051 TATACTTTTA TTTATTTTTT GTGCATCTAG GATCAGCTTA AAATATCTGG

10101 TTGTTATATT TTTTGTAAAA AAGAATATAG TCGAAAATGA ATGCCTTTAG

10151 ATGTCTTGAT CATGATATGA TCTCAAAAAT TGTCTTATAT AGCGAGAACA

10201 GCTACCAGAA TAATCTGTTT CGTGTCACTA TTTGTTTGTG CAATTGCGGT

10251 TTGGGATTTT TGTGGGTCGC AGTTCTCACG CCGCATACAA TTTGATGTTG

10301 CAATCGCAGT TCCTATAGAT CAAGTGAACT TAAGATGTAT GCACATGTAC

10351 TACTCACATT GTTCAGATGC TCGGCAGATG GGTGTTTGCT GCCTCCGCGA

10401 ATTAATAGCT CCTGATCCTC TTGGCCCATT GCCGGGATTT TTCACACTTT

10451 CCCCTGCTTA CCCACCCAAA ACCAATCACC ACCCCAATCA CTCAAAAAAC

10501 AAACAAAAAT AAGAAGCGAG AGGAGTTTTG GCACAGCACT TTGTGTTTAA

10551 TTGATGGCGT AAACCGCTTG GAGCTTCGTC ACGAAACCGC TGACAAAATG

10601 CAACTGAAGG CGGACATTGA CGCTACGTAA CGCTACAAAC GGTGGCGAAA

10651 GAGATAGCGG ACGCAGCGGC GAAAGAGACG GCGATATTTC TGTGGACAGA

10701 GAAGGAGGCA AACAGCGCTG ACTTTGAGTG GAATGTCATT TTGAGTGAGA

10751 GGTAATCGAA AGAACCTGGT ACATCAAATA CCCTTGGATC GAAGTAAATT

10801 TAAAACTGAT CAGATAAGTT CAATGATATC CAGTGCAGTA AAAAAAAAAA

10851 ATGTTTTTTT TATCTACTTT CCGCAAAAAT GGGTTTTATT AACTTACATA

10901 CATACTAGGC GCGCCCATAA GCTTGCATGC CTGCAGGTCG GAGTACTGTC

10951 CTCCGAGCGG AGTACTGTCC TCCGAGCGGA GTACTGTCCT CCGAGCGGAG

11001 TACTGTCCTC CGAGCGGAGT ACTGTCCTCC GAGCGGAGAC TCTAGCCCTA

11051 GGGCATGCCT GCAGGTCGGA GTACTGTCCT CCGAGCGGAG TACTGTCCTC

11101 CGAGCGGAGT ACTGTCCTCC GAGCGGAGTA CTGTCCTCCG AGCGGAGTAC

11151 TGTCCTCCGA GCGGAGACTC TAGCGCTAGC GACGTCGAGC GCCGGAGTAT

11201 AAATAGAGGC GCTTCGTCTA CGGAGCGACA ATTCAATTCA AACAAGCAAA

11251 GTGAACACGT CGCTAAGCGA AAGCTAAGCA AATAAACAAG CGCAGCTGAA

11301 CAAGCTAAAC AATCTGCAGT AAAGTGCAAG TTAAAGTGAA TCAATTAAAA

11351 GTAACCAGCA ACCAAGTAAA TCAACTGCAA CTACTGAAAT CTGCCAAGAA

11401 GTAATTATTG AATACAAGAA GAGAACTCTG AATAGATCTG CGGCCGCGGC

11451 TCGAGCAAAA TGGCCTCACC GTTGACCCGC TTTCTGTCGC TGAACCTGCT

11501 GCTGCTGGGT GAGTCGATTA TCCTGGGGAG TGGAGAAGCT AAGCCACAGG

11551 CACCCGAACT CCGAATCTTT CCAAAGAAAA TGGACGCCGA ACTTGGTCAG

11601 AAGGTGGACC TGGTATGTGA AGTGTTGGGG TCCGTTTCGC AAGGATGCTC

11651 TTGGCTCTTC CAGAACTCCA GCTCCAAACT CCCCCAGCCC ACCTTCGTTG

11701 TCTATATGGC TTCATCCCAC AACAAGATAA CGTGGGACGA GAAGCTGAAT

11751 TCGTCGAAAC TGTTTTCTGC CATGAGGGAC ACGAATAATA AGTACGTTCT

11801 CACCCTGAAC AAGTTCAGCA AGGAAAACGA AGGCTACTAT TTCTGCTCAG

11851 TCATCAGCAA CTCGGTGATG TACTTCAGTT CTGTCGTGCC AGTCCTTCAG

11901 AAAGTGAACT CTACTACTAC CAAGCCAGTG CTGCGAACTC CCTCACCTGT

11951 GCACCCTACC GGGACATCTC AGCCCCAGAG ACCAGAAGAT TGTCGGCCCC

12001 GTGGCTCAGT GAAGGGGACC GGATTGGACT TCGCCTGTGA TATTTACATC

12051 TGGGCACCCT TGGCCGGAAT CTGCGTGGCC CTTCTGCTGT CCTTGATCAT

12101 CACTCTCATC TGCTACCACA GCCGCGGATC CATGAGTAAA GGAGAAGAAC

12151 TTTTCACTGG AGTTGTCCCA ATTCTTGTTG AATTAGATGG TGATGTTAAT

12201 GGGCACAAAT TTTCTGTCAG TGGAGAGGGT GAAGGTGATG CAACATACGG

12251 AAAACTTACC CTTAAATTTA TTTGCACTAC TGGAAAACTA CCTGTTCCAT

12301 GGCCAACACT TGTCACTACT TTAACTTATG GTGTTCAATG CTTTTCAAGA

12351 TACCCAGATC ATATGAAACA GCATGACTTT TTCAAGAGTG CCATGCCCGA

12401 AGGTTATGTC CAGGAAAGAA CTATATTTTT CAAAGATGAC GGGAACTACA

12451 AGACACGTGC TGAAGTCAAG TTTGAAGGTG ATACCCTTGT TAATAGAATC

12501 GAGTTAAAAG GTATTGATTT TAAAGAAGAT GGAAACATTC TTGGACACAA

12551 ATTGGAATAC AACTATAACT CACACAATGT ATACATCATG GCAGACAAAC

12601 AAAAGAATGG AATCAAAGTT AACTTCAAAA TTAGACACAA CATTGAAGAT

12651 GGAAGCGTTC AACTAGCAGA CCATTATCAA CAAAATACTC CAATTGGCGA

12701 TGGCCCTGTC CTTTTACCAG ACAACCATTA CCTGTCCACA CAATCTGCCC

12751 TTTCGAAAGA TCCCAACGAA AAGAGAGACC ACATGGTCCT TCTTGAGTTT

12801 GTAACAGCTG CTGGGATTAC ACATGGCATG GATGAACTAT ACAAATAATC

12851 TAGAGGATCT TTGTGAAGGA ACCTTACTTC TGTGGTGTGA CATAATTGGA

12901 CAAACTACCT ACAGAGATTT AAAGCTCTAA GGTAAATATA AAATTTTTAA

12951 GTGTATAATG TGTTAAACTA CTGATTCTAA TTGTTTGTGT ATTTTAGATT

13001 CCAACCTATG GAACTGATGA ATGGGAGCAG TGGTGGAATG CCTTTAATGA

13051 GGAAAACCTG TTTTGCTCAG AAGAAATGCC ATCTAGTGAT GATGAGGCTA

13101 CTGCTGACTC TCAACATTCT ACTCCTCCAA AAAAGAAGAG AAAGGTAGAA

13151 GACCCCAAGG ACTTTCCTTC AGAATTGCTA AGTTTTTTGA GTCATGCTGT

13201 GTTTAGTAAT AGAACTCTTG CTTGCTTTGC TATTTACACC ACAAAGGAAA

13251 AAGCTGCACT GCTATACAAG AAAATTATGG AAAAATATTT GATGTATAGT

13301 GCCTTGACTA GAGATCATAA TCAGCCATAC CACATTTGTA GAGGTTTTAC

13351 TTGCTTTAAA AAACCTCCCA CACCTCCCCC TGAACCTGAA ACATAAAATG

13401 AATGCAATTG TTGTTGTTAA CTTGTTTATT GCAGCTTATA ATGGTTACAA

13451 ATAAAGCAAT AGCATCACAA ATTTCACAAA TAAAGCATTT TTTTCACTGC

13501 ATTCTAGTTG TGGTTTGTCC AAACTCATCA ATGTATCTTA TCATGTCTGG

13551 ATCACCGGTT ATGTTCGGCT TGTCGACATG CCCGCCGTGA CCGTCGAGAA

13601 CCCGCTGACG CTGCCCCGCG TATCCGCACC CGCCGACGCC GTCGCACGTC

13651 CCGTGCTCAC CGTGACCACC GCGCCCAGCG GTTTCGAGGG CGAGGGCTTC

13701 CCGGTGCGCC GCGCGTTCGC CGGGATCAAC TACCGCCACC TCGACCCGTT

13751 CATCATGATG GACCAGATGG GTGAGGTGGA GTACGCGCCC GGGGAGCCCA

13801 AGGGCACGCC CTGGCACCCG CACCGCGGCT TCGAGACCGT GACCTACATC

13851 GTCGACGGTA ACATGTGAGC AAAAGGCCAG CAAAAGGCCA GGAACCGTAA

13901 AAAGGCCGCG TTGCTGGCGT TTTTCCATAG GCTCCGCCCC CCTGACGAGC

13951 ATCACAAAAA TCGACGCTCA AGTCAGAGGT GGCGAAACCC GACAGGACTA

14001 TAAAGATACC AGGCGTTTCC CCCTGGAAGC TCCCTCGTGC GCTCTCCTGT

14051 TCCGACCCTG CCGCTTACCG GATACCTGTC CGCCTTTCTC CCTTCGGGAA

14101 GCGTGGCGCT TTCTCATAGC TCACGCTGTA GGTATCTCAG TTCGGTGTAG

14151 GTCGTTCGCT CCAAGCTGGG CTGTGTGCAC GAACCCCCCG TTCAGCCCGA

14201 CCGCTGCGCC TTATCCGGTA ACTATCGTCT TGAGTCCAAC CCGGTAAGAC

14251 ACGACTTATC GCCACTGGCA GCAGCCACTG GTAACAGGAT TAGCAGAGCG

14301 AGGTATGTAG GCGGTGCTAC AGAGTTCTTG AAGTGGTGGC CTAACTACGG

14351 CTACACTAGA AGAACAGTAT TTGGTATCTG CGCTCTGCTG AAGCCAGTTA

14401 CCTTCGGAAA AAGAGTTGGT AGCTCTTGAT CCGGCAAACA AACCACCGCT

14451 GGTAGCGGTG GTTTTTTTGT TTGCAAGCAG CAGATTACGC GCAGAAAAAA

14501 AGGATCTCAA GAAGATCCTT TGATCTTTTC TACGGGGTCT GACGCTCAGT

14551 GGAACGAAAA CTCACGTTAA GGGATTTTGG TCATGAGATT ATCAAAAAGG

14601 ATCTTCACCT AGATCCTTTT AAATTAAAAA TGAAGTTTTA AATCAATCTA

14651 AAGTATATAT GAGTAAACTT GGTCTGACAG TTACCAATGC TTAATCAGTG

14701 AGGCACCTAT CTCAGCGATC TGTCTATTTC GTTCATCCAT AGTTGCCTGA

14751 CTCCCCGTCG TGTAGATAAC TACGATACGG GAGGGCTTAC CATCTGGCCC

14801 CAGTGCTGCA ATGATACCGC GAGACCCACG CTCACCGGCT CCAGATTTAT

14851 CAGCAATAAA CCAGCCAGCC GGAAGGGCCG AGCGCAGAAG TGGTCCTGCA

14901 ACTTTATCCG CCTCCATCCA GTCTATTAAT TGTTGCCGGG AAGCTAGAGT

14951 AAGTAGTTCG CCAGTTAATA GTTTGCGCAA CGTTGTTGCC ATTGCTACAG

15001 GCATCGTGGT GTCACGCTCG TCGTTTGGTA TGGCTTCATT CAGCTCCGGT

15051 TCCCAACGAT CAAGGCGAGT TACATGATCC CCCATGTTGT GCAAAAAAGC

15101 GGTTAGCTCC TTCGGTCCTC CGATCGTTGT CAGAAGTAAG TTGGCCGCAG

15151 TGTTATCACT CATGGTTATG GCAGCACTGC ATAATTCTCT TACTGTCATG

15201 CCATCCGTAA GATGCTTTTC TGTGACTGGT GAGTACTCAA CCAAGTCATT

15251 CTGAGAATAG TGTATGCGGC GACCGAGTTG CTCTTGCCCG GCGTCAATAC

15301 GGGATAATAC CGCGCCACAT AGCAGAACTT TAAAAGTGCT CATCATTGGA

15351 AAACGTTCTT CGGGGCGAAA ACTCTCAAGG ATCTTACCGC TGTTGAGATC

15401 CAGTTCGATG TAACCCACTC GTGCACCCAA CTGATCTTCA GCATCTTTTA

15451 CTTTCACCAG CGTTTCTGGG TGAGCAAAAA CAGGAAGGCA AAATGCCGCA

15501 AAAAAGGGAA TAAGGGCGAC ACGGAAATGT TGAATACTCA TACTCTTCCT

15551 TTTTCAATAT TATTGAAGCA TTTATCAGGG TTATTGTCTC ATGAGCGGAT

15601 ACATATTTGA ATGTATTTAG AAAAATAAAC AAATAGGGGT TCCGCGCACA

15651 TTTCCCCGAA AAGTGCCACC TGAGTCTAAG AAACCATTAT TATCATGACA

15701 TTAACCTATA AAAATAGGCG

**HJP-382-actin5C-FloxSyn21Gal80-10XUAS-dTrpA1**

1 TATCACGAGG CCCTTTCGTC TTCAAGAATT CGTTTATCAC AAGTTTGTAC

51 AAAAAAGCAG GCTCCGCGGC CGCAATTCTA TATTCTAAAA ACACAAATGA

101 TACTTCTAAA AAAAAATCAT GAATGGCATC AACTCTGAAT CAAATCTTTG

151 CAGATGCACC TACTTCTCAT TTCCACTGTC ACATCATTTT TCCAGATCTC

201 GCTGCCTGTT ATGTGGCCCA CAAACCAAGA CACGTTTTAT GGCCATTAAA

251 GCTGGCTGAT CGTCGCCAAA CACCAAATAC ATAATGAATA TGTACACATT

301 CGAGAAAGAA GCGATCAAAG AAGCGTCTTC GGGCGGAGTA GGAGAATGCG

351 GAGGAGAAGG AGAACGAGCT GATCTAGTAT CTCTCCACAA TCCAATGCCA

401 ACTGACCAAC TGGCCATATT CGGAGCAATT TGAAGCCAAT TTCCATCGCC

451 TGGCGATCGC TCCATTCTTG GCTATATGTT TTTCACCGTT ACCCGGGGCC

501 ATTTTCAAAG ACTCGTCGGC AAGATAAGAT TGTGTCACTC GCTGTCTCTC

551 TTCATTTGTC GAAGAATGCT GAGGAATTTC GCGATGACGT CGGCGAGTAT

601 TTTGAAGAAT GAGAATAATT TGTATTTATA CGAAAATCAG TTAGTGGAAT

651 TTTCTACAAA AACATGTTAT CTATAGATAA TTTTGTTGCA AAATATGTTG

701 ACTATGACAA AGATTGTATG TATATACCTT TAATGTATTC TCATTTTCTT

751 ATGTATTTAT AATGGCAATG ATGATACTGA TGATATTTTA AGATGATGCC

801 AGACCAAAAG GCTTGAATTT CTGCGTCTTT TGCCGAACGC AGTGCATGTG

851 CAATTGTTGT TTTTTGGAAT ATTCAATTTT CGGACTGTCC GCTTTGATTT

901 CAGTTTCTTG GCTTATTCAA AAAGCAAAGT AAAGCCAAAA AAGCGAGATG

951 GCAATACCAA ATGCGGCAAA ACGGTAGTGG AAGGAAAGGG GTGCGGGGCA

1001 GCGGAAGGAA GGGTGGGGCG GGGCGTGGCG GGGTCTGTGG CTGGGCGCGA

1051 CGTCACCGAC GTTGGAGCCA CTCCTTTGAC CATGTGTGCG TGTGTGTATT

1101 ATTCGTGTCT CGCCACTCGC CGGTTGTTTT TTTCTTTTTA TGCTGCGCTC

1151 TCTCTAGCGC CATCTCGCTT ACGCATGCTC AACGCACCGC ATGTTGCCGT

1201 TTCCTTTTAT GCGTCATTTT GGCTCGAAAT AGGCAATTAT TTAAACAAAG

1251 ATTAGTCAAC GAAAACGCTA AAATAAATAA GTCTACAATA TGGTTACTTA

1301 TTGCCATGTG TGTGCAGCCA ACGATAGCAA CAAAAGCAAC AACACAGGTG

1351 GCTTTCCCTC TTTCACTTTT TGTTTGCAAG CCGCGTGCGA GCAAGACGGC

1401 ACGACCGGCA AACGCAATTA CGCTGACAAA GAGCAGACGA AGTTTTGGCG

1451 AAAAACATCA AGGCGCCTGA TACGAATGCA TTTGCAATAA CAATTGCGAT

1501 ATTTAATATT GTTTATGAAG CTGTTTGACT TCAAAACACA CAAAAAAAAA

1551 AATAAAACAA ATTATTTGAA AGAGAATTAG GAATCGGACG CTTATCGTTA

1601 GGGTAACAAC AAGAAATGCT TACTGAGTCA CAGCCTCTGG AAAACTGCCG

1651 CAAGCCAGAG AGAGAGAGAA AAAGAGGGAG AGCAGCTTAG ACCGCATGTG

1701 CTTGTGTGTG AGGCGTCTCT CTCTTCGTCT CTGTTGCGCA AACGCATAGA

1751 CTGCACTGAA AAAATCGATT ACCTATTTTT TATGAATGAA TATTTGCACT

1801 ATTACTATTC AAAACTATTA AGATAGCAAT CACATTCAAT AGCCAAATAC

1851 TATACCACCT GAGCGATGCA ACGAAATGAT CAATTTGAGC AAAAATGCTG

1901 CATATTTAGG ACGGCATCAT TATAGAAATG CTTCTTGCTG TGTACTTTTC

1951 TCTCGTCTGG CAGCTGTTTC GCCGTTATTG TTAAAACCGG CTTAAGTTAG

2001 GTGTGTTTTC TACGACTAGT GAATGCCCTA CTAGAAGATG TGTGTTGCAC

2051 AAAATGTCCC TGGAATAACC AATTTGAAGT GCAGATAGCA GTAAACGTAA

2101 GCTAATATGA ATATTATTTA ACTGTAATGT TTTAATATCG CTGGACATTA

2151 CTAATAAACC CACTATAAAC ACATGTACAT ATGTATGTTT TGGCATACAA

2201 TGAGTAGTTG GGGAAAAAAT GTGTAAAAGC ACCGTGACCA TCACAGCATA

2251 AAGATAACCA GCTGAAGTAT CGAATATGAG TAACCCCCAA ATTGAATCAC

2301 ATGCCGCAAC TGATAGGACC CATGGAAGTA CACTCTTCAT GGCGATATAC

2351 AAGACACACA CAAGCACGAA CACCCAGTTG CGGAGGAAAT TCTCCGTAAA

2401 TGAAAACCCA ATCGGCGAAC AATTCATACC CATATATGGT AAAAGTTTTG

2451 AACGCGACTT GAGAGCGGAG AGCATTGCGG CTGATAAGGT TTTAGCGCTA

2501 AGCGGGCTTT ATAAAACGGG CTGCGGGACC AGTTTTCATA TCACTACCGT

2551 TTGAGTTCTT GTGCTGTGTG GATACTCCTC CCGACACAAA GCCGCTCCAT

2601 CAGCCAGCAG TCGTCTAATC CAGAGACACC AAACCGAAAG ACTTAATTTA

2651 TATTTATTTA ATTAATTTTA ATAAAACACA CCAAATGTAA GTAGCTTTCC

2701 CCTTCCCAAC AACAAAACAC CATCGAACCA CTCCCACCAA GAAAAAGCAA

2751 TAATCGAGAA AAGCCGCGGA AAATGTGTGA TTTTTTTTGT AAACAAAACA

2801 TTTTTTTATG TGCCAGTGCT GAAAGTGATC AAAAAATACT AGCCACGAGC

2851 TAAAGAGTTA TTGTATTGAC CAAAACTCCA AAAATACCCA AGTTTGGCCC

2901 TAAATTGTCA ATCAAAATAC CAATAGGTCG AAAGACATCA AAATTAACAA

2951 AACCAGGGTT TCAAATACCA TAACTCAAGA ATCAGGATTA CAACTGCAGA

3001 TTTCAGGATA TATACATACA AATTATACGA AATTATAAAA ACCAAAGCAA

3051 TTCAATAGCC CCAACTCAAA TGTTAGGATC TAATATAGTG TTTAAAGCCA

3101 AGCTCGCTGA TGTGGGCGTG TCACGATTTC ACCCAAAGAT ATGCCAAATT

3151 ACGAATTGCA AATCAATTCG CCAACACTTT CTTTTTTTCC CACGCCCTAA

3201 AACACCAGAT CATCCATAAA TGTACATACA TACAGTATAT GCATATTATA

3251 ATCTGTAAAA CTAGATCAGG TTCTTGAAAA TAGTGACGTA GGCAGCCGTT

3301 TTGGCTGAAG CAGAAATTTT TGCCGGTTTT TCAAAGTTGT AGTTGCAAAA

3351 ATGGAGAAAA CCTTCGAGCA TTCGTTCATA TACACACACT CACGCGCAAA

3401 ATAACGAGAG AGAGTGTATG TGTGTGTGAG AGAGCGAAAG CCAGACGACG

3451 GTTTGCTTTT CGCCTCGAAA CATGACCATA TATGGTCACA AAACTTGGCC

3501 GCCGCAATTC AACACACCAG CGCTCTCCTT CGCACCCATA GCGACCATGG

3551 CGCGGAGCGA GCGAGATGGC GAGAGCGAGC GACGCCTATG GCGACGTCGA

3601 CGCAGGCAGC GATTGAAAAA CGCAGTTAAC TGGCATTCAA CATTCACCAG

3651 CCACTTTCAG TCGGTTTATT CCAGTCATTC CTTTCAAACC GTGCGGTCGC

3701 TTAGCTCAGC CTCGCCACTT GCGTTTACAG TAGTTTTCAC GCCTTGAATT

3751 TGTTAAATCG AACAAAAAGG TAAAGTTTAA CTAGCTTTGA AAAGTTTCGT

3801 GGCTCTTAAT TGTTAAATTT TCTAGAGTGC GTTTAGTGTT TTTTTTTTTT

3851 TTTATTTTGT AATGTTAATT TCGGGTTCCA ATTCGAGTTT TAGGCAGCCG

3901 CCATTTTAAG GGCGCATACA CACAGGCAAC TGTGCTCTCT TTGCGGCTTT

3951 CTTTTGCACC GGCATTCGTT AAGTGTCGTC TAGAAGCTTC TCCCCTCCCT

4001 TTTCGGCATA TTCGTATTGT GGTTTTAATT TTTCGGGGCG GGGCCTTCTA

4051 TTTTGTAACT GTTCTTTTAA TTTCTTATTA CAATTCGATC GCAAGTGAAA

4101 ATCAGTTTTC AATCGGAAAA GTATTTTTTT ATGAAATTTT TTTTTGTCCA

4151 AGATTAAAAT TTTGTACTAA AAAAACGTAC ATTGCATTGA GTGATTTTTA

4201 ATTGTACACG AAAAACAAGT TAGTTTGTTA TGACAATTGT ACTTTGGTAG

4251 ACCAGCGCAG TCCAAGGAAA CCACGCAAAT TCTCAGTTTT TTTTTTGCCA

4301 TTTCTACATT ACCAAATAAG GTAACCAAAA ACTAATGGGA AATCCGCATT

4351 CTTTCCATTG CAGCTTACAG GATCGATCCC CGGGTACCGC GCCGACCCAG

4401 CTTTCTTGTA CAAAGTGGTG ATAAACGGCC GGCATAACTT CGTATAATGT

4451 ATGCTATACG AAGTTATCCT GCAGGAACTT AAAAAAAAAA ATCAAAATGG

4501 ATTACAACAA AAGGAGTAGT GTGAGTACGG TGCCGAATGC TGCTCCCATT

4551 CGCGTGGGCT TCGTGGGATT GAACGCGGCT AAGGGTTGGG CCATTAAAAC

4601 GCATTATCCA GCCATACTGC AGCTGAGCTC CCAGTTCCAA ATAACAGCGC

4651 TTTATTCCCC CAAGATCGAG ACGTCCATTG CGACAATTCA GAGGCTGAAG

4701 TTGTCCAACG CCACAGCTTT CCCAACACTC GAGAGCTTCG CCTCGAGCTC

4751 CACGATCGAT ATGATTGTGA TCGCAATCCA GGTGGCTTCC CACTACGAGG

4801 TGGTAATGCC CCTGCTCGAG TTTTCGAAGA ACAATCCTAA TCTGAAGTAT

4851 TTGTTTGTGG AATGGGCACT GGCCTGCTCG CTCGATCAGG CGGAGTCGAT

4901 CTATAAGGCC GCTGCAGAGC GGGGCGTGCA AACAATAATC AGTCTGCAGG

4951 GACGAAAGAG CCCGTACATT TTGCGGGCAA AAGAGCTTAT CTCCCAGGGC

5001 TACATCGGCG ATATAAATAG CATCGAGATT GCAGGTAACG GAGGTTGGTA

5051 CGGTTACGAG CGGCCGGTGA AAAGCCCGAA ATACATTTAC GAGATCGGAA

5101 ACGGAGTTGA TCTGGTGACC ACCACGTTCG GTCACACGAT AGATATATTG

5151 CAGTACATGA CCAGTTCGTA CTTCAGCCGT ATCAATGCCA TGGTGTTTAA

5201 CAACATTCCA GAACAGGAGC TCATTGATGA GAGGGGCAAT CGCCTGGGCC

5251 AGCGGGTCCC AAAGACTGTC CCAGATCATC TCTTGTTCCA GGGCACGCTG

5301 CTCAACGGAA ATGTTCCTGT GTCCTGTTCC TTTAAGGGAG GCAAGCCTAC

5351 GAAAAAGTTC ACCAAGAATC TGGTCATAGA TATCCATGGT ACAAAGGGTG

5401 ATCTGAAGCT GGAGGGTGAC GCTGGATTTG CGGAAATCTC GAATCTCGTT

5451 CTGTATTATT CGGGCACCCG CGCCAATGAT TTTCCCCTGG CCAATGGTCA

5501 GCAGGCACCC TTGGACCCCG GCTACGACGC AGGCAAGGAG ATTATGGAGG

5551 TGTATCACCT CCGAAACTAC AACGCGATCG TTGGTAATAT ACACCGGCTC

5601 TACCAGAGTA TCAGCGATTT CCACTTCAAC ACCAAGAAAA TCCCCGAGCT

5651 TCCATCGCAG TTCGTAATGC AAGGCTTCGA TTTCGAAGGC TTTCCCACCC

5701 TGATGGATGC CTTGATCTTG CATCGCCTGA TTGAAAGTGT CTACAAGTCG

5751 AATATGATGG GCAGTACCCT GAATGTTTCC AACATCTCCC ACTACTCGCT

5801 TTAAACGCGT AGGATCTTTG TGAAGGAACC TTACTTCTGT GGTGTGACAT

5851 AATTGGACAA ACTACCTACA GAGATTTAAA GCTCTAAGGT AAATATAAAA

5901 TTTTTAAGTG TATAATGTGT TAAACTACTG ATTCTAATTG TTTGTGTATT

5951 TTAGATTCCA ACCTATGGAA CTGATGAATG GGAGCAGTGG TGGAATGCCT

6001 TTAATGAGGA AAACCTGTTT TGCTCAGAAG AAATGCCATC TAGTGATGAT

6051 GAGGCTACTG CTGACTCTCA ACATTCTACT CCTCCAAAAA AGAAGAGAAA

6101 GGTAGAAGAC CCCAAGGACT TTCCTTCAGA ATTGCTAAGT TTTTTGAGTC

6151 ATGCTGTGTT TAGTAATAGA ACTCTTGCTT GCTTTGCTAT TTACACCACA

6201 AAGGAAAAAG CTGCACTGCT ATACAAGAAA ATTATGGAAA AATATTTGAT

6251 GTATAGTGCC TTGACTAGAG ATCATAATCA GCCATACCAC ATTTGTAGAG

6301 GTTTTACTTG CTTTAAAAAA CCTCCCACAC CTCCCCCTGA ACCTGAAACA

6351 TAAAATGAAT GCAATTGTTG TTGTTAACTT GTTTATTGCA GCTTATAATG

6401 GTTACAAATA AAGCAATAGC ATCACAAATT TCACAAATAA AGCATTTTTT

6451 TCACTGCATT CTAGTTGTGG TTTGTCCAAA CTCATCAATG TATCTTATCA

6501 TGTCTGGATC CCTAGGATAA CTTCGTATAA TGTATGCTAT ACGAAGTTAT

6551 GGTACCGCTA GTTGGCCACG GGTGCGCATG ATCGTGCTCC TGTCGTTGAG

6601 GACCCGGCTA GGCTGGCGGG GTTGCCTTAC TGGTTAGCAG AATGAATCAC

6651 CGATACGCGA GCGAACGTGA AGCGACTGCT GCTGCAAAAC GTCTGCGACC

6701 TGAGCAACAA CATGAATGGT CTTCGGTTTC CGTGTTTCGT AAAGTCTGGA

6751 AACGCGGAAG TCAGCGCCCT GCACCATTAT GTTCCGGACT AGTTCCAGTG

6801 AAATCCAAGC ATTTTCTAAA TTAAATGTAT TCTTATTATT ATAGTTGTTA

6851 TTTTTGATAT ATATAAACAA CACTATTATG CCCACCATTT TTTTGAGATG

6901 CATCTACACA AGGAACAAAC ACTGGATGTC ACTTTCAGTT CAAATTGTAA

6951 CGCTAATCAC TCCGAACAGG TCACAAAAAA TTACCTTAAA AAGTCATAAT

7001 ATTAAATTAG AATAAATATA GCTGTGAGGG AAATATATAC AAATATATTG

7051 GAGCAAATAA ATTGTACATA CAAATATTTA TTACTAATTT CTATTGAGAC

7101 GAAATGAACC ACTCGGAACC ATTTGAGCGA ACCGAATCGC GCGGAACTAA

7151 CGACAGTCGC TCCAAGGTCG TCGAACAAAA GGTGAATGTG TTGCGGAGAG

7201 CGGGTGGGAG ACAGCGAAAG AGCAACTACG AAACGTGGTG TGGTGGAGGT

7251 GAATTATGAA GAGGGCGCGC GATTTGAAAA GTATGTATAT AAAAAATATA

7301 TCCCGGTGTT TTATGTAGCG ATAAACGAGT TTTTGATGTA AGGTATGCAG

7351 GTGTGTAAGT CTTTTGGTTA GAAGACAAAT CCAAAGTCTA CTTGTGGGGA

7401 TGTTCGAAGG GGAAATACTT GTATTCTATA GGTCATATCT TGTTTTTATT

7451 GGCACAAATA TAATTACATT AGCTTTTTGA GGGGGCAATA AACAGTAAAC

7501 ACGATGGTAA TAATGGTAAA AAAAAAAAAC AAGCAGTTAT TTCGGATATA

7551 TGTCGGCTAC TCCTTGCGTC GGGCCCGAAG TCTTAGAGCC AGATATGCGA

7601 GCACCCGGAA GCTCACGATG AGAATGGCCA GACCCACGTA GTCCAGCGGC

7651 AGATCGGCGG CGGAGAAGTT AAGCGTCTCC AGGATGACCT TGCCCGAACT

7701 GGGGCACGTG GTGTTCGACG ATGTGCAGCT AATTTCGCCC GGCTCCACGT

7751 CCGCCCATTG GTTAATCAGC AGACCCTCGT TGGCGTAACG GAACCATGAG

7801 AGGTACGACA ACCATTTGAG GTATACTGGC ACCGAGCCCG AGTTCAAGAA

7851 GAAGCCGCCA AAGAGCAGGA ATGGTATGAT AACCGGCGGA CCCACAGACA

7901 GCGCCATCGA GGTCGAGGAG CTGGCGCAGG ATATTAGATA TCCGAAGGAC

7951 GTTGACACAT TGGCCACCAG AGTGACCAGC GCCAGGCAGT TGAAGAAGTG

8001 CAGCACTCCG GCCCGCAGTC CGATCATCGG ATAGGCAATC GCCGTGAAGA

8051 CCAGTGGCAC TGTGAGAAAA AGCGGCAATT CGGCAATCGT TTTGCCCAGA

8101 AAGTATGTGT CACAGCGATA AAGTCGACTT CGGGCCTCCC TCATAAAAAC

8151 TGGCAGCTCT GAGGTGAACA CCTAAATCGA ATCGATTCAT TAGAAAGTTA

8201 GTAAATTATT GAAATGCAAA TGTATTCTAA ACATGACTTA CATTTATCGT

8251 GGCAAAGACG TTTTGAAAGG TCATGTTGGT CAGGAAGAGG AAGATGGCTC

8301 CGTTGATATT CATCACACCC ACTTGCGTGA GTTGTTGGCC CAAAAAGATG

8351 AGGCCAATCA AGATGGCAAC CATCTGCAAA TTAAAATGTT ACTCGCATCT

8401 CATTAATATT CGCGAGTTAA ATGAAATTTA TTTATCTTCT GCAAAACTAT

8451 AAACTATACA TCTCATTGAA AAAAACTAAG AAGGGTGTGG AATCAGGCAA

8501 TTCTATCTAA AATCTAGCGA ATTTGTTTCC AAGAATTGTA AGCGTTATAT

8551 CATTTGTTTC CACTGGAACC ACTCACCGTT GTCTGAATAA GTCGCACTTT

8601 TACGAGGAGT GGTTCCTTGA GCACCGACAG CCAGGATCGC CACAGGACCG

8651 CCCGGAACTG CATGAACCAG GTGGCCTTGT AGGTGTACCC ATTCTCCGGC

8701 TGCTCCAGTG GCTTCTCCAG ATTTTTGGTG GCCAACAACT GCTCCATATC

8751 CCGGGCTACT TTGCTAATGG CAAAATTGTC GCATATCTTG GCGATCCGAT

8801 CACGGGACTC GATCTCCCGT CCGGGCACAA CGGCCAACAC CTGTACGTAA

8851 AAGTCCGCCG GATTGTAGTT GGTAGGACAC TGGGCACCCA CGCTGGATAG

8901 GAGTTGAGAT GTTATGTAAT ACTAGATACC CTTAATAAAC ACATCGAACT

8951 CACTAGGAAA AGAAGTCGAC GGCTTCGCTG GGAGTGCCCA AGAAAGCTAC

9001 CCTGCCCTCG GCCATCAGAA GGATCTTGTC AAAGAGCTCA AACAGCTCGG

9051 AAGACGGCTG ATGAATGGTC AGGATGACGG TCTTGCCCTT CTGCGACAGC

9101 TTCTTCAGCA CCTGGACGAC GCTGTGGGCG GTAAAGGAGT CCAGTCCGGA

9151 GGTGGGCTCA TCGCAGATCA GAAGCGGCGG ATCGGTTAGA GCCTCGGAGG

9201 CGAATGCCAG ACGCTTCCTT TCTCCGCCGG ACAGACCTTT CACCCTGCCG

9251 GGCACACCGA TGATCGTGTG CTGACATTTG CTGAGCGAAA GCTCCTGGAT

9301 CACCTGATCC ACGCGGGCCA CTCGCTGCCG ATAGGTCAGA TGTCGTGGCA

9351 TCCGCACCAT GGCTTGGAAA ATCAGGTGTT CCCTGGCCGT TAGGGAGCCG

9401 ATAAAGAGGT CATCCTGCTG GACATAGGCG CACCTGGCCT GCATCTCCTT

9451 GGCGTCCACA GGTTGGCCAT TGAGCAGTCG CATCCCGGAT GGCGATACTT

9501 GGATGCCCTG CGGCGATCGA AAGGCAAGGG CATTCAGCAG GGTCGTCTTT

9551 CCGGCACCGG AACTGCCCAT CACGGCCAAA AGTTCGCCCG GATAGGCCAC

9601 GCCGCAAACT GAGTTTCAAA TTGGTAATTG GACCCTTTAT TAAGATTTCA

9651 CACAGATCAG CCGACTGCGA ATAGAAACTC ACCGTTCTTG AGCAAATGTT

9701 TCCTGGGCGC CGGTATGTGT CGCTCGTTGC AGAATAGTCC GCGTGTCCGG

9751 TTGACCAGCT GCCGCCATCC GGAGCCCGGC TGATTGACCG CCCCAAAGAT

9801 GTCCATATTG TGCCAGGCAT AGGTGAGGTT CTCGGCTAGT TGGCCGCTCC

9851 CTGAACCGGA GTCCTCCGGC GGACTGGGTG GCCGGAGCGT GCCGTAGTTT

9901 TTGGCCTGCC CGAAGCCCTG GTTAATGCAG CTCTGCGAAG CCGCTCCGCT

9951 GTCACCCTGC AATGATAGGG GATCTCAAAT ATCAACTACT AGCGTTATGC

10001 TCATCTAACC CCGAACAAAA AGTACCCCGA AGTATCCTAC GAAGTAGGTT

10051 TATACTTTTA TTTATTTTTT GTGCATCTAG GATCAGCTTA AAATATCTGG

10101 TTGTTATATT TTTTGTAAAA AAGAATATAG TCGAAAATGA ATGCCTTTAG

10151 ATGTCTTGAT CATGATATGA TCTCAAAAAT TGTCTTATAT AGCGAGAACA

10201 GCTACCAGAA TAATCTGTTT CGTGTCACTA TTTGTTTGTG CAATTGCGGT

10251 TTGGGATTTT TGTGGGTCGC AGTTCTCACG CCGCATACAA TTTGATGTTG

10301 CAATCGCAGT TCCTATAGAT CAAGTGAACT TAAGATGTAT GCACATGTAC

10351 TACTCACATT GTTCAGATGC TCGGCAGATG GGTGTTTGCT GCCTCCGCGA

10401 ATTAATAGCT CCTGATCCTC TTGGCCCATT GCCGGGATTT TTCACACTTT

10451 CCCCTGCTTA CCCACCCAAA ACCAATCACC ACCCCAATCA CTCAAAAAAC

10501 AAACAAAAAT AAGAAGCGAG AGGAGTTTTG GCACAGCACT TTGTGTTTAA

10551 TTGATGGCGT AAACCGCTTG GAGCTTCGTC ACGAAACCGC TGACAAAATG

10601 CAACTGAAGG CGGACATTGA CGCTACGTAA CGCTACAAAC GGTGGCGAAA

10651 GAGATAGCGG ACGCAGCGGC GAAAGAGACG GCGATATTTC TGTGGACAGA

10701 GAAGGAGGCA AACAGCGCTG ACTTTGAGTG GAATGTCATT TTGAGTGAGA

10751 GGTAATCGAA AGAACCTGGT ACATCAAATA CCCTTGGATC GAAGTAAATT

10801 TAAAACTGAT CAGATAAGTT CAATGATATC CAGTGCAGTA AAAAAAAAAA

10851 ATGTTTTTTT TATCTACTTT CCGCAAAAAT GGGTTTTATT AACTTACATA

10901 CATACTAGGC GCGCCCATAA GCTTGCATGC CTGCAGGTCG GAGTACTGTC

10951 CTCCGAGCGG AGTACTGTCC TCCGAGCGGA GTACTGTCCT CCGAGCGGAG

11001 TACTGTCCTC CGAGCGGAGT ACTGTCCTCC GAGCGGAGAC TCTAGCCCTA

11051 GGGCATGCCT GCAGGTCGGA GTACTGTCCT CCGAGCGGAG TACTGTCCTC

11101 CGAGCGGAGT ACTGTCCTCC GAGCGGAGTA CTGTCCTCCG AGCGGAGTAC

11151 TGTCCTCCGA GCGGAGACTC TAGCGCTAGC GACGTCGAGC GCCGGAGTAT

11201 AAATAGAGGC GCTTCGTCTA CGGAGCGACA ATTCAATTCA AACAAGCAAA

11251 GTGAACACGT CGCTAAGCGA AAGCTAAGCA AATAAACAAG CGCAGCTGAA

11301 CAAGCTAAAC AATCTGCAGT AAAGTGCAAG TTAAAGTGAA TCAATTAAAA

11351 GTAACCAGCA ACCAAGTAAA TCAACTGCAA CTACTGAAAT CTGCCAAGAA

11401 GTAATTATTG AATACAAGAA GAGAACTCTG AATAGATCTA AAAGGTAGGT

11451 TCAACCACTG ATGCCTAGGC ACACCGAAAC GACTAACCCT AATTCTTATC

11501 CTTTACTTCA GGCGGCCGCC ATGACTTCGG GCGACAAGGA GACTCCTAAG

11551 AGGGAAGATT TCGCCAGTGC ACTGCGATTC TTGATGGGCG GCTGTGCCCG

11601 CGAACCGGAA ATGACTGCAA TGGCGCCGCT TAACCTGCCC AAAAAGTGGG

11651 CACGCATCCT GCGGATGTCC TCGACGCCAA AGATACCAAT CGTGGACTAT

11701 CTGGAGGCGG CTGAGTCCGG AAACCTTGAC GACTTCAAGC GACTCTTCAT

11751 GGCGGACAAC TCGCGCATTG CTTTAAAGGA TGCGAAAGGA CGAACGGCTG

11801 CCCATCAGGC GGCGGCCCGT AATAGGGTTA ACATTTTGCG GTACATTCGC

11851 GACCAGAATG GCGACTTTAA TGCGAAGGAT AATGCCGGCA ATACCCCGCT

11901 CCACATCGCC GTGGAGAGCG ATGCCTACGA CGCTCTGGAC TATCTATTGT

11951 CCATCCCAGT GGATACGGGA GTGCTGAACG AGAAGAAGCA GGCACCAGTG

12001 CACTTGGCCA CCGAGCTGAA CAAAGTGAAG TCCCTTCGGG TGATGGGTCA

12051 GTACCGCAAT GTCATCGATA TTCAGCAGGG CGGCGAACAT GGACGTACAG

12101 CTCTGCACTT GGCCGCCATC TATGATCACG AGGAGTGCGC TCGCATCCTG

12151 ATAACTGAGT TCGATGCATG CCCACGTAAG CCCTGTAACA ATGGTTATTA

12201 TCCCATACAC GAAGCGGCCA AGAATGCCAG CTCCAAGACA ATGGAGGTCT

12251 TCTTCCAGTG GGGCGAGCAG CGCGGCTGCA CCCGCGAGGA GATGATATCC

12301 TTCTACGACT CGGAGGGCAA TGTGCCGCTC CATTCGGCTG TCCATGGTGG

12351 CGACATCAAG GCTGTGGAGC TGTGCCTCAA GTCCGGGGCC AAGATATCTA

12401 CGCAGCAACA CGATCTCTCG ACGCCAGTGC ACCTGGCTTG TGCCCAGGGA

12451 GCCATAGACA TTGTAAAGCT CATGTTCGAG ATGCAGCCAA TGGAGAAGCG

12501 ACTATGTCTG AGTTGCACGG ATGTGCAGAA GATGACGCCG CTGCACTGCG

12551 CCTCCATGTT CGATCATCCG GACATCGTGT CCTATCTGGT AGCCGAGGGA

12601 GCGGACATCA ATGCCCTGGA CAAGGAGCAT CGCTCTCCGT TGCTGCTGGC

12651 GGCATCTCGT AGCGGTTGGA AAACAGTCCA CCTCCTGATT CGCCTGGGGG

12701 CGTGCATTAG TGTCAAGGAC GCCGCCGCCC GCAATGTGCT GCACTTCGTC

12751 ATCATGAACG GCGGCCGGCT GACGGACTTC GCGGAGCAGG TGGCCAATTG

12801 CCAGACGCAG GCGCAGCTGA AGCTGCTGCT CAACGAGAAG GACAGCATGG

12851 GCTGCTCGCC GCTGCACTAC GCCAGTCGGG ATGGGCACAT CCGTTCGTTG

12901 GAGAACCTCA TTCGACTGGG AGCCTGCATC AACCTGAAGA ACAACAACAA

12951 CGAGAGTCCG CTGCACTTTG CCGCACGCTA CGGAAGATAC AATACGGTGC

13001 GGCAGCTCTT GGATTCCGAG AAGGGATCCT TCATCATCAA CGAAAGTGAC

13051 GGTGCAGGGA TGACACCACT GCACATATCC TCGCAGCAAG GACACACGCG

13101 AGTGGTGCAG CTGCTACTCA ATCGAGGAGC CCTGCTCCAT CGGGACCACA

13151 CCGGACGCAA TCCTCTCCAG CTAGCGGCCA TGTCCGGATA CACCGAGACC

13201 ATCGAGCTGC TGCACTCGGT GCACTCGCAT CTGCTCGATC AGGTGGACAA

13251 GGATGGGAAC ACCGCTCTTC ACCTGGCCAC CATGGAGAAT AAGCCCCATG

13301 CGATCTCCGT GCTGATGTCT ATGGGCTGTA AGCTGGTCTA CAACGTTCTG

13351 GACATGAGCG CCATTGACTA TGCCATCTAC TACAAATATC CGGAGGCTGC

13401 CCTGGCCATG GTCACCCACG AGGAGCGGGC CAACGAGGTG ATGGCTCTGC

13451 GTTCCGACAA GCATCCGTGC GTGACCCTCG CCCTAATTGC CTCCATGCCC

13501 AAGGTATTCG AGGCGGTGCA GGACAAGTGC ATTACCAAGG CCAATTGCAA

13551 GAAGGACTCG AAGAGTTTCT ACATCAAATA CTCTTTCGCA TTCTTGCAAT

13601 GCCCCTTTAT GTTTGCCAAG ATTGATGAGA AAACCGGAGA GTCGATTACG

13651 ACCGCCAGTC CCATTCCGTT GCCGGCTTTG AATACCATGG TAACACATGG

13701 CAGGGTGGAG CTGCTGGCCC ATCCGCTCAG TCAGAAGTAT CTGCAGATGA

13751 AGTGGAATTC CTACGGCAAG TACTTTCACC TGGCCAACCT GCTAATCTAC

13801 TCGATATTCC TGGTCTTTGT AACCATCTAC TCTTCGCTGA TGATGAACAA

13851 CATCGAACTG AAGGCTGGGG ACAACAAGAC GATGAGTCAA TACTGCAATA

13901 TGGGATGGGA GCAGCTGACC ATGAATCTCT CGCAGAACCC GTCGGTGGCA

13951 TCACAGATTC GTTTGGATTC CTGCGAGGAG CGTATAAATA GAACCACTGC

14001 AATACTTTTC TGTGCGGTGG TCATCGTGGT CTATATACTG CTCAACTCGA

14051 TGCGGGAACT AATACAGATA TACCAGCAGA AATTGCACTA TATCCTGGAG

14101 ACAGTTAATT TGATATCCTG GGTGCTGTAC ATCTCGGCTT TAGTGATGGT

14151 AACACCGGCA TTTCAGCCGG ATGGAGGAAT CAATACCATT CATTACTCGG

14201 CCGCTTCAAT AGCAGTCTTT CTGTCGTGGT TCCGATTGCT ACTGTTCCTG

14251 CAAAGATTCG ACCAGGTCGG CATCTATGTG GTCATGTTCT TGGAGATTCT

14301 GCAGACGCTC ATTAAAGTGC TGATGGTATT CTCCATACTT ATAATCGCCT

14351 TTGGTCTGGC TTTCTATATA CTACTTTCAA AGATTATTGA CCCCCAACCG

14401 AACCACTTGT CCTTCTCCAA CATACCCATG TCCTTGCTGC GAACTTTCTC

14451 AATGATGCTG GGCGAGCTGG ACTTTGTGGG TACCTATGTG AACACCTACT

14501 ATCGGGATCA GTTGAAAGTG CCCATGACAT CCTTTTTGAT TTTGAGTGTC

14551 TTTATGATCC TTATGCCCAT TCTTCTGATG AACTTGCTCA TCGGTTTGGC

14601 CGTCGGCGAT ATTGAGTCAG TGCGTCGCAA TGCCCAGCTC AAGAGACTGG

14651 CCATGCAGGT GGTGCTCCAC ACGGAGCTGG AGAGGAAGTT GCCCCATGTC

14701 TGGCTGCAGC GAGTTGACAA GATGGAGCTG ATTGAGTATC CCAATGAAAC

14751 CAAGTGCAAG CTGGGCTTCT GCGATTTCAT CCTGCGCAAG TGGTTCTCGA

14801 ATCCATTCAC CGAGGATTCC TCCATGGACG TCATCTCCTT CGACAACAAT

14851 GATGACTACA TCAACGCAGA ATTGGAACGG CAGAGGCGAA AGTTGCGCGA

14901 CATAAGTCGC ATGCTGGAGC AACAGCACCA TCTGGTTCGG CTCATTGTCC

14951 AAAAGATGGA GATCAAGACG GAGGCGGATG ACGTGGACGA GGGTATATCC

15001 CCAAACGAGT TGCGATCCGT CGTCGGTTTG AGATCGGCAG GCGGAAATCG

15051 ATGGAACTCG CCGCGAGTCC GGAATAAACT CCGAGCCGCC CTGAGCTTCA

15101 ATAAGAGCAT GTAGCTCGAG TCTAGAGGAT CTTTGTGAAG GAACCTTACT

15151 TCTGTGGTGT GACATAATTG GACAAACTAC CTACAGAGAT TTAAAGCTCT

15201 AAGGTAAATA TAAAATTTTT AAGTGTATAA TGTGTTAAAC TACTGATTCT

15251 AATTGTTTGT GTATTTTAGA TTCCAACCTA TGGAACTGAT GAATGGGAGC

15301 AGTGGTGGAA TGCCTTTAAT GAGGAAAACC TGTTTTGCTC AGAAGAAATG

15351 CCATCTAGTG ATGATGAGGC TACTGCTGAC TCTCAACATT CTACTCCTCC

15401 AAAAAAGAAG AGAAAGGTAG AAGACCCCAA GGACTTTCCT TCAGAATTGC

15451 TAAGTTTTTT GAGTCATGCT GTGTTTAGTA ATAGAACTCT TGCTTGCTTT

15501 GCTATTTACA CCACAAAGGA AAAAGCTGCA CTGCTATACA AGAAAATTAT

15551 GGAAAAATAT TTGATGTATA GTGCCTTGAC TAGAGATCAT AATCAGCCAT

15601 ACCACATTTG TAGAGGTTTT ACTTGCTTTA AAAAACCTCC CACACCTCCC

15651 CCTGAACCTG AAACATAAAA TGAATGCAAT TGTTGTTGTT AACTTGTTTA

15701 TTGCAGCTTA TAATGGTTAC AAATAAAGCA ATAGCATCAC AAATTTCACA

15751 AATAAAGCAT TTTTTTCACT GCATTCTAGT TGTGGTTTGT CCAAACTCAT

15801 CAATGTATCT TATCATGTCT GGATCACCGG TTATGTTCGG CTTGTCGACA

15851 TGCCCGCCGT GACCGTCGAG AACCCGCTGA CGCTGCCCCG CGTATCCGCA

15901 CCCGCCGACG CCGTCGCACG TCCCGTGCTC ACCGTGACCA CCGCGCCCAG

15951 CGGTTTCGAG GGCGAGGGCT TCCCGGTGCG CCGCGCGTTC GCCGGGATCA

16001 ACTACCGCCA CCTCGACCCG TTCATCATGA TGGACCAGAT GGGTGAGGTG

16051 GAGTACGCGC CCGGGGAGCC CAAGGGCACG CCCTGGCACC CGCACCGCGG

16101 CTTCGAGACC GTGACCTACA TCGTCGACGG TAACATGTGA GCAAAAGGCC

16151 AGCAAAAGGC CAGGAACCGT AAAAAGGCCG CGTTGCTGGC GTTTTTCCAT

16201 AGGCTCCGCC CCCCTGACGA GCATCACAAA AATCGACGCT CAAGTCAGAG

16251 GTGGCGAAAC CCGACAGGAC TATAAAGATA CCAGGCGTTT CCCCCTGGAA

16301 GCTCCCTCGT GCGCTCTCCT GTTCCGACCC TGCCGCTTAC CGGATACCTG

16351 TCCGCCTTTC TCCCTTCGGG AAGCGTGGCG CTTTCTCATA GCTCACGCTG

16401 TAGGTATCTC AGTTCGGTGT AGGTCGTTCG CTCCAAGCTG GGCTGTGTGC

16451 ACGAACCCCC CGTTCAGCCC GACCGCTGCG CCTTATCCGG TAACTATCGT

16501 CTTGAGTCCA ACCCGGTAAG ACACGACTTA TCGCCACTGG CAGCAGCCAC

16551 TGGTAACAGG ATTAGCAGAG CGAGGTATGT AGGCGGTGCT ACAGAGTTCT

16601 TGAAGTGGTG GCCTAACTAC GGCTACACTA GAAGAACAGT ATTTGGTATC

16651 TGCGCTCTGC TGAAGCCAGT TACCTTCGGA AAAAGAGTTG GTAGCTCTTG

16701 ATCCGGCAAA CAAACCACCG CTGGTAGCGG TGGTTTTTTT GTTTGCAAGC

16751 AGCAGATTAC GCGCAGAAAA AAAGGATCTC AAGAAGATCC TTTGATCTTT

16801 TCTACGGGGT CTGACGCTCA GTGGAACGAA AACTCACGTT AAGGGATTTT

16851 GGTCATGAGA TTATCAAAAA GGATCTTCAC CTAGATCCTT TTAAATTAAA

16901 AATGAAGTTT TAAATCAATC TAAAGTATAT ATGAGTAAAC TTGGTCTGAC

16951 AGTTACCAAT GCTTAATCAG TGAGGCACCT ATCTCAGCGA TCTGTCTATT

17001 TCGTTCATCC ATAGTTGCCT GACTCCCCGT CGTGTAGATA ACTACGATAC

17051 GGGAGGGCTT ACCATCTGGC CCCAGTGCTG CAATGATACC GCGAGACCCA

17101 CGCTCACCGG CTCCAGATTT ATCAGCAATA AACCAGCCAG CCGGAAGGGC

17151 CGAGCGCAGA AGTGGTCCTG CAACTTTATC CGCCTCCATC CAGTCTATTA

17201 ATTGTTGCCG GGAAGCTAGA GTAAGTAGTT CGCCAGTTAA TAGTTTGCGC

17251 AACGTTGTTG CCATTGCTAC AGGCATCGTG GTGTCACGCT CGTCGTTTGG

17301 TATGGCTTCA TTCAGCTCCG GTTCCCAACG ATCAAGGCGA GTTACATGAT

17351 CCCCCATGTT GTGCAAAAAA GCGGTTAGCT CCTTCGGTCC TCCGATCGTT

17401 GTCAGAAGTA AGTTGGCCGC AGTGTTATCA CTCATGGTTA TGGCAGCACT

17451 GCATAATTCT CTTACTGTCA TGCCATCCGT AAGATGCTTT TCTGTGACTG

17501 GTGAGTACTC AACCAAGTCA TTCTGAGAAT AGTGTATGCG GCGACCGAGT

17551 TGCTCTTGCC CGGCGTCAAT ACGGGATAAT ACCGCGCCAC ATAGCAGAAC

17601 TTTAAAAGTG CTCATCATTG GAAAACGTTC TTCGGGGCGA AAACTCTCAA

17651 GGATCTTACC GCTGTTGAGA TCCAGTTCGA TGTAACCCAC TCGTGCACCC

17701 AACTGATCTT CAGCATCTTT TACTTTCACC AGCGTTTCTG GGTGAGCAAA

17751 AACAGGAAGG CAAAATGCCG CAAAAAAGGG AATAAGGGCG ACACGGAAAT

17801 GTTGAATACT CATACTCTTC CTTTTTCAAT ATTATTGAAG CATTTATCAG

17851 GGTTATTGTC TCATGAGCGG ATACATATTT GAATGTATTT AGAAAAATAA

17901 ACAAATAGGG GTTCCGCGCA CATTTCCCCG AAAAGTGCCA CCTGAGTCTA

17951 AGAAACCATT ATTATCATGA CATTAACCTA TAAAAATAGG CG
