# Supplementary material for: Cre-assisted fine-mapping of neural circuits using orthogonal split inteins
Source: eLife. 2020 Apr 14;9:e53041. doi: 10.7554/eLife.53041 (PMC7217698; doi:10.7554/eLife.53041)
Supplement: Transparent reporting form [file elife-53041-transrepform.docx]

***eLife’s* transparent reporting form**

We encourage authors to provide detailed information *within their submission* to facilitate the interpretation and replication of experiments. Authors can upload supporting documentation to indicate the use of appropriate reporting guidelines for health-related research (see [EQUATOR Network](http://www.equator-network.org/%20)), life science research (see the [BioSharing Information Resource](https://biosharing.org/)), or the [ARRIVE guidelines](http://www.plosbiology.org/article/info:doi/10.1371/journal.pbio.1000412) for reporting work involving animal research. Where applicable, authors should refer to any relevant reporting standards documents in this form.

If you have any questions, please consult our Journal Policies and/or contact us: [editorial@elifesciences.org](mailto:editorial@elifesciences.org).

**Sample-size estimation**

- You should state whether an appropriate sample size was computed when the study was being designed
- You should state the statistical method of sample size computation and any required assumptions
- If no explicit power analysis was used, you should describe how you decided what sample (replicate) size (number) to use

Please outline where this information can be found within the submission (e.g., sections or figure legends), or explain why this information doesn’t apply to your submission:

Two experiments described in the Results involve examination of multiple samples: the 15 crosses conducted to demonstrate variability of the TH-Gal4 subpatterns (Fig. 2) and the SparCLIn crosses used to establish the essential role of the PE^RK^ neurons in proboscis extension (PE; pp. 13). In the latter case, the correlation between PE^RK^ neuron presence (or absence) in the expression pattern and the presence (or absence) of a PE phenotype was 1 for a sample size of n=74, thus vastly exceeding the probability of this outcome expected by chance (e.g. sample size=3 using the criteria alpha=0.001, beta=0.99: <https://www2.ccrb.cuhk.edu.hk/stat/other/correlation.htm#2>) and obviating the need for an independent estimate of sample size. For the TH-Gal4 results, the principal goal was to establish the existence of variability in the expression patterns produced by SpaRCLIn rather than to quantitatively compare the frequencies of each category of expression within or across groups (i.e. intersections). The sample sizes (n=9-18) conformed with general practice for similar studies, and the outcomes sufficiently demonstrated the fact of variability, while at the same time demonstrating a degree of repeatability well above chance levels. We believe that this level of analysis is sufficient information for readers to assess the capability of the system.

**Replicates**

- You should report how often each experiment was performed
- You should include a definition of biological versus technical replication
- The data obtained should be provided and sufficient information should be provided to indicate the number of independent biological and/or technical replicates
- If you encountered any outliers, you should describe how these were handled
- Criteria for exclusion/inclusion of data should be clearly stated
- High-throughput sequence data should be uploaded before submission, with a private link for reviewers provided (these are available from both GEO and ArrayExpress)

Please outline where this information can be found within the submission (e.g., sections or figure legends), or explain why this information doesn’t apply to your submission:

The number of biological replicates (i.e. the number of animals or CNS preparations of a given genotype) for each experiment is given either in the text of the Results section where the experiment is described or in the corresponding figure/figure legend.

**Statistical reporting**

- Statistical analysis methods should be described and justified
- Raw data should be presented in figures whenever informative to do so (typically when N per group is less than 10)
- For each experiment, you should identify the statistical tests used, exact values of N, definitions of center, methods of multiple test correction, and dispersion and precision measures (e.g., mean, median, SD, SEM, confidence intervals; and, for the major substantive results, a measure of effect size (e.g., Pearson's r, Cohen's d)
- Report exact p-values wherever possible alongside the summary statistics and 95% confidence intervals. These should be reported for all key questions and not only when the p-value is less than 0.05.

Please outline where this information can be found within the submission (e.g., sections or figure legends), or explain why this information doesn’t apply to your submission:

As noted above, the n values for each experiment are provided in the figures. The standard deviation of cell counts for the TH-Gal4 experiments are also reported in Fig. 2.

(For large datasets, or papers with a very large number of statistical tests, you may upload a single table file with tests, Ns, etc., with reference to sections in the manuscript.)

**Group allocation**

- Indicate how samples were allocated into experimental groups (in the case of clinical studies, please specify allocation to treatment method); if randomization was used, please also state if restricted randomization was applied
- Indicate if masking was used during group allocation, data collection and/or data analysis

Please outline where this information can be found within the submission (e.g., sections or figure legends), or explain why this information doesn’t apply to your submission:

The only experimental groups of importance in the manuscript are those generated to identify Rk-expressing neurons required for proboscis extension (PE). Flies were grouped first on the basis of their PE activation phenotypes without knowledge of their expression patterns, which were naturally randomized by the SparCLIn method and secondarily determined by immunohistochemistry.

**Additional data files (“source data”)**

- We encourage you to upload relevant additional data files, such as numerical data that are represented as a graph in a figure, or as a summary table
- Where provided, these should be in the most useful format, and they can be uploaded as “Source data” files linked to a main figure or table
- Include model definition files including the full list of parameters used
- Include code used for data analysis (e.g., R, MatLab)
- Avoid stating that data files are “available upon request”

Please indicate the figures or tables for which source data files have been provided:

Source data for the TH-Gal4 intersections shown in Fig. 2I are provided (i.e. Figure2I.xlsx)
